# Synthesis of Nonsymmetrically Substituted 2,3-Dialkoxyphenazine Derivatives and Preliminary Examination of Their Cytotoxicity

**DOI:** 10.1021/acs.joc.2c01901

**Published:** 2023-01-13

**Authors:** Paweł Ręka, Jarosław Grolik, Katarzyna M. Stadnicka, Maria Kołton-Wróż, Paweł Wołkow

**Affiliations:** † Department of Organic Chemistry, Faculty of Chemistry, Jagiellonian University, Gronostajowa 2, 30-387 Kraków, Poland; ‡ Department of Crystal Chemistry and Crystal Physics, Faculty of Chemistry, Jagiellonian University, Gronostajowa 2, 30-387 Kraków, Poland; § Center for Medical Genomics—OMICRON, Jagiellonian University Medical College, Kopernika 7c, 31-034 Kraków, Poland

## Abstract

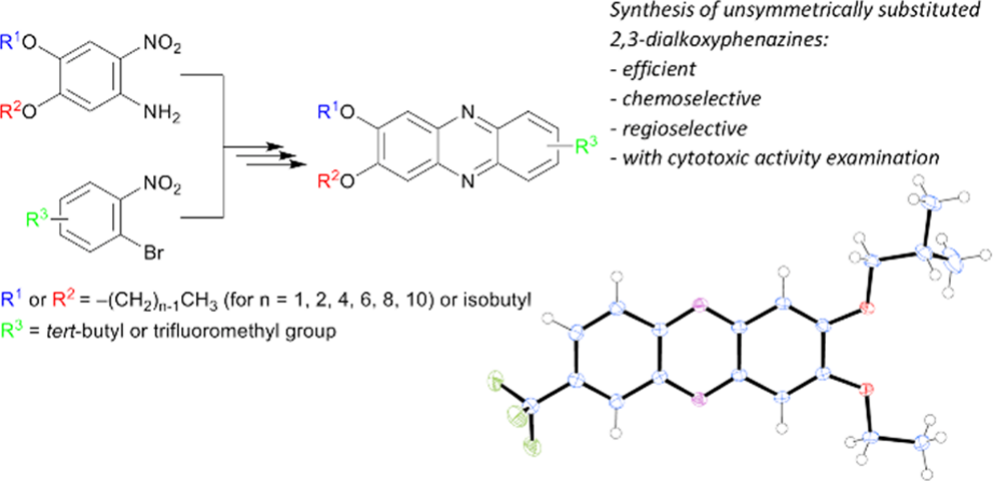

Fourteen new 2,3-dialkoxyphenazine derivatives with two different alkoxy groups bearing R^1^ and R^2^ alkyl chains, defined as −CH_2_CH(CH_3_)_2_ and −(CH_2_)_*n*−1_CH_3_ for *n* = 1, 2, 4, 6, 8, and 10, were prepared *via* regioselective synthesis. The applied synthetic protocol is based on the following reactions: the Buchwald–Hartwig coupling of a nonsymmetrically substituted 4,5-dialkoxy-2-nitroaniline with a 1-bromo-2-nitrobenzene derivative featuring additional *tert*-butyl, trifluoromethyl or two methoxy groups; the reduction of bis(2-nitrophenyl)amine; and a final step of tandem-like oxidation that leads to the preparation of a heterocyclic phenazine system. The regioselectivity of these steps and the molecular structure of the compounds under investigation were confirmed by nuclear magnetic resonance and additionally by single-crystal X-ray diffraction performed for some examples of **5** and **6** phenazine series. For 7-(*tert*-butyl)-3-isobutoxy-2-(octyloxy)phenazine (**5f**), 3-(hexyloxy)-2-isobutoxy-7-(trifluoromethyl)phenazine (**6e**), and 2,3-bis(hexyloxy)-7,8-dimethoxyphenazine (**7**), viability and cytotoxicity assays were performed on the LoVo human colon adenocarcinoma cell line, with **5f** confirmed to exhibit cytotoxicity.

## Introduction

**Figure 1:**
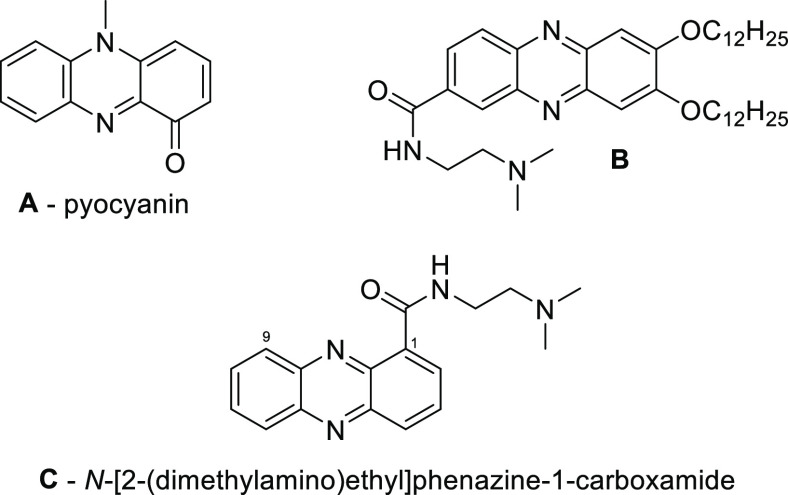
Examples of natural and synthetic phenazine derivatives.

Of the more than 6000 compounds reported to feature a phenazine system described in the literature,^1^ several hundreds have been reported to exhibit biological activity, such as antibacterial, antiparasitic, neuroprotective, insecticidal, anti-inflammatory,^2^ antifungal,^3^ and antitumor properties.^4^ The first known example of a phenazine-based natural product was pyocyanin (Figure 1, example **A**), which is the characteristic blue pigment produced by *Pseudomonas aeruginosa* present on human skin, blue pus, and on certain other materials.^5^ Although this pigment was extracted from a colony of this microorganism in 1860 by Fordos,^5^ the structure of pyocyanin remained unknown until the first half of the 20th century. The biological properties of pyocyanin^6^ and those of other phenazine derivatives isolated later from natural products (such as the new family of phenazines referred as dermacozines^7^ described in 2010) were found to be important in drug research^2^ and became the leading structures in the synthesis of compounds that exhibit antitumor^8^ or antibiotic^9^ properties. The antitumor activity of phenazines is usually associated with topoisomerase inhibition^10^ and DNA intercalation,^11^ but a more specific interaction with proteins could also be responsible for their activity.^12^ The antitumor properties of phenazines containing long alkyl or alkoxy groups at their C-2 and C-3 positions, similar to the structures presented in this article, have been described in the literature.^12,13^ Among these examples, there are 7,8-didodecylphenazine-2,3-diamine, which is important in the new therapeutic strategy of castration-resistant prostate cancer treatment,^12^ and 2,3-dialkoxyphenazine substituted at the C-7 position (Figure 1, example **B**), the derivatives of which have known antitumor properties.^13^ Moreover, the derivatives are potential new drug candidates for use in pancreatic cancer therapy.^14^ Compounds with a phenazine core structure have also been found to exhibit such properties as the formation of liquid crystals,^15^ mechanochromism in a dyad with phenothiazine,^16^ oxidation-sensitive fluorescence that allows selective hypochlorite ion detection,^17^ and reductive biomolecule monitoring and imaging.^18^ Also, many other applications of phenazines have been reported, such as their use in pesticides,^19^ in optical sensing,^20^ asymmetric electrocatalysis,^21^ electrochemical sensing and biosensing,^22^ aqueous organic redox flow batteries,^23^ organic LEDs,^24^ and organic semiconductors.^25,26^

The improvement of the desired properties of such compounds *via* precise and nonsymmetric substitution is important in the synthesis of derivatives with excellent biological activity. For example, a significant increase in antitumor activity can be induced in *N*-[2-(dimethylamino)ethyl] phenazine-1-carboxamide (Figure 1, example **C**) through the introduction of an alkoxy group at the C-9 position. The compound **C** has been shown to increase life span (ILS) in a mouse Lewis lung carcinoma model to 57% for a dose of 150 mg kg^–1^. Moreover, when the 9-methoxy derivative^27,28^ was used, the ILS was 128% for a dose of 100 mg kg^–1^.

From the methods used to prepare the alkoxy-phenazine derivatives,^29^ the most frequent are those based on the cyclocondensation of substituted *o*-phenylenediamines with some *o*-quinones, similar to the procedure first described by Kehrmann and Memod.^30^ In these methods, alkoxy groups can be introduced using substituted *o*-phenylenediamine^31,32^ or *o*-quinone^33^ and also by alkylation of hydroxyphenazines.^13^ It is impossible to obtain nonsymmetrically substituted compounds in a chemoselective way using Wohl–Aue,^34^ Nietzki–Ernst,^35^ Waterman–Vivian,^36^ or the previously described synthetic procedures. The best methods that allow control of ring substitution are the Buchwald–Hartwig^37^ reaction and the Ecker–Steiner^38^ method, especially when mild oxidants are used.^39^

**Figure 2:**
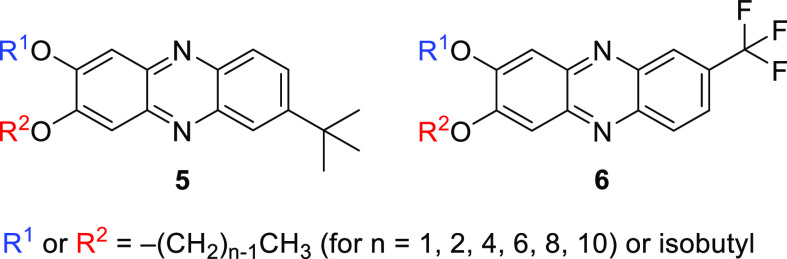
Structures of the obtained series **5** and **6** phenazine derivatives.

In our study, the developed synthetic procedure allows for the synthesis of the designed regioisomer with the positions of substituents depending on the structure of the applied substrates. In this procedure, nonsymmetrically substituted 4,5-dialkoxy-2-nitroanilines are coupled with the bromo-2-nitrobenzene derivatives and then two nitro groups are reduced to amines to prepare a phenazine under conditions similar to those described by Tomlinson.^39^ In this way, 14 new 2,3-dialkoksyphenazine derivatives were obtained, the structures of which are shown in Figure 2.

## Results and Discussion

### General Procedure for the Synthesis of Substituted 2,3-Dialkoxyphenazines (Scheme 1)

The main idea for the synthesis of nonsymmetrically substituted phenazines came from the recently described method for the transetherification of 4,5-dialkoxy-2-nitroanilines,^40^ which allows for efficient regioselective substitution of the alkoxy chain at the C-5 position (i.e., in the para position to the nitro group). Unsymmetrically substituted nitroanilines are then coupled with 1-bromo-2-nitrobenzene derivatives *via* the Buchwald–Hartwig reaction. In this research, 1-bromo-2-nitro-4-(trifluoromethyl)benzene and 2-bromo-4-(*tert*-butyl)-1-nitrobenzene were chosen as the examples of compounds with substituents having different impacts on the biological activity of the final product. A series of bis(2-nitrophenyl)amine derivatives were synthesized by Buchwald–Hartwig coupling and then converted to phenazines *via* reduction followed by tandem-like oxidation under mild conditions using ferric chloride. The yields of the Buchwald–Hartwig coupling and phenazine synthesis are shown in Table 1.

**Table 1:** Yields of Buchwald–Hartwig Coupling and Phenazine Formation

compound	R^1^^a^	R^2^^a^	yield of **2** (%)	yield of **3** (%)	yield of **5** (%)	yield of **6** (%)
**a**	*n* = 1	–^*i*^Bu	92	62	81	61
**b**	*n* = 2	–^*i*^Bu	95	79	82	79
**c**	–^*i*^Bu	*n* = 2	96	71	88	69
**d**	*n* = 4	–^*i*^Bu	95	50	55	65
**e**	*n* = 6	–^*i*^Bu	77	75	77	70
**f**	*n* = 8	–^*i*^Bu	96	63	65	67
**g**	*n* = 10	–^*i*^Bu	78	34	77	60

a R^1^ and R^2^ = −(CH_2_)_*n–*1_CH_3_ (*n* = 1, 2, 4, 6, 8, and 10) or isobutyl (−^*i*^Bu).

### Coupling of Nitroanilines with 1-Bromo-2-nitrobenzenes

**Scheme 1:**
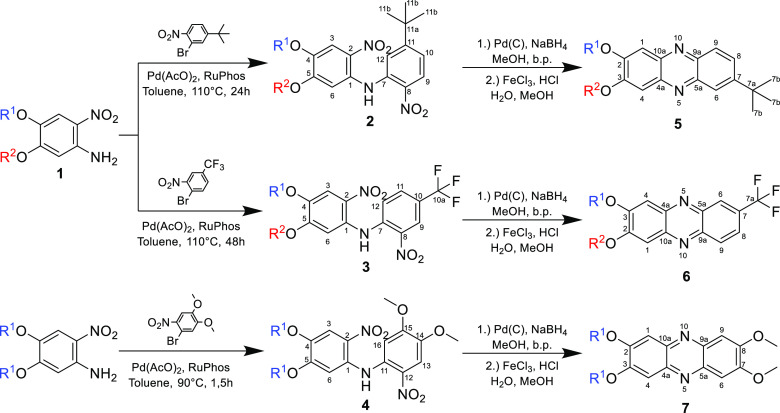
General Synthesis Scheme of 2,3-Dialkoxyphenazine Derivatives from 4,5-Dialkoxy-2-nitroaniline; R^1^ and R^2^ can be Straight Alkyl Chains −(CH_2_)_*n*−1_CH_3_ (*n* = 1, 2, 4, 6, 8, and 10) or Isobutyl Groups. For 4 and 5, R^1^ = R^2^ = *n*-Hexyl Groups

Compounds **2** and **3** were synthesized *via* the Buchwald–Hartwig reaction. This reaction allows for the selective formation of a new N–C-7 bond in compounds **2** and **3** (Scheme 1), which determines the mutual position of the substituents in the nitroaniline and benzene rings. 1-Bromo-2-nitrobenzenes with *tert*-butyl (electron-donating) and trifluoromethyl (electron-withdrawing) groups were chosen due to the different impact on the benzene ring reactivity. The reaction time was increased from 24 to 48 h when a compound substituted with a trifluoromethyl group was used instead of a *tert*-butyl group. The decrease in the reaction rate was caused by the deactivation of the aromatic system due to the presence of an electron-withdrawing trifluoromethyl group. The opposite effect was even stronger for 1-bromo-4,5-dimethoxy-2-nitrobenzene (**4**), where the two electron-donating groups allowed the reaction time to be reduced to 1.5 h at a lower temperature. Buchwald–Hartwig coupling is an effective method in the synthesis of bis(2-nitrophenyl)amine derivatives and can be applied for a wide range of compounds.^41^

### Synthesis of Phenazines from Bis(2-nitrophenyl)amines

**Scheme 2:**
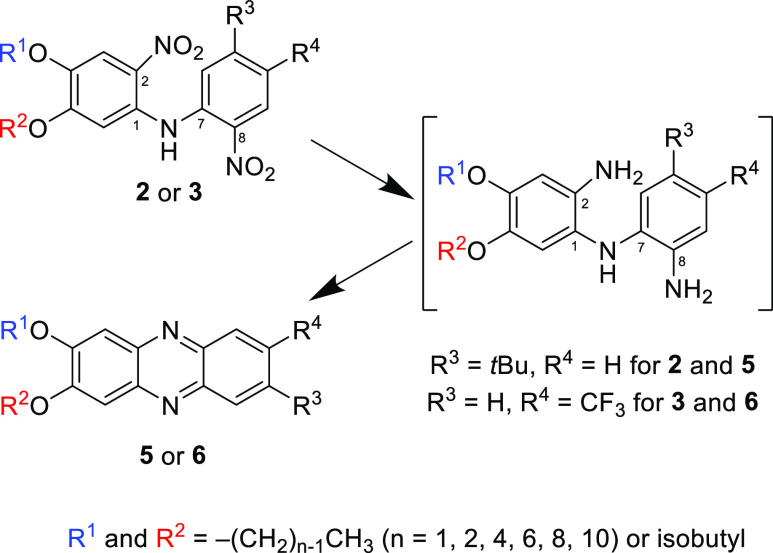
Synthesis of Phenazines from Bis(2-nitrophenyl)amine Derivatives

The final compounds were synthesized *via* a two-step procedure carried out in a tandem-like scheme, to avoid uncontrolled oxidation of the intermediates in air (Scheme 2). Reduction was carried out with palladium on a charcoal catalyst with sodium tetrahydroborate, which was used as a hydrogen source instead of gaseous hydrogen. The reaction was carried out by the slow addition of NaBH_4_ powder to a gently boiling solution of substrate **2** or **3** in the presence of a catalyst, until the solution became colorless, as described in the procedure reported earlier.^42,43^ The reaction mixture was then filtered through a pad of silica gel directly into a flask containing dilute hydrochloric acid to minimize the oxidation of the highly reactive amine intermediates by air. The solution was then concentrated under vacuum, diluted with the additional amount of hydrochloric acid, and stirred with ferric chloride overnight. Details of the procedure are described in the Experimental Section.

Using ferric chloride as a mild oxidation agent is also important for reaction regioselectivity.^44^ The formation of a ferric complex with amines implies the phenazine ring closure in a specific position (Scheme 2). Only one regioisomer is formed, and the position of the alkoxy groups in the phenazines (**5** or **6**) is determined by the substitution of the substrate (**2** or **3**). The use of oxidizing reagents, which are usually effective in the synthesis of phenazines,^44^ results in the formation of a mixture in which, aside from the desired product, the formation of other aminophenazine compounds is also possible.

### Nuclear Magnetic Resonance

To analyze the structures of all described compounds, 1D and 2D nuclear magnetic resonance (NMR) spectra were recorded on a Bruker AVANCE III 300 MHz spectrometer using deuterated chloroform as the solvent and standard reference for ^13^C NMR with tetramethylsilane as the standard reference substance for proton (^1^H) NMR spectroscopy. For compounds **3** and **6**, fluorine (^19^F) NMR spectra were also recorded using hexafluorobenzene (−162.9 ppm) as a reference. For selected representative examples, **2b**, **3b**, **5b**, and **6b**, heteronuclear single quantum coherence (HSQC) and heteronuclear multiple bond correlation (HMBC) spectra were also measured to assign the signals of the carbon atoms and to confirm the positions of the alkoxy groups. The presence of solvents used in chromatography and crystallization is visible in some of the ^1^H spectra in the form of a singlet at 5.30 ppm, a singlet at 1.56 ppm, and multiple signals between 0.80 and 1.50 ppm, corresponding to dichloromethane, water, and hexanes, respectively.

Analysis of the splitting patterns, coupling constants, and the influence of two electron-withdrawing nitro groups on the chemical shifts of the attached *ortho* hydrogen for compounds **2** allows to distinguish unambiguously the signals for protons H-3, H-6, H-9 (∼8.15 ppm, d, ^3^*J*_*o*,H9–H10_ ≈ 9 Hz), and H-12 (∼7.59 ppm, d, ^4^*J*_*m*,H10–H12_ ≈ 2 Hz). A doublet of doublets can be attributed to proton H-10 (∼7.10 ppm, dd, ^3^*J*_*o*,H9–H10_ ≈ 9 Hz, ^4^*J*_*m*,H10–H12_ ≈ 2 Hz), as a result of its coupling with H-9 and H-12. The position of the alkoxy chains can be assigned by correlations of protons H-4a and H-5a with C-4 and C-5, respectively, in the HMBC spectra of **2b** (Figures S12 and S13). In the HMBC spectra, correlations of carbon C-11a with the *tert*-butyl group featuring H-10 and H-12 also confirm the position of the *tert*-butyl group at C-11. The NMR assignment for **2** and **3** series was performed in an analogy to **2b** spectra.

The splitting patterns for compounds **3** are very similar to those of **2**, with different chemical shifts for H-9 (8.48 ppm, d, ^4^*J*_*m*,H9–H11_ = 2.2 Hz), H-11 (7.67 ppm, dd, ^3^*J*_*o*,H11–H12_ = 8.75 Hz, ^4^*J*_*m*,H9–H11_ = 2.2 Hz), and H-12 (7.54 ppm, d, ^3^*J*_*o*,H11–H12_ = 8.75 Hz) due to the presence of the additional electron-withdrawing trifluoromethyl substituent at position C-10 instead of *tert*-butyl at C-11. The ^19^F NMR spectra of compounds **3** show a singlet with a chemical shift at around −63.4 ppm, which confirms the presence of the trifluoromethyl group in the compounds. An additional coupling of ^13^C to ^19^F splits the signals of C-10a in the ^13^C NMR of compounds **3** into quartets, and as a result, the intensity of these signals decreases, meaning that they are not observed in the spectra.

In the ^1^H NMR spectra of phenazines **5** and **6**, protons H-6, H-8, and H-9 can be assigned in the same way as for **2** and **3**, but due to aromatic ring symmetry, the signals from H-1 and H-4 cannot be distinguished, and this prevented the assignment of alkoxy substituents. The integration of H-2a and H-3a in **5** and **6** confirms the presence of two different alkoxy groups in these compounds, but their positions cannot be unequivocally assigned.

**Figure 3:**
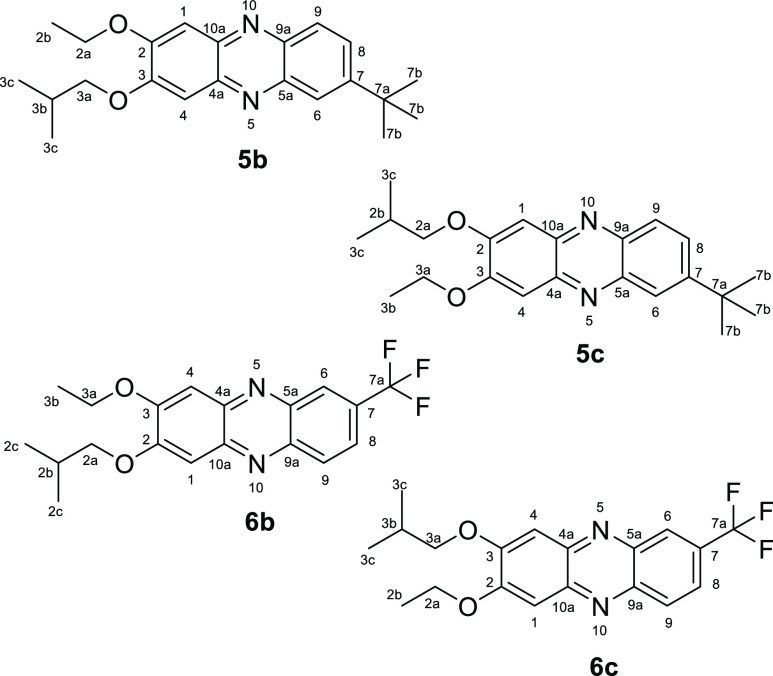
Comparation of the inversely substituted isomers of **5** and **6**.

The distinction of isomers **5b** from **5c** and **6b** from **6c** (Figure 3) was achieved by analysis of differences in the “fingerprint” region of the collected infrared (IR) spectra (Figures S91 and S92 for **5b**/**5c**, Figures S127 and S128 for **6b**/**6c**). To prove that only one proper regioisomer was formed in the reaction, single-crystal X-ray analysis was performed for the crystalline phases of the representative compounds of **5** and **6** series.

### Crystal Structure

**Figure 4:**
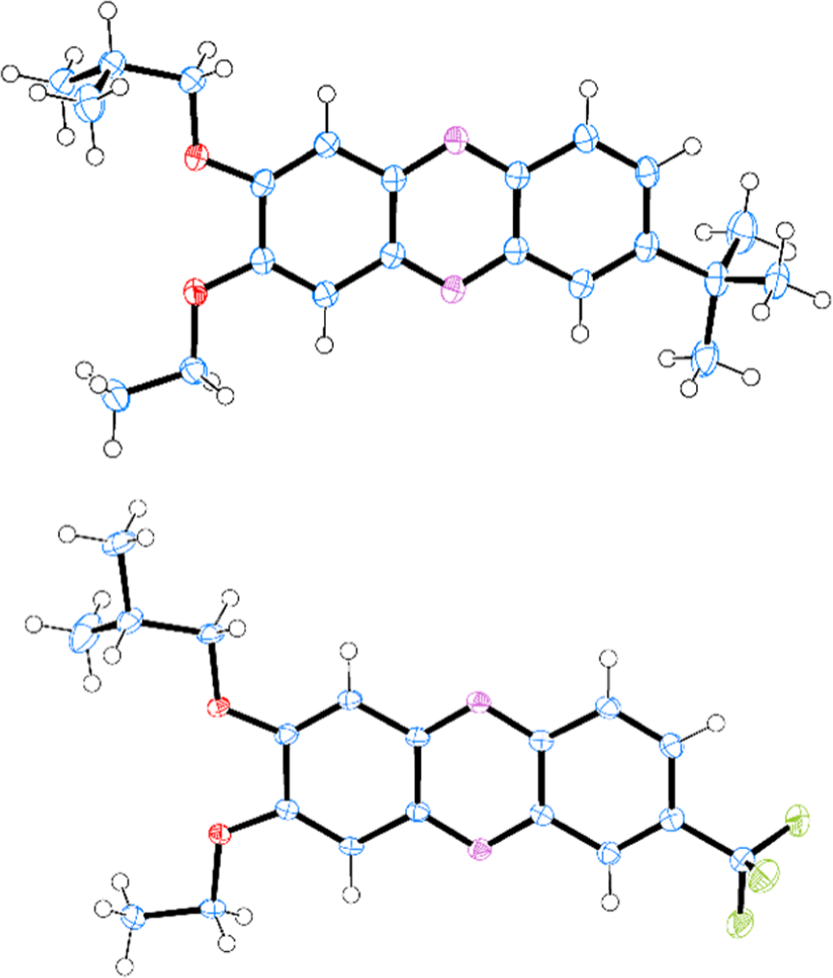
Conformation of the molecule **5c** (top) from the structure of its hydrate and **6b** from its structure with 1,2-dibromoethane (bottom). C, N, O, and F atoms are marked in blue, magenta, red, and green colors, respectively. The atoms are represented by displacement ellipsoids at the 50% probability level. H-atoms are shown as spheres in an arbitrary scale.

To confirm the molecular structure of the final compounds, single-crystal X-ray diffraction measurements were performed for the representative compounds **5c** and **6b**. Both compounds were crystallized under ambient conditions from methanol solutions, slowly diluted with water by establishing a water–methanol vapor equilibrium conditions in a sealed vial. The **6b** was additionally recrystallized from 1,2-dibromoethane. Although some attempts were made to obtain the crystals of inversely substituted **5b** and **6c** (Figure 3), all cases resulted in the formation of amorphous precipitates. This suggests that the position of the isobutoxy substituent in relation to the *tert*-butyl or trifluoromethyl group in the molecular structure is a crucial factor in the formation of a crystalline phase. When these groups are on opposite sides of the molecular core (such as in **5c** and **6b**), the steric hindrance is minimized and the compounds form crystalline phases. The molecular structure of **5c** and **6b** as determined from single-crystal X-ray diffraction experiments for the crystals of **5c hydrate** and **6b solvate** with 1,2-dibromoethane is given in Figure 4. Selected bond lengths, valence angles, and torsion angles for the studied molecules in the structures of **5c hydrate** and pure **6b** at *T* = 100 K and in **6b solvate** at *T* = 120 K are compared in Table S2.

**Figure 5:**
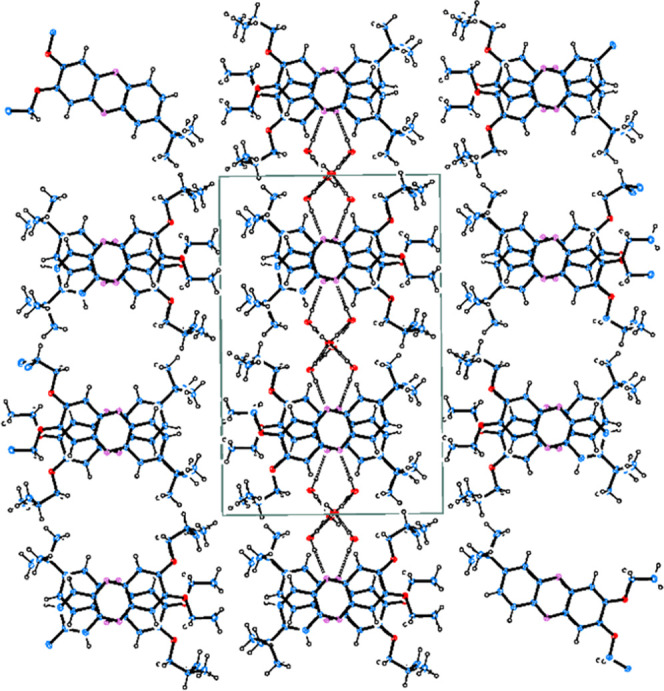
Packing of the phenazine and water molecules in the structure of **5c hydrate** viewed along [001]. The stacking of molecules is observed along *c* direction with the geometrical parameters for the relation of the pyrazine gravity centers Cg2^i^ at (*x*, −*y* + 1/2, *z* + 1/2)···Cg2 at (*x*, *y*, *z*)···Cg2^ii^ at (*x*, −*y* + 1/2, *z* – 1/2) being 3.453, 3.453 Å, and 178.9°, with Cg2^i^···π···Cg2^ii^ distances −3.348 and +3.348 Å, and off-sets 0.845 and 0.245 Å, respectively. O–H···N and O–H···O hydrogen bonds are marked by dashed lines.

The **5c** crystallizes as a hydrate in the monoclinic space group *P*2_1_/*c*, with the phenazine molecules arranged in columns along the *c* direction due to π–π interactions (Figure 5). Water molecules, joined together by a system of mutual O–H···O hydrogen bonds, fulfil the channels along the [001]. The phenazine columns are joined by the hydrogen bonds of N···H–O type to the water molecules to form layers extended parallel to *bc*. The three symmetrically independent positions of water molecules are not fully occupied, and the molecular ratio of phenazine to H_2_O was found to be different for different crystals. The appropriate drawings related to the crystal structure of **5c hydrate** are shown in Figures S153–S155. The geometrical parameters of hydrogen bonds and π–π interactions observed in the crystal structure are given in Table S3.

**Figure 6:**
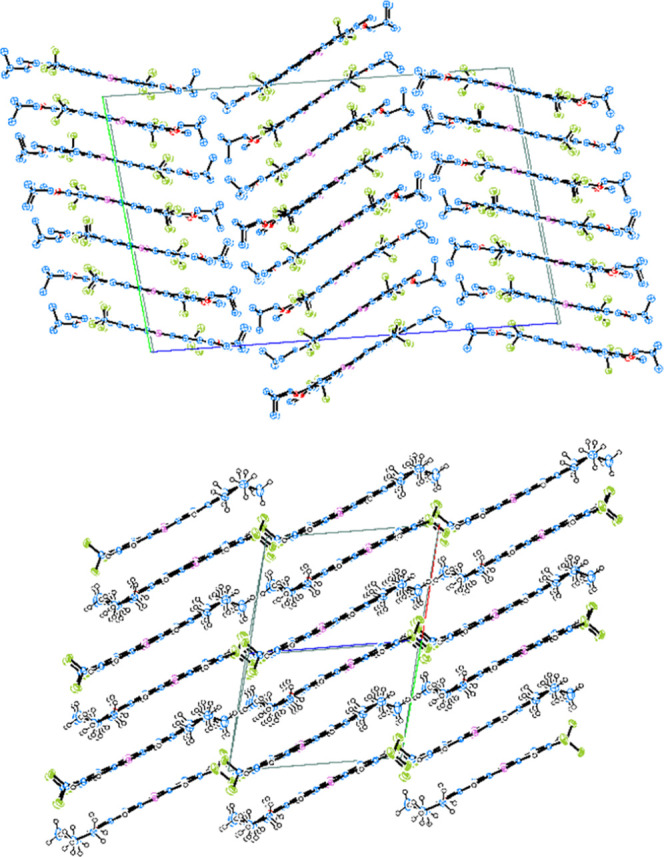
Packing of the molecules in the triclinic crystal structure of pure **6b** (top) viewed along [100] according to the refinement of the atom positions with isotropic displacement parameters and shown with H-atoms omitted for clarity; the mutual arrangement of the pyrazine molecules in the structure of **6b solvate** viewed along [110] (bottom) with the solvent molecules omitted for clarity. Unit cell directions *a*, *b****,*** and *c* are marked by red, green, and blue lines, respectively.

In the triclinic crystal structure of pure **6b** (space group *P*1̅), obtained under the same conditions as **5c**, no water molecules are present. The unit cell of the structure contains 24 molecules of **6b**, 12 of which (**A–L**) are symmetrically independent. Essential structural features of **6b** are presented in Figures S156 and S157. The asymmetric unit contents indicate that there are some “mistakes” in the mutual orientations of the molecules, which made the crystals of very poor quality. In the **6b** crystal structure, the molecules are joined *via* weak interactions both between the phenazine neighboring molecules to form columns along the *b* direction and by weak C–H···N, C–H···F and van der Waals interactions in between the columns. Figure 6 (top) shows the arrangement of the molecules viewed along [100], with the columns observed in *b* direction and the H atoms omitted for clarity. The summary of the hydrogen bond geometry observed in the crystal structure of pure **6b** is given in Table S3.

To get better geometrical parameters for the molecule of **6b**, an attempt of its recrystallization from 1,2-dibromoethane was performed and gave the triclinic crystals (space group *P*1̅) containing one molecule of **6b** (shown in Figure 4) and one solvent molecule in the asymmetric unit (Figure S158). Packing of the molecules in the structure of **6b** solvate, similar to that observed in the structure of pure **6b**, is shown in Figure 6 (bottom). In the structure of **6b solvate**, the dimers of phenazine molecules related to the center of symmetry at (1/2,1/2,1/2) are present and shown in Figure S159 with the pyrazine center of gravity Cg2 at (*x*, *y*, *z*) to Cg2 at (−*x* + 1, −*y* + 1, −*z* + 1) distance of 3.647 Å, the distance of Cg2 at (−*x* + 1, −*y* + 1, −*z* + 1) to the π system of the pyrazine ring equals 3.335 Å and off-set 1.476 Å. The geometrical details of the hydrogen bond interactions and the dimer in the **6b solvate** crystal structure are given in Table S3.

### Cytotoxicity toward the LoVo Cell Line

**Chart 1:**
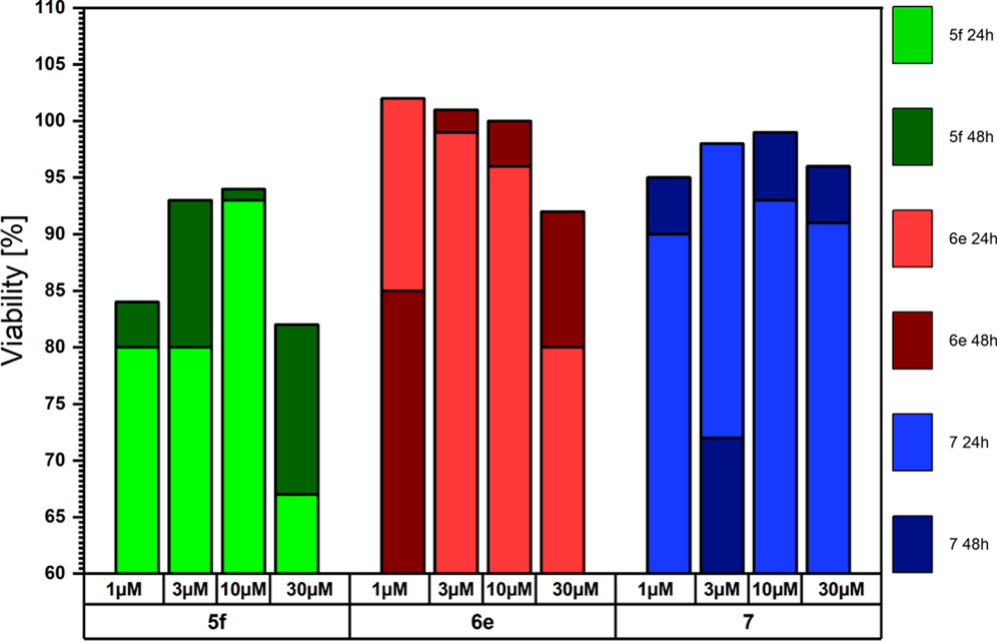
Viability of Cells in the Presence of Phenazines **5f**, **6e**, and **7** after Incubation for 24 and 48 h

**Chart 2:**
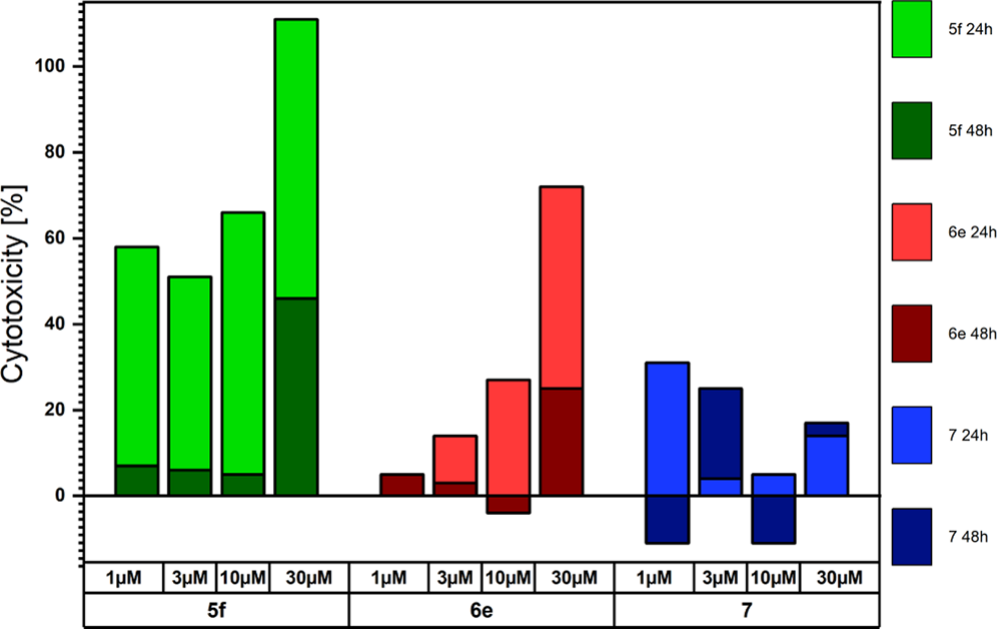
Cytotoxicity of Phenazines **5f**, **6e**, and **7** in Reference to a Control after Incubation for 24 and 48 h

Of the **5** and **6** series of phenazine compounds, **5f** and **6e** were chosen to perform cytotoxicity tests and the results were compared to those obtained for 2,3,7,8-tetraalkoxyphenazine **7**. The compounds were tested against the LoVo cell line, with the determination of both viability and cytotoxicity after 24 and 48 h of incubation with phenazines dissolved in dimethyl sulfoxide (DMSO) to final concentrations of 1, 3, 10, or 30 μM. Pure DMSO was used to determine the potential toxicity of the solvent. Doxorubicin (10 μM) and anthracycline with anticancer cytotoxic properties were used as a positive control. All results are presented compared to control cells, cultured without any exogeneous drug, a phenazine compound, or solvent additions, which were assumed to have 100% viability and 0% cytotoxicity. The viability in the presence of **5f**, **6e**, and **7** phenazines is presented in Chart 1 and the cytotoxicity in Chart 2. In both charts, the values are relative, in reference to the control, and the two colors for each compound correspond to two measurements made after 24 h (lighter color) and 48 h (darker color) of incubation. The experiments confirmed the cytotoxicity of compound **5f**. **6e** and **7** seem to exhibit much lower cytotoxicity, suggesting a strong dependence of the compound activity on the change of the substituents, which needs to be investigated in future experiments. For **5f**, **6e**, and **7** compounds, the activity after 48 h was lower than that after 24 h, which suggests that the phenazines are not stable in the cell culture environment or are metabolized to inactive products.

## Conclusions

This study presents a new synthetic protocol that allows, for the first time, to obtain nonsymmetrically substituted 2,3-dialkoxyphenazines (**5**, **6**), in good yields (50–88%). The change of steric and electronic properties of molecules in result of nonsymmetrical substitution has the crucial impact in the interaction of ligand with receptor^45^ in biological systems. The 14 new phenazines were synthesized from bis(2-nitrophenyl)amine derivatives **2**, **3**, and **4**, which were prepared *via* the Buchwald–Hartwig reaction of recently described, non-symmetrically substituted 4,5-dialkoxy-2-nitroanilines^40^ with 1-bromo-2-nitrobenzene derivatives. In the reaction, three substrates with different reactivities were successfully coupled with nitroanilines by adjusting only the reaction time and temperature. This confirms that the applied synthetic route can be successfully used in the synthesis of 2,3-dialkoxyphenazines from substrates with different reactivities, with electron-donor and electron-withdrawing groups. Using unsymmetrically substituted 4,5-dialkoxy-2-nitroanilines allows for the regioselective synthesis of 2,3-dialkoxyphenazines with two different alkoxy groups substituted in the designed positions. The molecular structure of the final compounds and the regioselectivity of the reactions were proven by NMR spectroscopy and, in the case of crystalline **5c hydrate**, pure **6b** and **6b solvate** were also confirmed by the single-crystal X-ray diffraction methods. For three examples (**5f**, **6e**, and **7**), viability and cytotoxicity experiments were performed. The results of tests performed on the LoVo cell line showed that compound **5f** exhibits promising cytotoxicity. The presence of two different alkoxy-groups in 2,3-dialkoxyphenaizne derivatives that can be obtained *via* the presented synthetic protocol may have a great impact on the optimization of their pharmacological properties.

## Experimental Section

All of the NMR spectra were collected on a Bruker AVANCE III 300 MHz spectrometer. Structural assignments were made with additional information from gCOSY, gHSQC, and gHMBC experiments. All attenuated total reflectance IR (ATR-IR) spectra were measured on the Thermo Scientific NICOLET iS5 spectrometer using the iD5 ATR interface. Melting points were measured on a polarized light microscope (Axioscope A1 Pol) using a thermostatic interface (LINKAM LTSE420). The high-resolution mass spectrometry (HRMS) data were determined on a Bruker Daltonics micrOTOF-Q II spectrometer.

### Crystallographic Data

X-ray diffraction experiments for single crystals of **5c hydrate**, pure **6b**, and **6b solvate** were performed using either a Rigaku XtaLAB Synergy-S or SuperNova diffractometers employing the CrysAlisPro softwares (Rigaku—Oxford Diffraction)^46^ for data collection, cell refinement, and data reduction. Crystal data, intensity measurement conditions, and structure refinement details for **5c hydrate** and **6b** at *T* = 100 K and for **6b solvate** at *T* = 120 K are given in Table S1. The phase problem was solved by direct methods using SIR92^47^ for **5c hydrate** and SHELXT’2014/5^48^ for pure **6b** and **6b solvate**. The structural parameters were refined by the method of full-matrix least squares on F^2^ using SHELXL’2013/4.^49^ Drawings of these structures were prepared using ORTEP-3^50^ All programs were operated under the WinG integrated system (version 2014.1).^50^ Crystallographic data were deposited with the Cambridge Crystallographic Data Centre under the numbers CCDC 2193582, CCDC 2193583, and CCDC 2193584 for **5c hydrate**, pure **6b**, and **6b solvate**, respectively. Copies of the data can be obtained, free of charge, on application to CCDC, 12 Union Road, Cambridge CB2 1EZ, UK, (fax: +44(0)1223 336033 or Email: deposit@ccdc.cam.ac.uk).

### Cell Viability and Cytotoxicity Examination

Cells from the LoVo colorectal cancer cell line (ATCC CCL-229, Manassas, USA) were grown to 70–80% confluence in the F-12K culture medium (Gibco, USA) supplemented with 10% foetal bovine serum (Sigma-Aldrich, USA) and a penicillin–streptomycin mixture (Lonza Biosciences, USA). To optimize the cell culture conditions, initial experiments were performed using LoVo cells in the abovementioned medium, cells in the same medium supplemented with DMSO, and cells in the same medium with DMSO, with each of the three phenazine compounds added to final concentrations of 100, 10, 1, and 0.01 μM, respectively. Phenazine compounds at initial concentrations of 1.934 mM (**7**), 2.086 mM (**6e**), and 2.043 mM (**5f**) dissolved in DMSO were added to the media to reach the final concentrations.

For this assay, 4 × 10^4^ cells were used in each well of the plate. To wells without any cells added, to some wells culture media were added only, and to others culture media and DMSO were added to the cells to act as negative controls. Moreover, antineoplastic anthracycline and doxorubicin (a final concentration of 10 μM) were added to some of the cells to act as a positive control for this assay. After optimization of the cell culture conditions, cells were grown as previously described in the presence of phenazine compounds at final concentrations in the media of 1, 3, 10, and 30 μM, respectively. Cells were collected after 24 or 48 h of incubation with the phenazines. The impact of the phenazine derivatives on cell viability and cytotoxicity was evaluated by the MultiTox-Fluor Multiplex Cytotoxicity assay (Promega, USA), according to the manufacturer’s instructions.

### Starting Materials

2-Bromo-4-(*tert*-butyl)-1-nitrobenzene used in **2** was synthesized according to known literature procedures. Unsymmetrical nitroanilines (**1**) were obtained as described.^40^ The solvent used in the synthesis of **2** and **3** was dried as described in the literature, distilled, and then stored over 4 Å molecular sieves. All other reagents and solvents were used as obtained without further purification.

### Synthesis of 4-Bromo-1,2-dimethoxybenzene

Veratrole (1.382 g, 10 mmol) was dissolved in DCM (20 mL), to which 1.758 g of bromine (11 mmol) in 10 mL of DCM was added dropwise. The reaction mixture was stirred under argon for 48 h and then washed with sodium thiosulfate solution and brine. The organic layer was dried over anhydrous magnesium sulfate and concentrated on a rotary evaporator. The crude product was then purified by column chromatography with DCM on silica gel to obtain the pure product in 88% yield (1.910 g). ^1^H NMR (CDCl_3_, 300 MHz, δ ppm): 7.04 (dd, ^3^*J*_H5–H6_ = 8.54 Hz, ^4^*J*_H5–H3_ = 2.32 Hz, 1H, H_5_), 6.99 (d, ^4^*J*_H5–H3_ = 2.32 Hz, 1H, H_3_), 6.74 (d, ^3^*J*_H5–H6_ = 8.64 Hz, 1H, H_6_), 3.87 (s, 3H, H_2a_), 3.86 (s, 3H, H_1a_).

### Synthesis of 1-Bromo-4,5-dimethoxy-2-nitrobenzene

Concentrated nitric acid (10 mL, 140 mmol) was cooled to −5 °C in an ice-water bath, and then 4-bromo-1,2-dimethoxybenzene (1 g, 4.607 mmol) was added in small portions to maintain the reaction temperature at around −5 °C. After 25 min of stirring, the reaction mixture was poured into water (50 mL). The precipitate was vacuum-filtered and washed with water (30 mL) before being dried under vacuum and used without further purification. The procedure resulted in a yellow crystalline solid with 42% (507 mg) yield of titled compound. ^1^H NMR (CDCl_3_, 300 MHz, δ ppm): 7.57 (s, 1H, H_3_), 7.12 (s, 1H, H_6_), 3.97 (s, 3H, H_5a_), 3.94 (s, 3H, H_4a_).

### Synthesis of **2** and **3***via* the Buchwald–Hartwig Coupling General Procedure

To a 10 mL threaded tube, **1** (1 equiv, 0.5 mmol), palladium(II) acetate (6 mg, 0.06 equiv, 0.03 mmol), RuPhos (12 mg, 0.06 equiv, 0.03 mmol), caesium carbonate (650 mg, 4 equiv, 2 mmol), and 2-bromo-4-(*tert*-butyl)-1-nitrobenzene (1 equiv, 0.5 mmol, in the synthesis of **2**) or 1-bromo-2-nitro-4-(trifluoromethyl)benzene (1 equiv, 0.5 mmol, in the synthesis of **3**) were added. The tube was then flushed several times with argon before adding toluene (2 mL) and flushing again. The tube was sealed, and the mixture was heated at 110 °C for 24 h (**2**) or 48 h (**3**) on an oil bath. After this time, the reaction mixture was cooled to room temperature, diluted with DCM (2 mL), filtered through a pad of silica gel, and washed out with DCM. The solution was then concentrated on a rotatory evaporator and purified by column chromatography on silica gel with a gradient elution of hexane:DCM (4:1 to 0:1).

### *N*-(5-(*tert*-Butyl)-2-nitrophenyl)-5-isobutoxy-4-methoxy-2-nitroaniline (**2a**)

The general procedure resulted in a red powder with 92% (192 mg) yield of the titled compound. mp = 135–145 °C. ^1^H NMR (CDCl_3_, 300 MHz, δ ppm): 11.18 (s, 1H, H_N–H_), 8.15 (d, ^3^*J*_H9–H10_ = 8.89 Hz, 1H, H_9_), 7.71 (s, 1H, H_3_), 7.59 (d, ^4^*J*_H12–H10_ = 2.00 Hz, 1H, H_12_), 7.10 (dd, ^3^*J*_H9–H10_ = 8.89 Hz, ^4^*J*_H10–H12_ = 2.00 Hz, 1H, H_10_), 6.97 (s, 1H, H_6_), 3.93 (s, 3H, H_4a_), 3.70 (d, ^3^*J*_H5a–H5b_ = 6.75 Hz, 2H, H_5a_), 2.25–2.11 (m, 1H, H_5b_), 1.32 (s, 9H, H_11b_), 1.01 (d, ^3^*J*_H5b–H5c_ = 6.75 Hz, 6H, H_5c_). ^13^C{^1^H} NMR (CDCl_3_, 75 MHz, δ ppm): 159.9 (C_8_), 155.8 (C_5_), 144.9 (C_4_), 137.8 (C_7_), 137.0 (C_11_), 134.5 (C_2_), 131.1 (C_1_), 127.3 (C_9_), 119.8 (C_10_), 117.4 (C_12_), 108.9 (C_3_), 102.4 (C_6_), 76.5 (C_5a_), 57.2 (C_4a_), 36.2 (C_11a_), 31.5 (C_11b_), 28.7 (C_5b_), 19.8 (C_5c_). FT-IR (ATR, *v*_max_, (neat)/cm^–1^): 3270, 2961, 2932, 2899, 2871, 1605, 1582, 1515, 1487, 1468, 1441, 1318, 1274, 1250, 1208, 1194, 1085, 1066, 1025, 999, 992, 850, 837. HRMS (ESI): *m*/*z* calcd for C_21_H_27_N_3_O_6_Na [M + Na]^+^, 440.1793; found, 440.1795.

### *N*-(5-(*tert*-Butyl)-2-nitrophenyl)-4-ethoxy-5-isobutoxy-2-nitroaniline (**2b**)

The general procedure resulted in a dark orange powder with 95% (205 mg) yield of the titled compound. mp = 115–118 °C. ^1^H NMR (CDCl_3_, 300 MHz, δ ppm): 11.17 (s, 1H, H_N–H_), 8.14 (d, ^3^*J*_H9–H10_ = 8.91 Hz, 1H, H_9_), 7.71 (s, 1H, H_3_), 7.59 (d, ^4^*J*_H10–H12_ = 1.90 Hz, 1H, H_12_), 7.09 (dd, ^3^*J*_H9–H10_ = 8.91 Hz, ^4^*J*_H10–H12_ = 1.90 Hz, 1H, H_10_), 6.96 (s, 1H, H_6_), 4.03 (q, ^3^*J*_H4a–H4b_ = 6.93 Hz, 2H, H_4a_), 3.69 (d, ^3^*J*_H5a–H5b_ = 6.71 Hz, 2H, H_5a_), 2.24–2.10 (m, 1H, H_5b_), 1.48 (t, ^3^*J*_H4a–H4b_ = 7.05 Hz 3H, H_4b_), 1.32 (s, 9H, H_11b_), 1.02 (d, ^3^*J*_H5b–H5c_ = 6.62 Hz, 6H, H_5c_). ^13^C{^1^H} NMR (CDCl_3_, 75 MHz, δ ppm): 159.6 (C_8_), 156.1 (C_5_), 144.2 (C_4_), 137.9 (C_7_), 136.8 (C_11_), 134.4 (C_2_), 131.4 (C_1_), 127.3 (C_9_), 119.8 (C_10_), 117.4 (C_12_), 110.5 (C_3_), 102.6 (C_6_), 76.4 (C_5a_), 65.9 (C_4a_), 36.1 (C_11a_), 31.5 (C_11b_), 28.7 (C_5b_), 19.7 (C_5c_), 15.2 (C_4b_). FT-IR (ATR, *v*_max_, (neat)/cm^–1^): 3314, 2974, 2930, 2871, 1610, 1579, 1514, 1485, 1467, 1435, 1417, 1397, 1350, 1319, 1252, 1208, 1197, 1082, 1064, 1045, 1014, 646, 872, 848, 818, 803, 758. HRMS (ESI): *m*/*z* calcd for C_22_H_29_N_3_O_6_Na [M + Na]^+^, 454.1949; found, 454.1949.

### *N*-(5-(*tert*-Butyl)-2-nitrophenyl)-5-ethoxy-4-isobutoxy-2-nitroaniline (**2c**)

The general procedure resulted in a dark orange powder with 96% (207 mg) yield of the titled compound. mp = 156–158 °C. ^1^H NMR (CDCl_3_, 300 MHz, δ ppm): 11.12 (s, 1H, H_N–H_), 8.14 (d, ^3^*J*_H9–H10_ = 8.92 Hz, 1H, H_9_), 7.69 (s, 1H, H_3_), 7.56 (d, ^4^*J*_H10–H12_ = 2.04 Hz, 1H, H_12_), 7.09 (dd, ^3^*J*_H9–H10_ = 8.92 Hz, ^4^*J*_H10–H12_ = 2.04 Hz, 1H, H_10_), 6.94 (s, 1H, H_6_), 4.02 (q, ^3^*J*_H5a–H5b_ = 6.98 Hz, 2H, H_5a_), 3.81 (d, ^3^*J*_H4a–H4b_ = 6.64 Hz, 2H, H_4a_), 2.25–2.11 (m, 1H, H_4b_), 1.46 (t, ^3^*J*_H5a–H5b_ = 7.00 Hz 3H, H_5b_), 1.31 (s, 9H, H_11b_), 1.07 (d, ^3^*J*_H4b–H4c_ = 6.71 Hz, 6H, H_4c_). ^13^C{^1^H} NMR (CDCl_3_, 75 MHz, δ ppm): 159.7 (C_8_), 155.9 (C_5_), 144.5 (C_4_), 138.0 (C_7_), 136.8 (C_11_), 134.3 (C_2_), 131.4 (C_1_), 127.2 (C_9_), 119.8 (C_10_), 117.2 (C_12_), 110.3 (C_3_), 102.8 (C_6_), 76.6 (C_4a_), 65.6 (C_5a_), 36.1 (C_11a_), 31.5 (C_11b_), 28.9 (C_4b_), 19.9 (C_4c_), 15.2 (C_5b_). FT-IR (ATR, *v*_max_, (neat)/cm^–1^): 3307, 3107, 2959, 2928, 2904, 2871, 1611, 1579, 1533, 1513, 1487, 1468, 1436, 1413, 1395, 1365, 1352, 1319, 1274, 1250, 1201, 1177, 1083, 1068, 1040, 1021, 960, 926, 887, 867, 849, 819, 806, 756, 700. HRMS (ESI): *m*/*z* calcd for C_22_H_29_N_3_O_6_Na [M + Na]^+^, 454.1949; found, 454.1951.

### 4-Butoxy-*N*-(5-(*tert*-butyl)-2-nitrophenyl)-5-isobutoxy-2-nitroaniline (**2d**)

The general procedure resulted in an orange powder with 95% (220 mg) yield of the titled compound. mp = 139–141 °C. ^1^H NMR (CDCl_3_, 300 MHz, δ ppm): 11.18 (s, 1H, H_N–H_), 8.15 (d, ^3^*J*_H9–H10_ = 8.89 Hz, 1H, H_9_), 7.71 (s, 1H, H_3_), 7.71 (d, ^4^*J*_H10–H12_ = 1.97 Hz, 1H, H_12_), 7.09 (dd, ^3^*J*_H9–H10_ = 8.89 Hz, ^4^*J*_H10–H12_ = 1.97 Hz, 1H, H_10_), 6.96 (s, 1H, H_6_), 4.06 (t, ^3^*J*_H4a–H4b_ = 6.42 Hz, 2H, H_4a_), 3.69 (d, ^3^*J*_H5a–H5b_ = 6.67 Hz, 2H, H_5a_), 2.24–2.09 (m, 1H, H_5b_), 1.90–1.79 (m, 2H, H_4b_), 1.62–1.48 (m, 2H, H_4c_), 1.32 (s, 9H, H_11b_), 1.02 (d, ^3^*J*_H5b–H5c_ = 6.69 Hz, 6H, H_5c_), 1.01 (t, ^3^*J*_H4c–H4d_ = 7.38 Hz, 3H, H_4d_). ^13^C{^1^H} NMR (CDCl_3_, 75 MHz, δ ppm): 159.6 (C_8_), 156.2 (C_5_), 144.5 (C_4_), 137.9 (C_7_), 136.9 (C_11_), 134.4 (C_2_), 131.2 (C_1_), 127.3 (C_9_), 119.7 (C_10_), 117.4 (C_12_), 110.3 (C_3_), 102.6 (C_6_), 76.3 (C_5a_), 70.0 (C_4a_), 36.2 (C_11a_), 31.7 (C_4b_), 31.5 (C_11b_), 28.8 (C_5b_), 19.8 (C_4c_), 19.7 (C_5c_), 14.5 (C_4d_). FT-IR (ATR, *v*_max_, (neat)/cm^–1^): 3320, 2959, 2930, 2870, 1609, 1575, 1513, 1486, 1469, 1454, 1432, 1406, 1347, 1319, 1271, 1248, 1199, 1177, 1084, 1066, 1014, 966, 953, 925, 867, 846, 826, 757, 702. HRMS (ESI): *m*/*z* calcd for C_24_H_33_N_3_O_6_Na [M + Na]^+^, 482.2262; found, 482.2265.

### *N*-(5-(*tert*-Butyl)-2-nitrophenyl)-4-(hexyloxy)-5-isobutoxy-2-nitroaniline (**2e**)

The general procedure resulted in an orange powder with 77% (190 mg) yield of the titled compound. mp = 110–117 °C. ^1^H NMR (CDCl_3_, 300 MHz, δ ppm): 11.18 (s, 1H, H_N–H_), 8.15 (d, ^3^*J*_H9–H10_ = 8.67 Hz, 1H, H_9_), 7.70 (s, 1H, H_3_), 7.59 (d, ^4^*J*_H10–H12_ = 1.90 Hz, 1H, H_12_), 7.09 (dd, ^3^*J*_H9–H10_ = 8.67 Hz, ^4^*J*_H10–H12_ = 1.90 Hz, 1H, H_10_), 6.96 (s, 1H, H_6_), 4.05 (t, ^3^*J*_H4a–H4b_ = 6.50 Hz, 2H, H_4a_), 3.69 (d, ^3^*J*_H5a–H5b_ = 6.50 Hz, 2H, H_5a_), 2.23–2.10 (m, 1H, H_5b_), 1.91–1.80 (m, 2H, H_4b_), 1.58–1.45 (m, 2H, H_4c_), 1.44–1.33 (m, 4H, H_4d,4e_), 1.35 (s, 9H, H_11b_), 1.02 (d, ^3^*J*_H5b–H5c_ = 6.77 Hz, 6H, H_5c_), 0.93 (t, ^3^*J*_H4e–H4f_ = 7.31 Hz, 3H, H_4f_). ^13^C{^1^H} NMR (CDCl_3_, 75 MHz, δ ppm): 159.6 (C_8_), 156.2 (C_5_), 144.5 (C_4_), 137.9 (C_7_), 136.9 (C_11_), 134.4 (C_2_), 131.2 (C_1_), 127.2 (C_9_), 119.7 (C_10_), 117.4 (C_12_), 110.3 (C_3_), 102.6 (C_6_), 76.3 (C_5a_), 70.3 (C_4a_), 36.2 (C_11a_), 32.1 (C_4d_), 31.5 (C_11b_), 29.6 (C_4b_), 28.8 (C_5b_), 26.3 (C_4c_), 23.2 (C_4e_), 19.7 (C_5c_), 14.6 (C_4f_). FT-IR (ATR, *v*_max_, (neat)/cm^–1^): 3276, 3106, 1956, 2925, 2871, 2855, 1744, 1623, 1610, 1581, 1515, 1488, 1470, 1438, 1421, 1396, 1351, 1337, 1323, 1251, 1197, 1083, 1072, 1043, 1021, 996, 956, 852, 825, 759, 702. HRMS (ESI): *m*/*z* calcd for C_26_H_37_N_3_O_6_Na [M + Na]^+^, 510.2575; found, 510.2575.

### *N*-(5-(*tert*-Butyl)-2-nitrophenyl)-5-isobutoxy-2-nitro-4-(octyloxy)aniline (**2f**)

The general procedure resulted in a light orange powder with 96% (249 mg) yield of titled compound. mp = 100–102 °C. ^1^H NMR (CDCl_3_, 300 MHz, δ ppm): 11.18 (s, 1H, H_N–H_), 8.15 (d, ^3^*J*_H9–H10_ = 8.92 Hz, 1H, H_9_), 7.70 (s, 1H, H_3_), 7.59 (d, ^4^*J*_H10–H12_ = 2.09 Hz, 1H, H_12_), 7.09 (dd, ^3^*J*_H9–H10_ = 8.92 Hz, ^4^*J*_H10–H12_ = 2.09 Hz, 1H, H_10_), 6.96 (s, 1H, H_6_), 4.04 (t, ^3^*J*_H4a–H4b_ = 6.46 Hz, 2H, H_4a_), 3.69 (d, ^3^*J*_H5a–H5b_ = 6.69 Hz, 2H, H_5a_), 2.24–2.09 (m, 1H, H_5b_), 1.91–1.79 (m, 2H, H_4b_), 1.55–1.45 (m, 2H, H_4c_), 1.44–1.24 (m, 8H, H_4d,4e,4f,4g_), 1.32 (s, 9H, H_11b_), 1.02 (d, ^3^*J*_H5b–H5c_ = 6.75 Hz, 6H, H_5c_), 0.90 (t, ^3^*J*_H4g–H4h_ = 7.02 Hz, 3H, H_4h_). ^13^C{^1^H} NMR (CDCl_3_, 75 MHz, δ ppm): 159.6 (C_8_), 156.1 (C_5_), 144.5 (C_4_), 137.9 (C_7_), 136.7 (C_11_), 134.3 (C_2_), 131.2 (C_1_), 127.3 (C_9_), 119.7 (C_10_), 117.4 (C_12_), 110.3 (C_3_), 102.6 (C_6_), 76.3 (C_5a_), 70.3 (C_4a_), 36.2 (C_11a_), 32.4 (C_4f_), 31.5 (C_11b_), 29.9 (C_4b_), 29.9 (C_4d_), 29.7 (C_4e_), 28.8 (C_5b_), 26.6 (C_4c_), 23.3 (C_4g_), 19.7 (C_5c_), 14.2 (C_4h_). FT-IR (ATR, *v*_max_, (neat)/cm^–1^): 3300, 2958, 2928, 2873, 2856, 1610, 1581, 1535, 1515, 1486, 1468, 1437, 1395, 1352, 1320, 1252, 1198, 1084, 1068, 1044, 1014, 996, 970, 955, 861, 850, 824, 809, 759, 700. HRMS (ESI): *m*/*z* calcd for C_28_H_41_N_3_O_6_Na [M + Na]^+^, 538.2888; found, 538.2888.

### *N*-(5-(*tert*-Butyl)-2-nitrophenyl)-4-(decyloxy)-5-isobutoxy-2-nitroaniline (**2g**)

The general procedure resulted in a light orange powder with 78% (212 mg) yield of titled compound. mp = 89–100 °C. ^1^H NMR (CDCl_3_, 300 MHz, δ ppm): 11.18 (s, 1H, H_N–H_), 8.15 (d, ^3^*J*_H9–H10_ = 8.86 Hz, 1H, H_9_), 7.70 (s, 1H, H_3_), 7.59 (d, ^4^*J*_H10–H12_ = 1.97 Hz, 1H, H_12_), 7.09 (dd, ^3^*J*_H9–H10_ = 8.86 Hz, ^4^*J*_H10–H12_ = 1.97 Hz, 1H, H_10_), 6.96 (s, 1H, H_6_), 4.04 (t, ^3^*J*_H4a–H4b_ = 6.40 Hz, 2H, H_4a_), 3.69 (d, ^3^*J*_H5a–H5b_ = 6.65 Hz, 2H, H_5a_), 2.24–2.09 (m, 1H, H_5b_), 1.91–1.79 (m, 2H, H_4b_), 1.57–1.45 (m, 2H, H_4c_), 1.44–1.24 (m, 12H, H_4d,4e,4f,4g,4h,4i_), 1.32 (s, 9H, H_11b_), 1.02 (d, ^3^*J*_H5b–H5c_ = 6.77 Hz, 6H, H_5c_), 0.90 (t, ^3^*J*_H4i–H4j_ = 6.67 Hz, 3H, H_4j_). ^13^C{^1^H} NMR (CDCl_3_, 75 MHz, δ ppm): 159.6 (C_8_), 156.2 (C_5_), 144.5 (C_4_), 137.9 (C_7_), 136.9 (C_11_), 134.4 (C_2_), 131.3 (C_1_), 127.3 (C_9_), 119.7 (C_10_), 117.4 (C_12_), 110.3 (C_3_), 102.6 (C_6_), 76.3 (C_5a_), 70.3 (C_4a_), 36.2 (C_11a_), 32.4 (C_4h_), 31.5 (C_11b_), 30.2–29.8 (C_4b,4d,4e,4f_), 29.7 (C_4g_), 28.8 (C_5b_), 26.6 (C_4c_), 23.3 (C_4i_), 19.7 (C_5c_), 14.7 (C_4j_). FT-IR (ATR, *v*_max_, (neat)/cm^–1^): 3307, 2958, 2926, 2869, 2855, 1610, 1580, 1515, 1486, 1468, 1435, 1393, 1347, 1319, 1252, 1199, 1082, 1064, 1040, 1001, 953, 860, 820, 807, 757, 737, 698. HRMS (ESI): *m*/*z* calcd for C_22_H_29_N_3_O_6_Na [M + Na]^+^, 566.3201; found, 566.3201.

### 5-Isobutoxy-4-methoxy-2-nitro-*N*-(2-nitro-4-(trifluoromethyl)phenyl)aniline (**3a**)

The general procedure resulted in an orange powder with 62% (135 mg) yield of titled compound. mp = 136–139 °C. ^1^H NMR (CDCl_3_, 300 MHz, δ ppm): 11.06 (s, 1H, H_N–H_), 8.51 (d, ^4^*J*_H9–H11_ = 2.19 Hz, 1H, H_9_), 7.70 (s, 1H, H_3_), 7.68 (dd, ^3^*J*_H11–H12_ = 8.93 Hz, ^4^*J*_H9–H11_ = 2.19 Hz, 1H, H_11_), 7.53 (d, ^3^*J*_H11–H12_ = 8.93 Hz, 1H, H_12_), 6.94 (s, 1H, H_6_), 3.96 (s, 3H, H_4a_), 3.76 (d, ^3^*J*_H5b–H5c_ = 6.68 Hz, 2H, H_5a_), 2.19 (m, 1H, H_5b_), 1.05 (d, ^3^*J*_H5b–H5c_ = 6.79 Hz, 6H, H_5c_). ^13^C{^1^H} NMR (CDCl_3_, 75 MHz, δ ppm): 155.3 (C_5_), 146.8 (C_4_), 142.3 (C_7_), 136.4 (C_8_), 133.8 (C_2_), 132.0 (q, ^3^*J*_C–F_ = 3 Hz, C_11_), 130.8 (C_1_), 125.5 (q, ^3^*J*_C–F_ = 4 Hz C_9_), 122.9–121.9 (m, C_10_), 118.8 (C_12_), 109.1 (C_3_), 105.7 (C_6_), 76.6 (C_5a_), 57.2 (C_4a_), 28.8 (C_5b_), 19.7 (C_5c_), signal from C_10a_ is missing. ^19^F NMR (CDCl_3_, 282 MHz, δ ppm): −63.43 (s, 3F, F_CF3_). FT-IR (ATR, *v*_max_, (neat)/cm^–1^): 3303, 3113, 2976, 2960, 2932, 2919, 2878, 2851, 2834, 1633, 1613, 1584, 1514, 1465, 1442, 1412, 1359, 1323, 1301, 1258, 1180, 1153, 1127, 1109, 1085, 1066, 1034, 1005, 975, 914, 892, 866, 853, 826, 799, 783, 758, 683. HRMS (ESI): *m*/*z* calcd for C_18_H_18_N_3_O_6_F_3_Na [M + Na]^+^, 452.1040; found, 452.1041.

### 4-Ethoxy-5-isobutoxy-2-nitro-*N*-(2-nitro-4-(trifluoromethyl)phenyl)aniline (**3b**)

The general procedure resulted in an orange powder with 79% (176 mg) yield of titled compound. mp = 100–104 °C. ^1^H NMR (CDCl_3_, 300 MHz, δ ppm): 11.06 (s, 1H, H_N–H_), 8.48 (d, ^4^*J*_H9–H11_ = 2.20 Hz, 1H, H_9_), 7.68 (s, 1H, H_3_), 7.67 (dd, ^3^*J*_H11–H12_ = 8.75 Hz, ^4^*J*_H9–H11_ = 2.20 Hz, 1H, H_11_), 7.54 (d, ^3^*J*_H11–H12_ = 8.75 Hz, 1H, H_12_), 6.95 (s, 1H, H_6_), 4.16 (q, ^3^*J*_H4a–H4b_ = 6.96 Hz, 2H, H_4a_), 3.77 (d, ^3^*J*_H5a–H5b_ = 6.57 Hz, 2H, H_5a_), 2.18 (m, 1H, H_5b_), 1.50 (t, ^3^*J*_H4a–H4b_ = 6.96 Hz, 3H, H_4b_), 1.05 (d, ^3^*J*_H5b–H5c_ = 6.71 Hz, 6H, H_5c_). ^13^C{^1^H} NMR (CDCl_3_, 75 MHz, δ ppm): 155.7 (C_5_), 146.2 (C_4_), 142.3 (C_7_), 136.2 (C_8_), 133.8 (C_2_), 132.0 (q, ^3^*J*_C–F_ = 3 Hz, C_11_), 130.7 (C_1_), 125.4 (q, ^3^*J*_C–F_ = 4 Hz C_9_), 122.9–121.9 (m, C_10_), 118.8 (C_12_), 110.5 (C_3_), 106.0 (C_6_), 76.5 (C_5a_), 66.0 (C_4a_), 28.8 (C_5b_), 19.2 (C_5c_), 15.2 (C_4b_), signal from C_10a_ is missing. ^19^F NMR (CDCl_3_, 282 MHz, δ ppm): −63.39 (s, 3F, F_CF3_). FT-IR (ATR, *v*_max_, (neat)/cm^–1^): 3318, 3104, 2978, 2961, 2921, 2871, 2851, 1634, 1583, 1572, 1543, 1517, 1504, 1470, 1435, 1397, 1358, 1324, 1275, 1256, 1236, 1217, 1197, 1175, 1147, 1104, 1082, 1064, 1040, 1005, 920, 900, 879, 840, 824, 809, 782, 763, 748, 683. HRMS (ESI): *m*/*z* calcd for C_19_H_20_N_3_O_6_F_3_Na [M + Na]^+^, 466.1197; found, 466.1197.

### 5-Ethoxy-4-isobutoxy-2-nitro-*N*-(2-nitro-4-(trifluoromethyl)phenyl)aniline (**3c**)

The general procedure resulted in an orange powder with 71% (158 mg) yield of titled compound. mp = 138–142.5 °C. ^1^H NMR (CDCl_3_, 300 MHz, δ ppm): 11.08 (s, 1H, H_N–H_), 8.52 (d, ^4^*J*_H9–H11_ = 2.15 Hz, 1H, H_9_), 7.70 (s, 1H, H_3_), 7.66 (dd, ^3^*J*_H11–H12_ = 8.96 Hz, ^4^*J*_H9–H11_ = 2.15 Hz, 1H, H_11_), 7.53 (d, ^3^*J*_H11–H12_ = 8.96 Hz, 1H, H_12_), 6.94 (s, 1H, H_6_), 4.09 (q, ^3^*J*_H5a–H5b_ = 6.99 Hz, 2H, H_5a_), 3.84 (d, ^3^*J*_H4b–H4c_ = 6.78 Hz, 2H, H_4c_), 2.20 (m, 1H, H_4b_), 1.49 (t, ^3^*J*_H5a–H5b_ = 6.96 Hz, 3H, H_5b_), 1.08 (d, ^3^*J*_H4b–H4c_ = 6.74 Hz, 6H, H_4c_). ^13^C{^1^H} NMR (CDCl_3_, 75 MHz, δ ppm): 155.4 (C_5_), 146.4 (C_4_), 142.3 (C_7_), 136.3 (C_8_), 133.8 (C_2_), 131.9 (q, ^3^*J*_C–F_ = 3 Hz, C_11_), 130.7 (C_1_), 125.5 (q, ^3^*J*_C–F_ = 4 Hz C_9_), 123.0–121.9 (m, C_10_), 118.9 (C_12_), 110.3 (C_3_), 105.9 (C_6_), 76.7 (C_4a_), 66.0 (C_5a_), 28.8 (C_4b_), 19.8 (C_4c_), 15.1 (C_5b_), signal from C_10a_ is missing. ^19^F NMR (CDCl_3_, 282 MHz, δ ppm): −63.36 (s, 3F, F_CF3_). FT-IR (ATR, *v*_max_, (neat)/cm^–1^): 3312, 3104, 2978, 2967, 2947, 2926, 2880, 1637, 1614, 1583, 1538, 1520, 1495, 1471, 1444, 1424, 1396, 1365, 1322, 1277, 1261, 1233, 1213, 1192, 1156, 1111, 1082, 1071, 1036, 1020, 974, 913, 885, 853, 826, 809, 783, 761, 748, 684. HRMS (ESI): *m*/*z* calcd for C_19_H_20_N_3_O_6_F_3_Na [M + Na]^+^, 466.1197; found, 466.1198.

### 4-Butoxy-5-isobutoxy-2-nitro-*N*-(2-nitro-4-(trifluoromethyl)phenyl)aniline (**3d**)

The general procedure resulted in an orange powder with 50% (119 mg) yield of titled compound. mp = 125–126.5 °C. ^1^H NMR (CDCl_3_, 300 MHz, δ ppm): 11.06 (s, 1H, H_N–H_), 8.50 (d, ^4^*J*_H9–H11_ = 2.17 Hz, 1H, H_9_), 7.69 (s, 1H, H_3_), 7.67 (dd, ^3^*J*_H11–H12_ = 8.95 Hz, ^4^*J*_H9–H11_ = 2.17 Hz, 1H, H_11_), 7.53 (d, ^3^*J*_H11–H12_ = 8.95 Hz, 1H, H_12_), 6.93 (s, 1H, H_6_), 4.08 (t, ^3^*J*_H4a–H4b_ = 6.40 Hz, 2H, H_4a_), 3.76 (d, ^3^*J*_H5a–H5b_ = 6.55 Hz, 2H, H_5a_), 2.17 (m, 1H, H_5b_), 1.91–1.80 (m, 2H, H_4b_), 1.62–1.48 (m, 2H, H_4c_), 1.06 (d, ^3^*J*_H5b–H5c_ = 6.80 Hz, 6H, H_5c_), 1.02 (t, ^3^*J*_H4d–H4c_ = 7.40 Hz, 3H, H_4d_). ^13^C{^1^H} NMR (CDCl_3_, 75 MHz, δ ppm): 155.7 (C_5_), 146.4 (C_4_), 142.4 (C_7_), 136.2 (C_8_), 133.8 (C_2_), 131.9 (q, ^3^*J*_C–F_ = 3 Hz, C_11_), 130.6 (C_1_), 125.5 (q, ^3^*J*_C–F_ = 4 Hz C_9_), 122.9–121.9 (m, C_10_), 118.8 (C_12_), 110.3 (C_3_), 105.9 (C_6_), 76.4 (C_5a_), 70.1 (C_4a_), 31.6 (C_4b_), 28.9 (C_5b_), 19.8 (C_4c_), 19.7 (C_5c_), 14.4 (C_4d_), signal from C_10a_ is missing. ^19^F NMR (CDCl_3_, 282 MHz, δ ppm): −63.42 (s, 3F, F_CF3_). FT-IR (ATR, *v*_max_, (neat)/cm^–1^): 3299, 3102, 2963, 2932, 2877, 1733, 1633, 1611, 1584, 1539, 1518, 1491, 1472, 1463, 1445, 1424, 1397, 1364, 1327, 1306, 1282, 1258, 1212, 1175, 1159, 1133, 1084, 1036, 1005, 968, 911, 901, 848, 839, 807, 757, 687. HRMS (ESI): *m*/*z* calcd for C_21_H_25_N_3_O_6_F_3_ [M + H]^+^, 472.1690; found, 472.1688.

### 4-(Hexyloxy)-5-isobutoxy-2-nitro-*N*-(2-nitro-4-(trifluoromethyl)phenyl)aniline (**3e**)

The general procedure resulted in an orange powder with 75% (189 mg) yield of titled compound. mp = 61–66 °C. ^1^H NMR (CDCl_3_, 300 MHz, δ ppm): 11.07 (s, 1H, H_N–H_), 8.51 (d, ^4^*J*_H9–H11_ = 2.20 Hz, 1H, H_9_), 7.69 (s, 1H, H_3_), 7.66 (dd, ^3^*J*_H11–H12_ = 8.90 Hz, ^4^*J*_H9–H11_ = 2.20 Hz, 1H, H_11_), 7.51 (d, ^3^*J*_H11–H12_ = 8.90 Hz, 1H, H_12_), 6.91 (s, 1H, H_6_), 4.06 (t, ^3^*J*_H4a–H4b_ = 6.48 Hz, 2H, H_4a_), 3.74 (d, ^3^*J*_H5a–H5b_ = 6.41 Hz, 2H, H_5a_), 2.17 (m, 1H, H_5b_), 1.91–1.80 (m, 2H, H_4b_), 1.57–1.46 (m, 2H, H_4c_), 1.41–1.32 (m, 4H, H_4d,4e_), 1.05 (d, ^3^*J*_H5b–H5c_ = 6.76 Hz, 6H, H_5c_), 0.92 (t, ^3^*J*_H4e–H4f_ = 6.88 Hz, 3H, H_4f_). ^13^C{^1^H} NMR (CDCl_3_, 75 MHz, δ ppm): 155.7 (C_5_), 146.4 (C_4_), 142.4 (C_7_), 136.3 (C_8_), 133.9 (C_2_), 131.9 (q, ^3^*J*_C–F_ = 3 Hz, C_11_), 130.6 (C_1_), 125.5 (q, ^3^*J*_C–F_ = 2 Hz C_9_), 123.3–122.4 (m, C_10_), 118.8 (C_12_), 110.3 (C_3_), 105.9 (C_6_), 76.4 (C_5a_), 70.3 (C_4a_), 32.1 (C_4d_), 29.6 (C_4b_), 28.9 (C_5b_), 26.2 (C_4c_), 23.2 (C_4e_), 19.7 (C_5c_), 14.6 (C_4f_), signal from C_10a_ is missing. ^19^F NMR (CDCl_3_, 282 MHz, δ ppm): −63.43 (s, 3F, F_CF3_). FT-IR (ATR, *v*_max_, (neat)/cm^–1^): 3323, 3093, 2978, 2956, 2934, 2871, 2856, 1634, 1607, 1581, 1571, 1542, 1519, 1500, 1467, 1435, 1397, 1360, 1341, 1323, 1282, 1261, 1234, 1215, 1195, 1169, 1147, 1108, 1082, 1068, 1038, 1008, 986, 938, 917, 892, 874, 837, 825, 802, 781, 763, 741, 683. HRMS (ESI): *m*/*z* calcd for C_23_H_29_N_3_O_6_F_3_ [M + H]^+^, 500.2003; found, 500.2005.

### 5-Isobutoxy-2-nitro-*N*-(2-nitro-4-(trifluoromethyl)phenyl)-4-(octyloxy)aniline (**3f**)

The general procedure resulted in a dark yellow powder with 63% (167 mg) yield of titled compound. mp = 72–76 °C. ^1^H NMR (CDCl_3_, 300 MHz, δ ppm): 11.06 (s, 1H, H_N–H_), 8.50 (d, ^4^*J*_H9–H11_ = 2.18 Hz, 1H, H_9_), 7.68 (s, 1H, H_3_), 7.67 (dd, ^3^*J*_H11–H12_ = 8.90 Hz, ^4^*J*_H9–H11_ = 2.18 Hz, 1H, H_11_), 7.53 (d, ^3^*J*_H11–H12_ = 8.90 Hz, 1H, H_12_), 6.94 (s, 1H, H_6_), 4.07 (t, ^3^*J*_4a–4b_ = 6.53 Hz, 2H, H_4a_), 3.76 (d, ^3^*J*_H5a–H5b_ = 6.53 Hz, 2H, H_5a_), 2.18 (m, 1H, H_5b_), 1.92–1.81 (m, 2H, H_4b_), 1.57–1.45 (m, 2H, C_4c_), 1.44–1.24 (m, 8H, H_4d,4e,4f,4g_), 1.06 (d, ^3^*J*_H5b–H5c_ = 6.91 Hz, 6H, H_5c_), 0.90 (t, ^3^*J*_H4g–H4h_ = 6.91 Hz, 3H, H_4h_). ^13^C{^1^H} NMR (CDCl_3_, 75 MHz, δ ppm): 155.7 (C_5_), 146.4 (C_4_), 142.4 (C_7_), 136.2 (C_8_), 133.8 (C_2_), 132.0 (q, ^3^*J*_C–F_ = 3 Hz, C_11_), 130.6 (C_1_), 125.5 (q, ^3^*J*_C–F_ = 4 Hz C_9_), 122.9–121.9 (m, C_10_), 118.8 (C_12_), 110.3 (C_3_), 105.9 (C_6_), 76.4 (C_5a_), 70.3 (C_4a_), 32.4 (C_4f_), 29.9–29.8 (m, 2C, C_4b,4d_), 29.6 (C_4e_), 28.9 (C_5b_), 26.6 (C_4c_), 23.3 (C_4g_), 19.7 (C_5c_), 14.7 (C_4h_), signal from C_10a_ is missing. ^19^F NMR (CDCl_3_, 282 MHz, δ ppm): −63.42 (s, 3F, F_CF3_). FT-IR (ATR, *v*_max_, (neat)/cm^–1^): 3320, 3102, 2954, 2926, 2869, 2856, 1634, 1585, 1572, 1543, 1520, 1471, 1436, 1398, 1358, 1339, 1324, 1282, 1235, 1217, 1199, 1173, 1147, 1105, 1086, 1066, 1010, 988, 968, 917, 869, 874, 835, 822, 816, 783, 763, 746, 683. HRMS (ESI): *m*/*z* calcd for C_25_H_32_N_3_O_6_F_3_Na [M + Na]^+^, 550.2136; found, 550.2132.

### 4-(Decyloxy)-5-isobutoxy-2-nitro-*N*-(2-nitro-4-(trifluoromethyl)phenyl)aniline (**3g**)

The general procedure resulted in a dark yellow powder with 34% (96 mg) yield of titled compound. mp = 87–93 °C. ^1^H NMR (CDCl_3_, 300 MHz, δ ppm): 11.06 (s, 1H, H_N–H_), 8.51 (d, ^4^*J*_H9–H11_ = 2.05 Hz, 1H, H_9_), 7.69 (s, 1H, H_3_), 7.67 (dd, ^3^*J*_H11–H12_ = 8.97 Hz, ^4^*J*_H9–H11_ = 2.05 Hz, 1H, H_11_), 7.52 (d, ^3^*J*_H11–H12_ = 8.97 Hz, 1H, H_12_), 6.92 (s, 1H, H_6_), 4.07 (t, ^3^*J*_H4a–H4b_ = 6.42 Hz, 2H, H_4a_), 3.75 (d, ^3^*J*_H5a–H5b_ = 6.52 Hz, 2H, H_5a_), 2.18 (m, 1H, H_5b_), 1.92–1.81 (m, 2H, H_4b_), 1.57–1.45 (m, 2H, C_4c_), 1.43–1.23 (m, 12H, H_4d,4e,4f,4g,4h,4i_), 1.06 (d, ^3^*J*_H5b–H5c_ = 6.66 Hz, 6H, H_5c_), 0.89 (t, ^3^*J*_4Hi–4Hj_ = 6.90 Hz, 3H, H_4j_). ^13^C{^1^H} NMR (CDCl_3_, 75 MHz, δ ppm): 155.6 (C_5_), 146.5 (C_4_), 142.4 (C_7_), 136.3 (C_8_), 133.9 (C_2_), 132.0 (q, ^3^*J*_C–F_ = 3 Hz, C_11_), 130.6 (C_1_), 125.5 (q, ^3^*J*_C–F_ = 3 Hz C_9_), 122.8–121.9 (m, C_10_), 118.8 (C_12_), 110.3 (C_3_), 106.0 (C_6_), 76.4 (C_5a_), 70.3 (C_4a_), 32.5 (C_4h_), 30.2–29.8 (m, 4C, C_4b,4d,4e,4f_), 29.6 (C_4g_), 28.9 (C_5b_), 26.6 (C_4c_), 23.3 (C_4i_), 19.7 (C_5c_), 14.7 (C_4j_), signal from C_10a_ is missing. ^19^F NMR (CDCl_3_, 282 MHz, δ ppm): −63.43 (s, 3F, F_CF3_). FT-IR (ATR, *v*_max_, (neat)/cm^–1^): 3317, 3096, 2952, 2922, 2873, 2855, 1634, 1609, 1584, 1570, 1542, 1520, 1472, 1435, 1404, 1356, 1324, 1282, 1235, 1216, 1195, 1175, 1147, 1109, 1082, 1066, 1042, 1007, 975, 917, 894, 876, 837, 823, 816, 783, 759. HRMS (ESI): *m*/*z* calcd for C_27_H_36_N_3_O_6_F_3_Na [M + Na]^+^, 578.2449; found, 578.2449.

### Synthesis of *N*-(4,5-Bis(hexyloxy)-2-nitrophenyl)-4,5-dimethoxy-2-nitroaniline (**4**) *via* Buchwald–Hartwig Coupling

To a 10 mL threaded tube, 4,5-bis(hexyloxy)-2-nitroaniline (338 mg, 1 mmol), palladium(II) acetate (12 mg, 0.06 mmol), RuPhos (24 mg, 0.06 mmol), caesium carbonate (1300 mg, 4 mmol), and 1-bromo-4,5-dimethoxy-2-nitrobenzene (262 mg, 1 mmol) were added. The tube was flushed several times with argon before adding toluene (4 mL) and flushing again. The tube was then sealed, and the mixture was heated at 90 °C for 1.5 h on an oil bath. After this time, the reaction mixture was cooled to room temperature, diluted with DCM (4 mL), filtered through a pad of silica gel, and washed out with DCM. The solution was then concentrated on a rotatory evaporator and purified by column chromatography on silica gel with a gradient elution of DCM/methanol (1:0 to 95:5) to obtain the product as red powder in 93% (483 mg) yield. mp = 98–101 °C. ^1^H NMR (CDCl_3_, 300 MHz, δ ppm): 11.13 (s, 1H, H_N–H_), 7.68 and 7.67 (s, 2× 1H, H_3,13_), 6.93 and 6.92 (s, 2× 1H, H_6,16_), 4.03 (t, ^3^*J*_H4a–H4b_ = 6.60 Hz, 2H, H_4a_), 3.94 (t, ^3^*J*_H5a–H5b_ = 6.60 Hz, 2H, H_5a_), 3.94 (s, 3H, H_14a_), 3.85 (s, 3H, H_15a_), 1.90–1.77 (m, 4H, H_4b,5b_), 1.55–1.41 (m, 4H, H_4c,5c_), 1.41–1.28 (m, 8H, H_4d,4e,5d,5e_), 0.95–0.85 (m, 6H, H_4f,5f_). ^13^C{^1^H} NMR (CDCl_3_, 75 MHz, δ ppm): 155.9 and 155.7 (C_14,15_), 144.6 and 144.5 (C_4,5_), 134.8 and 133.9 (C_2,12_), 131.6 and 131.2 (C_1,11_), 110.2 and 108.6 (C_3,13_), 103.2 and 101.8 (C_6,16_), 70.3 and 70.2 (C_4a,5a_), 57.1 (C_14a,15a_), 32.1 and 32.0 (C_4d,5d_), 29.6 and 29.4 (C_4b,5b_), 26.3 and 26.2 (C_4c,5c_), 23.2 and 23.1 (C_4e,5e_), 14.6 and 14.5 (C_4f,5f_). FT-IR (ATR, *v*_max_, (neat)/cm^–1^): 3462, 3335, 3284, 3104, 2952, 2928, 2869, 2856, 1622, 1579, 1506, 1464, 1405, 1391, 1371, 1318, 1253, 1227, 1186, 1078, 1067, 1026, 995, 950, 926, 897, 859, 848, 820, 799, 779, 755. HRMS (ESI): *m*/*z* calcd for C_26_H_38_N_3_O_8_ [M + H]^+^, 520.2654; found, 520.2655.

### General Procedure for the Synthesis of Phenazines **5**, **6**, and **7**

Compound **2**, **3**, or **4** (1 equiv, 0.2 mmol) and palladium on charcoal (10% Pd, 13 mg, 0.05 equiv, 0.01 mmol) were placed in a 50 mL round-bottom flask. To this, methanol (∼30 mL) was added and the resulting mixture was heated to the point of gentle boiling on a heating mantle, where sodium tetrahydroborate was added in small portions (around 10 mg) until the solution became colorless. The solution was then filtered through silica gel directly into a 50 mL round-bottom flask containing a solution of hydrochloric acid (10%, ∼5 mL). The solution was then concentrated on a rotatory evaporator, and hydrochloric acid (10%, 10 mL) was then added. To the solution, ferric(III) chloride (195 mg, 3.6 equiv, 0.72 mmol) was added and the mixture was stirred overnight at room temperature. After this time, the mixture was diluted with water (100 mL) and extracted three times using DCM. The combined organic phases were washed with water and brine and then dried over anhydrous magnesium sulfate, before removal of the solvent using a rotatory evaporator. The crude product was then purified by column chromatography on silica gel with a gradient elution of DCM/methanol (1:0 to 95:5).

### 7-(*tert*-Butyl)-3-isobutoxy-2-methoxyphenazine (**5a**)

The general procedure resulted in a yellow powder with 81% (55 mg) yield of titled compound. mp = 45–47 °C. ^1^H NMR (CDCl_3_, 300 MHz, δ ppm): 8.08 (d, ^3^*J*_H8–H9_ = 9.43 Hz, 1H, H_9_), 8.06 (d, ^4^*J*_H6–H8_ = 2.13 Hz, 1H, H_6_), 7.85 (dd, ^3^*J*_H8–H9_ = 9.43 Hz, ^4^*J*_H8–H6_ = 2.13 Hz, 1H, H_8_), 7.39 (s, 1H, H_4_), 7.36 (s, 1H, H_1_), 4.09 (s, 3H, H_2a_), 4.02 (d, ^3^*J*_H3a–H3b_ = 6.53 Hz, 2H, H_3a_), 2.37–2.28 (m, 1H, H_3b_), 1.47 (s, 9H, H_7b_), 1.10 (d, ^3^*J*_H3b–H3c_ = 6.64 Hz, 6H, H_3c_). ^13^C{^1^H} NMR (CDCl_3_, 75 MHz, δ ppm): 155.2 and 154.6 (C_2,3_), 152.9 (C_5a_), 142.6 and 142.0 (C_4a,10a_), 142.5 (C_9a_), 141.2 (C_7_), 129.1 (C_8_), 128.8 (C_9_), 124.1 (C_6_), 106.4 and 105.9 (C_1,4_), 76.1 (C_3a_), 57.0 (C_2a_), 35.9 (C_7a_), 31.6 (C_7b_), 28.4 (C_3b_), 19.9 (C_3c_). FT-IR (ATR, *v*_max_, (neat)/cm^–1^): 3252, 3087, 3061, 3002, 2958, 2928, 2904, 2867, 1636, 1608, 1566, 1517, 1488, 1463, 1437, 1419, 1392, 1364, 1328, 1308, 1251, 1211, 1197, 1177, 1159, 1136, 1086, 1031, 1013, 966, 950, 905, 879, 855, 833, 818, 783. HRMS (ESI): *m*/*z* calcd for C_21_H_27_N_2_O_2_ [M + H]^+^, 339.2068; found, 339.2065.

### 7-(*tert*-Butyl)-2-ethoxy-3-isobutoxyphenazine (**5b**)

The general procedure resulted in a yellow powder with 82% (58 mg) yield of titled compound. mp = 43–45 °C. ^1^H NMR (CDCl_3_, 300 MHz, δ ppm): 8.08 (d, ^3^*J*_H8–H9_ = 9.17 Hz, 1H, H_9_), 8.06 (d, ^4^*J*_H6–H8_ = 2.05 Hz, 1H, H_6_), 7.84 (dd, ^3^*J*_H8–H9_ = 9.17 Hz, ^4^*J*_H8–H6_ = 2.05 Hz, 1H, H_8_), 7.35 (s, 1H, H_4_), 7.33 (s, 1H, H_1_), 4.30 (q, ^3^*J*_H2a–H2b_ = 6.98 Hz, 2H, H_2a_), 3.99 (d, ^3^*J*_H3a–H3b_ = 6.71 Hz, 2H, H_3a_), 2.37–2.23 (m, 1H, H_3b_), 1.57 (t, ^3^J_H2b–H2a_ = 6.98 Hz, 3H, H_2b_), 1.47 (s, 9H, H_7b_), 1.11 (d, ^3^*J*_H3b–H3c_ = 6.71 Hz, 6H, H_3c_). ^13^C{^1^H} NMR (CDCl_3_, 75 MHz, δ ppm): 154.8 and 154.6 (C_2,3_), 152.7 (C_5a_), 142.5 (C_9a_), 142.1 (C_4a,10a_), 141.1 (C_7_), 129.0 (C_8_), 128.7 (C_9_), 125.4 (C_6_), 106.3 (C_1,4_), 76.0 (C_3a_), 65.3 (C_2a_), 35.9 (C_7a_), 31.6 (C_7b_), 28.5 (C_3b_), 19.8 (C_3c_), 15.1 (C_2a_). FT-IR (ATR, *v*_max_, (neat)/cm^–1^): 3332, 2976, 2955, 2926, 2872, 2855, 1743, 1696, 1635, 1608, 1569, 1522, 1489, 1478, 1468, 1441, 1394, 1368, 1328, 1315, 1255, 1226, 1217, 1199, 1182,1109, 1092, 1043, 1029, 959, 932, 894, 860, 853, 841, 823, 787. HRMS (ESI): *m*/*z* calcd for C_22_H_29_N_2_O_2_ [M + H]^+^, 353.2224; found, 353.2223.

### 7-(*tert*-Butyl)-3-ethoxy-2-isobutoxyphenazine (**5c**)

The general procedure resulted in a yellow powder with 88% (62 mg) yield of titled compound. mp = 45–48 °C. ^1^H NMR (CDCl_3_, 300 MHz, δ ppm): 8.07 (d, ^3^*J*_H8–H9_ = 9.07 Hz, 1H, H_9_), 8.06 (d, ^4^*J*_H6–H8_ = 1.96 Hz, 1H, H_6_), 7.85 (dd, ^3^*J*_H8–H9_ = 9.07 Hz, ^4^*J*_H8–H6_ = 1.96 Hz, 1H, H_8_), 7.33 (s, 2H, H_1,4_), 4.30 (q, ^3^*J*_H3a–H3b_ = 6.97 Hz, 2H, H_3a_), 3.99 (d, ^3^*J*_H2a–H2b_ = 6.76 Hz, 2H, H_2a_), 2.26–2.35 (m, 1H, H_2b_), 1.59 (t, ^3^*J*_H3a–H3b_ = 6.98 Hz, 3H, H_3b_), 1.48 (s, 9H, H_7b_), 1.12 (d, ^3^*J*_H2b–H2c_ = 6.78 Hz, 6H, H_2c_). ^13^C{^1^H} NMR (CDCl_3_, 75 MHz, δ ppm): 154.8 and 154.6 (C_2,3_), 152.8 (C_5a_), 142.5 (C_9a_), 142.4 and 142.1 (C_4a,10a_), 141.1 (C_7_), 129.0 (C_8_), 128.7 (C_9_), 124.0 (C_6_), 106.3 and 106.2 (C_1,4_), 76.0 (C_2a_), 65.3 (C_3a_), 35.9 (C_7a_), 31.6 (C_7b_), 28.5 (C_2b_), 19.8 (C_2c_), 15.0 (C_3b_). FT-IR (ATR, *v*_max_, (neat)/cm^–1^): 3401, 3243, 2960, 2928, 2874, 2854, 1743, 1636, 1612, 1572, 1523, 1490, 1470, 1441, 1394, 1369, 1330, 1314, 1256, 1229, 1216, 1197, 1186, 1147, 1110, 1089, 1045, 1024, 996, 966, 937, 893, 839, 819. HRMS (ESI): *m*/*z* calcd for C_22_H_29_N_2_O_2_ [M + H]^+^, 353.2224; found, 353.2222.

### 2-Butoxy-7-(*tert*-butyl)-3-isobutoxyphenazine (**5d**)

The general procedure resulted in a light-yellow powder with 55% (42 mg) yield of titled compound. mp = 47–49 °C. ^1^H NMR (CDCl_3_, 300 MHz, δ ppm): 8.08 (d, ^3^*J*_H8–H9_ = 9.17 Hz, 1H, H_9_), 8.07 (d, ^4^*J*_H6–H8_ = 2.20 Hz, 1H, H_6_), 7.85 (dd, ^3^*J*_H8–H9_ = 9.17 Hz, ^4^*J*_H8–H6_ = 2.20 Hz, 1H, H_8_), 7.35 (s, 1H, H_4_), 7.33 (s, 1H, H_1_), 4.24 (t, ^3^*J*_H2a–H2b_ = 6.42 Hz, 2H, H_2a_), 3.99 (d, ^3^*J*_H3a–H3b_ = 6.52 Hz, 2H, H_3a_), 2.29 (m, 1H, H_3b_), 2.01–1.89 (m, 2H, H_2b_), 1.65–1.51 (m, 2H, H_2c_), 1.49 (s, 9H, H_7b_), 1.12 (d, ^3^*J*_H3b–H3c_ = 6.55 Hz, 6H, H_3c_), 1.04 (t, ^3^*J*_H2c–H2d_ = 7.49 Hz, 3H, H_2d_). ^13^C{^1^H} NMR (CDCl_3_, 75 MHz, δ ppm): 155.6 and 154.9 (C_2,3_), 152.7 (C_5a_), 142.5 (C_9a_), 142.5 and 142.2 (C_4a,10a_), 141.1 (C_7_), 129.0 (C_8_), 128.7 (C_9_), 124.1 (C_6_), 106.3 and 106.2 (C_1,4_), 75.9 (C_3a_), 69.5 (C_2a_), 35.9 (C_7a_), 31.6 (C_7b_), 31.5 (C_2b_), 28.6 (C_3b_), 19.9 (C_2c_), 19.8 (C_3c_), 14.5 (C_2d_). FT-IR (ATR, *v*_max_, (neat)/cm^–1^): 2958, 2925, 2873, 2854, 1744, 1634, 1608, 1565, 1519, 1488, 1463, 1436, 1393, 1364, 1327, 1308, 1250, 1213, 1196, 1176, 1143, 1083, 1022, 969, 914, 826, 722. HRMS (ESI): *m*/*z* calcd for C_24_H_33_N_2_O_2_ [M + H]^+^, 381.2537; found, 381.2538.

### 7-(*tert*-Butyl)-2-(hexyloxy)-3-isobutoxyphenazine (**5e**)

The general procedure resulted in a light-yellow powder with 77% (63 mg) yield of titled compound. mp = 38–40 °C. ^1^H NMR (CDCl_3_, 300 MHz, δ ppm): 8.08 (d, ^3^*J*_H8–H9_ = 9.17 Hz, 1H, H_9_), 8.07 (d, ^4^*J*_H6–H8_ = 2.05 Hz, 1H, H_6_), 7.85 (dd, ^3^*J*_H8–H9_ = 9.17 Hz, ^4^*J*_H8–H6_ = 2.05 Hz, 1H, H_8_), 7.35 (s, 1H, H_4_), 7.33 (s, 1H, H_1_), 4.22 (t, 2H, H_2a_), 3.99 (d, ^3^*J*_H3a–H3b_ = 6.71 Hz, 2H, H_3a_), 2.35–2.24 (m, 1H, H_3b_), 2.00–1.90 (m, 2H, H_2b_), 1.63–1.52 (m, 2H, H_2c_), 1.49 (s, 9H, H_7b_), 1.44–1.32 (m, 4H, H_2d,2e_), 1.12 (d, ^3^*J*_H3b–H3c_ = 6.71 Hz, 6H, H_3c_), 0.93 (t, ^3^*J*_H2e–H2f_ = 6.98 Hz, 3H, H_2f_). ^13^C{^1^H} NMR (CDCl_3_, 75 MHz, δ ppm): 155.0 (C_2,3_), 152.7 (C_5a_), 142.5 (C_9a_), 142.4 and 142.2 (C_4a,10a_), 141.1 (C_7_), 129.0 (C_8_), 128.7 (C_9_), 124.1 (C_6_), 106.2 and 106.1 (C_1,4_), 75.9 (C_3a_), 69.7 (C_2a_), 35.9 (C_7a_), 32.1 (C_2d_), 31.6 (C_7b_), 30.3 (C_2b_), 29.4 (C_3b_), 26.4 (C_2c_), 23.2 (C_2e_), 19.8 (C_3c_), 14.6 (C_2f_). FT-IR (ATR, *v*_max_, (neat)/cm^–1^): 2957, 2926, 2871, 2855, 1743, 1634, 1608, 1565, 1519, 1488, 1464, 1436, 1393, 1365, 1327, 1308, 1250, 1213, 1196, 1176, 1143, 1084, 1022. HRMS (ESI): *m*/*z* calcd for C_26_H_37_N_2_O_2_ [M + H]^+^, 409.2850; found, 409.2849.

### 7-(*tert*-Butyl)-3-isobutoxy-2-(octyloxy)phenazine (**5f**)

The general procedure resulted in a light-yellow powder with 65% (57 mg) yield of titled compound. mp = 67–68 °C. ^1^H NMR (CDCl_3_, 300 MHz, δ ppm): 8.07 (d, ^3^*J*_H8–H9_ = 9.22 Hz, 1H, H_9_), 8.06 (d, ^3^*J*_H6–H8_ = 2.22 Hz, 1H, H_6_), 7.85 (dd, ^3^*J*_H8–H9_ = 9.20 Hz, ^3^*J*_H8–H6_ = 2.22 Hz, 1H, H_8_), 7.34 (s, 1H, H_4_), 7.33 (s, 1H, H_1_), 4.22 (t, ^3^*J*_H2a–H2b_ = 6.51 Hz, 2H, H_2a_), 3.99 (d, ^3^*J*_H3a–H3b_ = 6.62 Hz, 2H, H_3a_), 2.28 (m, 1H, H_3b_), 2.06–1.88 (m, 2H, H_2b_), 1.60–1.49 (m, 2H, H_2c_), 1.48 (s, 9H, H_7b_), 1.45–1.25 (m, 8H, H_2d,2e,2f,2g_), 1.12 (d, ^3^*J*_H3b–H3c_ = 6.87 Hz, 6H, H_3c_), 0.90 (t, ^3^*J*_H2g–H2h_ = 6.87 Hz, 3H, H_2h_). ^13^C{^1^H} NMR (CDCl_3_, 75 MHz, δ ppm): 154.9 and 154.8 (C_2,3_), 152.7 (C_5a_), 142.5 (C_9a_), 142.4 and 142.2 (C_4a,10a_), 141.1 (C_7_), 129.0 (C_8_), 128.7 (C_9_), 124.1 (C_6_), 106.3 and 106.2 (C_1,4_), 75.9 (C_3a_), 69.7 (C_2a_), 35.9 (C_7a_), 32.4 (C_2f_), 31.6 (C_7b_), 30.3 (C_2b_), 29.9 (C_2d_), 29.5 (C_2e_), 28.6 (C_3b_), 26.7 (C_2c_), 23.3 (C_2g_), 19.8 (C_3c_), 14.7 (C_2h_). FT-IR (ATR, *v*_max_, (neat)/cm^–1^): 3085, 3056, 1976, 2954, 2921, 2871, 2855, 1744, 1634, 1607, 1562, 1519, 1489, 1466, 1434, 1392, 1364, 1327, 1307, 1297, 1249, 1213, 1195, 1177, 1140, 1084, 1038, 1022, 997, 948, 908, 885, 843, 829, 725. HRMS (ESI): *m*/*z* calcd for C_28_H_41_N_2_O_2_ [M + H]^+^, 437.3163; found, 437.3162.

### 7-(*tert*-Butyl)-2-(decyloxy)-3-isobutoxyphenazine (**5g**)

The general procedure resulted in a light-yellow viscous oil with 77% (72 mg) yield of titled compound. mp – compound does not solidify at room temperature. ^1^H NMR (CDCl_3_, 300 MHz, δ ppm): 8.07 (d, ^3^*J*_H8–H9_ = 9.18 Hz, 1H, H_9_), 8.06 (d, ^4^*J*_H6–H8_ = 2.26 Hz, 1H, H_6_), 7.84 (dd, ^3^*J*_H8–H9_ = 9.18 Hz, ^4^*J*_H8–H6_ = 2.26 Hz, 1H, H_8_), 7.34 (s, 1H, H_4_), 7.32 (s, 1H, H_1_), 4.21 (t, ^3^*J*_H2a–H2b_ = 6.45 Hz, 2H, H_2a_), 3.98 (d, ^3^*J*_H3a–H3b_ = 6.70 Hz, 2H, H_3a_), 2.28 (m, 1H, H_3b_), 2.00–1.88 (m, 2H, H_2b_), 1.60–1.49 (m, 2H, H_2c_), 1.48 (s, 9H, H_7b_), 1.45–1.22 (m, 12H, H_2d,2e,2f,2g,2h,2i_), 1.11 (d, ^3^*J*_H3b–H3c_ = 6.93 Hz, 6H, H_3c_), 0.89 (t, ^3^*J*_H2i–H2j_ = 6.66 Hz, 3H, H_2j_). ^13^C{^1^H} NMR (CDCl_3_, 75 MHz, δ ppm): 154.9 and 154.7 (C_2,3_), 152.7 (C_5a_), 142.5 (C_9a_), 142.4 and 142.2 (C_4a,10a_), 141.1 (C_7_), 128.9 (C_8_), 128.7 (C_9_), 124.1 (C_6_), 106.3 and 106.2 (C_1,4_), 75.8 (C_3a_), 69.7 (C_2a_), 35.9 (C_7a_), 32.4 (C_2h_), 31.6 (C_7b_), 30.4–29.8 (m, 4C, C_2b,2d,2e,2f_), 29.4 (C_2g_), 28.6 (C_3b_), 26.7 (C_2c_), 23.3 (C_2i_), 19.8 (C_3c_), 14.7 (C_2j_). FT-IR (ATR, *v*_max_, (neat)/cm^–1^): 3087, 3061, 2957, 2926, 2871, 2855, 1634, 1607, 1565, 1519, 1487, 1463, 1436, 1393, 1364, 1327, 1308, 1250, 1212, 1196, 1175, 1142, 1084, 1022, 994, 950, 911, 885, 840, 826, 792, 765, 750. HRMS (ESI): *m*/*z* calcd for C_30_H_45_N_2_O_2_ [M + H]^+^, 465.3476; found, 465.3475.

### 2-Isobutoxy-3-methoxy-7-(trifluoromethyl)phenazine (**6a**)

The general procedure resulted in a yellow powder with 61% (43 mg) yield of titled compound. mp = 145–147 °C. ^1^H NMR (CDCl_3_, 300 MHz, δ ppm): 8.48 (d, ^4^*J*_H6–H8_ = 2.11 Hz, 1H, H_6_), 8.25 (d, ^3^*J*_H8–H9_ = 9.06 Hz, 1H, H_9_), 7.88 (dd, ^3^*J*_H8–H9_ = 9.06 Hz, ^4^*J*_H8–H6_ = 2.11 Hz, 1H, H_8_), 7.39 (s, 2H, H_1,4_), 4.12 (s, 3H, H_3a_), 4.03 (d, ^3^*J*_H2a–H2b_ = 6.83 Hz, 2H, H_2a_), 2.38–2.29 (m, 1H, H_2b_), 1.13 (d, ^3^*J*_H2b–H2c_ = 6.69 Hz, 6H, H_2c_). ^13^C{^1^H} NMR (CDCl_3_, 75 MHz, δ ppm): 156.4 (C_2_), 156.0 (C_3_), 144.0 and 143.5 (C_4a,10a_), 143.1 (C_7_), 141.0 (C_9a_), 130.8 (C_9_), 127.7 (q, ^3^*J*_C–F_ = 4 Hz, C_6_), 124.7 (q, ^3^*J*_C–F_ = 3 Hz, C_8_), 106.1 (C_1_), 105.6 (C_4_), 76.4 (C_2a_), 57.2 (C_3a_), 28.5 (C_2b_), 19.6 (C_2c_), signals from C_7a_ and C_5a_ are missing. ^19^F NMR (CDCl_3_, 282 MHz, δ ppm): −63.84 (s, 3F, F_CF3_). FT-IR (ATR, *v*_max_, (neat)/cm^–1^): 3092, 3031, 2975, 2963, 2917,2875, 2851, 2836, 1642, 1612, 1567, 1527, 1488, 1464, 1448, 1429, 1417, 1394, 1369, 1341, 1327, 1283, 1269, 1255, 1221, 1194, 1162, 1141, 1111, 1052, 1014, 972, 959, 942, 905, 893, 837, 825, 790, 750, 735. HRMS (ESI): *m*/*z* calcd for C_18_H_18_N_2_O_2_F_3_ [M + H]^+^, 351.1315; found, 351.1313.

### 3-Ethoxy-2-isobutoxy-7-(trifluoromethyl)phenazine (**6b**)

The general procedure resulted in a yellow powder with 79% (58 mg) yield of titled compound. mp = 131–132 °C. ^1^H NMR (CDCl_3_, 300 MHz, δ ppm): 8.46 (d, ^4^*J*_H6–H8_ = 2.09 Hz, 1H, H_6_), 8.23 (d, ^3^*J*_H8–H9_ = 9.00 Hz, 1H, H_9_), 7.87 (dd, ^3^*J*_H8–H9_ = 9.00 Hz, ^4^*J*_H8–H6_ = 2.09 Hz, 1H, H_8_), 7.33 (s, 2H, H_1,4_), 4.33 (q, ^3^*J*_H3a–H3b_ = 6.95 Hz, 2H, H_3a_), 4.01 (d, ^3^*J*_H2a–H2b_ = 6.68 Hz, 2H, H_2a_), 2.31 (m, 1H, H_2b_), 1.60 (t, ^3^*J*_H3a–H2b_ = 6.89 Hz, 3H, H_3b_), 1.13 (d, ^3^*J*_H2b–H2c_ = 6.83 Hz, 6H, H_2c_). ^13^C{^1^H} NMR (CDCl_3_, 75 MHz, δ ppm): 156.2 (C_2_), 155.8 (C_3_), 143.9 and 143.6 (C_4a,10a_), 143.0 (C_7_), 141.0 (C_9a_), 130.8 (C_9_), 127.7 (q, ^3^*J*_C–F_ = 4 Hz, C_6_), 124.6 (q, ^3^*J*_C–F_ = 3 Hz, C_8_), 106.1 (C_1,4_), 76.2 (C_2a_), 65.6 (C_3a_), 28.6 (C_2b_), 19.8 (C_2c_), 15.0 (C_3b_), signals from C_7a_ and C_5a_ are missing. ^19^F NMR (CDCl_3_, 282 MHz, δ ppm): −63.84 (s, 3F, F_CF3_). FT-IR (ATR, *v*_max_, (neat)/cm^–1^): 3109, 3032, 3015, 2960, 2927, 2875, 2851, 1640, 1611, 1566, 1525, 1490, 1466, 1451, 1417, 1392, 1367, 1337, 1325, 1283, 1267, 1253, 1219, 1201, 1184, 1153, 1142, 1110, 1058, 1044, 1016, 972, 943, 933, 908, 889, 850, 822, 789, 751. HRMS (ESI): *m*/*z* calcd for C_19_H_20_N_2_O_2_F_3_ [M + H]^+^, 365.1472; found, 365.1469.

### 2-Ethoxy-3-isobutoxy-7-(trifluoromethyl)phenazine (**6c**)

The general procedure resulted in a yellow powder with 69% (51 mg) yield of titled compound. mp = 154–157 °C. ^1^H NMR (CDCl_3_, 300 MHz, δ ppm): 8.46 (d, ^4^*J*_H6–H8_ = 2.09 Hz, 1H, H_6_), 8.23 (d, ^3^*J*_H8–H9_ = 9.00 Hz, 1H, H_9_), 7.87 (dd, ^3^*J*_H8–H9_ = 9.00 Hz, ^4^*J*_H8–H6_ = 2.09 Hz, 1H, H_8_), 7.33 (s, 2H, H_1,4_), 4.33 (q, ^3^*J*_H2a–H2b_ = 6.95 Hz, 2H, H_2a_), 4.01 (d, ^3^*J*_H3a–H3b_ = 6.68 Hz, 2H, H_3a_), 2.31 (m, 1H, H_3b_), 1.60 (t, ^3^*J*_H2a–H2b_ = 6.89 Hz, 3H, H_2b_), 1.13 (d, ^3^*J*_H3b–H3c_ = 6.83 Hz, 6H, H_3c_). ^13^C{^1^H} NMR (CDCl_3_, 75 MHz, δ ppm): 156.2 (C_2_), 155.8 (C_3_), 143.9 and 143.6 (C_4a,10a_), 143.0 (C_7_), 141.0 (C_9a_), 130.8 (C_9_), 127.7 (q, ^3^*J*_C–F_ = 4 Hz, C_6_), 124.6 (q, ^3^*J*_C–F_ = 3 Hz, C_8_), 106.1 (C_1,4_), 76.2 (C_3a_), 65.6 (C_2a_), 28.6 (C_3b_), 19.8 (C_3c_), 15.0 (C_2b_), signals from C_7a_ and C_5a_ are missing.^19^F NMR (CDCl_3_, 282 MHz, δ ppm): −63.83 (s, 3F, F_CF3_). FT-IR (ATR, *v*_max_, (neat)/cm^–1^): 3105, 3039, 2965, 2936, 2897, 2875, 1641, 1611, 1572, 1524, 1490, 1468, 1451, 1417, 1393, 1368, 1338, 1326, 1301, 1283, 1283, 1267, 1251, 1220, 1189, 1178, 1150, 1141, 1102, 1057, 1043, 1024, 1000, 946, 928, 904, 890, 849, 928, 904, 890, 849, 830, 787, 751. HRMS (ESI): *m*/*z* calcd for C_19_H_20_N_2_O_2_F_3_ [M + H]^+^, 365.1472; found, 365.1471.

### 3-Butoxy-2-isobutoxy-7-(trifluoromethyl)phenazine (**6d**)

The general procedure resulted in a light-yellow powder with 65% (51 mg) yield of titled compound. mp = 123.0–124.5 °C. ^1^H NMR (CDCl_3_, 300 MHz, δ ppm): 8.46 (d, ^4^*J*_H6–H8_ = 2.04 Hz, 1H, H_6_), 8.24 (d, ^3^*J*_H8–H9_ = 9.17 Hz, 1H, H_9_), 7.87 (dd, ^3^*J*_H8–H9_ = 9.17 Hz, ^4^*J*_H8–H6_ = 2.04 Hz, 1H, H_8_), 7.33 (2× s, 2× 1H, H_1,4_), 4.33 (q, ^3^*J*_H3a–H3b_ = 6.95 Hz, 2H, H_3a_), 4.01 (d, ^3^*J*_H2a–H2b_ = 6.68 Hz, 2H, H_2a_), 2.30 (m, 1H, H_2b_), 2.01–1.90 (m, 2H, H_3b_), 1.64–1.54 (m, 2H, H_3c_), 1.13 (d, ^3^*J*_H2b–H2c_ = 6.78 Hz, 6H, H_2c_), 1.05 (t, ^3^*J*_H3c–H3d_ = 7.32 Hz, 3H, H_3d_). ^13^C{^1^H} NMR (CDCl_3_, 75 MHz, δ ppm): 156.3 (C_2_), 156.0 (C_3_), 144.0 and 143.6 (C_4a,10a_), 143.0 (C_7_), 141.0 (C_9a_), 130.8 (C_9_), 127.7 (q, ^3^*J*_C–F_ = 4 Hz, C_6_), 124.6 (q, ^3^*J*_C–F_ = 3 Hz, C_8_), 106.0 (C_4,1_), 76.1 (C_2a_), 69.7 (C_3a_), 31.4 (C_3b_), 28.6 (C_2b_), 19.9 (C_3c_), 19.8 (C_2c_), 14.4 (C_3d_), signals from C_7a_ and C_5a_ are missing. ^19^F NMR (CDCl_3_, 282 MHz, δ ppm): −63.81 (s, 3F, F_CF3_). FT-IR (ATR, *v*_max_, (neat)/cm^–1^): 3106, 3034, 3015, 2957, 2932, 2874, 1640, 1611, 1570, 1524, 1489, 1466, 1451, 1417, 1392, 1367, 1341, 1327, 1283, 1267, 1254, 1219, 1203, 1187, 1152, 1141, 1103, 1058, 1023, 1005, 967, 942, 920, 908, 852, 836, 824, 787, 751. HRMS (ESI): *m*/*z* calcd for C_21_H_24_N_2_O_2_F_3_ [M + H]^+^, 393.1785; found, 393.1782.

### 3-(Hexyloxy)-2-isobutoxy-7-(trifluoromethyl)phenazine (**6e**)

The general procedure resulted in a light-yellow powder with 70% (59 mg) yield of titled compound. mp = 95–96 °C. ^1^H NMR (CDCl_3_, 300 MHz, δ ppm): 8.46 (d, ^4^*J*_H6–H8_ = 2.23 Hz, 1H, H_6_), 8.24 (d, ^3^*J*_H8–H9_ = 9.07 Hz, 1H, H_9_), 7.87 (dd, ^3^*J*_H8–H9_ = 9.07 Hz, ^4^*J*_H8–H6_ = 2.23 Hz, 1H, H_8_), 7.34 (s, 1H, H_1_), 7.33 (s, 1H, H_4_), 4.24 (t, ^3^*J*_H3a–H3b_ = 6.36 Hz, 2H, H_3a_), 4.01 (d, ^3^*J*_H2a–H2b_ = 6.55 Hz, 2H, H_2a_), 2.30 (m, 1H, H_2b_), 2.02–1.91 (m, 2H, H_3b_), 1.63–1.52 (m, 2H, H_3c_), 1.45–1.36 (m, 4H, H_3d,3e_), 1.13 (d, ^3^*J*_H2b–H2c_ = 6.77 Hz, 6H, H_2c_), 0.94 (t, ^3^*J*_H3e–H3f_ = 7.09 Hz, 3H, H_3f_). ^13^C{^1^H} NMR (CDCl_3_, 75 MHz, δ ppm): 156.3 (C_2_), 156.0 (C_3_), 144.0 and 143.6 (C_4a,10a_), 143.0 (C_7_), 141.0 (C_9a_), 130.7 (C_9_), 127.7 (q, ^3^*J*_C–F_ = 4 Hz, C_6_), 124.5 (q, ^3^*J*_C–F_ = 3 Hz, C_8_), 106.1 (C_1_), 105.6 (C_4_), 76.1 (C_2a_), 70.0 (C_3a_), 32.1 (C_3d_), 29.3 (C_3b_), 28.6 (C_2b_), 26.3 (C_3c_), 23.2 (C_3e_), 19.8 (C_2c_), 14.6 (C_3f_), signals from C_7a_ and C_5a_ are missing. ^19^F NMR (CDCl_3_, 282 MHz, δ ppm): −63.82 (s, 3F, F_CF3_). FT-IR (ATR, *v*_max_, (neat)/cm^–1^): 3106, 3033, 3017, 2958, 2931, 2873, 2858, 1640, 1611, 1565, 1525, 1488, 1464, 1452, 1417, 1397, 1384, 1338, 1327, 1284, 1268, 1255, 1219, 1202, 1185, 1155, 1139, 1111, 1058, 1022, 997, 940, 909, 851, 825. HRMS (ESI): *m*/*z* calcd for C_23_H_28_N_2_O_2_F_3_ [M + H]^+^, 421.2098; found, 421.2096.

### 2-Isobutoxy-3-(octyloxy)-7-(trifluoromethyl)phenazine (**6f**)

The general procedure resulted in a light-yellow powder with 67% (61 mg) yield of titled compound. mp = 88–90 °C. ^1^H NMR (CDCl_3_, 300 MHz, δ ppm): 8.46 (d, ^4^*J*_H6–H8_ = 2.11 Hz, 1H, H_6_), 8.24 (d, ^3^*J*_H8–H9_ = 9.03 Hz, 1H, H_9_), 7.87 (dd, ^3^*J*_H8–H9_ = 9.03 Hz, ^4^*J*_H8–H6_ = 2.11 Hz, 1H, H_8_), 7.33 (2× s, 2× 1H, H_1,4_), 4.24 (t, ^3^*J*_H3a–H3b_ = 6.45 Hz, 2H, H_3a_), 4.01 (d, ^3^*J*_H2a–H2b_ = 6.64 Hz, 2H, H_2a_), 2.30 (m, 1H, H_2b_), 2.06–1.92 (m, 2H, H_3b_), 1.68–1.50 (m, 2H, H_3c_), 1.49–1.21 (m, 8H, H_3d,3e,3f,3g_), 1.13 (d, ^3^*J*_H2b–H2c_ = 6.64 Hz, 6H, H_2c_), 0.90 (t, ^3^*J*_H3g–H3h_ = 7.40 Hz, 3H, H_3h_). ^13^C{1H} NMR (CDCl_3_, 75 MHz, δ ppm): 156.3 (C_2_), 156.0 (C_3_), 144.0 and 143.6 (C_4a,10a_), 143.0 (C_7_), 141.0 (C_9a_), 130.8 (C_9_), 127.7 (q, ^3^*J*_C–F_ = 4 Hz, C_6_), 124.5 (q, ^3^*J*_C–F_ = 3 Hz, C_8_), 106.0 (C_1,4_), 76.1 (C_2a_), 70.0 (C_3a_), 32.4 (C_3f_), 30.9 (C_3d_), 30.3 (C_3b_), 29.9 (C_3e_), 28.6 (C_2b_), 26.4 (C_3c_), 23.3 (C_3g_), 19.8 (C_2c_), 14.7 (C_3h_), signals from C_7a_ and C_5a_ are missing. ^19^F NMR (CDCl_3_, 282 MHz, δ ppm): −63.82 (s, 3F, F_CF3_). FT-IR (ATR, *v*_max_, (neat)/cm^–1^): 3108, 3033, 3015, 2959, 2927, 2873, 2857, 1640, 1611, 1565, 1524, 1488, 1465, 1524, 1488, 1465, 1452, 1417, 1397, 1304, 1367, 1338, 1327, 1285, 1268, 1256, 1220, 1204, 1185, 1155, 1140, 1112, 1059, 1022, 970, 943, 910, 852, 825, 792, 752, 725. HRMS (ESI): *m*/*z* calcd for C_25_H_32_N_2_O_2_F_3_ [M + H]^+^, 449.2411; found, 449.2408.

### 3-(Decyloxy)-2-isobutoxy-7-(trifluoromethyl)phenazine (**6g**)

The general procedure resulted in a light-yellow powder with 60% (58 mg) yield of titled compound. mp = 79–81 °C. ^1^H NMR (CDCl_3_, 300 MHz, δ ppm): 8.46 (d, ^4^*J*_H6–H8_ = 2.03 Hz, 1H, H_6_), 8.24 (d, ^3^*J*_H8–H9_ = 8.96 Hz, 1H, H_9_), 7.87 (dd, ^3^*J*_H8–H9_ = 8.96 Hz, ^3^*J*_H8–H6_ = 2.03 Hz, 1H, H_8_), 7.34 (s, 1H, H_1_), 7.33 (s, 1H, H_4_), 4.25 (t, ^3^*J*_H3a–H3b_ = 6.56 Hz, 2H, H_3a_), 4.01 (d, ^3^*J*_H2a–H2b_ = 6.56 Hz, 2H, H_2a_), 2.30 (m, 1H, H_2b_), 2.08–1.91 (m, 2H, H_3b_), 1.65–1.50 (m, 2H, H_3c_), 1.48–1.24 (m, 12H, H_3d,3e,3f,3g,3h,3i_), 1.13 (d, ^3^*J*_H2b–H2c_ = 6.74 Hz, 6H, H_2c_), 0.89 (t, ^3^*J*_H3i–H3j_ = 6.42 Hz, 3H, H_3j_). ^13^C{^1^H} NMR (CDCl_3_, 75 MHz, δ ppm): 156.3 (C_2_), 156.0 (C_3_), 144.0 and 143.7 (C_4a,10a_), 143.0 (C_7_), 141.0 (C_9a_), 130.7 (C_9_), 127.7 (q, ^3^*J*_C–F_ = 4 Hz, C_6_), 124.6 (q, ^3^*J*_C–F_ = 3 Hz, C_8_), 106.0 (C_1,4_), 76.1 (C_2a_), 70.0 (C_3a_), 32.5 (C_3h_), 30.4–30.0 (C_3b,3d,3e,3f,3g_), 28.6 (C_2b_), 26.6 (C_3c_), 23.3 (C_3i_), 19.8 (C_2c_), 14.7 (C_3j_), signals from C_7a_ and C_5a_ are missing. ^19^F NMR (CDCl_3_, 282 MHz, δ ppm): −63.82 (s, 3F, F_CF3_). FT-IR (ATR, *v*_max_, (neat)/cm^–1^): 3106, 3056, 3033, 3013, 2957, 2924, 2871, 2853, 1640, 1611, 1565, 1524, 1488, 1464, 1448, 1417, 1397, 1384, 1365, 1338, 1327, 1284, 1268, 1256, 1219, 1204, 1186, 1155, 1139, 1112, 1058, 1022, 942, 910, 852, 825, 787, 748, 722. HRMS (ESI): *m*/*z* calcd for C_27_H_36_N_2_O_2_F_3_ [M + H]^+^, 477.2724; found, 477.2720.

### 2,3-Bis(hexyloxy)-7,8-dimethoxyphenazine (**7**)

The general procedure resulted in a yellow powder with 50% (44 mg) yield of titled compound. mp = 106–108 °C. ^1^H NMR (CDCl_3_, 300 MHz, δ ppm): 7.35 (s, 2H, H_6,9_), 7.31 (s, 2H, H_1,4_), 4.21 (t, ^3^*J*_H2a–H2b,H3a–H3b_ = 6.61 Hz, 4H, H_2a,3a_), 4.08 (s, 6H, H_7a,8a_), 2.00–1.89 (m, 4H, H_2b,3b_), 1.61–1.48 (m, 4H, H_2c,3c_), 1.43–1.34 (m, 8H, H_2d,2e,3d,3e_), 0.93 (t, ^3^*J*_H2e–H2f,H3e–H3f_ = 7.10 Hz, 6H, H_2f,3f_). ^13^C{^1^H} NMR (CDCl_3_, 75 MHz, δ ppm): 153.8 (C_2,3_), 153.6 (C_7,8_), 140.5 (C_4a,10a_), 140.2 (C_5a,9a_), 106.4 (C_6,9_), 106.0 (C_1,4_), 69.8 (C_2a,3a_), 56.9 (C_7a,8a_), 32.2 (C_2d,3d_), 29.4 (C_2b,3b_), 26.4 (C_2c,3c_), 23.2 (C_2e,3e_), 14.6 (C_2f,3f_). FT-IR (ATR, *v*_max_, (neat)/cm^–1^): 3093, 3076, 3011, 3002, 2950, 2926, 2864, 2855, 2830, 1738, 1671, 1635, 1593, 1528, 1485, 1464, 1436, 1422, 1387, 1363, 1287, 1267, 1237, 1206, 1186, 1154, 1074, 1043, 1032, 1009, 992, 951, 928, 916, 842, 831, 765, 737, 723. HRMS (ESI): *m*/*z* calcd for C_19_H_20_N_2_O_2_F_3_ [M + H]^+^, 441.2748; found, 441.2748.

### Procedure for the Synthesis of 4-(Hexyloxy)-5-isobutoxy-2-nitro-*N*-(2-nitro-4-(trifluoromethyl)phenyl)aniline (**3e**) on 2 mmol Scale

To a 18 mL threaded tube, **1e** (621 mg, 1 equiv, 2 mmol), palladium(II) acetate (24 mg, 0.06 equiv, 0.11 mmol), RuPhos (50 mg, 0.06 equiv, 0.11 mmol), caesium carbonate (2.6 g, 4 equiv, 8 mmol), and 1-bromo-2-nitro-4-(trifluoromethyl)benzene (540 mg, 1 equiv, 2 mmol) were added. The tube was then flushed several times with argon before adding toluene (10 mL) and flushing again. The tube was sealed, and the mixture was heated at 110 °C for 48 h on an oil bath. After this time, the reaction mixture was cooled to room temperature, diluted with DCM (10 mL), filtered through a pad of silica gel, and washed out with DCM. The solution was then concentrated on a rotatory evaporator and purified by column chromatography on silica gel with DCM. The procedure resulted in an orange powder with 92% (920 mg) yield of titled compound.

### Procedure for the Synthesis of 3-(Hexyloxy)-2-isobutoxy-7-(trifluoromethyl)phenazine (**6e**) on 1.5 mmol Scale

Compound **3e** (750 mg, 1 equiv, 1.5 mmol) and palladium on charcoal (10% Pd, 70 mg, 0.03 equiv, 0.05 mmol) were placed in a 250 mL round-bottom flask. To this, methanol (∼120 mL) was added and the resulting mixture was heated to the point of gentle boiling on a heating mantle over a magnetic stirrer, where sodium tetrahydroborate was added in small portions (around 50 mg) until the solution became colorless. The solution was then filtered through the pad of silica gel directly into a 250 mL round-bottom flask containing hydrochloric acid (36%, ∼10 mL). Directly to this solution, ferric(III) chloride (1.45 g, 3.6 equiv, 5.4 mmol) dissolved in 5 mL of hot water was added and the mixture was stirred at room temperature for about 20 min (until solution changes color from dark-blue to orange). After this time, the mixture was concentrated to around 50 mL on rotary evaporator, diluted with water (150 mL), and extracted three times using DCM. The combined organic phases were washed with water and brine and then dried over anhydrous magnesium sulfate, before removal of the solvent using a rotatory evaporator. The crude product was then purified by column chromatography on silica gel with a DCM/methanol (95:5). The procedure resulted in a light-yellow powder with 94% (595 mg) yield of titled compound.

## Data Availability

Rest of the data underlying this study is available in the published article and its online Supporting Information.

## Supporting Information Available

^1^H NMR spectra of primary substrates, ^1^H, ^13^C, ^19^F NMR, IR, and HRMS spectra of bis(2-nitrophenyl)amine derivatives, ^1^H, ^13^C, ^19^F NMR, IR, and HRMS spectra of phenazine derivatives, crystallographic data for **5c** hydrate and **6b** and **6b** solvate (PDF)
