# Supplementary material for: Synthesis of Nonsymmetrically Substituted 2,3-Dialkoxyphenazine Derivatives and Preliminary Examination of Their Cytotoxicity
Source: J Org Chem. 2023 Jan 13;88(3):1339–51. doi: 10.1021/acs.joc.2c01901 (PMC9903326; doi:10.1021/acs.joc.2c01901)
Supplement: Supplementary file 1 — jo2c01901_si_001.pdf [file jo2c01901_si_001.pdf]

Supporting information

Synthesis of non-symmetrically substituted 2,3-dialkoxyphenazine derivatives and preliminary examination of their cytotoxicity

Paweł Ręka,[a] Jarosław Grolik,[a]* Katarzyna M. Stadnicka[b], Maria Kołton-Wróż[c] and Paweł Wołkow[c]

[a.] Department of Organic Chemistry, Faculty of Chemistry, Jagiellonian University, 30-387 Kraków, Gronostajowa 2, Poland.

[b.] Department of Crystal Chemistry and Crystal Physics, Faculty of Chemistry, Jagiellonian University, 30-387 Kraków, Gronostajowa 2, Poland.

[c.] Center for Medical Genomics – OMICRON, Jagiellonian University Medical College, 31-034 Kraków, Kopernika 7c, Poland.

*Corresponding author: jaroslaw.grolik@uj.edu.pl

Table of Content:

^{1}H NMR spectra of primary substrates S3
- 4-bromo-1,2-dimethoxybenzene S3
- 1-bromo-4,5-dimethoxy-2-nitrobenzene S4

^{1}H, ^{13}C, ^{19}F NMR, IR and HRMS spectra of bis(2-nitrophenyl)amine derivatives S5
- *N*-(5-(*tert*-butyl)-2-nitrophenyl)-5-isobutoxy-4-methoxy-2-nitroaniline (**2a**) S5
- *N*-(5-(*tert*-butyl)-2-nitrophenyl)-4-ethoxy-5-isobutoxy-2-nitroaniline (**2b**) S8
  - HSQC spectra of (**2b**) S11
  - HMBC spectra of (**2b**) S12
- *N*-(5-(*tert*-butyl)-2-nitrophenyl)-5-ethoxy-4-isobutoxy-2-nitroaniline (**2c**) S14
- 4-butoxy-*N*-(5-(*tert*-butyl)-2-nitrophenyl)-5-isobutoxy-2-nitroaniline (**2d**) S17
- *N*-(5-(*tert*-butyl)-2-nitrophenyl)-4-(hexyloxy)-5-isobutoxy-2-nitroaniline (**2e**) S20
- *N*-(5-(*tert*-butyl)-2-nitrophenyl)-5-isobutoxy-2-nitro-4-(octyloxy)aniline (**2f**) S23
- *N*-(5-(*tert*-butyl)-2-nitrophenyl)-4-(decyloxy)-5-isobutoxy-2-nitroaniline (**2g**) S26
- 5-isobutoxy-4-methoxy-2-nitro-*N*-(2-nitro-4-(trifluoromethyl)phenyl)aniline (**3a**) S29
- 4-ethoxy-5-isobutoxy-2-nitro-*N*-(2-nitro-4-(trifluoromethyl)phenyl)aniline (**3b**) S33
  - HSQC spectra of (**3b**) S37
  - HMBC spectra of (**3b**) S38
- 5-ethoxy-4-isobutoxy-2-nitro-*N*-(2-nitro-4-(trifluoromethyl)phenyl)aniline (**3c**) S40
- 4-butoxy-5-isobutoxy-2-nitro-*N*-(2-nitro-4-(trifluoromethyl)phenyl)aniline (**3d**) S44
- 4-(hexyloxy)-5-isobutoxy-2-nitro-*N*-(2-nitro-4-(trifluoromethyl)phenyl)aniline (**3e**) S48
- 5-isobutoxy-2-nitro-*N*-(2-nitro-4-(trifluoromethyl)phenyl)-4-(octyloxy)aniline (**3f**) S52
- 4-(decyloxy)-5-isobutoxy-2-nitro-*N*-(2-nitro-4-(trifluoromethyl)phenyl)aniline (**3g**) S56
- *N*-(4,5-bis(hexyloxy)-2-nitrophenyl)-4,5-dimethoxy-2-nitroaniline (**4**) S60

^{1}H, ^{13}C, ^{19}F NMR, IR and HRMS spectra of phenazine derivatives S64
- 7-(*tert*-butyl)-3-isobutoxy-2-methoxyphenazine (**5a**) S64
- 7-(*tert*-butyl)-2-ethoxy-3-isobutoxyphenazine (**5b**) S67
- 7-(*tert*-butyl)-3-ethoxy-2-isobutoxyphenazine (**5c**) S71
  - Comparison of IR spectra of compounds **5b** and **5c** S75

2-butoxy-7-(*tert*-butyl)-3-isobutoxyphenazine (**5d**) S76
7-(*tert*-butyl)-2-(hexyloxy)-3-isobutoxyphenazine (**5e**) S79
7-(*tert*-butyl)-3-isobutoxy-2-(octyloxy)phenazine (**5f**) S82
7-(*tert*-butyl)-2-(decyloxy)-3-isobutoxyphenazine (**5g**) S85
2-isobutoxy-3-methoxy-7-(trifluoromethyl)phenazine (**6a**) S88
3-ethoxy-2-isobutoxy-7-(trifluoromethyl)phenazine (**6b**) S92
HSQC spectra of (**6b**) S96
HMBC spectra of (**6b**) S97
2-ethoxy-3-isobutoxy-7-(trifluoromethyl)phenazine (**6c**) S99
Comparison of IR spectra of compounds **6b** and **6c** S103
3-butoxy-2-isobutoxy-7-(trifluoromethyl)phenazine (**6d**) S104
3-(hexyloxy)-2-isobutoxy-7-(trifluoromethyl)phenazine (**6e**) S108
2-isobutoxy-3-(octyloxy)-7-(trifluoromethyl)phenazine (**6f**) S112
3-(decyloxy)-2-isobutoxy-7-(trifluoromethyl)phenazine (**6g**) S116
2,3-bis(hexyloxy)-7,8-dimethoxyphenazine (**7**) S120

Crystallographic data for 5c hydrate, 6b and 6b solvate S123

Table S1 .... S123
Table S2 .... S125
Table S3 .... S128
Figures for **5c hydrate** S130
Figures for pure **6b** and **6b solvate** S133

On NMR spectra signals form solvents and contaminations are marked as following:

Chloroform (residual in chloroform-d on ^{1}H NMR spectra) – ▼

DCM – ◆

Grease – ●

Hexanes – ■

Water – ✚

Perfluorobenzene – ⬇

Acetone – ⬆

4-bromo-1,2-dimethoxybenzene.

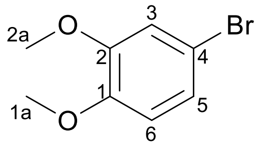

4-bromo-1,2-dimethoxybenzene

^{1}H NMR ($CDCl_3$, 300 MHz, δ ppm): 7.04 (dd, $^3J_{H5–H6}$ = 8.54Hz, $^4J_{H5–H3}$ = 2.32Hz, 1H, H_5), 6.99 (d, $^4J_{H5–H3}$ = 2.32 Hz, 1H, H_3), 6.74 (d, $^3J_{H5–H6}$ = 8.64 Hz, 1H, H_6), 3.87 (s, 3H, H_{2a}), 3.86 (s, 3H, H_{1a}).

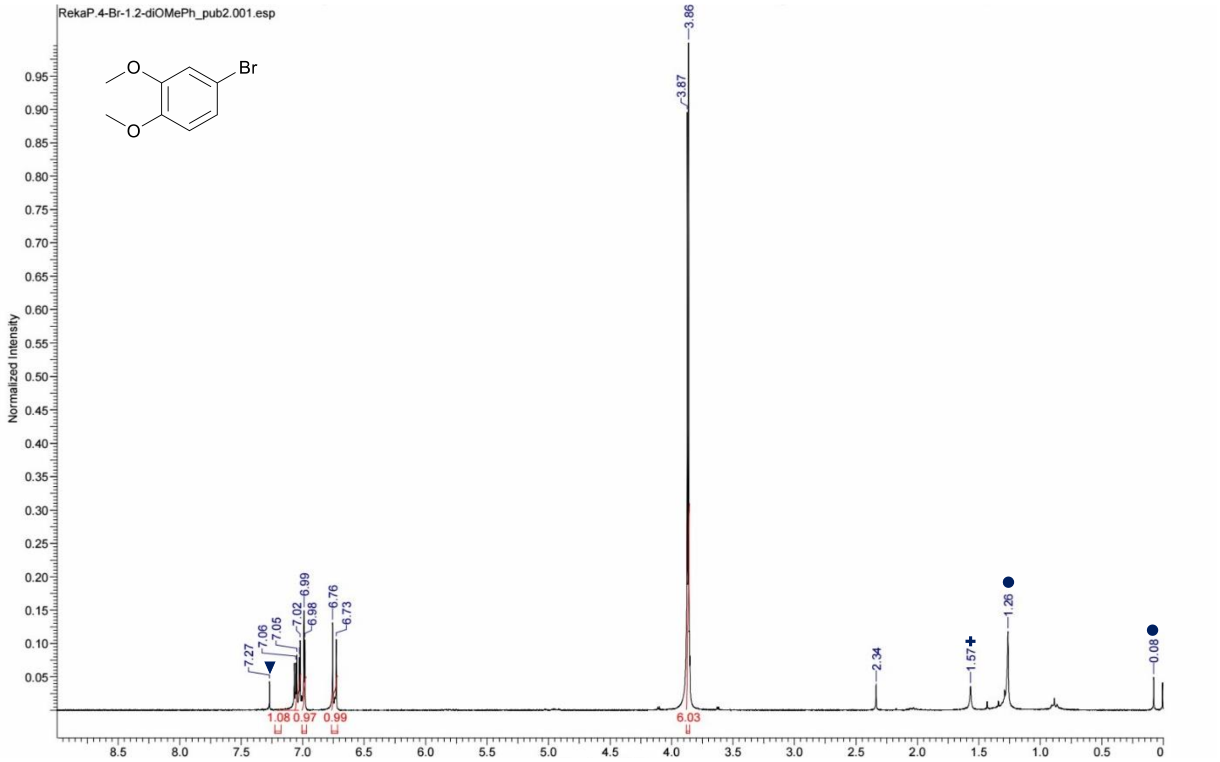

Figure S1. ^{1}H NMR ($CDCl_3$, 300 MHz, δ ppm) spectrum of **4-bromo-1,2-dimethoxybenzene**.

1-bromo-4,5-dimethoxy-2-nitrobenzene.

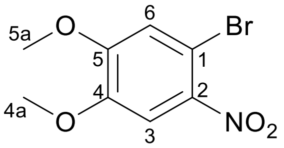

1-bromo-4,5-dimethoxy-2-nitrobenzene

^{1}H NMR ($CDCl_3$, 300 MHz, δ ppm): 7.57 (s, 1H, H_3), 7.12 (s, 1H, H_6), 3.97 (s, 3H, H_{5a}), 3.94 (s, 3H, H_{4a}).

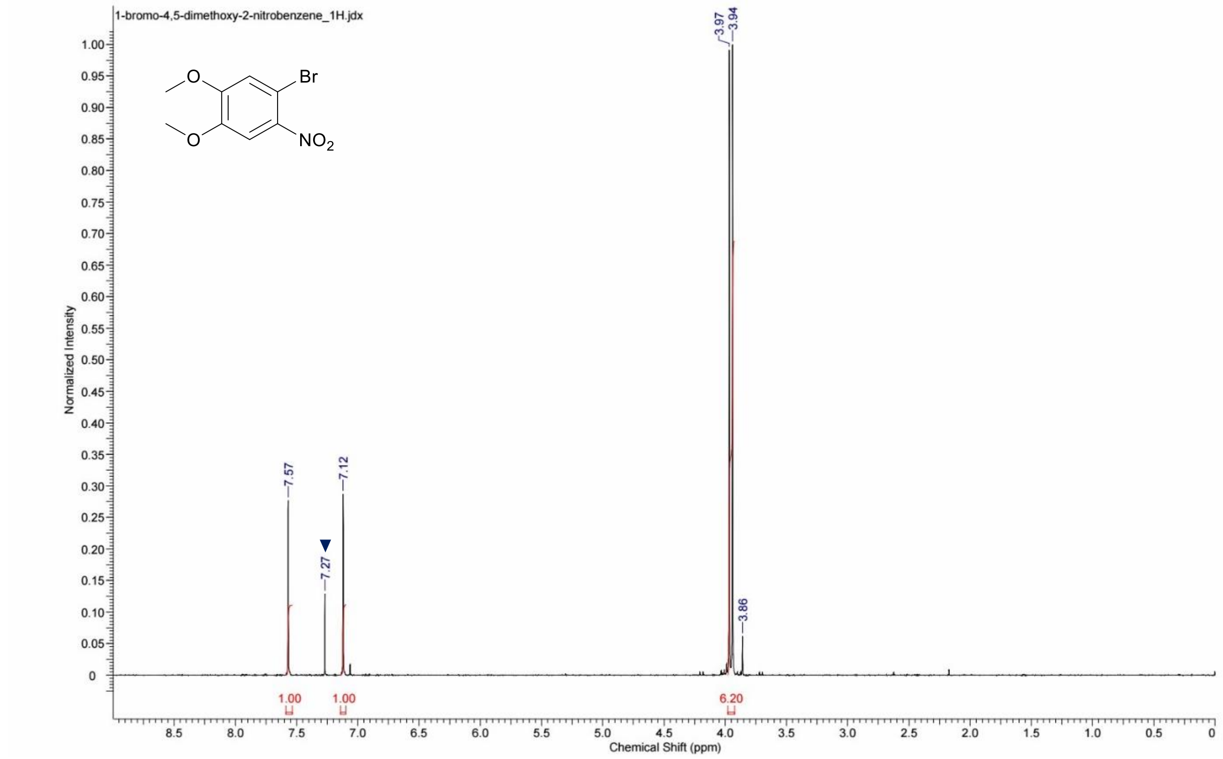

Figure S2. ^{1}H NMR ($CDCl_3$, 300 MHz, δ ppm) spectrum of **1-bromo-4,5-dimethoxy-2-nitrobenzene**.

***N*-(5-(tert-butyl)-2-nitrophenyl)-5-isobutoxy-4-methoxy-2-nitroaniline (2a).**

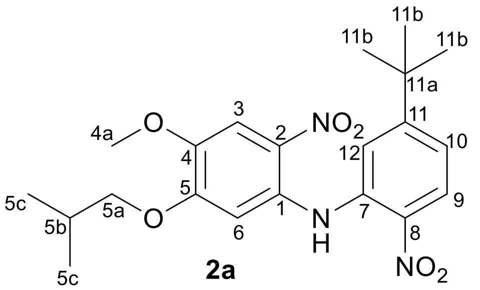

^{1}H NMR ($CDCl_3$, 300 MHz, δ ppm): 11.18 (s, 1H, H_{N-H}), 8.15 (d, $^{3}J_{H9-H10}$ = 8.89 Hz, 1H, H_9), 7.71 (s, 1H, H_3), 7.59 (d, $^{4}J_{H12-H10}$ = 2.00 Hz, 1H, H_{12}), 7.10 (dd, $^{3}J_{H9-H10}$ = 8.89 Hz, $^{4}J_{H10-H12}$ = 2.00Hz, 1H, H_{10}), 6.97 (s, 1H, H_6), 3.93 (s, 3H, H_{4a}), 3.70 (d, $^{3}J_{H5a-H5b}$ = 6.75 Hz, 2H, H_{5a}), 2.25 – 2.11 (m, 1H, H_{5b}), 1.32 (s, 9H, H_{11b}), 1.01 (d, $^{3}J_{H5b-H5c}$ = 6.75 Hz, 6H, H_{5c}).

$^{13}C\{^{1}H\}$ NMR ($CDCl_3$, 75 MHz, δ ppm): 159.9 (C_8), 155.8 (C_5), 144.9 (C_4), 137.8 (C_7), 137.0 (C_{11}), 134.5 (C_2), 131.1 (C_1), 127.3 (C_9), 119.8 (C_{10}), 117.4 (C_{12}), 108.9 (C_3), 102.4 (C_6), 76.5 (C_{5a}), 57.2 (C_{4a}), 36.2 (C_{11a}), 31.5 (C_{11b}), 28.7 (C_{5b}), 19.8 (C_{5c}).

FT-IR (ATR, ν_{max}, (neat)/cm^{-1}): 3270, 2961, 2932, 2899, 2871, 1605, 1582, 1515, 1487, 1468, 1441, 1318, 1274, 1250, 1208, 1194, 1085, 1066, 1025, 999, 992, 850, 837.

HRMS (ESI) *m/z* Calculated for $C_{21}H_{27}N_3O_6Na$ $[M+Na]^+$, 440.1793; found: 440.1795.

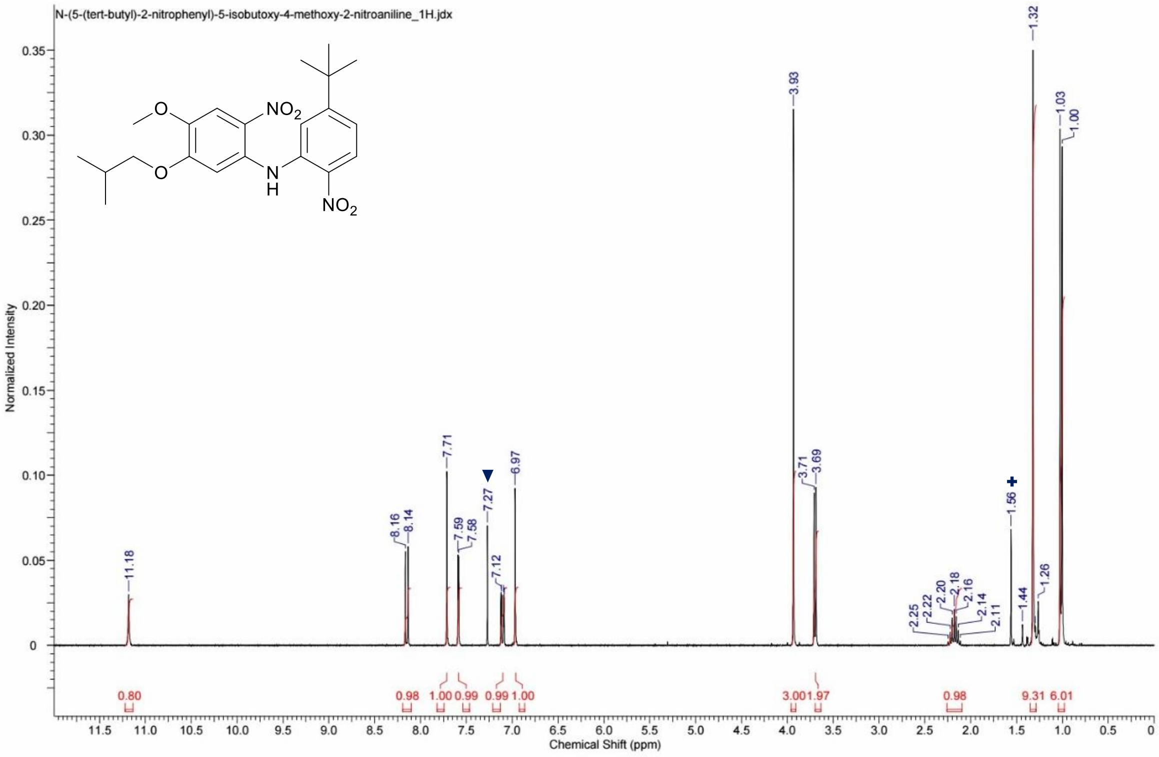

Figure S3. ^{1}H ($CDCl_3$, 300 MHz, δ ppm) NMR spectrum of **2a**.

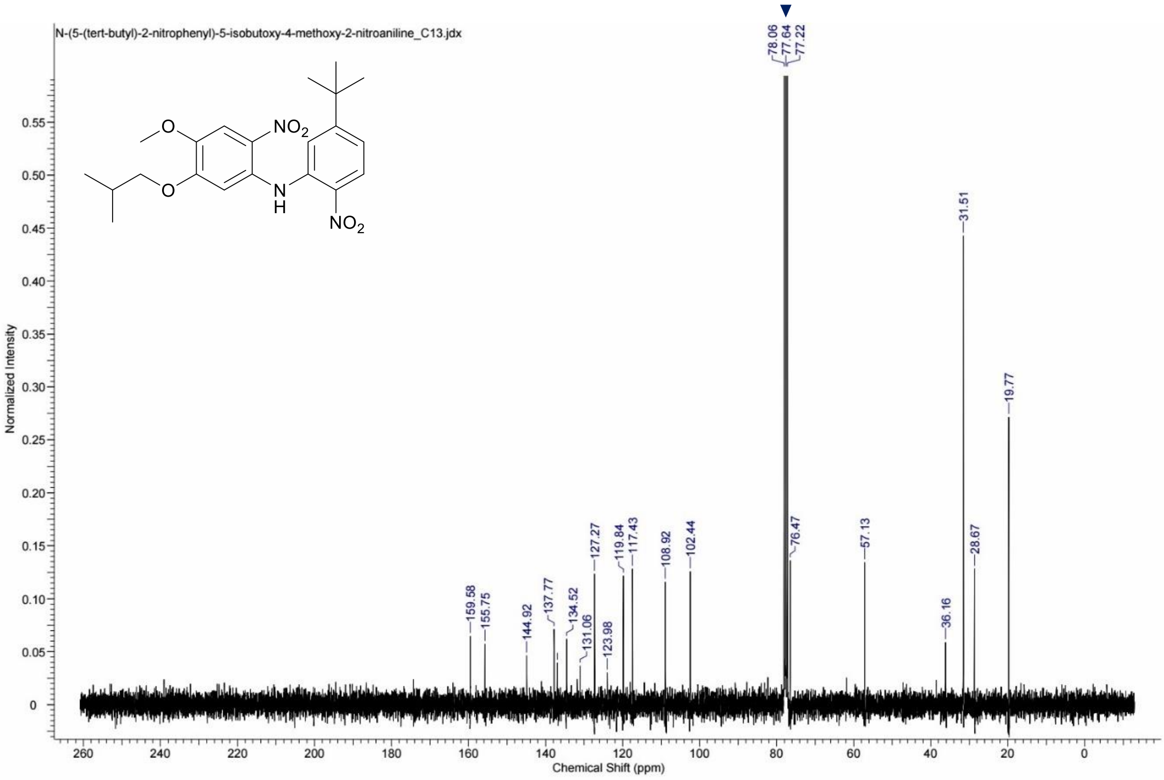

Figure S4. $^{13}C\{^{1}H\}$ NMR ($CDCl_3$, 75 MHz, δ ppm) NMR spectrum of **2a**.

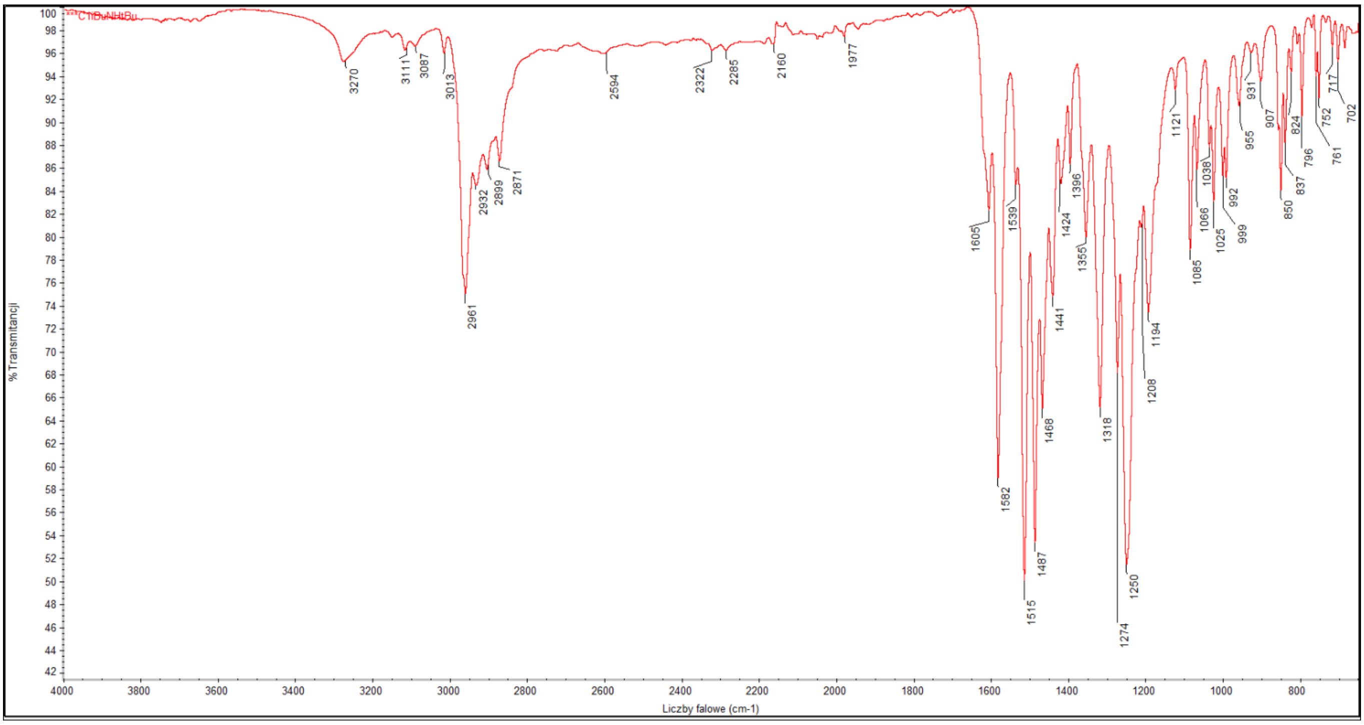

Figure S5. IR spectrum of **2a**.

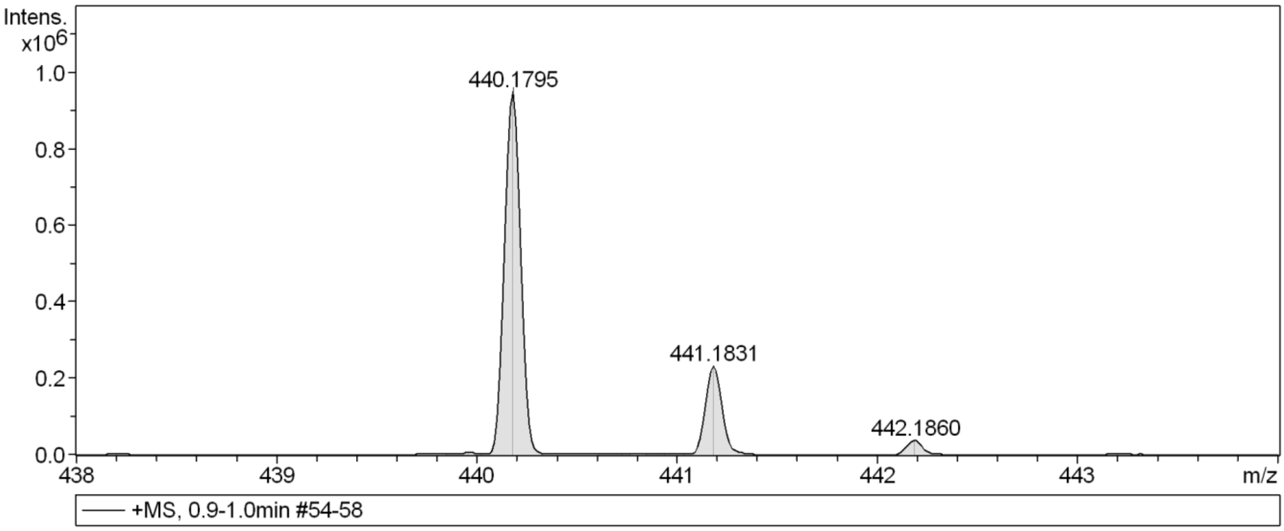

Figure S6. HRMS (ESI) spectrum of **2a**.

N-(5-(tert-butyl)-2-nitrophenyl)-4-ethoxy-5-isobutoxy-2-nitroaniline (2b).

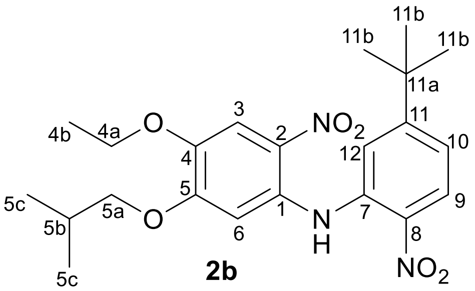

^{1}H NMR ($CDCl_3$, 300 MHz, δ ppm): 11.17 (s, 1H, $H_{N–H}$), 8.14 (d, $^3J_{H9–H10}$ = 8.91 Hz, 1H, H_9), 7.71 (s, 1H, H_3), 7.59 (d, $^4J_{H10–H12}$ = 1.90 Hz, 1H, H_{12}), 7.09 (dd, $^3J_{H9–H10}$ = 8.91 Hz, $^4J_{H10–H12}$ = 1.90 Hz, 1H, H_{10}), 6.96 (s, 1H, H_6), 4.03 (q, $^3J_{H4a–H4b}$ = 6.93 Hz, 2H, H_{4a}), 3.69 (d, $^3J_{H5a–H5b}$ = 6.71 Hz, 2H, H_{5a}), 2.24 – 2.10 (m, 1H, H_{5b}), 1.48 (t, $^3J_{H4a–H4b}$ = 7.05 Hz 3H, H_{4b}), 1.32 (s, 9H, H_{11b}), 1.02 (d, $^3J_{H5b–H5c}$ = 6.62 Hz, 6H, H_{5c}).

^{13}C{^{1}H} NMR ($CDCl_3$, 75 MHz, δ ppm): 159.6 (C_8), 156.1 (C_5), 144.2 (C_4), 137.9 (C_7), 136.8 (C_{11}), 134.4 (C_2), 131.4 (C_1), 127.3 (C_9), 119.8 (C_{10}), 117.4 (C_{12}), 110.5 (C_3), 102.6 (C_6), 76.4 (C_{5a}), 65.9 (C_{4a}), 36.1 (C_{11a}), 31.5 (C_{11b}), 28.7 (C_{5b}), 19.7 (C_{5c}), 15.2 (C_{4b}).

FT-IR (ATR, ν_{max}, (neat)/cm^{-1}): 3314, 2974, 2930, 2871, 1610, 1579, 1514, 1485, 1467, 1435, 1417, 1397, 1350, 1319, 1252, 1208, 1197, 1082, 1064, 1045, 1014, 646, 872, 848, 818, 803, 758.

HRMS (ESI) *m/z* Calculated for $C_{22}H_{29}N_3O_6Na$ [M+Na]$^+$, 454.1949; found: 454.1949.

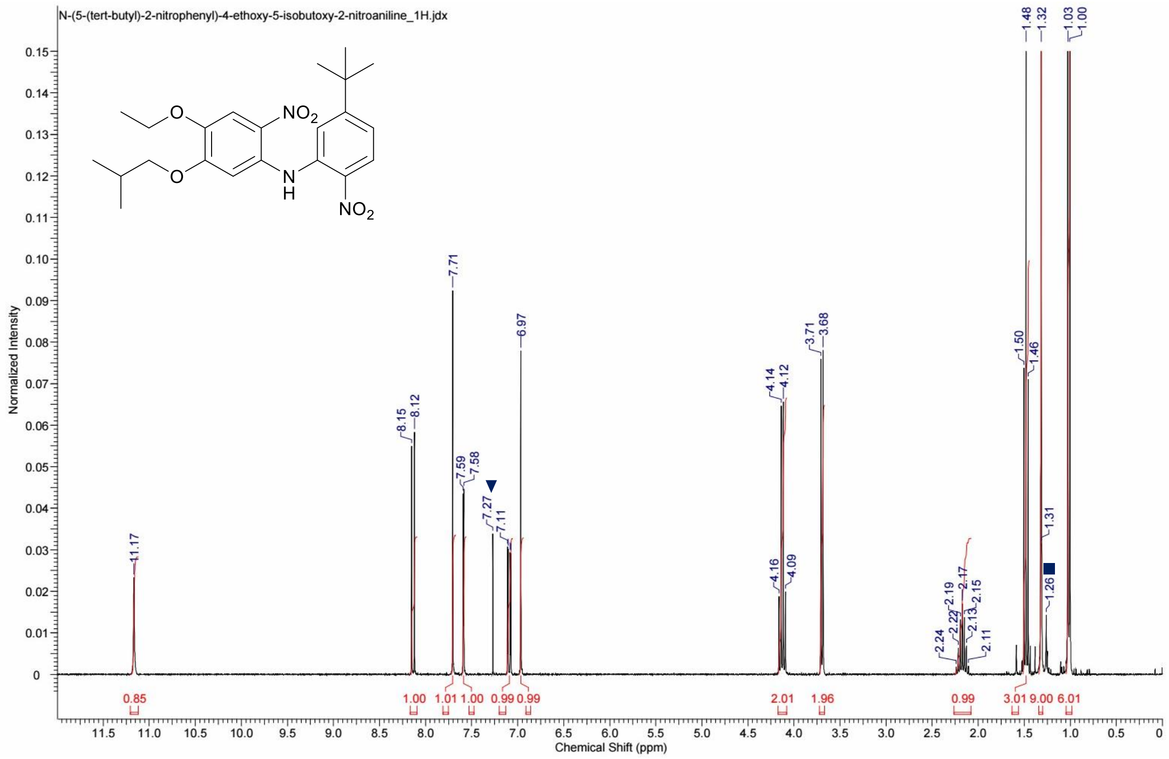

Figure S7. ^{1}H ($CDCl_3$, 300 MHz, δ ppm) NMR spectrum of **2b**.

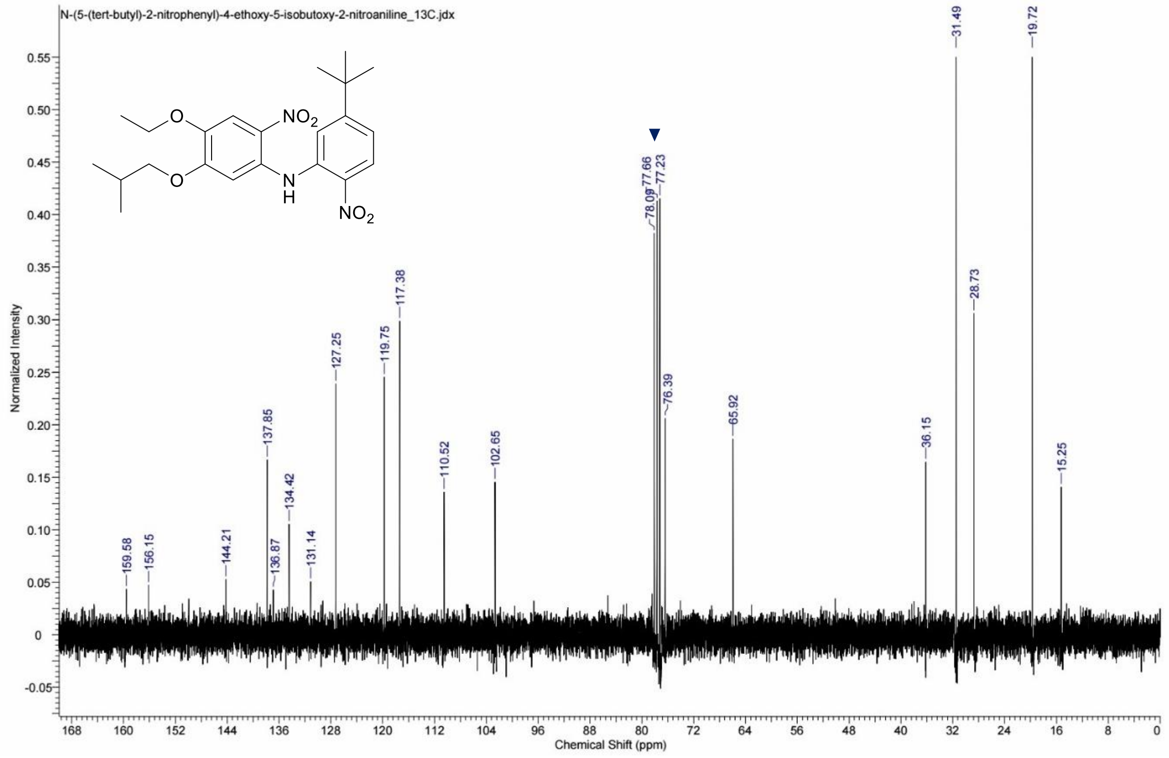

Figure S8. $^{13}C\{^{1}H\}$ NMR ($CDCl_3$, 75 MHz, δ ppm) NMR spectrum of **2b**.

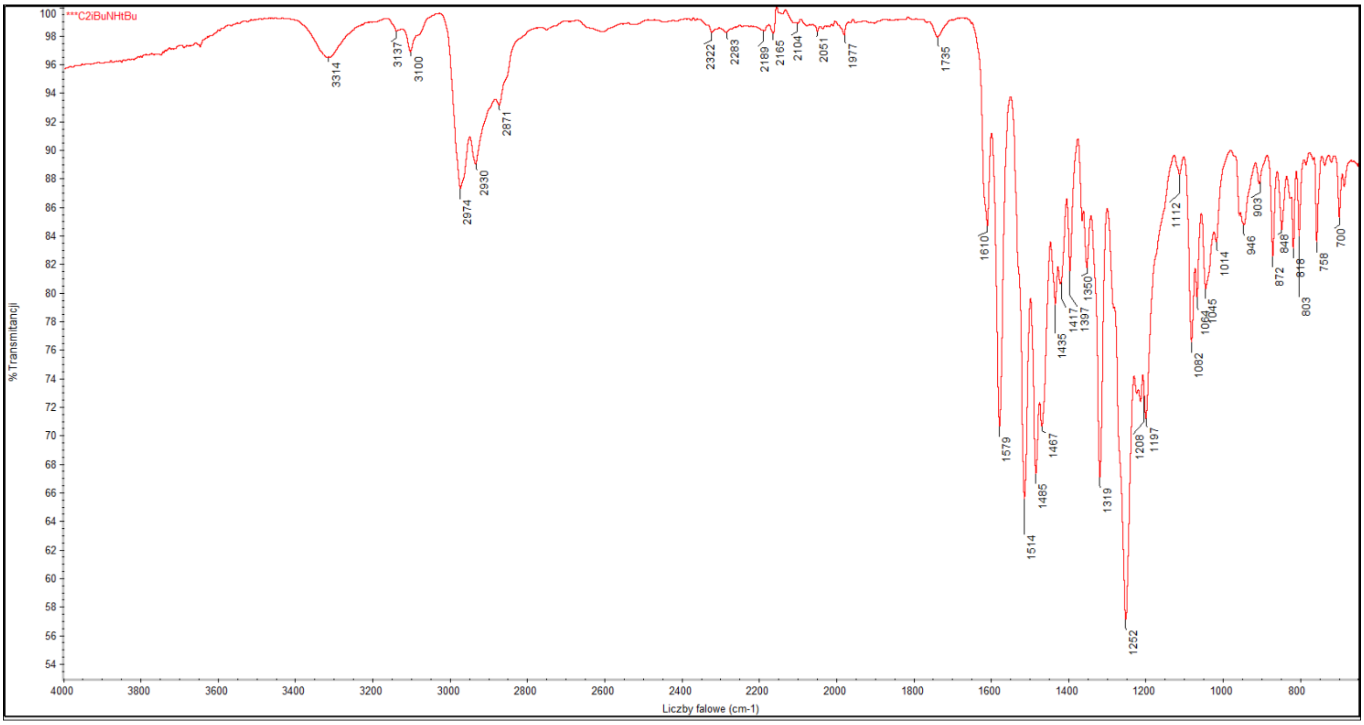

Figure S9. IR spectrum of **2b**.

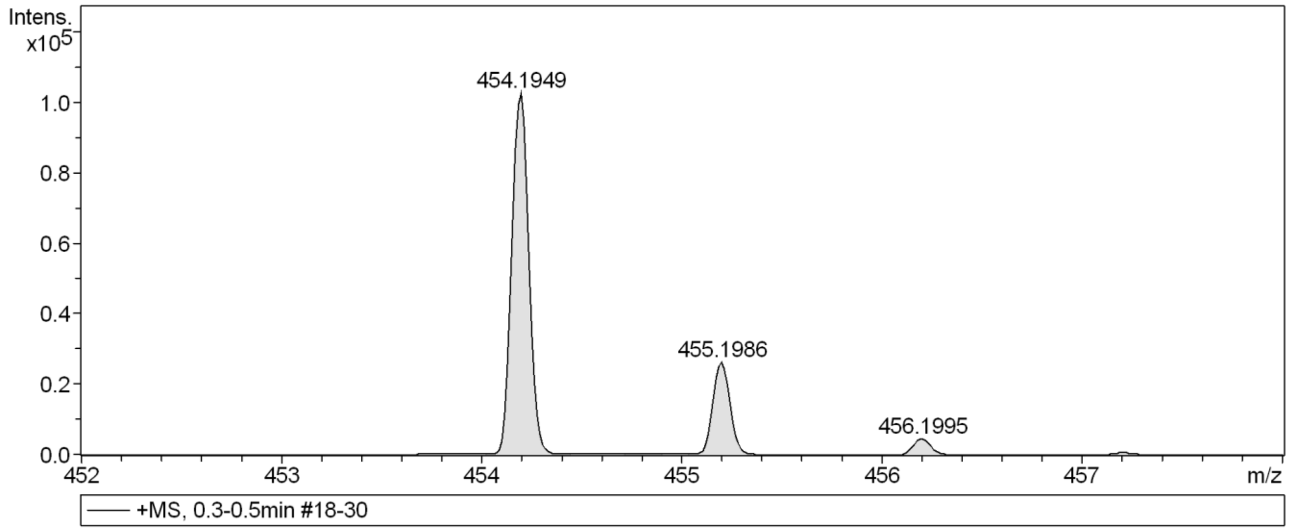

Figure S10. HRMS (ESI) spectrum of **2b.**

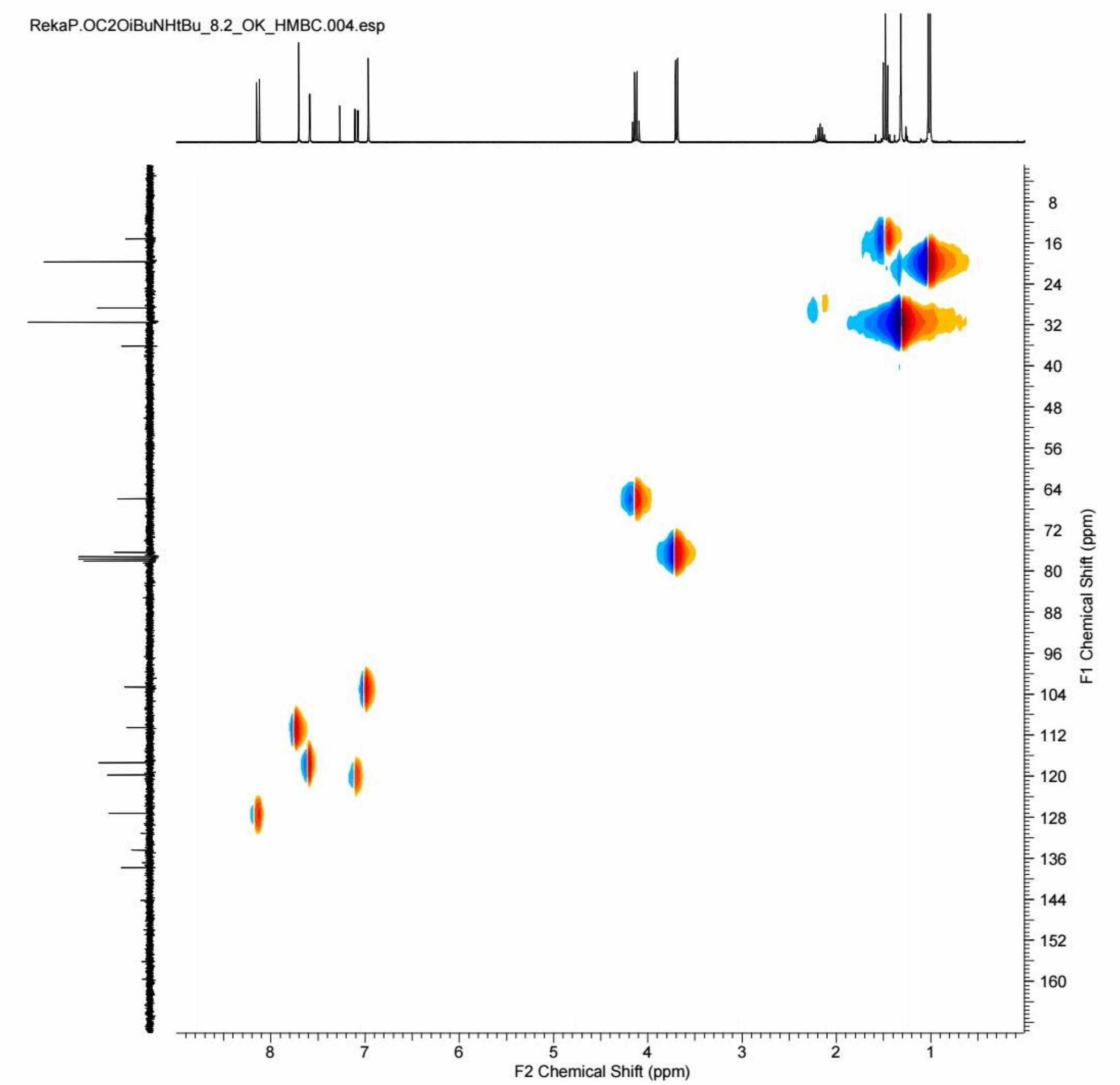

Figure S11. HSQC spectrum of **2b**.

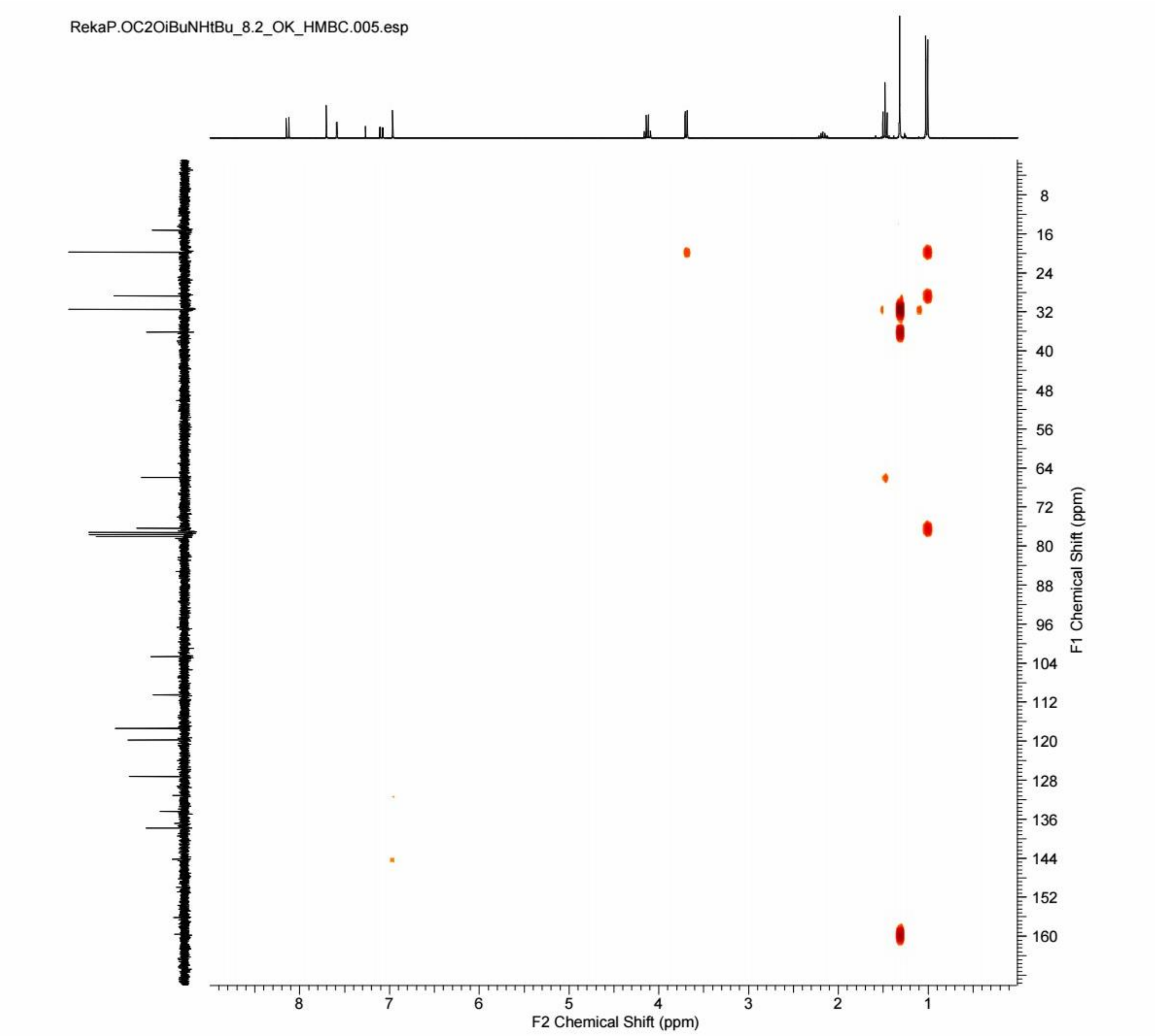

Figure S12. HMBC spectrum of **2b**.

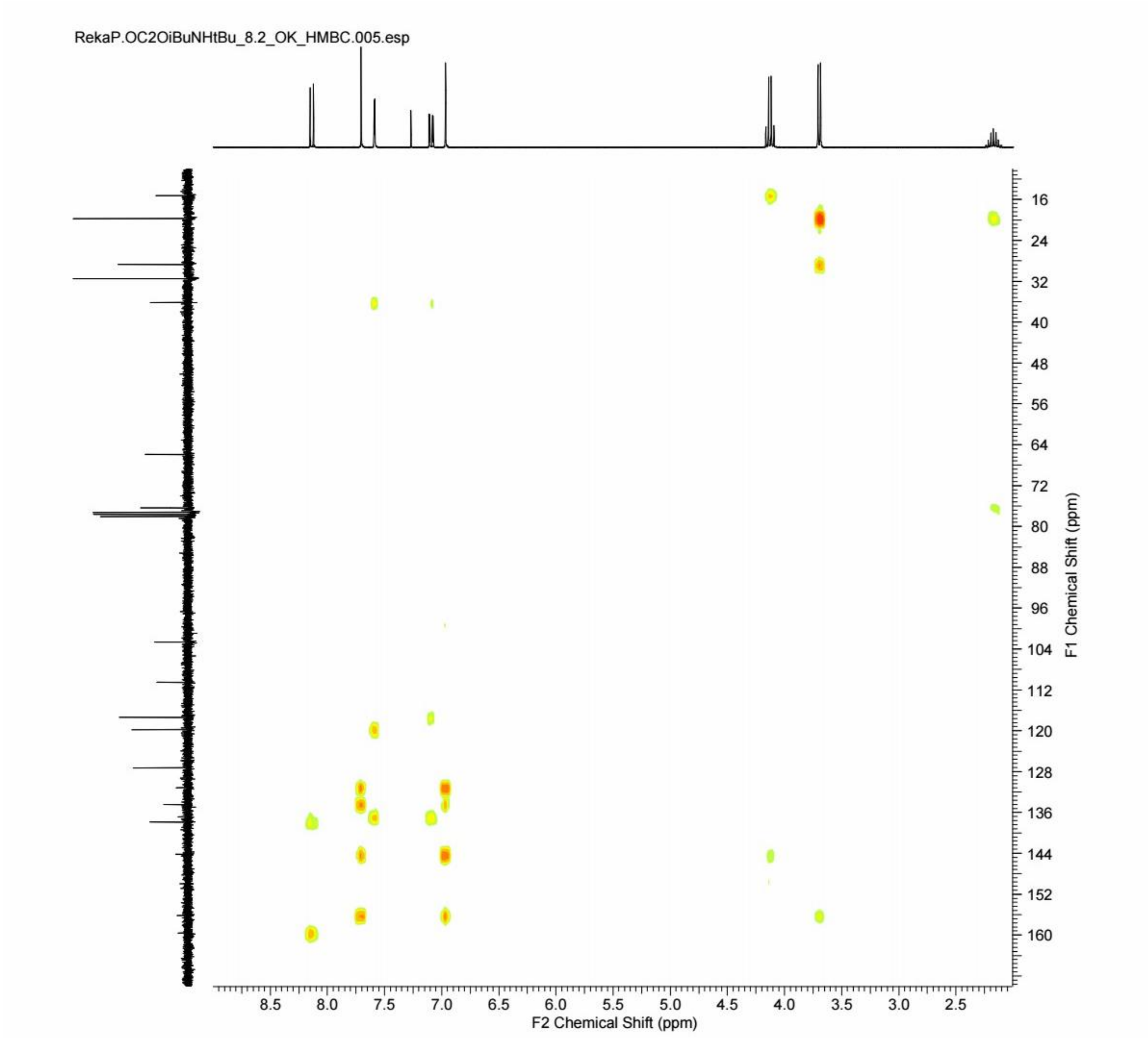

Figure S13. Zoomed region of HMBC spectrum of **2b**.

_N_-(5-(tert-butyl)-2-nitrophenyl)-5-ethoxy-4-isobutoxy-2-nitroaniline (2c).

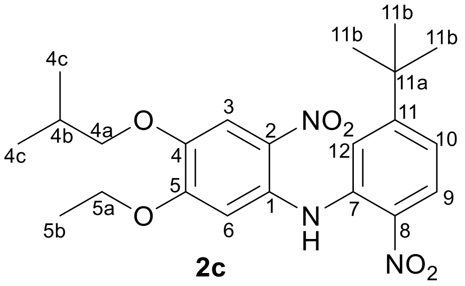

2c

^{1}H NMR ($CDCl_3$, 300 MHz, δ ppm): 11.12 (s, 1H, $H_{N–H}$), 8.14 (d, $^3J_{H9–H10}$ = 8.92 Hz, 1H, H_9), 7.69 (s, 1H, H_3), 7.56 (d, $^4J_{H10–H12}$ = 2.04 Hz, 1H, H_{12}), 7.09 (dd, $^3J_{H9–H10}$ = 8.92 Hz, $^4J_{H10–H12}$ = 2.04 Hz, 1H, H_{10}), 6.94 (s, 1H, H_6), 4.02 (q, $^3J_{H5a–H5b}$ = 6.98 Hz, 2H, H_{5a}), 3.81 (d, $^3J_{H4a–H4b}$ = 6.64 Hz, 2H, H_{4a}), 2.25 – 2.11 (m, 1H, H_{4b}), 1.46 (t, $^3J_{H5a–H5b}$ = 7.00 Hz 3H, H_{5b}), 1.31 (s, 9H, H_{11b}), 1.07 (d, $^3J_{H4b–H4c}$ = 6.71 Hz, 6H, H_{4c}).

^{13}C{^{1}H} NMR ($CDCl_3$, 75 MHz, δ ppm): 159.7 (C_8), 155.9 (C_5), 144.5 (C_4), 138.0 (C_7), 136.8 (C_{11}), 134.3 (C_2), 131.4 (C_1), 127.2 (C_9), 119.8 (C_{10}), 117.2 (C_{12}), 110.3 (C_3), 102.8 (C_6), 76.6 (C_{4a}), 65.6 (C_{5a}), 36.1 (C_{11a}), 31.5 (C_{11b}), 28.9 (C_{4b}), 19.9 (C_{4c}), 15.2 (C_{5b}).

FT-IR (ATR, ν_{max}, (neat)/cm^{-1}): 3307, 3107, 2959, 2928, 2904, 2871, 1611, 1579, 1533, 1513, 1487, 1468, 1436, 1413, 1395, 1365, 1352, 1319, 1274, 1250, 1201, 1177, 1083, 1068, 1040, 1021, 960, 926, 887, 867, 849, 819, 806, 756, 700.

HRMS (ESI) *m/z* Calculated for $C_{22}H_{29}N_3O_6Na$ $[M+Na]^+$, 454.1949; found: 454.1951.

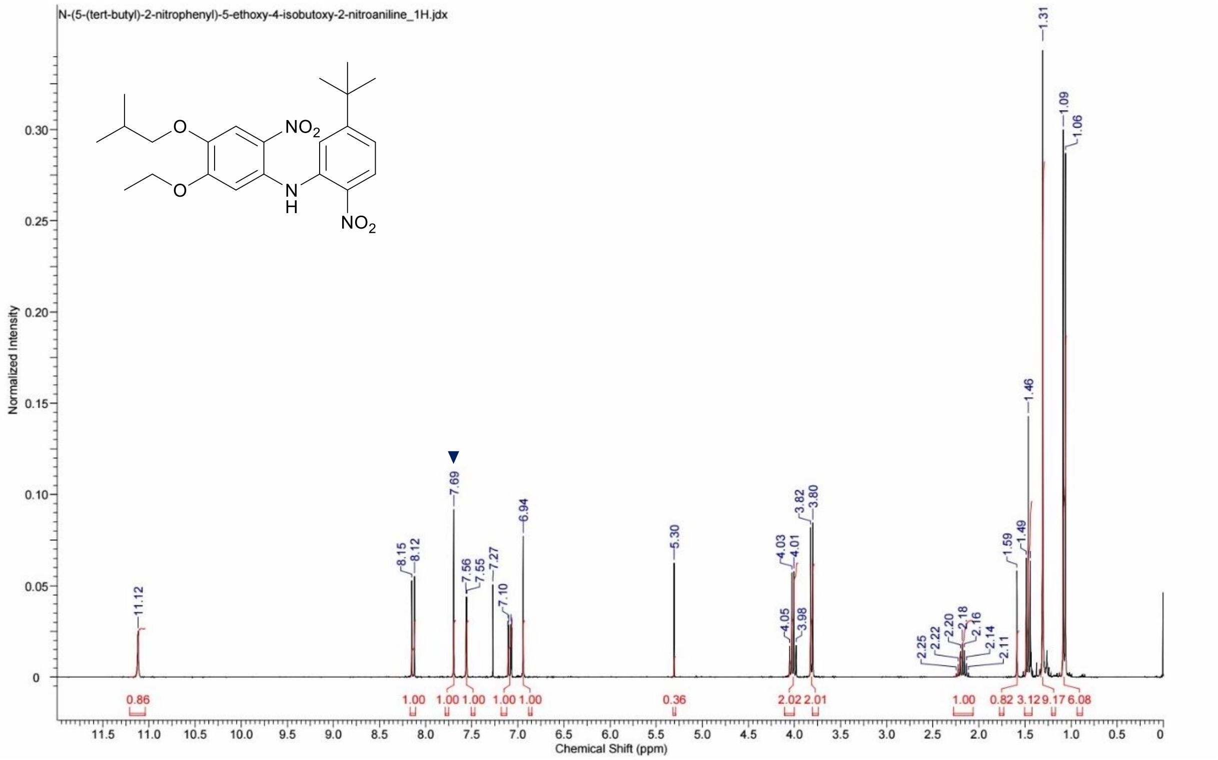

Figure S14. ^{1}H NMR ($CDCl_3$, 300 MHz, δ ppm) spectrum of **2c**.

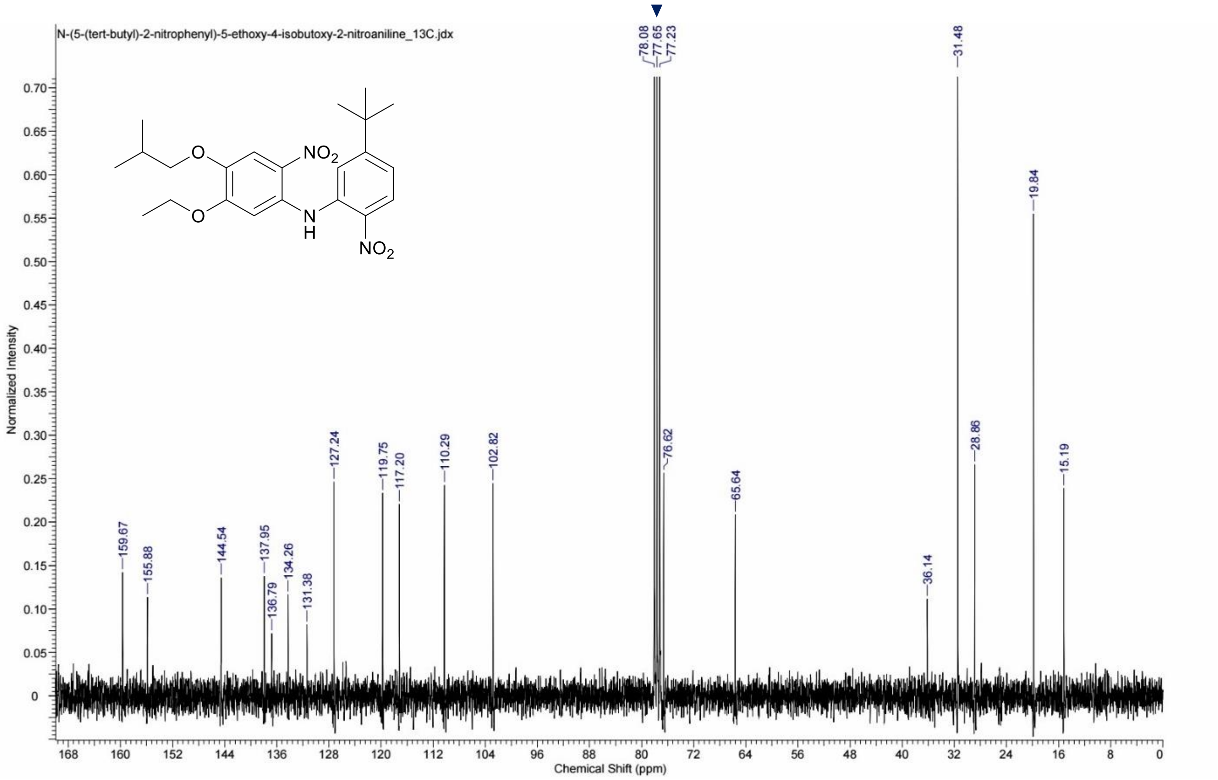

Figure S15. $^{13}C\{^1H\}$ NMR ($CDCl_3$, 75 MHz, δ ppm) NMR spectrum of **2c**.

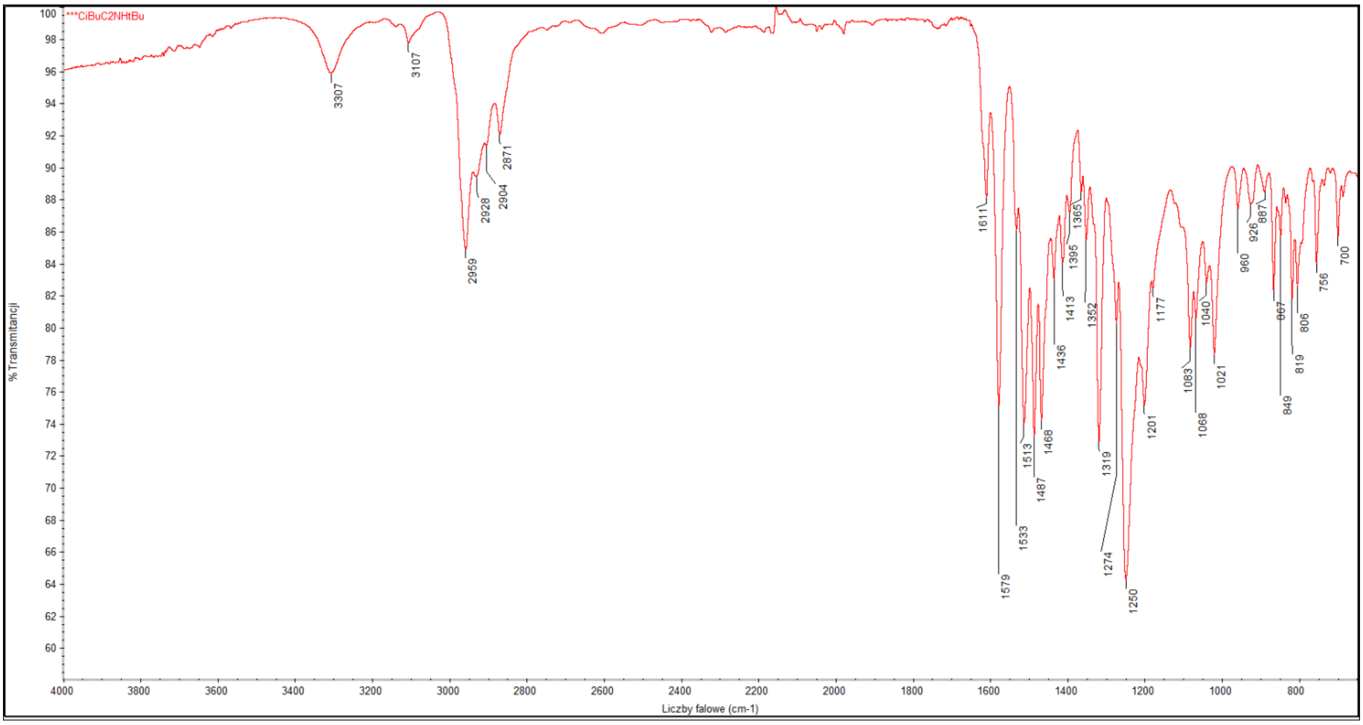

Figure S16. IR spectrum of **2c**.

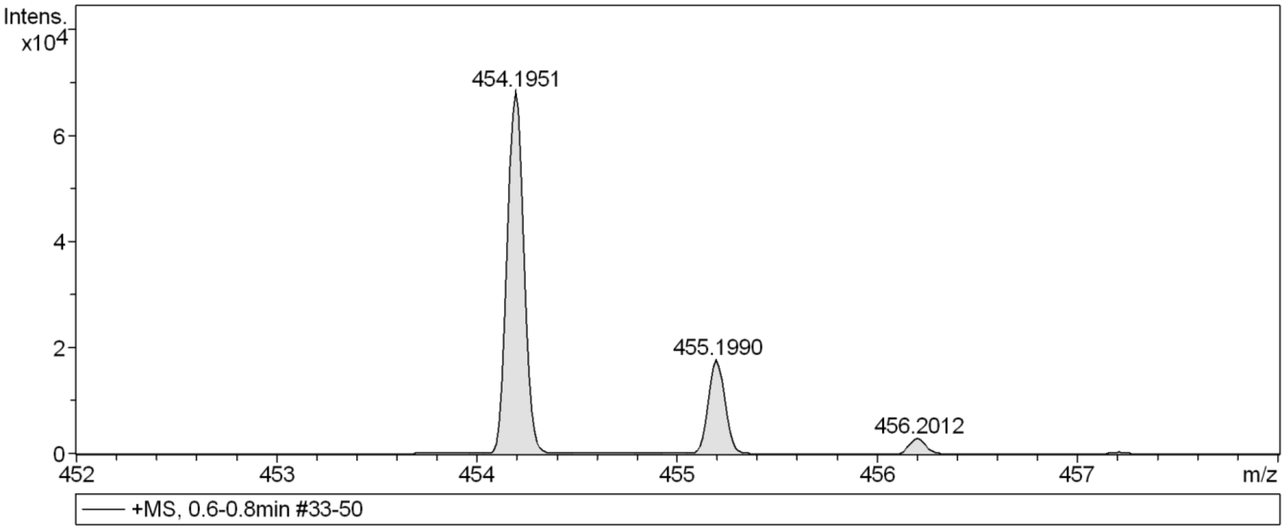

Figure S17. HRMS (ESI) spectrum of **2c**.

4-butoxy-*N*-(5-(tert-butyl)-2-nitrophenyl)-5-isobutoxy-2-nitroaniline (2d).

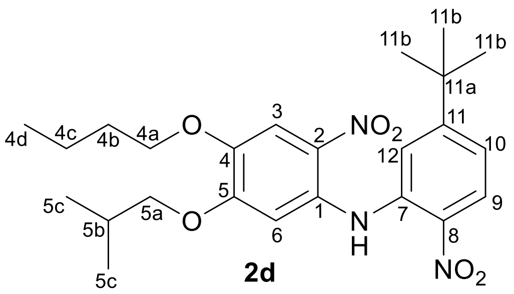

^{1}H NMR ($CDCl_3$, 300 MHz, δ ppm): 11.18 (s, 1H, $H_{N–H}$), 8.15 (d, $^3J_{H9–H10}$ = 8.89 Hz, 1H, H_9), 7.71 (s, 1H, H_3), 7.71 (d, $^4J_{H10–H12}$ = 1.97 Hz, 1H, H_{12}), 7.09 (dd, $^3J_{H9–H10}$ = 8.89 Hz, $^4J_{H10–H12}$ = 1.97 Hz, 1H, H_{10}), 6.96 (s, 1H, H_6), 4.06 (t, $^3J_{H4a–H4b}$ = 6.42Hz, 2H, H_{4a}), 3.69 (d, $^3J_{H5a–H5b}$ = 6.67 Hz, 2H, H_{5a}), 2.24 – 2.09 (m, 1H, H_{5b}), 1.90 – 1.79 (m, 2H, H_{4b}), 1.62 – 1.48 (m, 2H, H_{4c}), 1.32 (s, 9H, H_{11b}), 1.02 (d, $^3J_{H5b–H5c}$ = 6.69 Hz, 6H, H_{5c}), 1.01 (t, $^3J_{H4c–H4d}$ = 7.38 Hz, 3H, H_{4d}).

^{13}C{^{1}H} NMR ($CDCl_3$, 75 MHz, δ ppm): 159.6 (C_8), 156.2 (C_5), 144.5 (C_4), 137.9 (C_7), 136.9 (C_{11}), 134.4 (C_2), 131.2 (C_1), 127.3 (C_9), 119.7 (C_{10}), 117.4 (C_{12}), 110.3 (C_3), 102.6 (C_6), 76.3 (C_{5a}), 70.0 (C_{4a}), 36.2 (C_{11a}), 31.7 (C_{4b}), 31.5 (C_{11b}), 28.8 (C_{5b}), 19.8 (C_{4c}), 19.7 (C_{5c}), 14.5 (C_{4d}).

FT-IR (ATR, ν_{max}, (neat)/cm^{-1}): 3320, 2959, 2930, 2870, 1609, 1575, 1513, 1486, 1469, 1454, 1432, 1406, 1347, 1319, 1271, 1248, 1199, 1177, 1084, 1066, 1014, 966, 953, 925, 867, 846, 826, 757, 702.

HRMS (ESI) *m/z* Calculated for $C_{24}H_{33}N_3O_6Na$ [M+Na]$^+$, 482.2262; found: 482.2265.

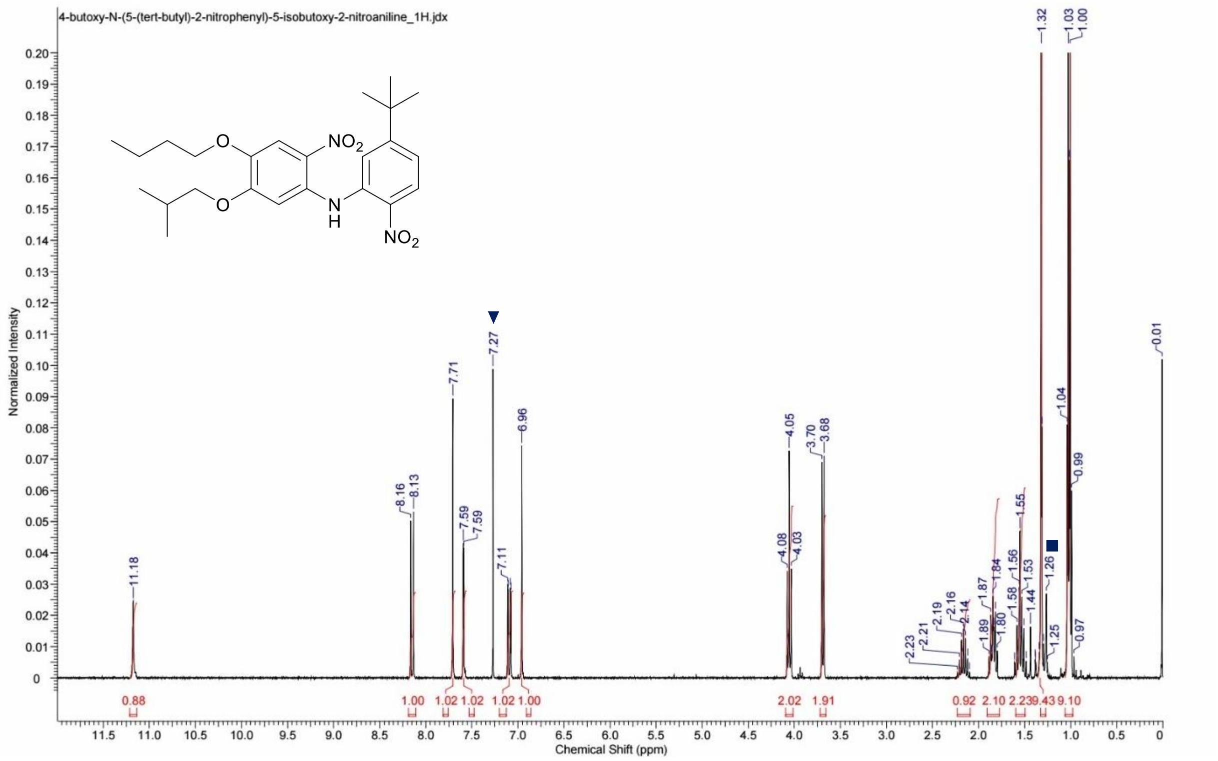

Figure S18. ^{1}H NMR ($CDCl_3$, 300 MHz, δ ppm) spectrum of **2d**.

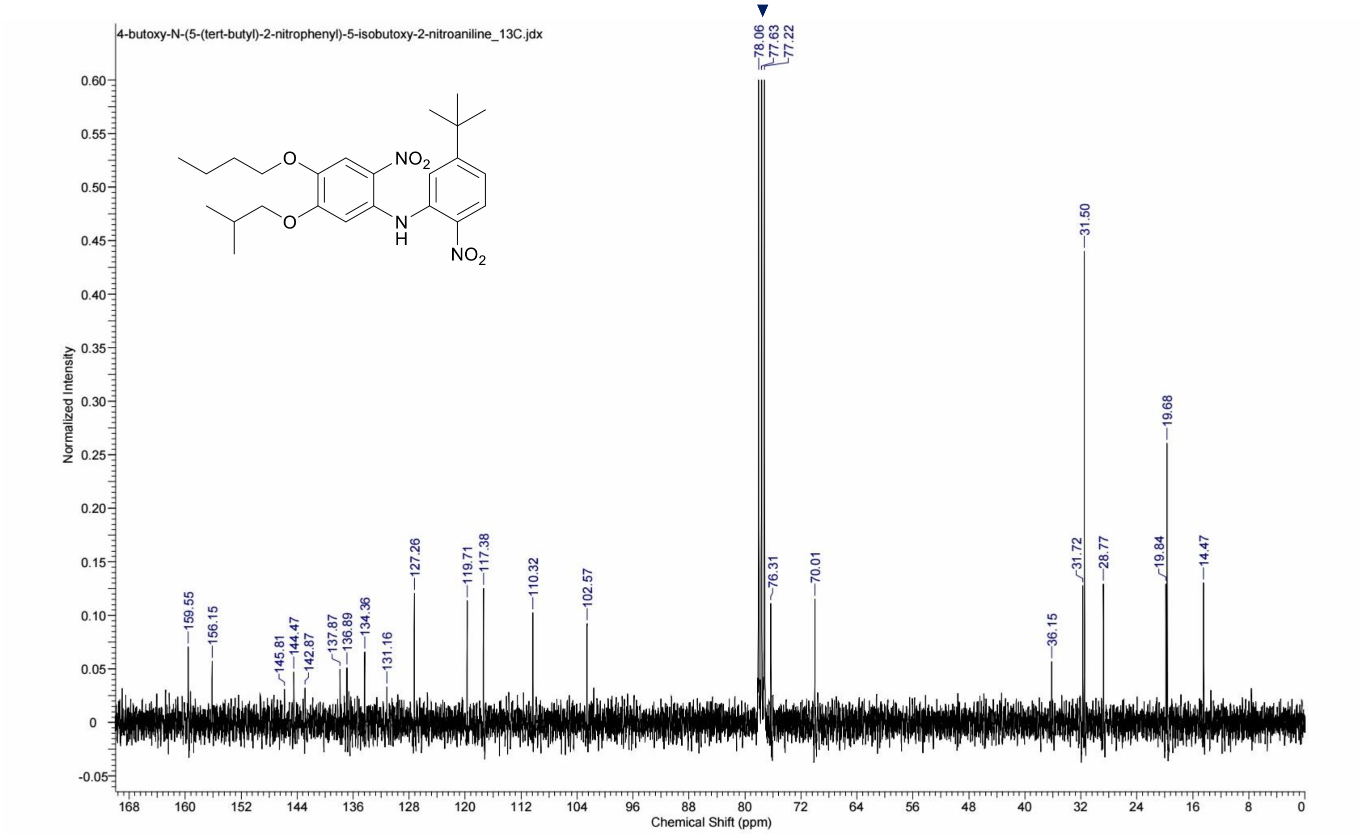

Figure S19. $^{13}C\{^{1}H\}$ NMR ($CDCl_3$, 75 MHz, δ ppm) NMR spectrum of **2d**.

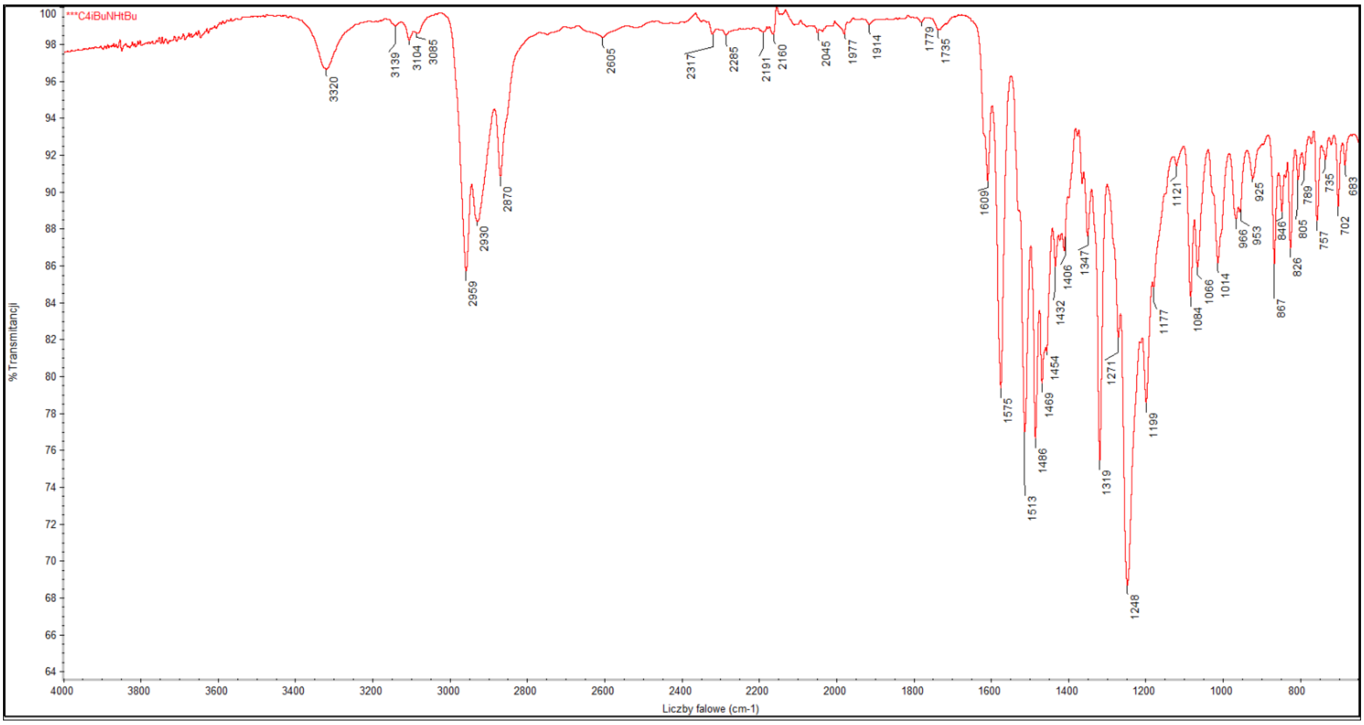

Figure S20. IR spectrum of **2d**.

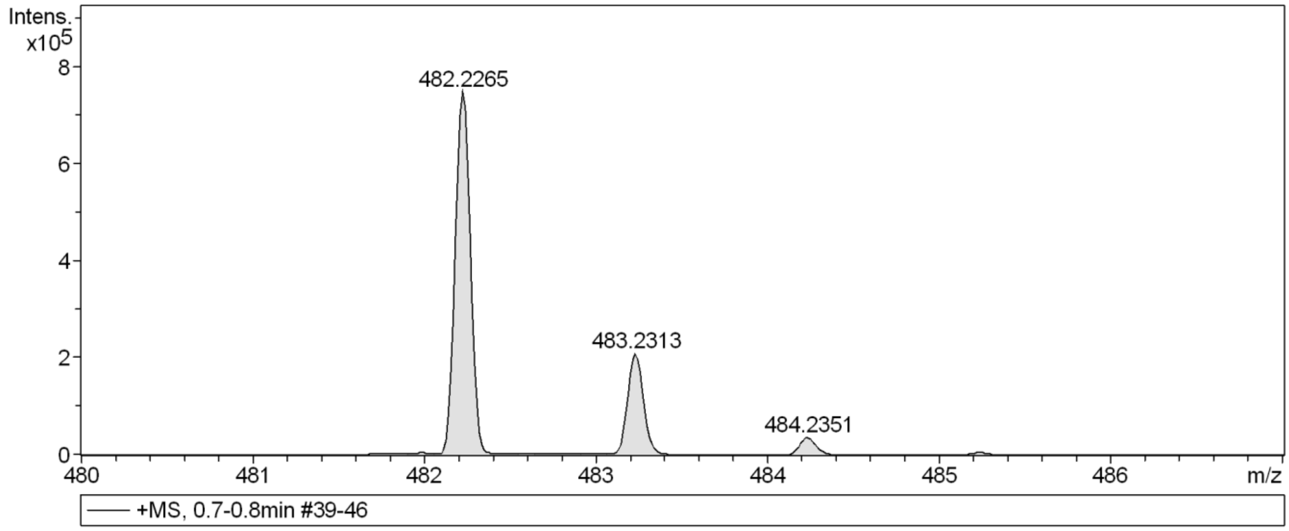

Figure S21. HRMS (ESI) spectrum of **2d**.

_N_-(5-(tert-butyl)-2-nitrophenyl)-4-(hexyloxy)-5-isobutoxy-2-nitroaniline (2e).

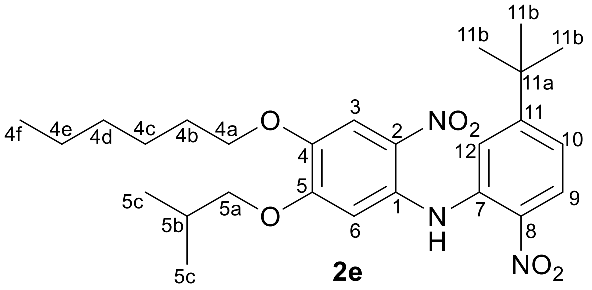

2e

^{1}H NMR ($CDCl_3$, 300 MHz, δ ppm): 11.18 (s, 1H, H_{N-H}), 8.15 (d, $^{3}J_{H9-H10}$ = 8.67 Hz, 1H, H_9), 7.70 (s, 1H, H_3), 7.59 (d, $^{4}J_{H10-H12}$ = 1.90Hz, 1H, H_{12}), 7.09 (dd, $^{3}J_{H9-H10}$ = 8.67 Hz, $^{4}J_{H10-H12}$ = 1.90 Hz, 1H, H_{10}), 6.96 (s, 1H, H_6), 4.05 (t, $^{3}J_{H4a-H4b}$ = 6.50 Hz, 2H, H_{4a}), 3.69 (d, $^{3}J_{H5a-H5b}$ = 6.50 Hz, 2H, H_{5a}), 2.23 – 2.10 (m, 1H, H_{5b}), 1.91 – 1.80 (m, 2H, H_{4b}), 1.58 – 1.45 (m, 2H, H_{4c}), 1.44 – 1.33 (m, 4H, $H_{4d,4e}$), 1.35 (s, 9H, H_{11b}), 1.02 (d, $^{3}J_{H5b-H5c}$ = 6.77 Hz, 6H, H_{5c}), 0.93 (t, $^{3}J_{H4e-H4f}$ = 7.31 Hz, 3H, H_{4f}).

^{13}C{^{1}H} NMR ($CDCl_3$, 75 MHz, δ ppm): 159.6 (C_8), 156.2 (C_5), 144.5 (C_4), 137.9 (C_7), 136.9 (C_{11}), 134.4 (C_2), 131.2 (C_1), 127.2 (C_9), 119.7 (C_{10}), 117.4 (C_{12}), 110.3 (C_3), 102.6 (C_6), 76.3 (C_{5a}), 70.3 (C_{4a}), 36.2 (C_{11a}), 32.1 (C_{4d}), 31.5 (C_{11b}), 29.6 (C_{4b}), 28.8 (C_{5b}), 26.3 (C_{4c}), 23.2 (C_{4e}), 19.7 (C_{5c}), 14.6 (C_{4f}).

FT-IR (ATR, ν_{max}, (neat)/cm^{-1}): 3276, 3106, 1956, 2925, 2871, 2855, 1744, 1623, 1610, 1581, 1515, 1488, 1470, 1438, 1421, 1396, 1351, 1337, 1323, 1251, 1197, 1083, 1072, 1043, 1021, 996, 956, 852, 825, 759, 702.

HRMS (ESI) *m/z* Calculated for $C_{26}H_{37}N_3O_6Na$ $[M+Na]^+$, 510.2575; found: 510.2575.

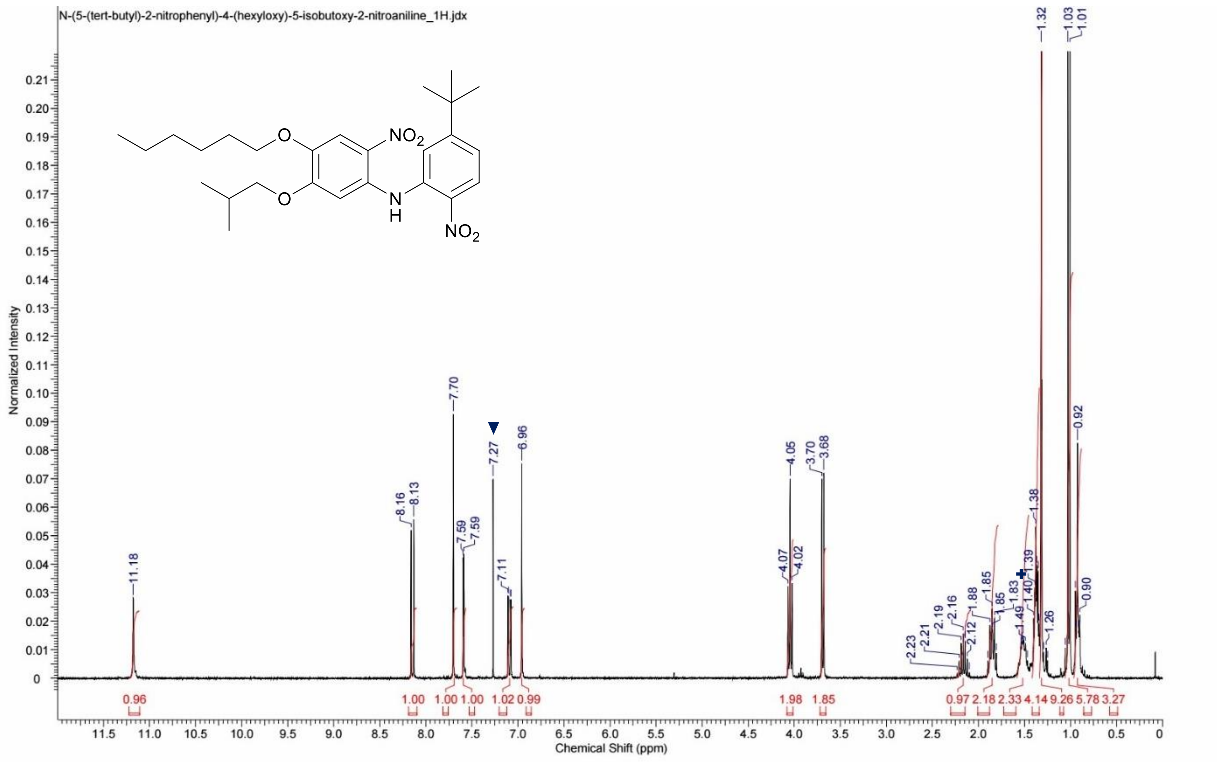

Figure S22. ^{1}H ($CDCl_3$, 300 MHz, δ ppm) NMR spectrum of **2e**.

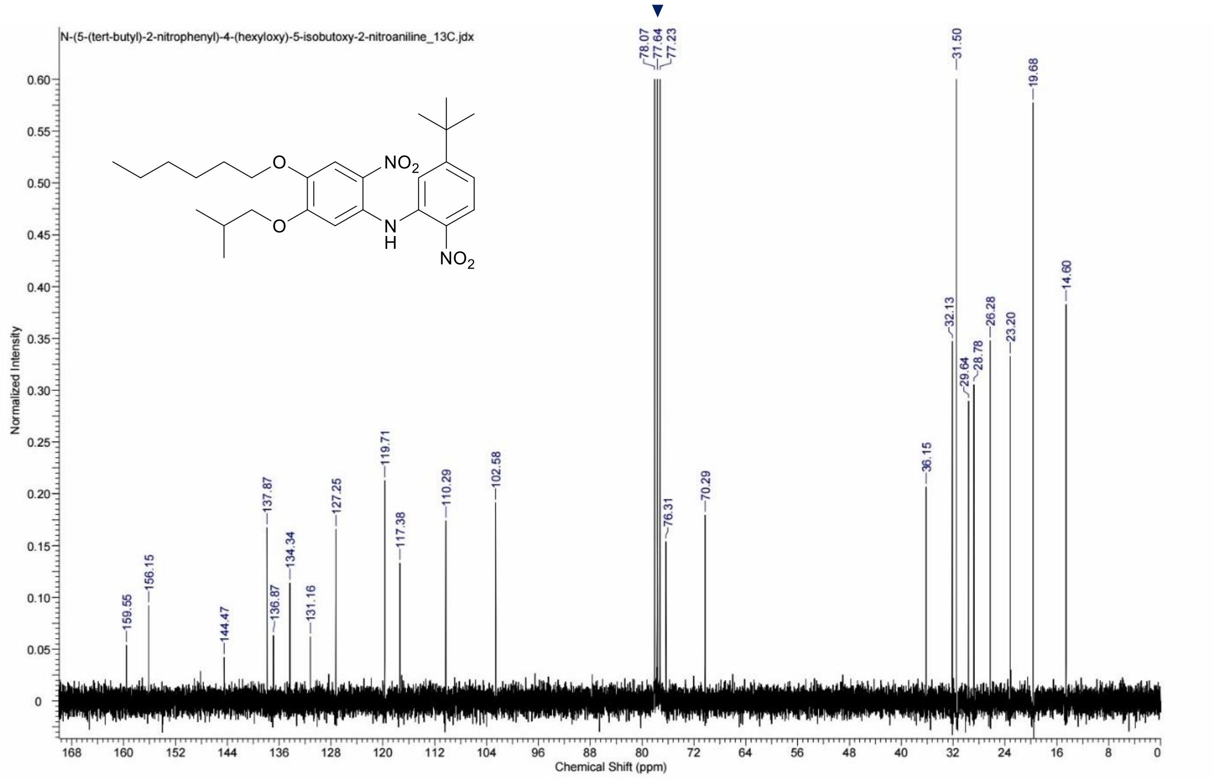

Figure S23. ^{13}C NMR{^{1}H} NMR ($CDCl_3$, 75 MHz, δ ppm) spectrum of **2e**.

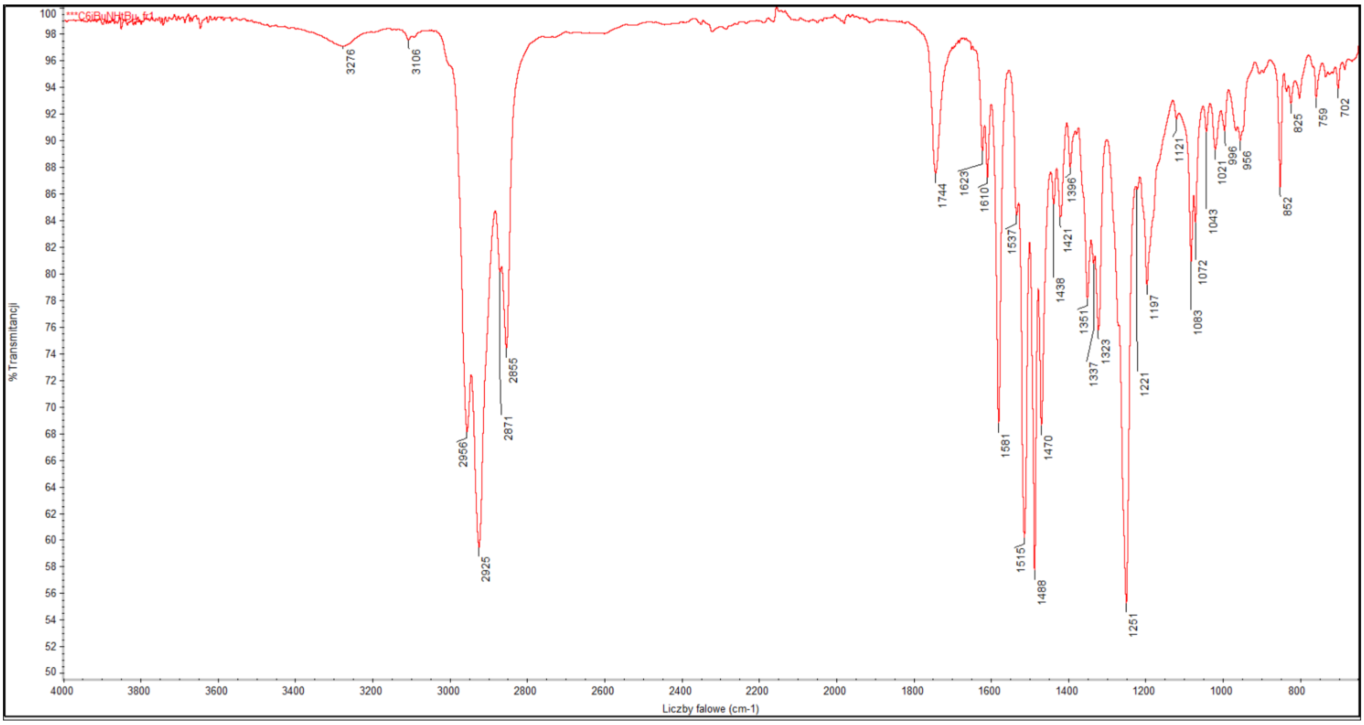

Figure S24. IR spectrum of **2e**.

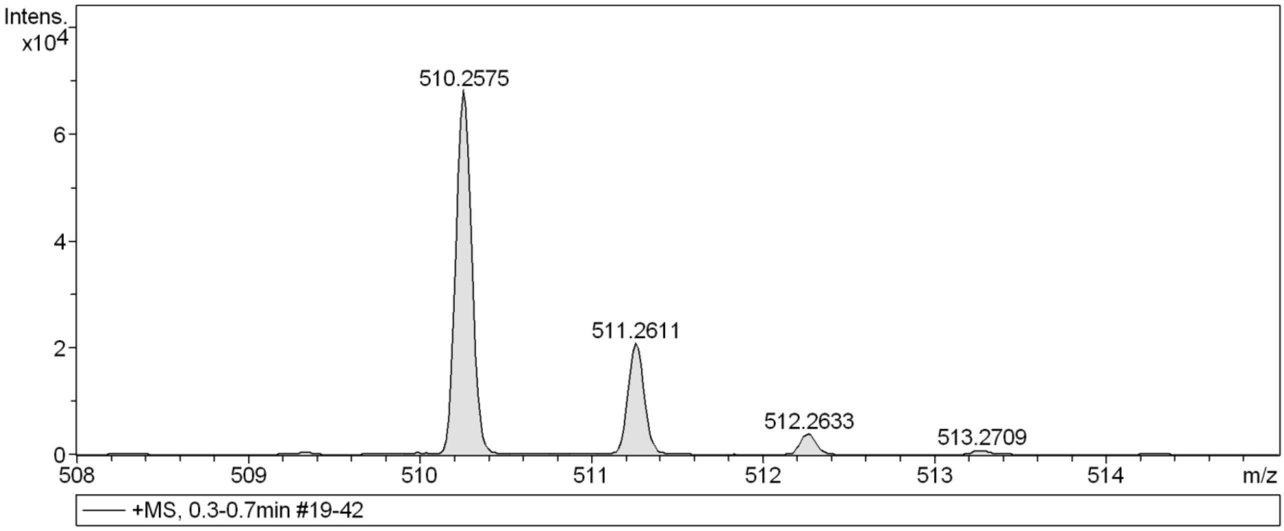

Figure S25. HRMS (ESI) spectrum of **2e**.

N-(5-(tert-butyl)-2-nitrophenyl)-5-isobutoxy-2-nitro-4-(octyloxy)aniline (2f).

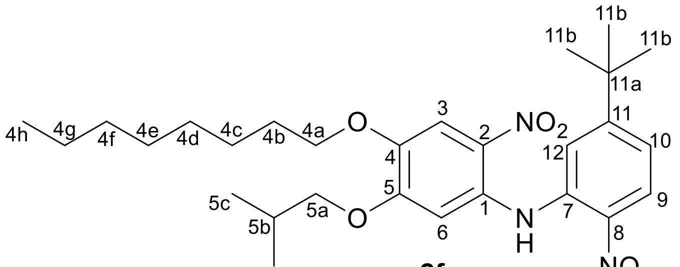

2f

^{1}H NMR ($CDCl_3$, 300 MHz, δ ppm): 11.18 (s, 1H, H_{N-H}), 8.15 (d, $^3J_{H9-H10}$ = 8.92 Hz, 1H, H_9), 7.70 (s, 1H, H_3), 7.59 (d, $^4J_{H10-H12}$ = 2.09 Hz, 1H, H_{12}), 7.09 (dd, $^3J_{H9-H10}$ = 8.92 Hz, $^4J_{H10-H12}$ = 2.09 Hz, 1H, H_{10}), 6.96 (s, 1H, H_6), 4.04 (t, $^3J_{H4a-H4b}$ = 6.46 Hz, 2H, H_{4a}), 3.69 (d, $^3J_{H5a-H5b}$ = 6.69 Hz, 2H, H_{5a}), 2.24 – 2.09 (m, 1H, H_{5b}), 1.91 – 1.79 (m, 2H, H_{4b}), 1.55 – 1.45 (m, 2H, H_{4c}), 1.44 – 1.24 (m, 8H, $H_{4d,4e,4f,4g}$), 1.32 (s, 9H, H_{11b}), 1.02 (d, $^3J_{H5b-H5c}$ = 6.75 Hz, 6H, H_{5c}), 0.90 (t, $^3J_{H4g-H4h}$ = 7.02 Hz, 3H, H_{4h}).

$^{13}C\{^1H\}$ NMR ($CDCl_3$, 75 MHz, δ ppm): 159.6 (C_8), 156.1 (C_5), 144.5 (C_4), 137.9 (C_7), 136.7 (C_{11}), 134.3 (C_2), 131.2 (C_1), 127.3 (C_9), 119.7 (C_{10}), 117.4 (C_{12}), 110.3 (C_3), 102.6 (C_6), 76.3 (C_{5a}), 70.3 (C_{4a}), 36.2 (C_{11a}), 32.4 (C_{4f}), 31.5 (C_{11b}), 29.9 (C_{4b}), 29.9 (C_{4d}), 29.7 (C_{4e}), 28.8 (C_{5b}), 26.6 (C_{4c}), 23.3 (C_{4g}), 19.7 (C_{5c}), 14.2 (C_{4h}).

FT-IR (ATR, ν_{max}, (neat)/cm^{-1}): 3300, 2958, 2928, 2873, 2856, 1610, 1581, 1535, 1515, 1486, 1468, 1437, 1395, 1352, 1320, 1252, 1198, 1084, 1068, 1044, 1014, 996, 970, 955, 861, 850, 824, 809, 759, 700.

HRMS (ESI) *m/z* Calculated for $C_{28}H_{41}N_3O_6Na$ $[M+Na]^+$, 538.2888; found: 538.2888.

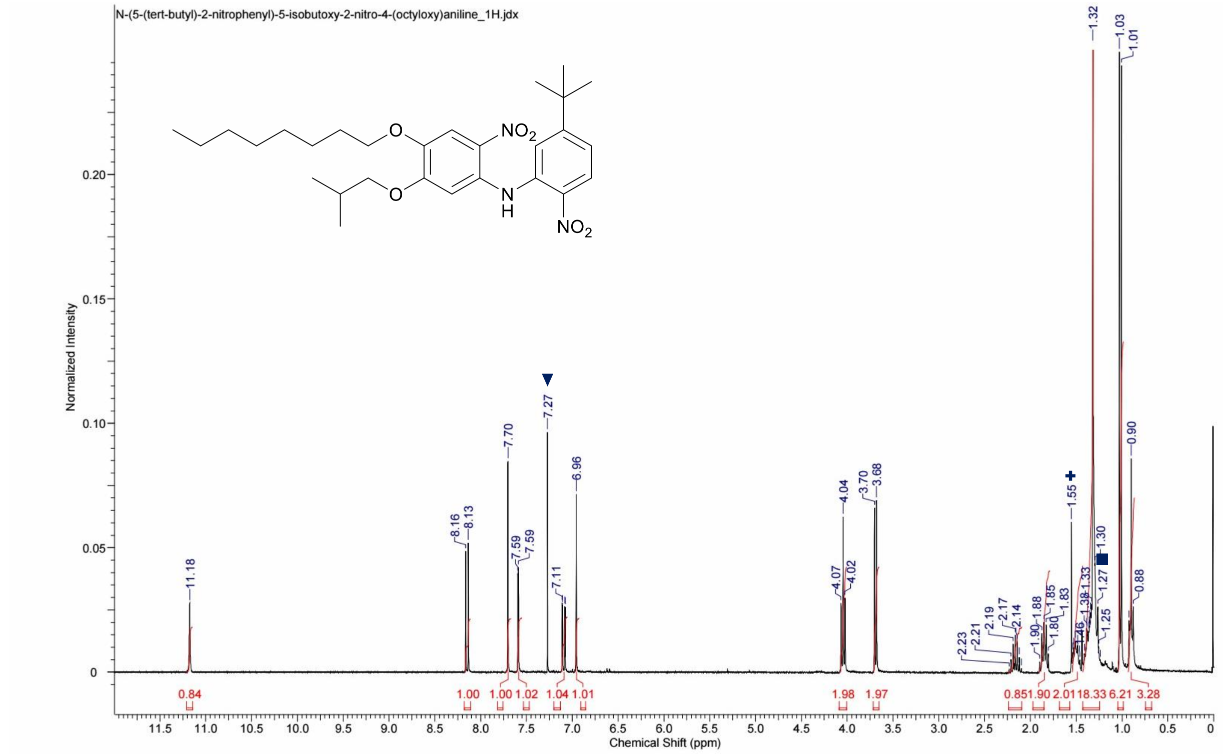

Figure S26. ^{1}H NMR ($CDCl_3$, 300 MHz, δ ppm) spectrum of **2f**.

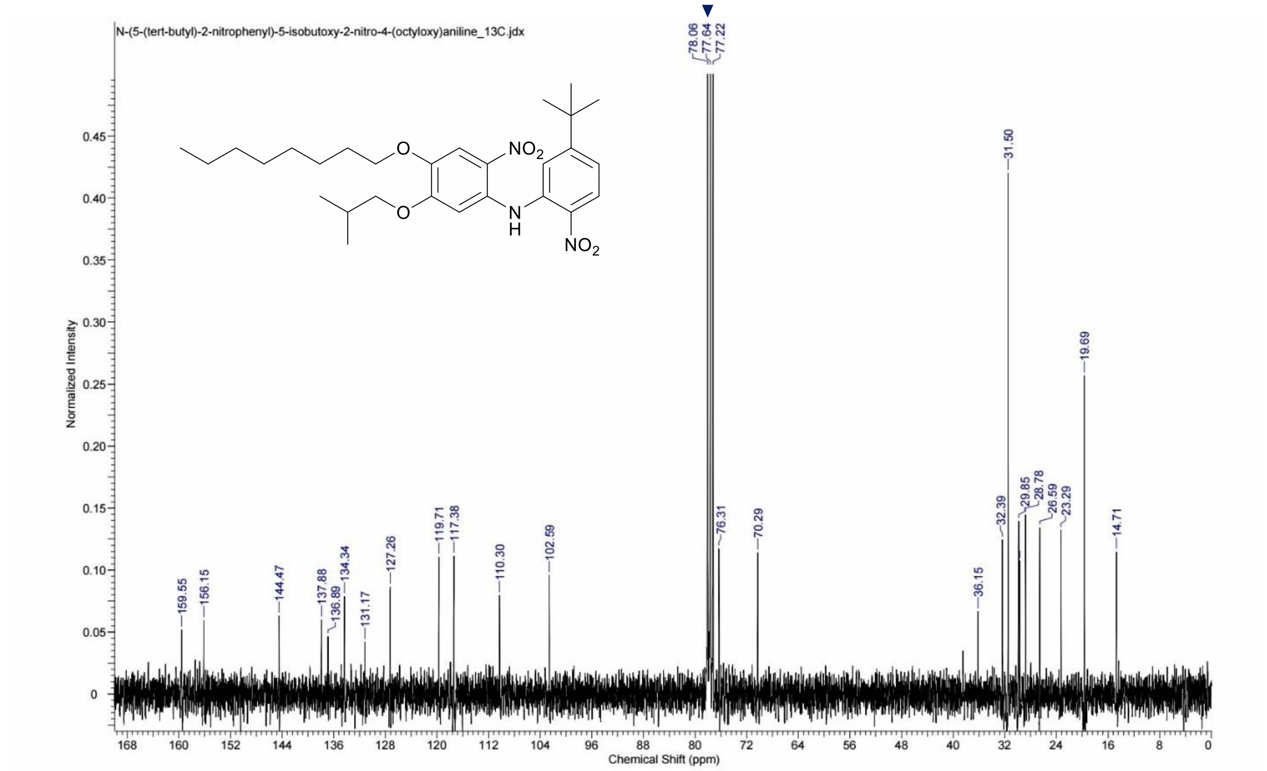

Figure S27. $^{13}C\{^{1}H\}$ NMR ($CDCl_3$, 75 MHz, δ ppm) NMR spectrum of **2f**.

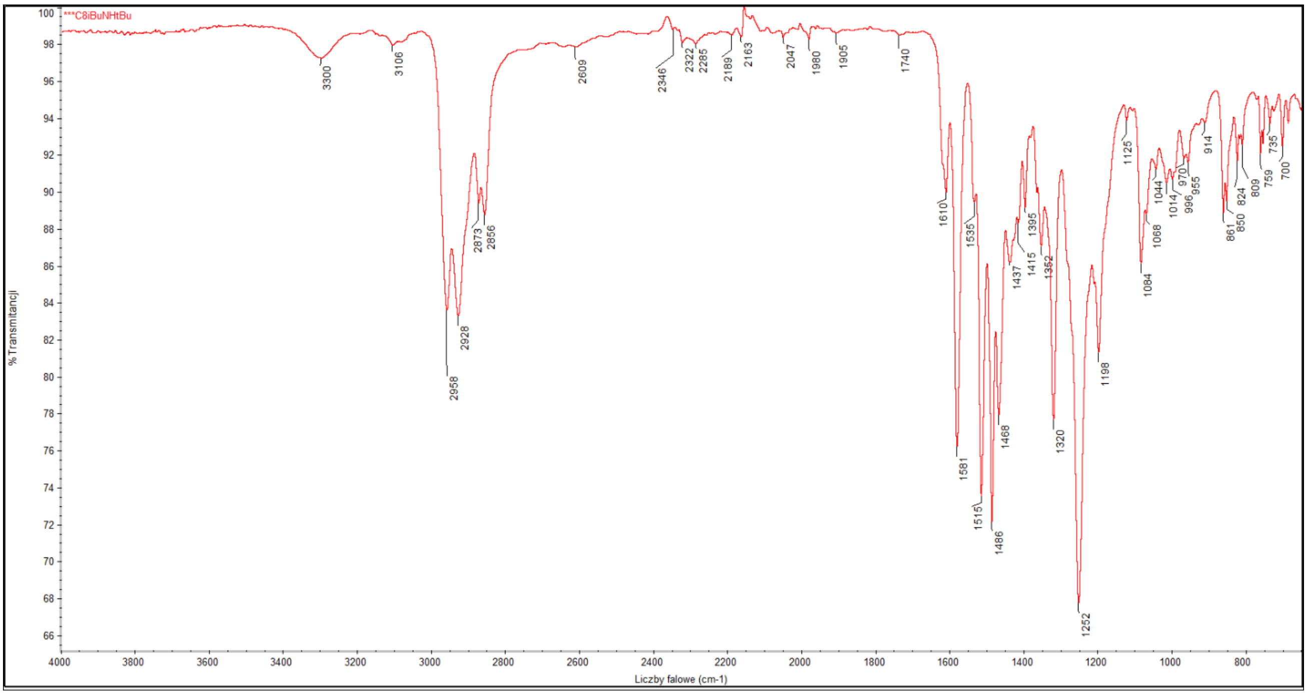

Figure S28. IR spectrum of **2f**.

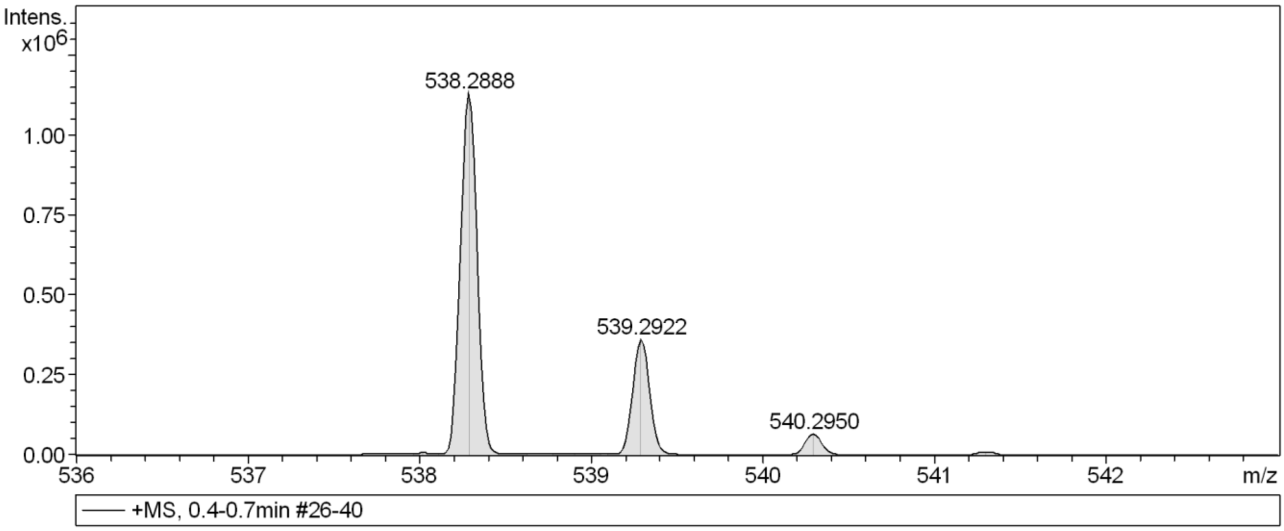

Figure S29. HRMS (ESI) spectrum of **2f**.

***N*-(5-(tert-butyl)-2-nitrophenyl)-4-(decyloxy)-5-isobutoxy-2-nitroaniline (2g).**

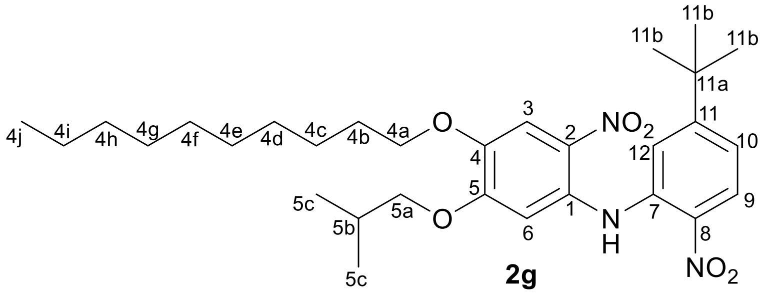

2g

^{1}H NMR ($CDCl_3$, 300 MHz, δ ppm): 11.18 (s, 1H, H_{N-H}), 8.15 (d, $^3J_{H9-H10}$ = 8.86 Hz, 1H, H_9), 7.70 (s, 1H, H_3), 7.59 (d, $^4J_{H10-H12}$ = 1.97 Hz, 1H, H_{12}), 7.09 (dd, $^3J_{H9-H10}$ = 8.86 Hz, $^4J_{H10-H12}$ = 1.97Hz, 1H, H_{10}), 6.96 (s, 1H, H_6), 4.04 (t, $^3J_{H4a-H4b}$ = 6.40 Hz, 2H, H_{4a}), 3.69 (d, $^3J_{H5a-H5b}$ = 6.65 Hz, 2H, H_{5a}), 2.24 – 2.09 (m, 1H, H_{5b}), 1.91 – 1.79 (m, 2H, H_{4b}), 1.57 – 1.45 (m, 2H, H_{4c}), 1.44 – 1.24 (m, 12H, $H_{4d,4e,4f,4g,4h,4i}$), 1.32 (s, 9H, H_{11b}), 1.02 (d, $^3J_{H5b-H5c}$ = 6.77 Hz, 6H, H_{5c}), 0.90 (t, $^3J_{H4i-H4j}$ = 6.67 Hz, 3H, H_{4j}).

^{13}C{^{1}H} NMR ($CDCl_3$, 75 MHz, δ ppm): 159.6 (C_8), 156.2 (C_5), 144.5 (C_4), 137.9 (C_7), 136.9 (C_{11}), 134.4 (C_2), 131.3 (C_1), 127.3 (C_9), 119.7 (C_{10}), 117.4 (C_{12}), 110.3 (C_3), 102.6 (C_6), 76.3 (C_{5a}), 70.3 (C_{4a}), 36.2 (C_{11a}), 32.4 (C_{4h}), 31.5 (C_{11b}), 30.2 – 29.8 ($C_{4b,4d,4e,4f}$), 29.7 (C_{4g}), 28.8 (C_{5b}), 26.6 (C_{4c}), 23.3 (C_{4i}), 19.7 (C_{5c}), 14.7 (C_{4j}).

FT-IR (ATR, ν_{max}, (neat)/cm^{-1}): 3307, 2958, 2926, 2869, 2855, 1610, 1580, 1515, 1486, 1468, 1435, 1393, 1347, 1319, 1252, 1199, 1082, 1064, 1040, 1001, 953, 860, 820, 807, 757, 737, 698.

HRMS (ESI) *m/z* Calculated for $C_{22}H_{29}N_3O_6Na$ [M+Na]$^+$, 566.3201; found: 566.3201.

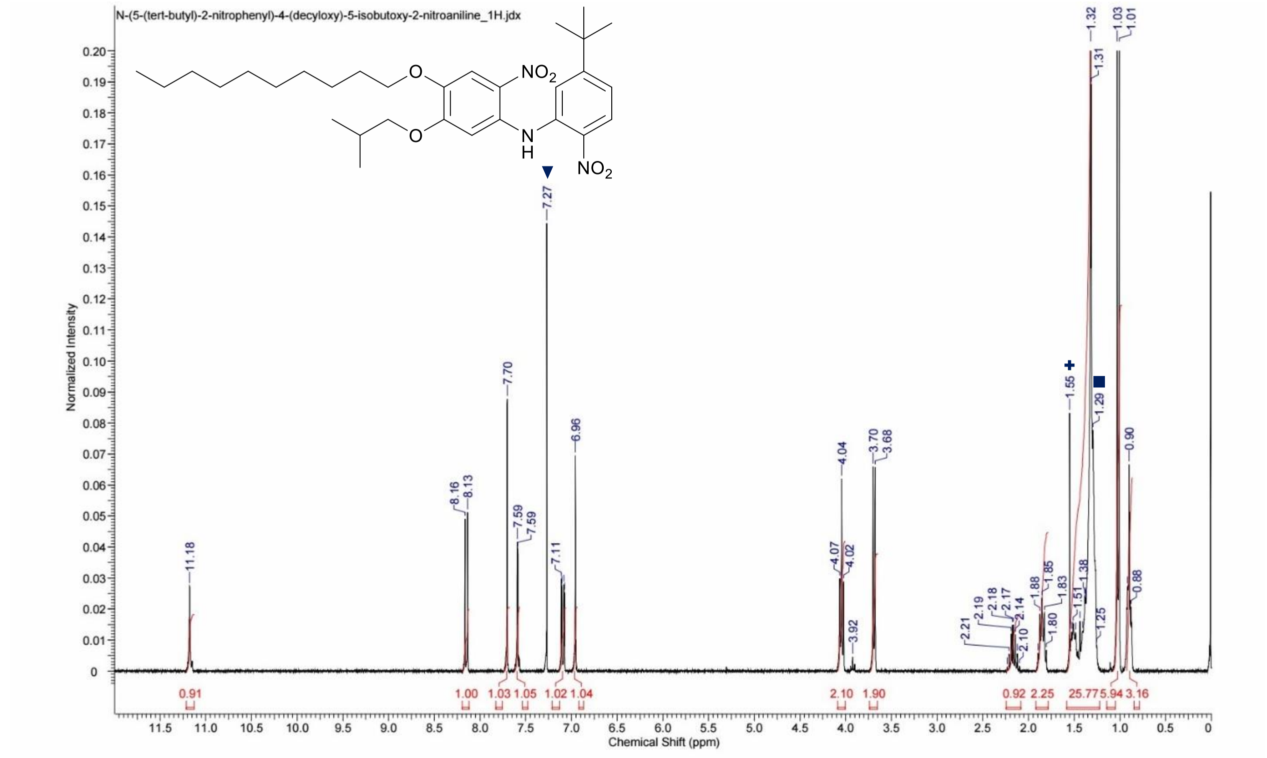

Figure S30. ^{1}H NMR ($CDCl_3$, 300 MHz, δ ppm) spectrum of **2g**.

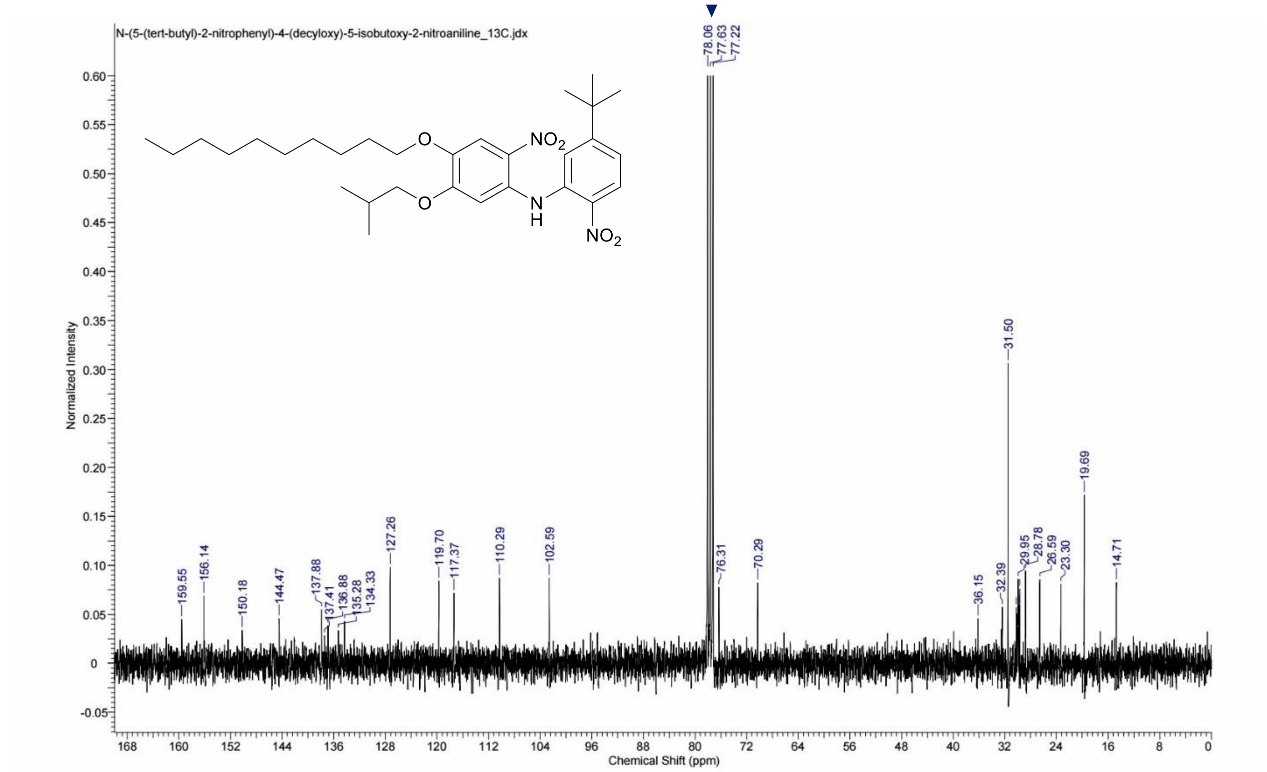

Figure S31. $^{13}C\{^{1}H\}$ NMR ($CDCl_3$, 75 MHz, δ ppm) NMR spectrum of **2g**.

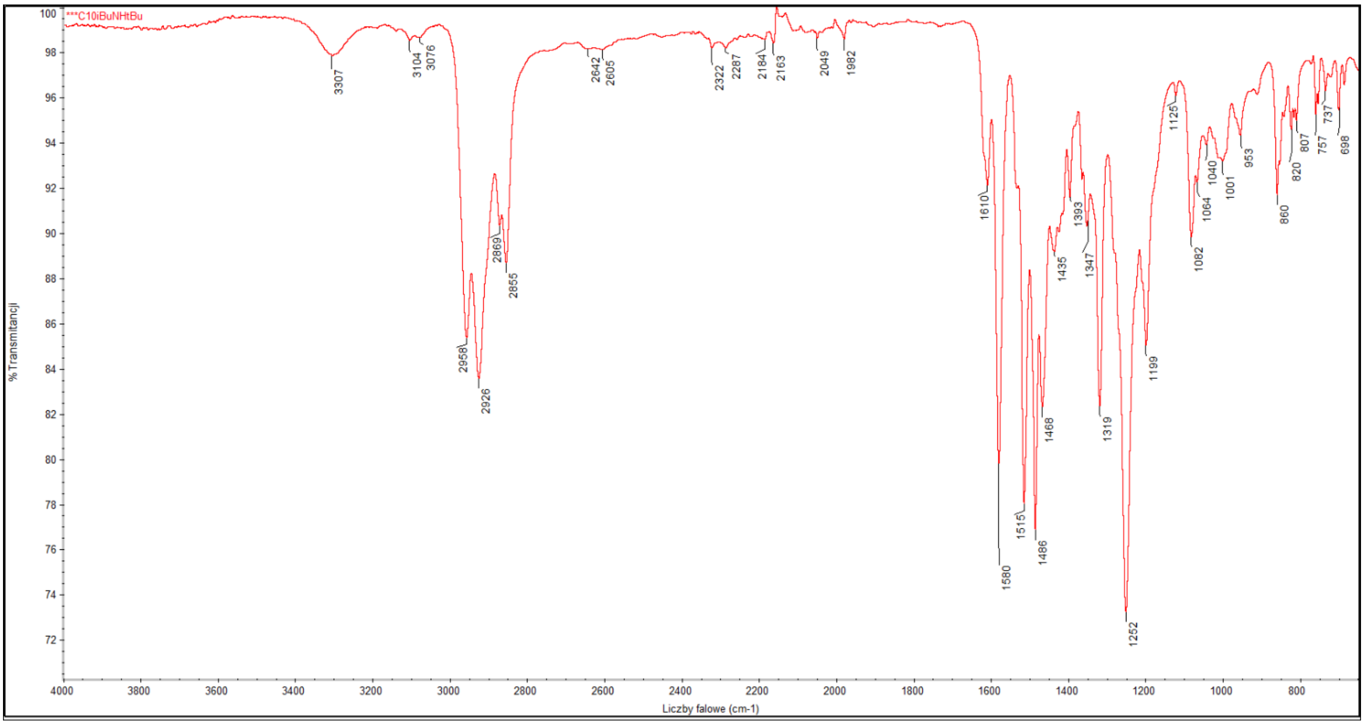

Figure S32. IR spectrum of **2g**.

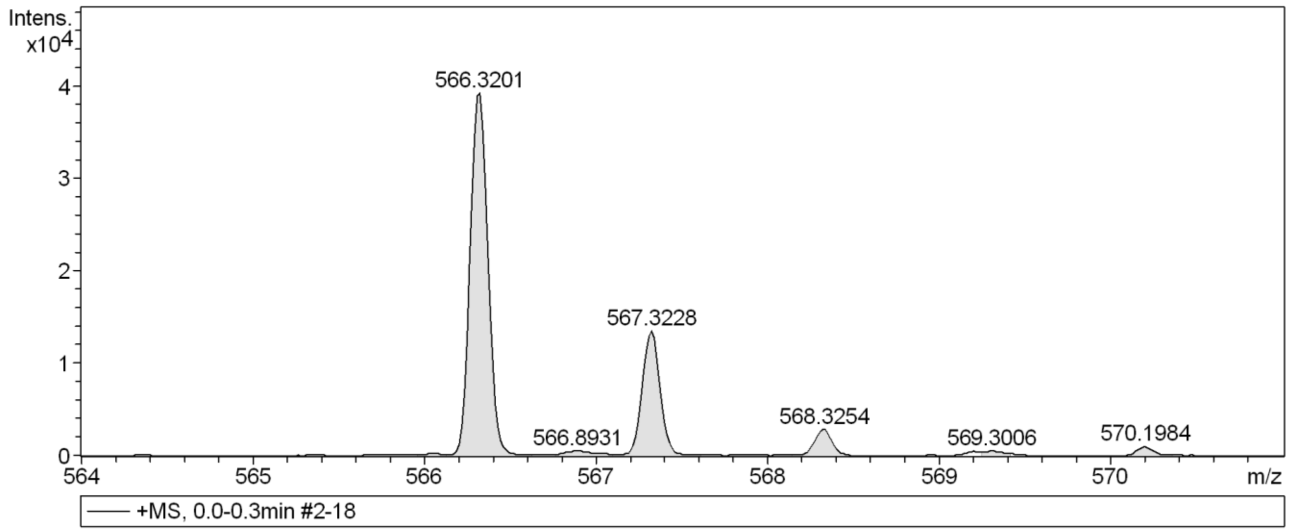

Figure S33. HRMS (ESI) spectrum of **2g**.

5-isobutoxy-4-methoxy-2-nitro-*N*-(2-nitro-4-(trifluoromethyl)phenyl)aniline (3a).

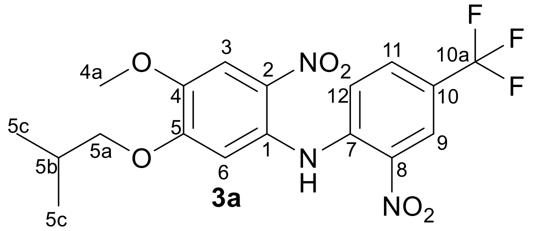

3a

^{1}H NMR ($CDCl_3$, 300 MHz, δ ppm): 11.06 (s, 1H, H_{N-H}), 8.51 (d, $^{4}J_{H9-H11}$ = 2.19 Hz, 1H, H_9), 7.70 (s, 1H, H_3), 7.68 (dd, $^{3}J_{H11-H12}$ = 8.93 Hz, $^{4}J_{H9-H11}$ = 2.19 Hz, 1H, H_{11}), 7.53 (d, $^{3}J_{H11-H12}$ = 8.93 Hz, 1H, H_{12}), 6.94 (s, 1H, H_6), 3.96 (s, 3H, H_{4a}), 3.76 (d, $^{3}J_{H5b-H5c}$ = 6.68 Hz, 2H, H_{5a}), 2.19 (m, 1H, H_{5b}), 1.05 (d, $^{3}J_{H5b-H5c}$ = 6.79 Hz, 6H, H_{5c}).

^{13}C{^{1}H} NMR ($CDCl_3$, 75 MHz, δ ppm): 155.3 (C_5), 146.8 (C_4), 142.3 (C_7), 136.4 (C_8), 133.8 (C_2), 132.0 (q, $^{3}J_{C-F}$ = 3 Hz, C_{11}), 130.8 (C_1), 125.5 (q, $^{3}J_{C-F}$ = 4 Hz C_9), 122.9 – 121.9 (m, C_{10}), 118.8 (C_{12}), 109.1 (C_3), 105.7 (C_6), 76.6 (C_{5a}), 57.2 (C_{4a}), 28.8 (C_{5b}), 19.7 (C_{5c}), signal from C_{10a} is missing.

^{19}F NMR ($CDCl_3$, 282 MHz, δ ppm): –63.43 (s, 3F, F_{CF3}).

FT-IR (ATR, v_{max}, (neat)/cm^{-1}): 3303, 3113, 2976, 2960, 2932, 2919, 2878, 2851, 2834, 1633, 1613, 1584, 1514, 1465, 1442, 1412, 1359, 1323, 1301, 1258, 1180, 1153, 1127, 1109, 1085, 1066, 1034, 1005, 975, 914, 892, 866, 853, 826, 799, 783, 758, 683.

HRMS (ESI) *m/z* Calculated for $C_{18}H_{18}N_3O_6F_3Na$ $[M+Na]^+$, 452.1040; found: 452.1041.

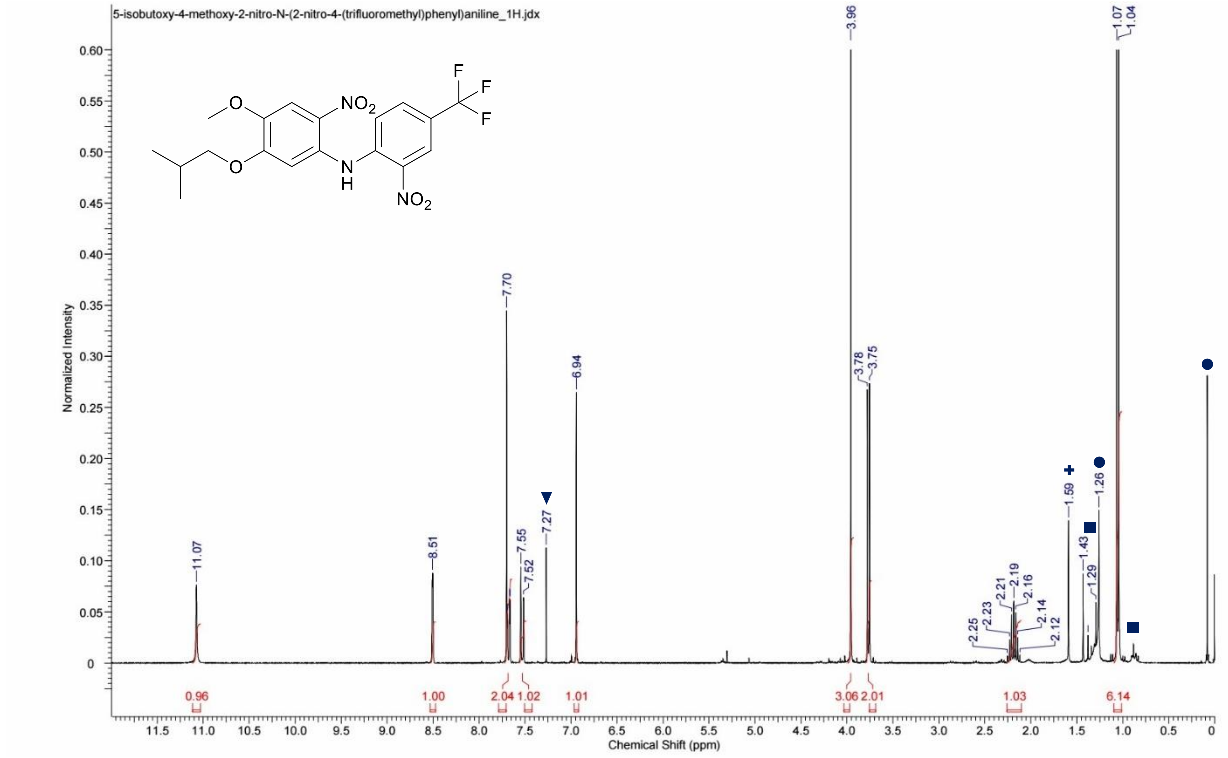

Figure S34. ^{1}H NMR ($CDCl_3$, 300 MHz, δ ppm) spectrum of **3a**.

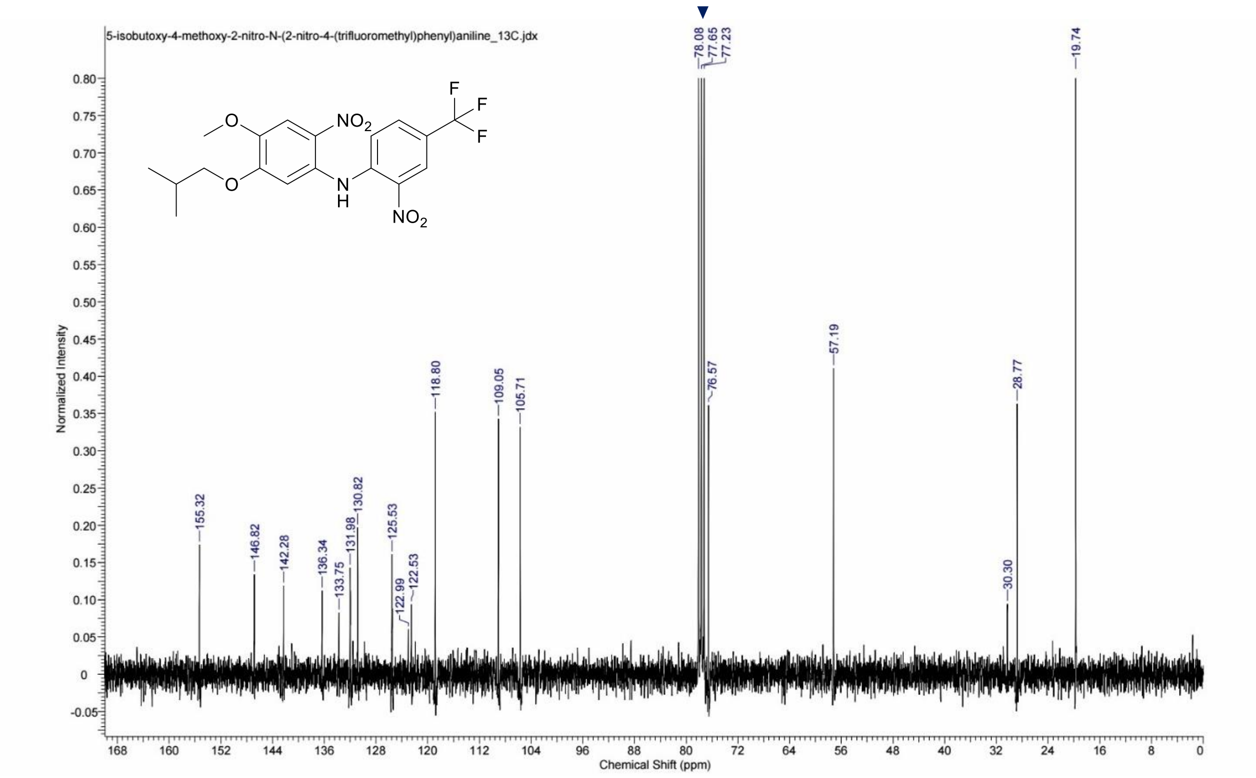

Figure S35. $^{13}C\{^{1}H\}$ NMR ($CDCl_3$, 75 MHz, δ ppm) NMR spectrum of **3a**.

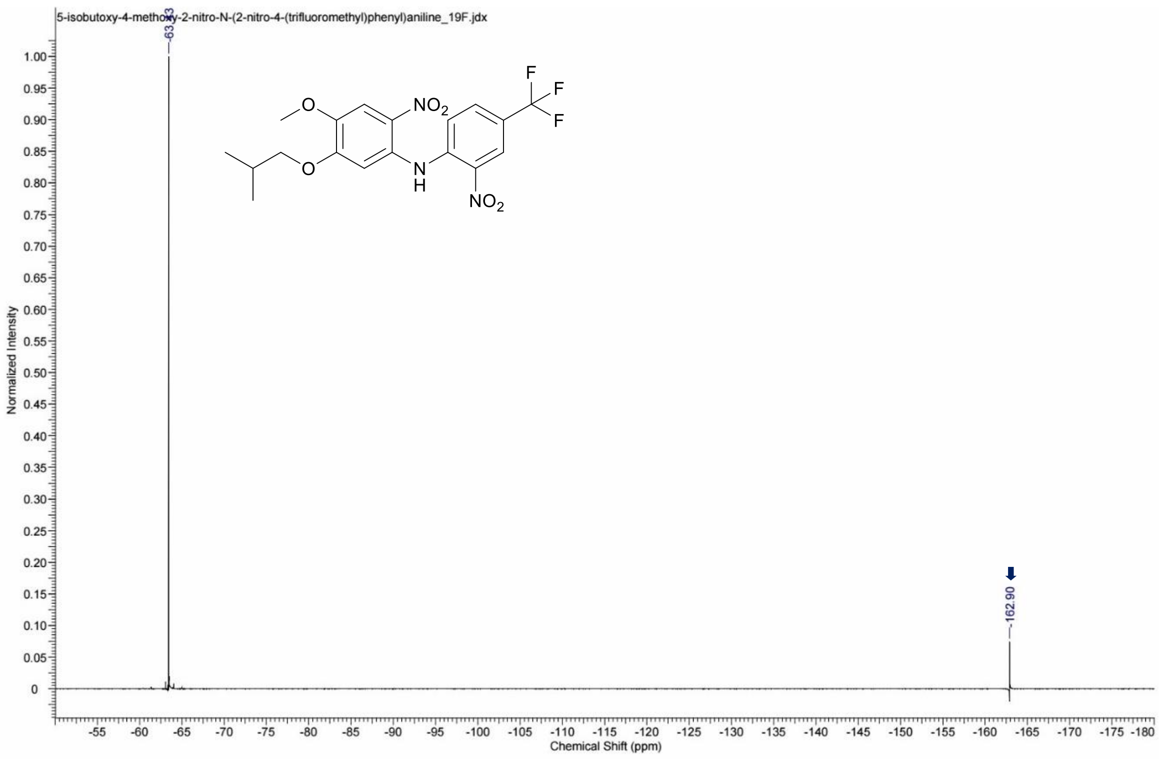

Figure S36. ^{19}F NMR ($CDCl_3$, 282 MHz, δ ppm) spectrum of **3a.**

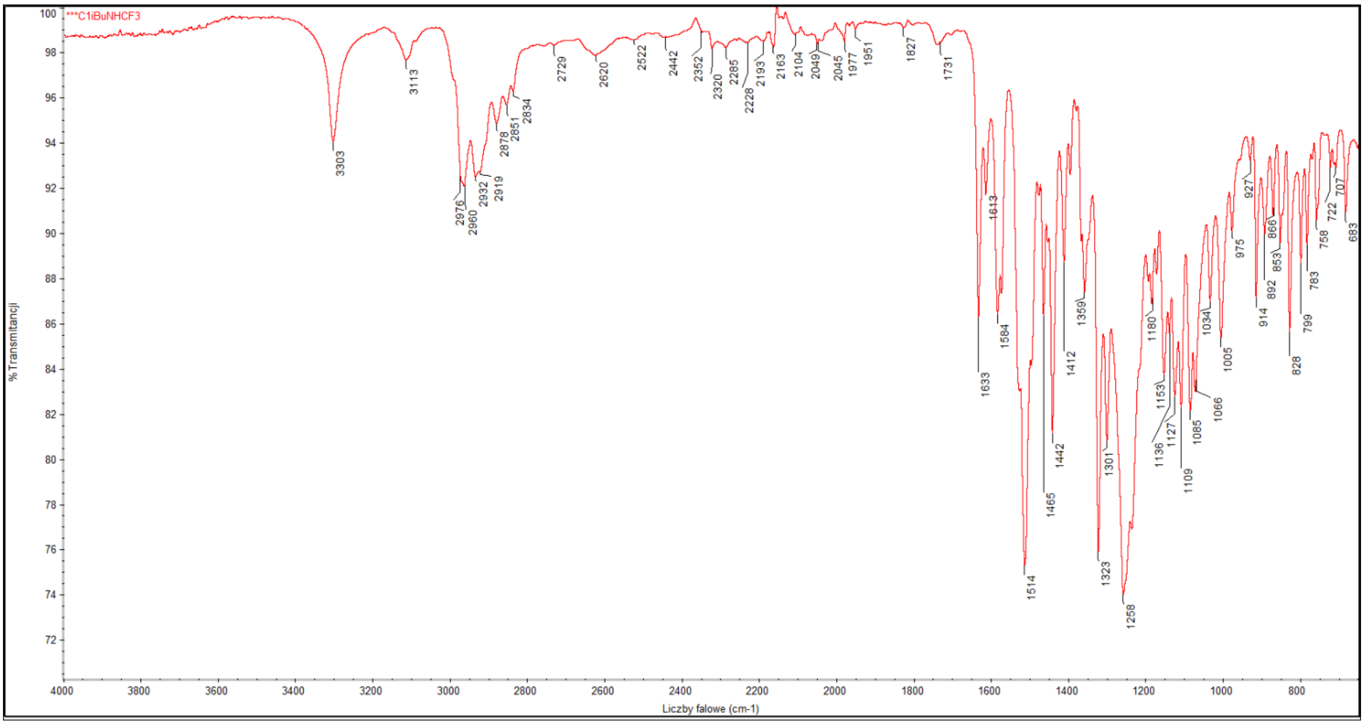

Figure S37. IR spectrum of **3a**.

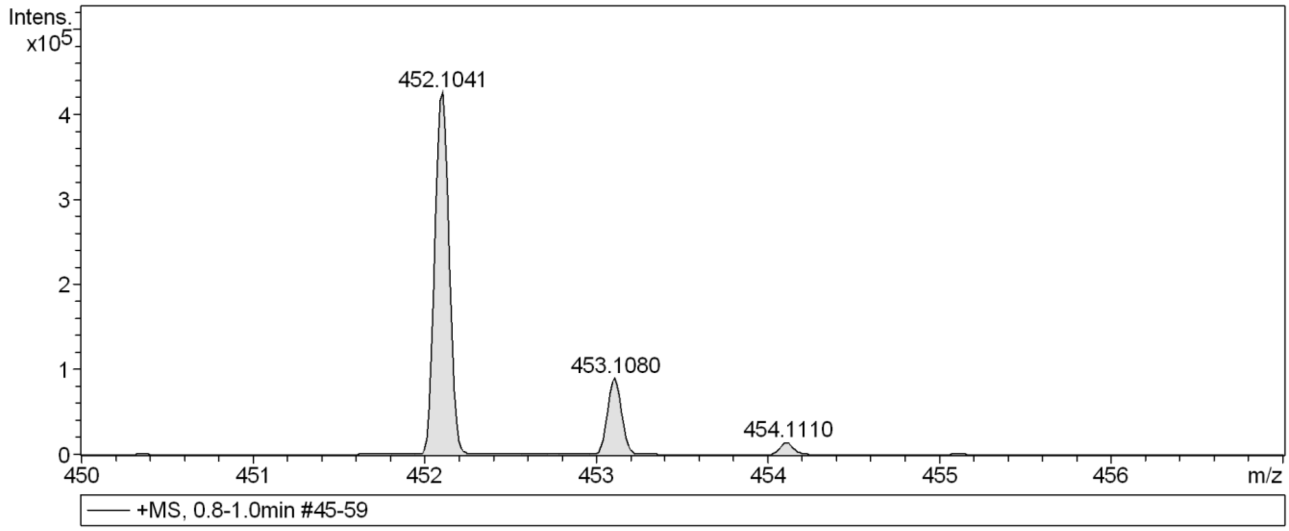

Figure S38. HRMS (ESI) spectrum of **3a**.

4-ethoxy-5-isobutoxy-2-nitro-*N*-(2-nitro-4-(trifluoromethyl)phenyl)aniline (3b).

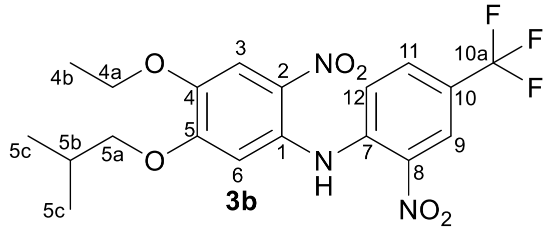

3b

^{1}H NMR ($CDCl_3$, 300 MHz, δ ppm): 11.06 (s, 1H, H_{N-H}), 8.48 (d, $^{4}J_{H9-H11}$ = 2.20 Hz, 1H, H_9), 7.68 (s, 1H, H_3), 7.67 (dd, $^{3}J_{H11-H12}$ = 8.75 Hz, $^{4}J_{H9-H11}$ = 2.20 Hz, 1H, H_{11}), 7.54 (d, $^{3}J_{H11-H12}$ = 8.75 Hz, 1H, H_{12}), 6.95 (s, 1H, H_6), 4.16 (q, $^{3}J_{H4a-H4b}$ = 6.96 Hz, 2H, H_{4a}), 3.77 (d, $^{3}J_{H5a-H5b}$ = 6.57 Hz, 2H, H_{5a}), 2.18 (m, 1H, H_{5b}), 1.50 (t, $^{3}J_{H4a-H4b}$ = 6.96 Hz, 3H, H_{4b}), 1.05 (d, $^{3}J_{H5b-H5c}$ = 6.71 Hz, 6H, H_{5c}).

^{13}C{^{1}H} NMR ($CDCl_3$, 75 MHz, δ ppm): 155.7 (C_5), 146.2 (C_4), 142.3 (C_7), 136.2 (C_8), 133.8 (C_2), 132.0 (q, $^{3}J_{C-F}$ = 3 Hz, C_{11}), 130.7 (C_1), 125.4 (q, $^{3}J_{C-F}$ = 4 Hz C_9), 122.9 – 121.9 (m, C_{10}), 118.8 (C_{12}), 110.5 (C_3), 106.0 (C_6), 76.5 (C_{5a}), 66.0 (C_{4a}), 28.8 (C_{5b}), 19.2 (C_{5c}), 15.2 (C_{4b}), signal from C_{10a} is missing.

^{19}F NMR ($CDCl_3$, 282 MHz, δ ppm): –63.39 (s, 3F, F_{CF3}).

FT-IR (ATR, ν_{max}, (neat)/cm^{-1}): 3318, 3104, 2978, 2961, 2921, 2871, 2851, 1634, 1583, 1572, 1543, 1517, 1504, 1470, 1435, 1397, 1358, 1324, 1275, 1256, 1236, 1217, 1197, 1175, 1147, 1104, 1082, 1064, 1040, 1005, 920, 900, 879, 840, 824, 809, 782, 763, 748, 683.

HRMS (ESI) *m/z* Calculated for $C_{19}H_{20}N_3O_6F_3Na$ $[M+Na]^+$, 466.1197; found: 466.1197.

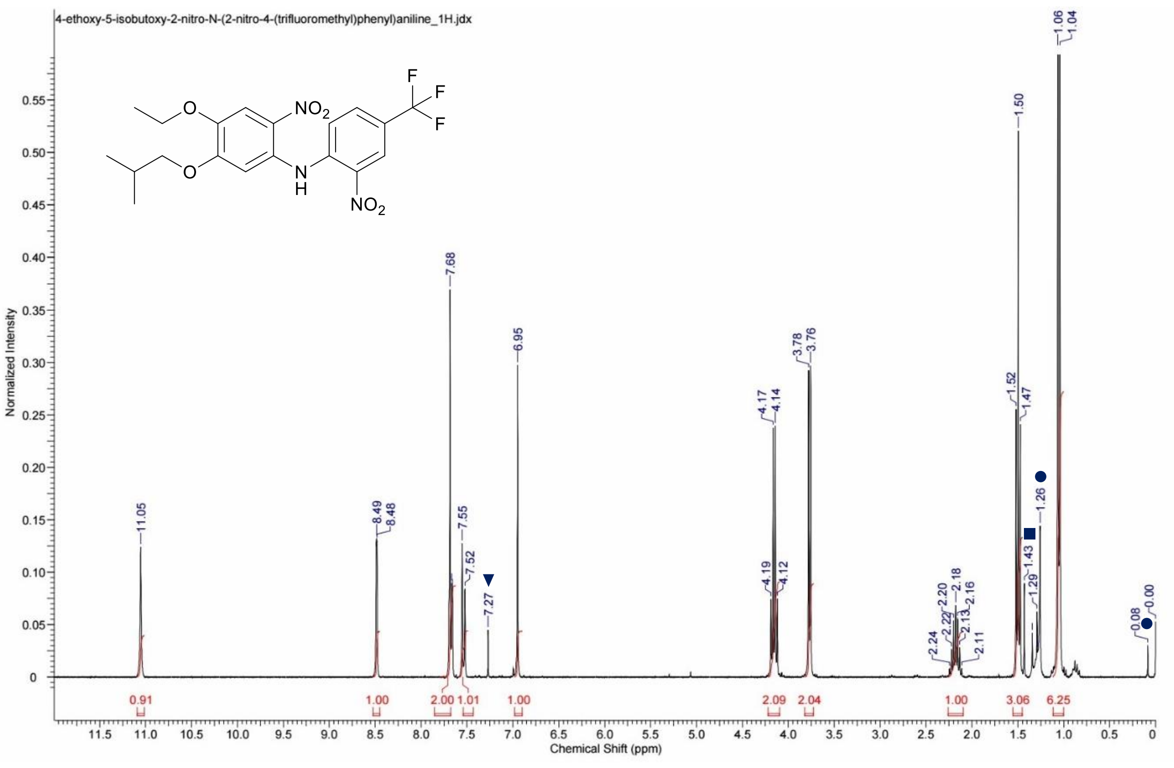

Figure S39. ^{1}H NMR ($CDCl_3$, 300 MHz, δ ppm) spectrum of **3b**.

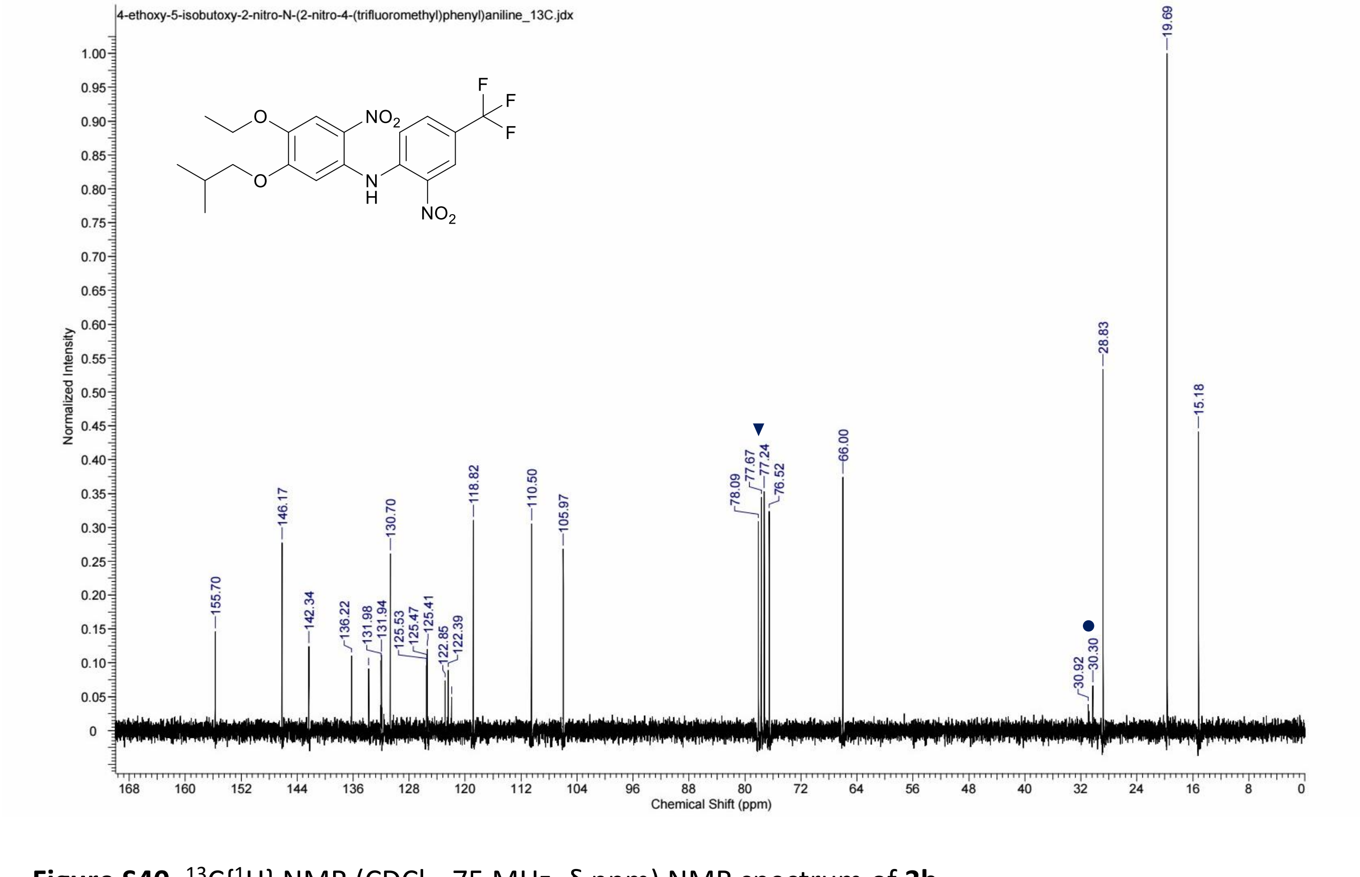

Figure S40. $^{13}C\{^{1}H\}$ NMR ($CDCl_3$, 75 MHz, δ ppm) NMR spectrum of **3b**.

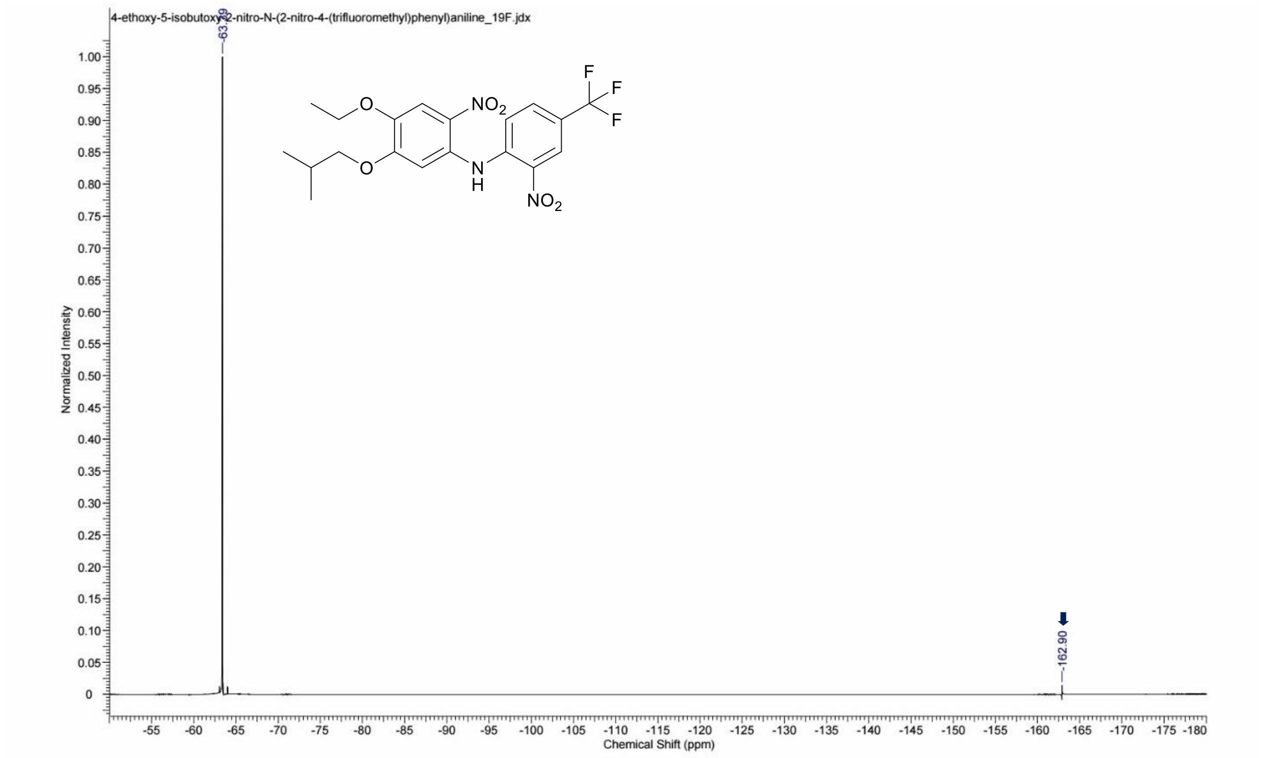

Figure S41. ^{19}F NMR ($CDCl_3$, 282 MHz, δ ppm) spectrum of **3b**.

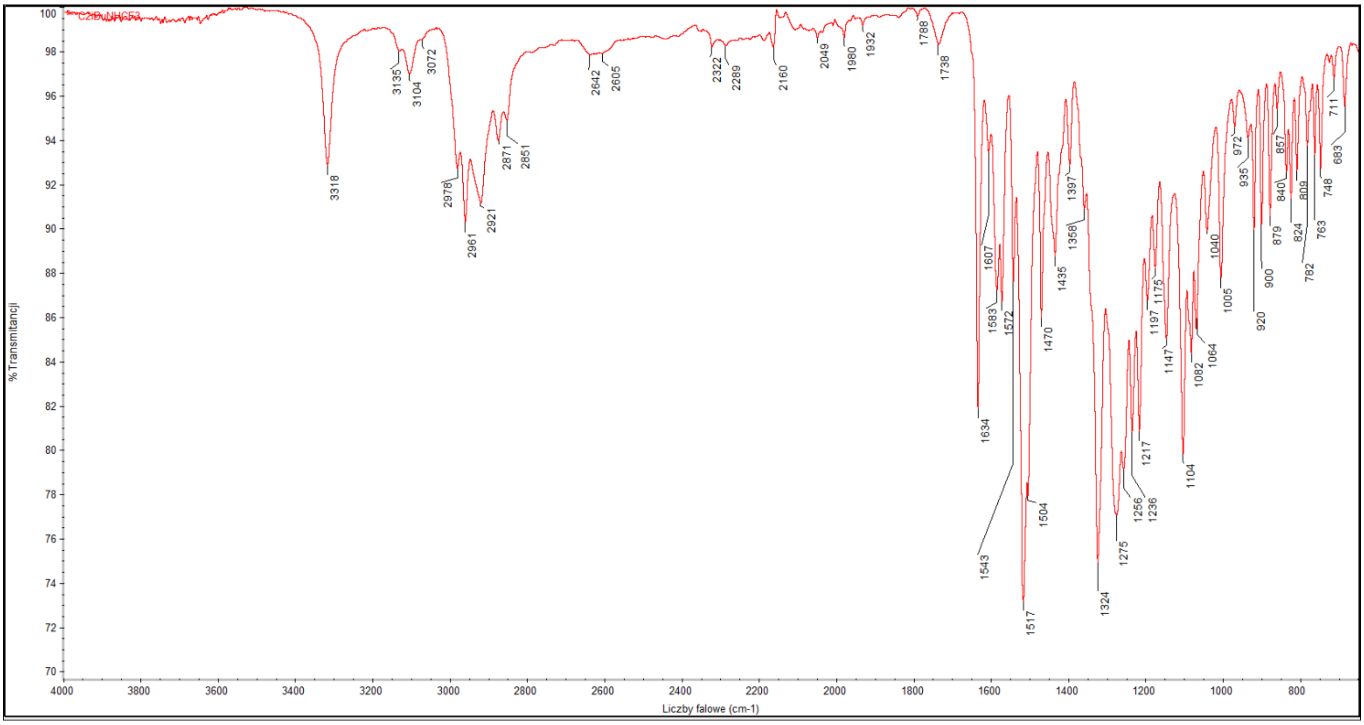

Figure S42. IR spectrum of **3b**.

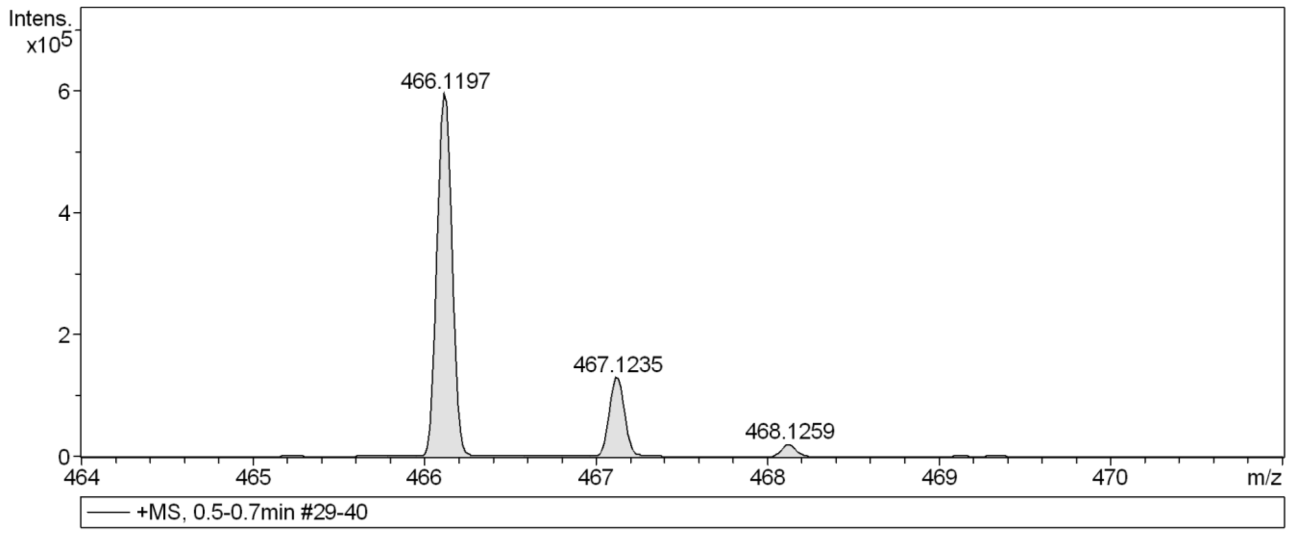

Figure S43. HRMS (ESI) spectrum of **3b**.

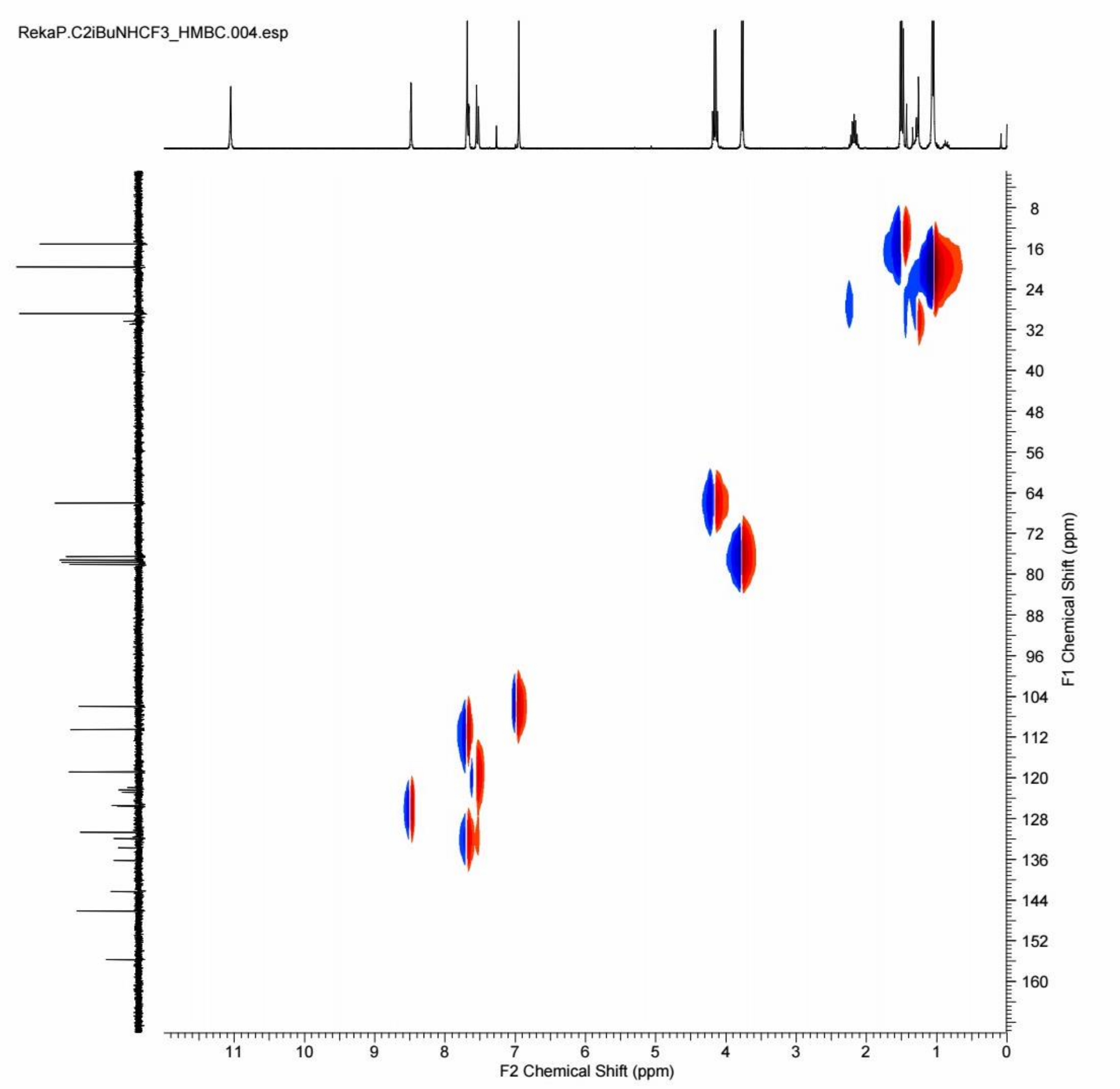

Figure S44. HSQC spectrum of **3b**.

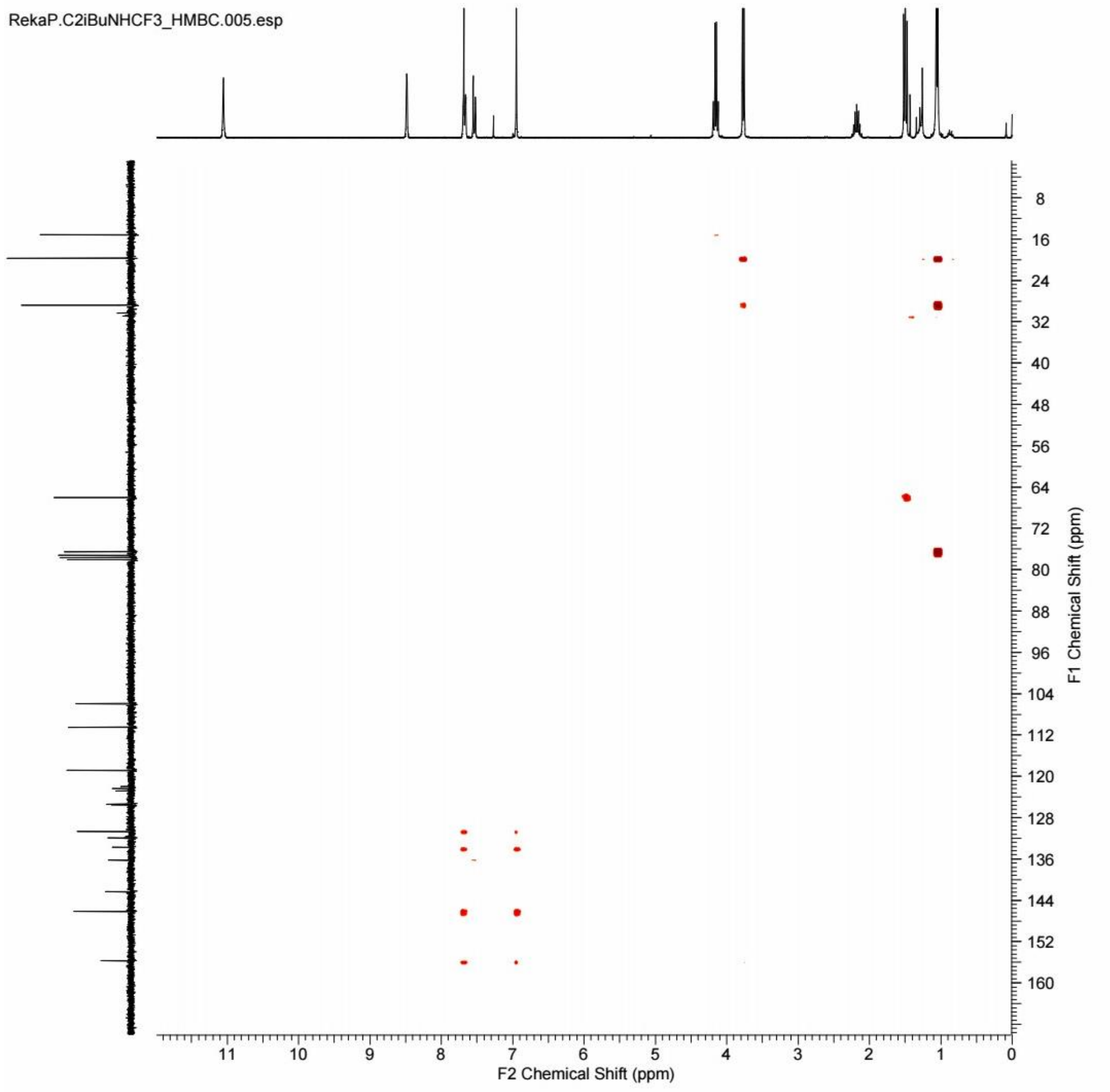

Figure S45. HMBC spectrum of **3b**.

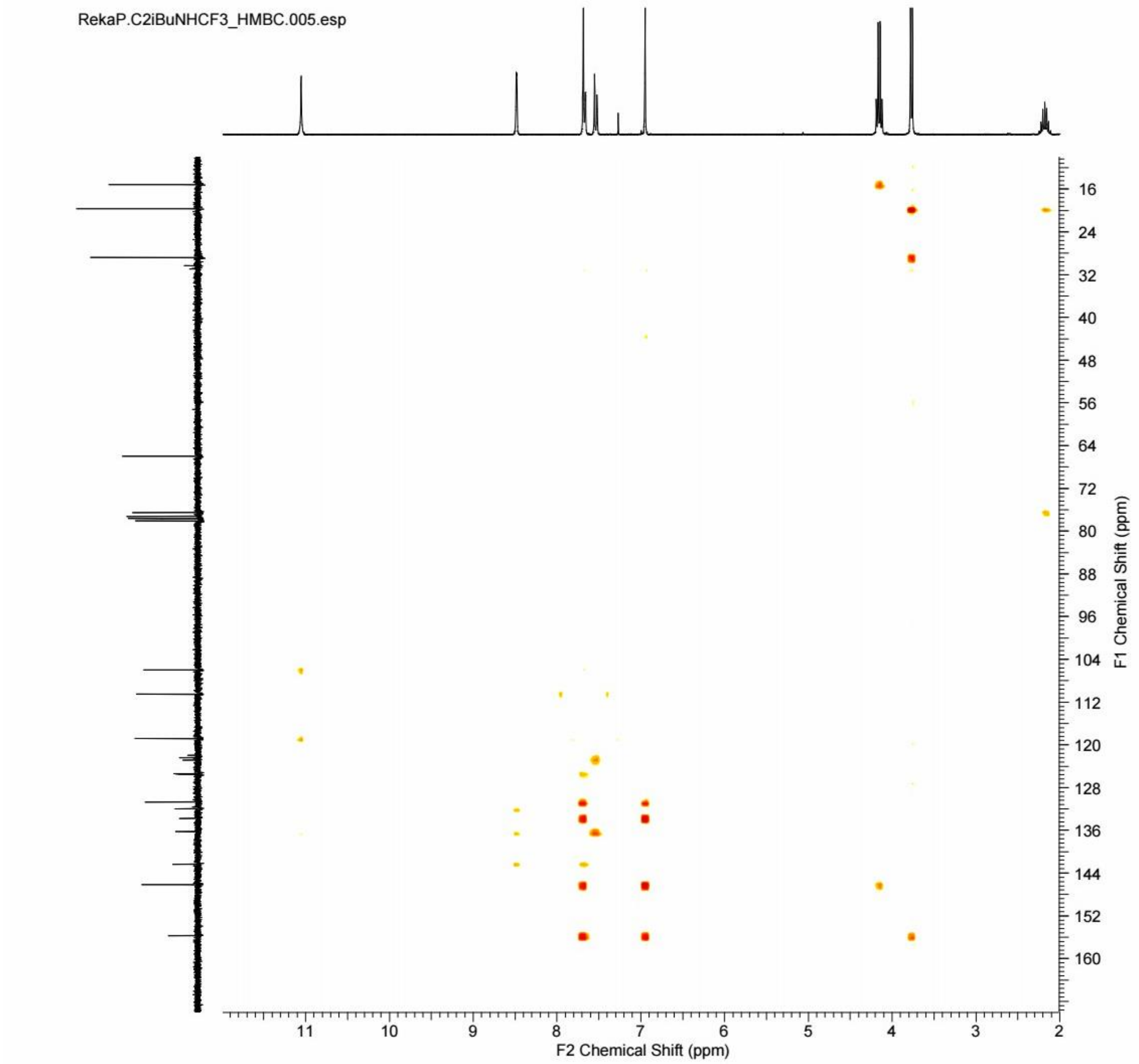

Figure S46. Zoomed region of HMBC spectrum of **3b**.

5-ethoxy-4-isobutoxy-2-nitro-*N*-(2-nitro-4-(trifluoromethyl)phenyl)aniline (3c).

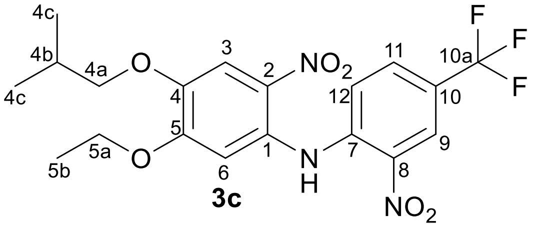

3c

^{1}H NMR ($CDCl_3$, 300 MHz, δ ppm): 11.08 (s, 1H, $H_{N–H}$), 8.52 (d, $^{4}J_{H9–H11}$ = 2.15 Hz, 1H, H_9), 7.70 (s, 1H, H_3), 7.66 (dd, $^{3}J_{H11–H12}$ = 8.96 Hz, $^{4}J_{H9–H11}$ = 2.15 Hz, 1H, H_{11}), 7.53 (d, $^{3}J_{H11–H12}$ = 8.96 Hz, 1H, H_{12}), 6.94 (s, 1H, H_6), 4.09 (q, $^{3}J_{H5a–H5b}$ = 6.99 Hz, 2H, H_{5a}), 3.84 (d, $^{3}J_{H4b–H4c}$ = 6.78 Hz, 2H, H_{4c}), 2.20 (m, 1H, H_{4b}), 1.49 (t, $^{3}J_{H5a–H5b}$ = 6.96 Hz, 3H, H_{5b}), 1.08 (d, $^{3}J_{H4b–H4c}$ = 6.74 Hz, 6H, H_{4c}).

^{13}C{^{1}H} NMR ($CDCl_3$, 75 MHz, δ ppm): 155.4 (C_5), 146.4 (C_4), 142.3 (C_7), 136.3 (C_8), 133.8 (C_2), 131.9 (q, $^{3}J_{C–F}$ = 3 Hz, C_{11}), 130.7 (C_1), 125.5 (q, $^{3}J_{C–F}$ = 4 Hz C_9), 123.0 – 121.9 (m, C_{10}), 118.9 (C_{12}), 110.3 (C_3), 105.9 (C_6), 76.7 (C_{4a}), 66.0 (C_{5a}), 28.8 (C_{4b}), 19.8 (C_{4c}), 15.1 (C_{5b}), signal from C_{10a} is missing.

^{19}F NMR ($CDCl_3$, 282 MHz, δ ppm): –63.36 (s, 3F, F_{CF3}).

FT-IR (ATR, ν_{max}, (neat)/cm^{-1}): 3312, 3104, 2978, 2967, 2947, 2926, 2880, 1637, 1614, 1583, 1538, 1520, 1495, 1471, 1444, 1424, 1396, 1365, 1322, 1277, 1261, 1233, 1213, 1192, 1156, 1111, 1082, 1071, 1036, 1020, 974, 913, 885, 853, 826, 809, 783, 761, 748, 684.

HRMS (ESI) *m/z* Calculated for $C_{19}H_{20}N_3O_6F_3Na$ [M+Na]$^+$, 466.1197; found: 466.1198.

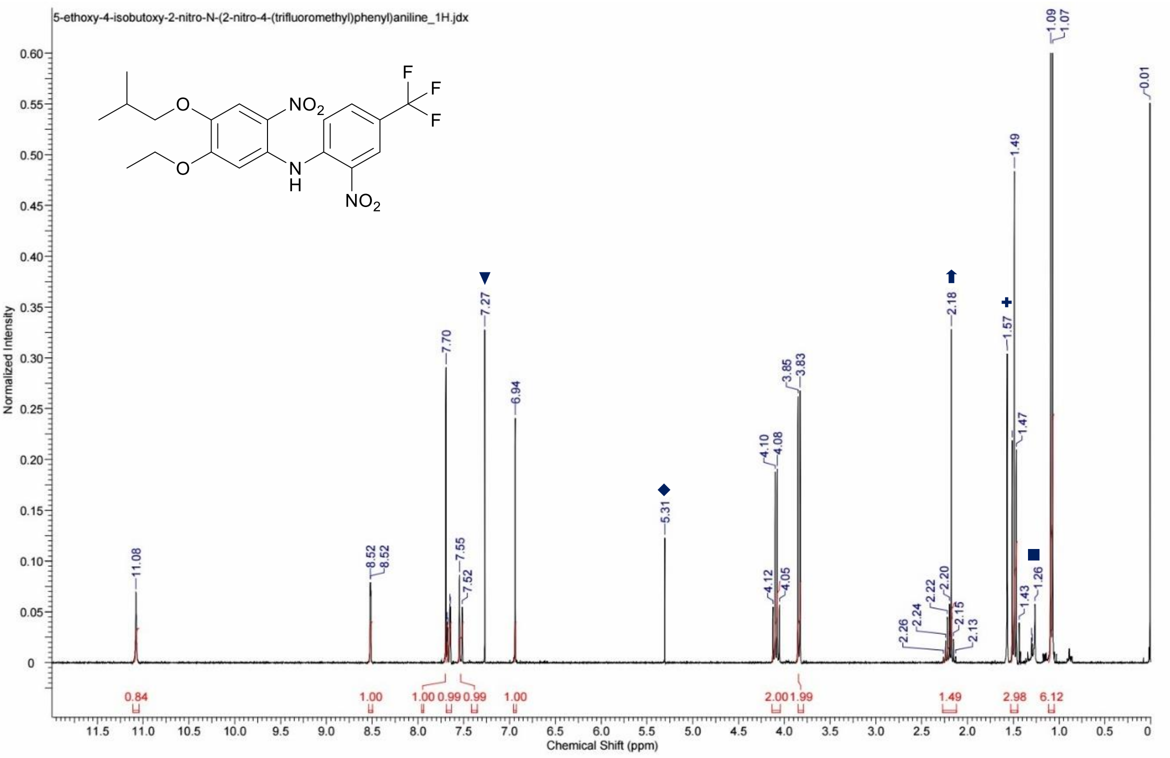

Figure S47. ^{1}H NMR ($CDCl_3$, 300 MHz, δ ppm) spectrum of **3c**.

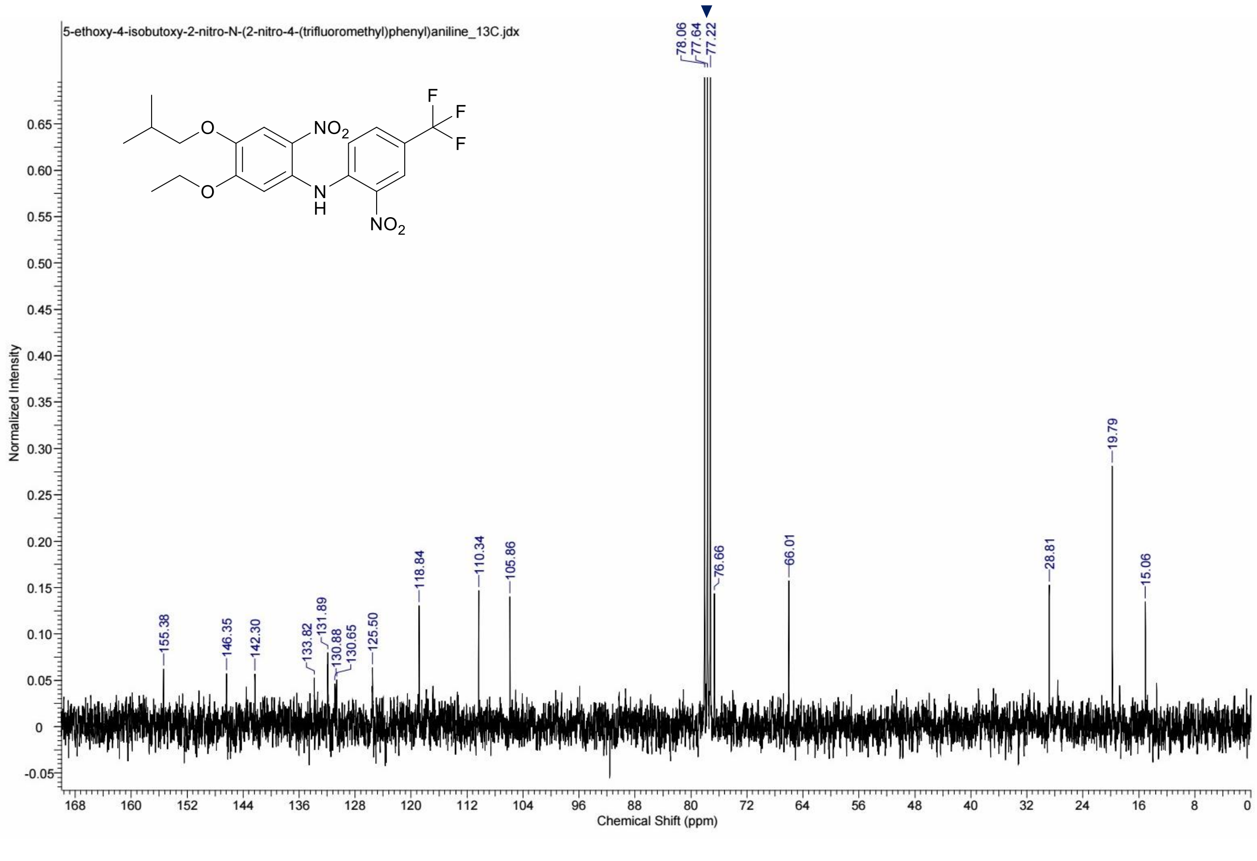

Figure S48. $^{13}C\{^1H\}$ NMR ($CDCl_3$, 75 MHz, δ ppm) NMR spectrum of **3c**.

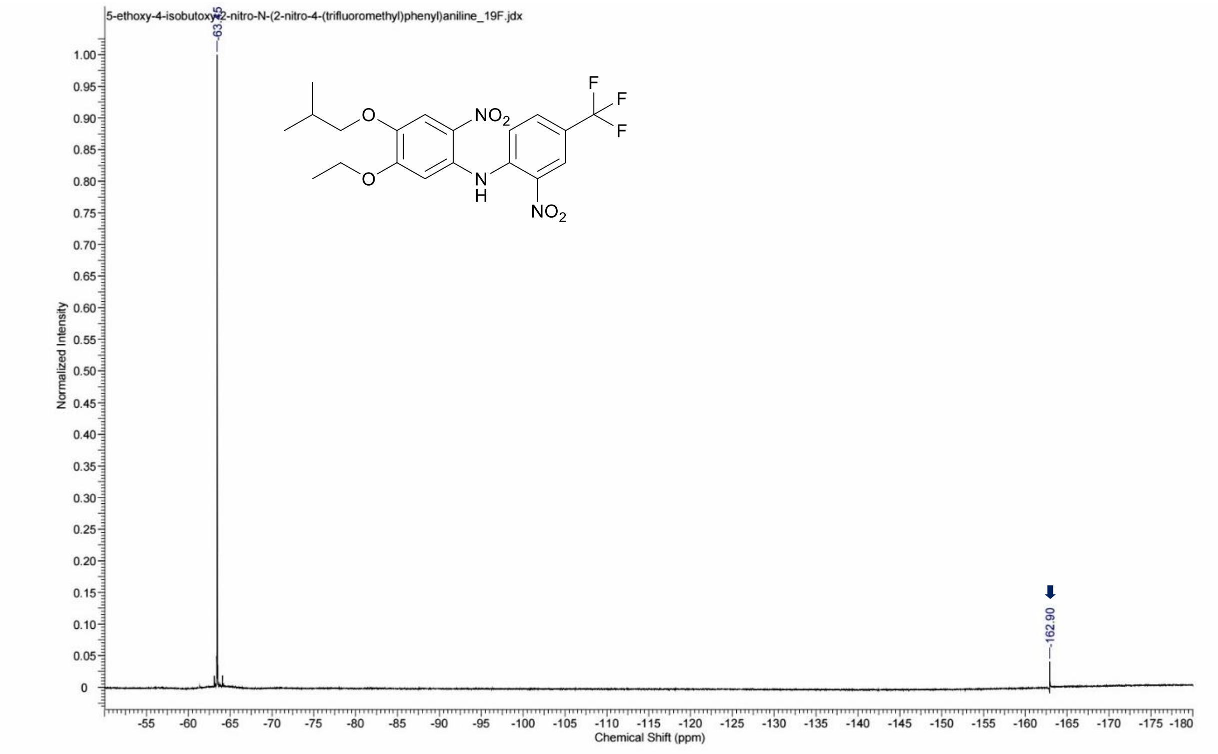

Figure S49. ^{19}F (CDCl$_3$, 282 MHz, δ ppm) NMR spectrum of **3c**.

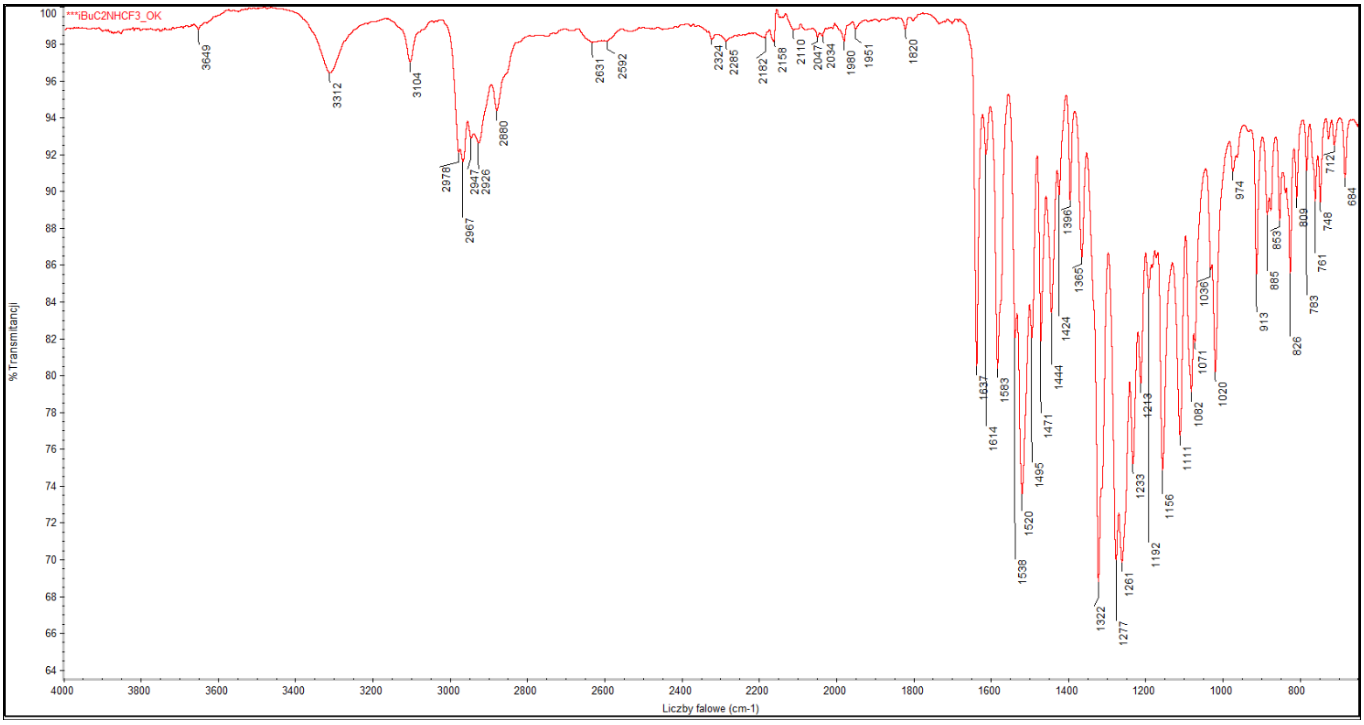

Figure S50. IR spectrum of **3c**.

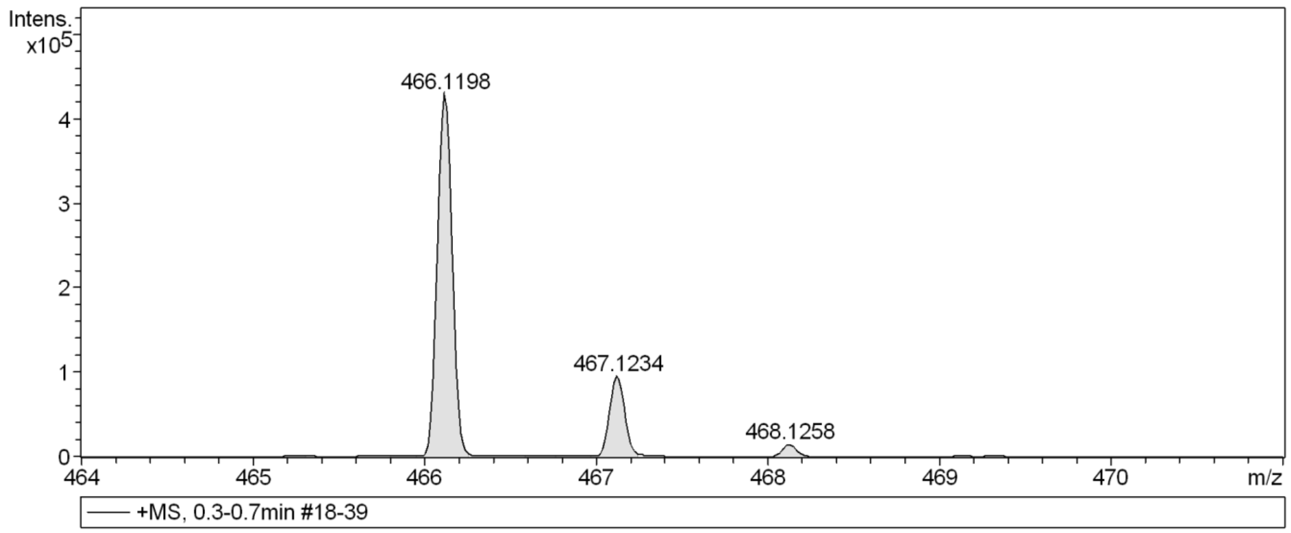

Figure S51. HRMS (ESI) spectrum of **3c**.

4-butoxy-5-isobutoxy-2-nitro-*N*-(2-nitro-4-(trifluoromethyl)phenyl)aniline (3d).

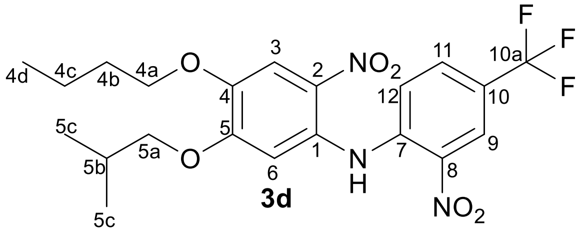

^{1}H NMR ($CDCl_3$, 300 MHz, δ ppm): 11.06 (s, 1H, H_{N-H}), 8.50 (d, $^{4}J_{H9-H11}$ = 2.17 Hz, 1H, H_9), 7.69 (s, 1H, H_3), 7.67 (dd, $^{3}J_{H11-H12}$ = 8.95 Hz, $^{4}J_{H9-H11}$ = 2.17 Hz, 1H, H_{11}), 7.53 (d, $^{3}J_{H11-H12}$ = 8.95 Hz, 1H, H_{12}), 6.93 (s, 1H, H_6), 4.08 (t, $^{3}J_{H4a-H4b}$ = 6.40 Hz, 2H, H_{4a}), 3.76 (d, $^{3}J_{H5a-H5b}$ = 6.55 Hz, 2H, H_{5a}), 2.17 (m, 1H, H_{5b}), 1.91 – 1.80 (m, 2H, H_{4b}), 1.62 – 1.48 (m, 2H, H_{4c}), 1.06 (d, $^{3}J_{H5b-H5c}$ = 6.80 Hz, 6H, H_{5c}), 1.02 (t, $^{3}J_{H4d-H4c}$ = 7.40 Hz, 3H, H_{4d}).

^{13}C{^{1}H} NMR ($CDCl_3$, 75 MHz, δ ppm): 155.7 (C_5), 146.4 (C_4), 142.4 (C_7), 136.2 (C_8), 133.8 (C_2), 131.9 (q, $^{3}J_{C-F}$ = 3 Hz, C_{11}), 130.6 (C_1), 125.5 (q, $^{3}J_{C-F}$ = 4 Hz C_9), 122.9 – 121.9 (m, C_{10}), 118.8 (C_{12}), 110.3 (C_3), 105.9 (C_6), 76.4 (C_{5a}), 70.1 (C_{4a}), 31.6 (C_{4b}), 28.9 (C_{5b}), 19.8 (C_{4c}), 19.7 (C_{5c}), 14.4 (C_{4d}), signal from C_{10a} is missing.

^{19}F NMR ($CDCl_3$, 282 MHz, δ ppm): –63.42 (s, 3F, F_{CF3}).

FT-IR (ATR, ν_{max}, (neat)/cm^{-1}): 3299, 3102, 2963, 2932, 2877, 1733, 1633, 1611, 1584, 1539, 1518, 1491, 1472, 1463, 1445, 1424, 1397, 1364, 1327, 1306, 1282, 1258, 1212, 1175, 1159, 1133, 1084, 1036, 1005, 968, 911, 901, 848, 839, 807, 757, 687.

HRMS (ESI) *m/z* Calculated for $C_{21}H_{25}N_3O_6F_3$ $[M+H]^+$, 472.1690; found: 472.1688.

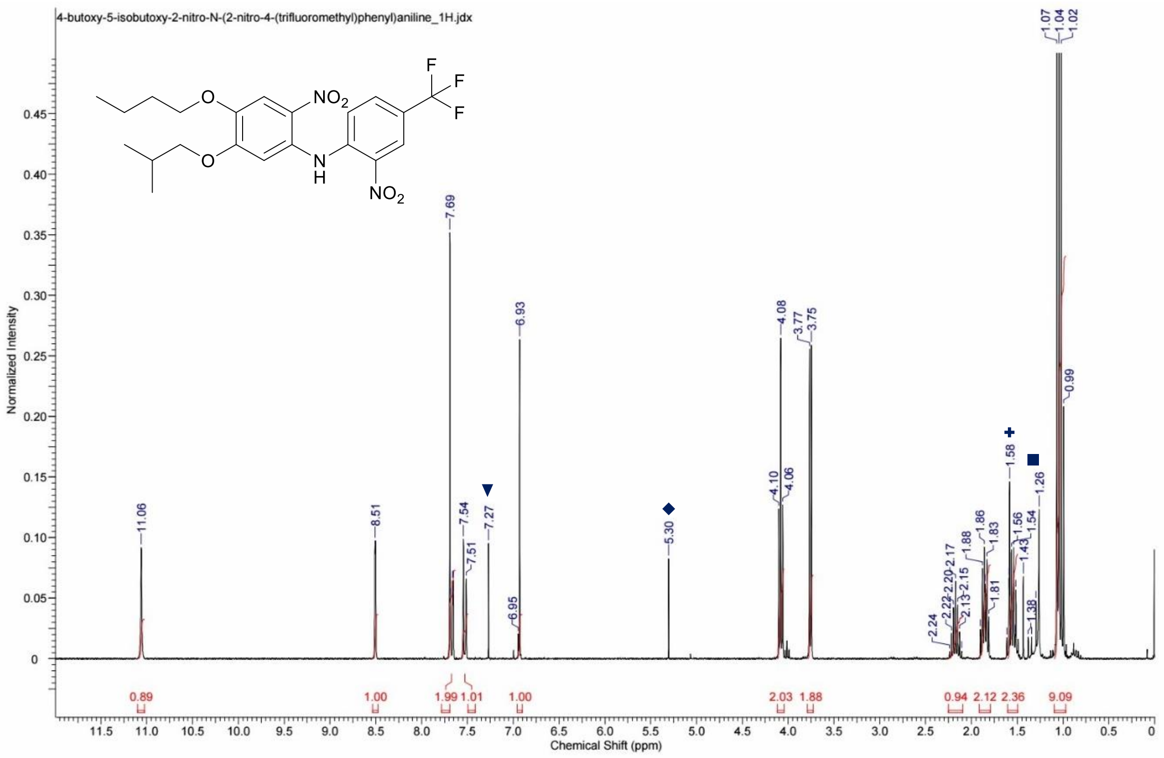

Figure S52. ^{1}H NMR ($CDCl_3$, 300 MHz, δ ppm) spectrum of **3d**.

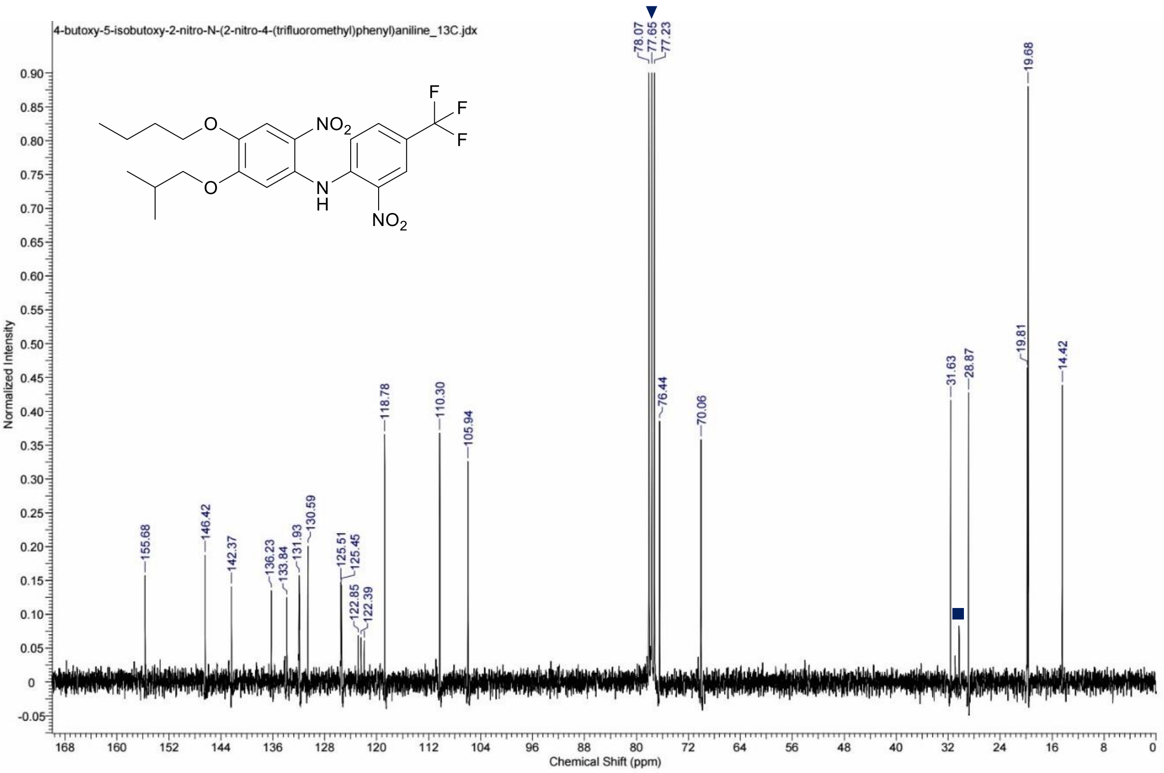

Figure S53. $^{13}C\{^{1}H\}$ NMR ($CDCl_3$, 75 MHz, δ ppm) NMR spectrum of **3d**.

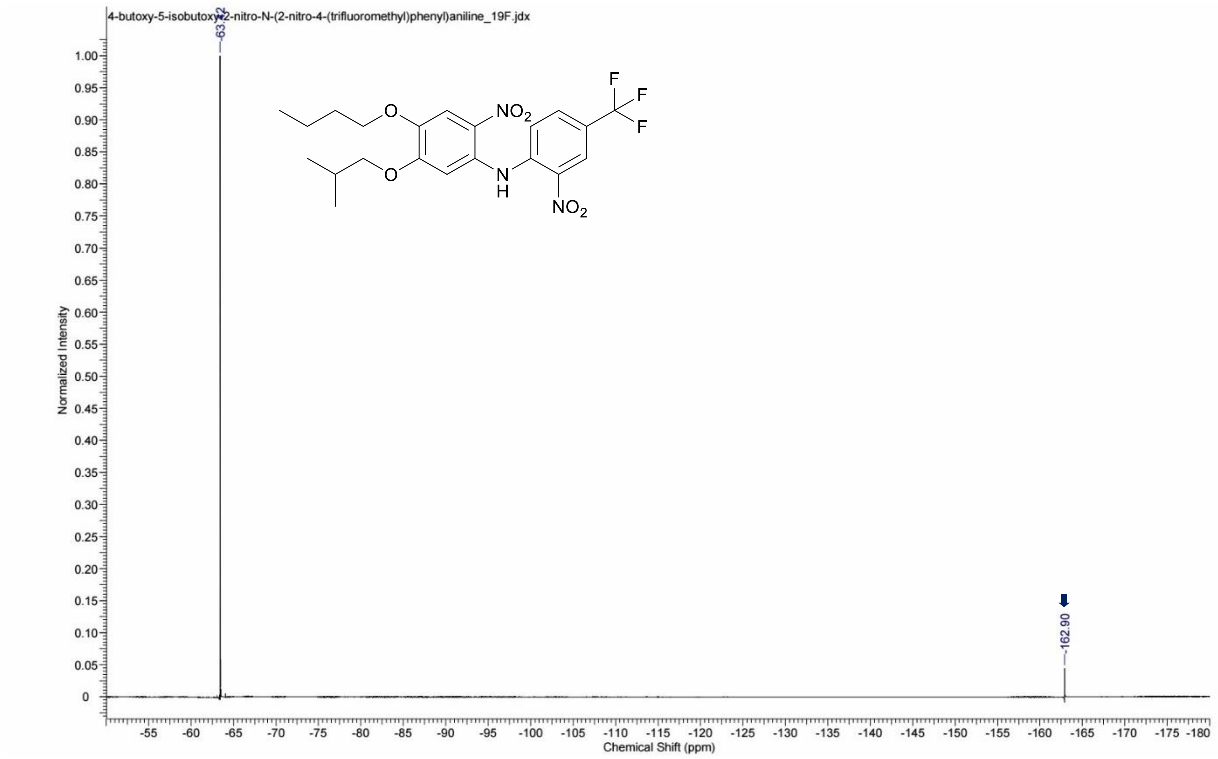

Figure S54. ^{19}F NMR ($CDCl_3$, 282 MHz, δ ppm) spectrum of **3d**.

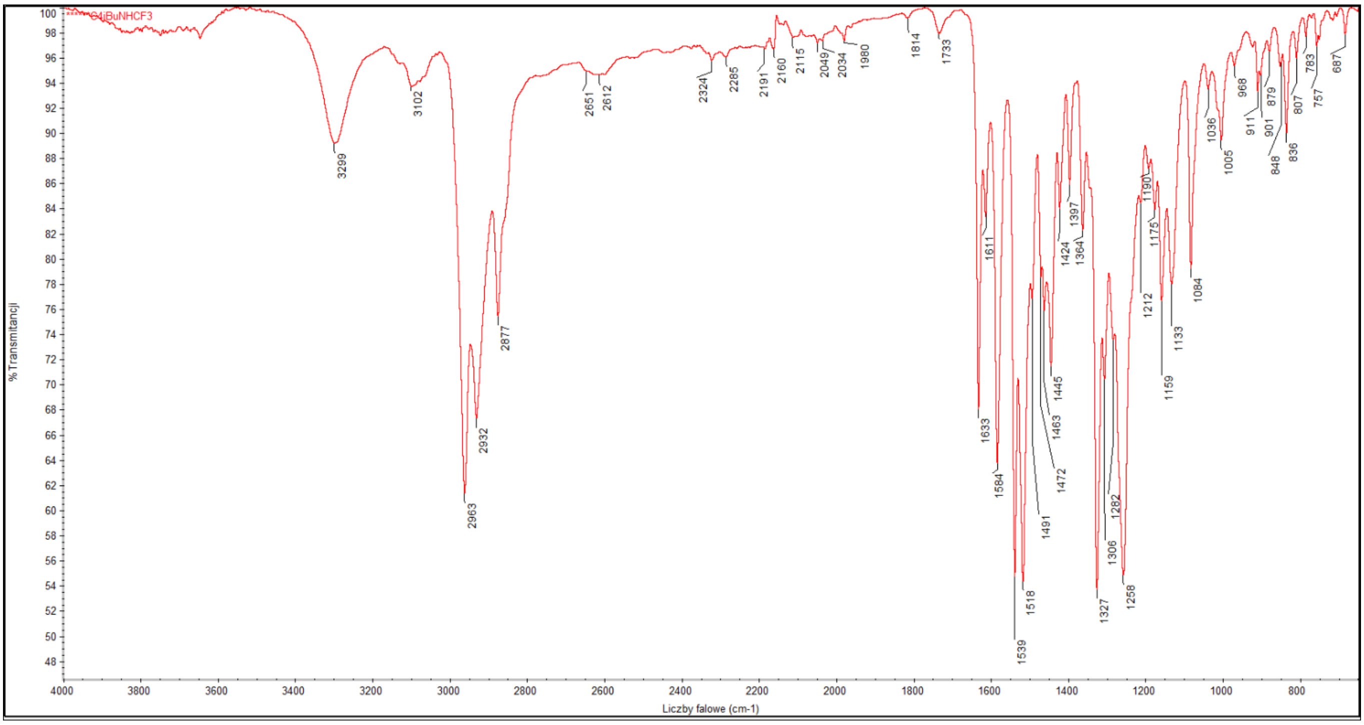

Figure S55. IR spectrum of **3d**.

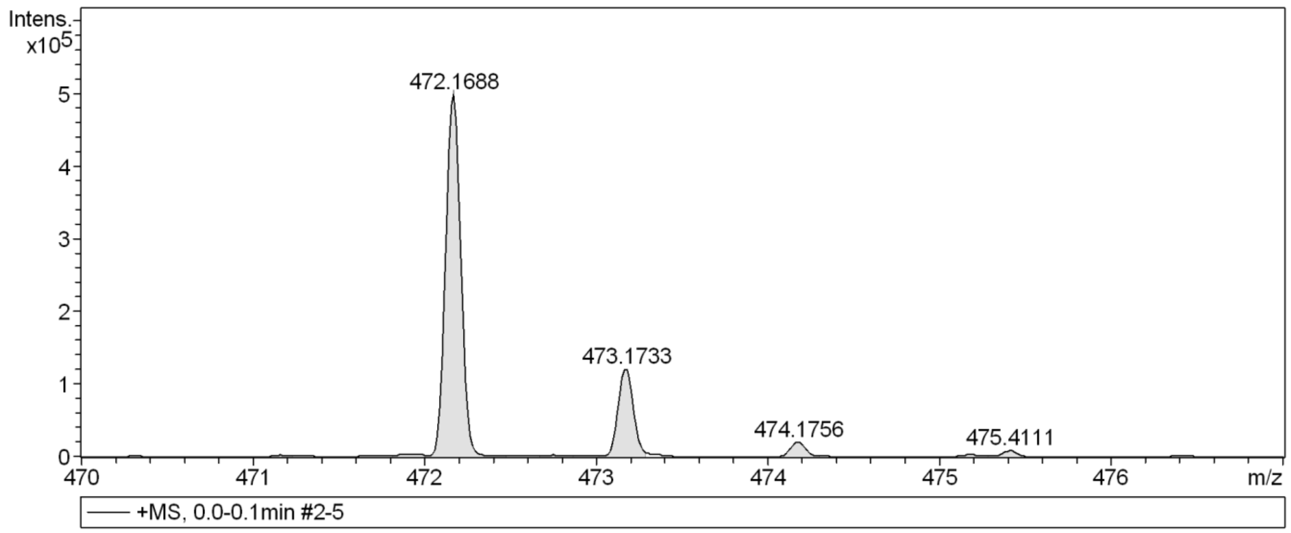

Figure S56. HRMS (ESI) spectrum of **3d**.

4-(hexyloxy)-5-isobutoxy-2-nitro-*N*-(2-nitro-4-(trifluoromethyl)phenyl)aniline (3e).

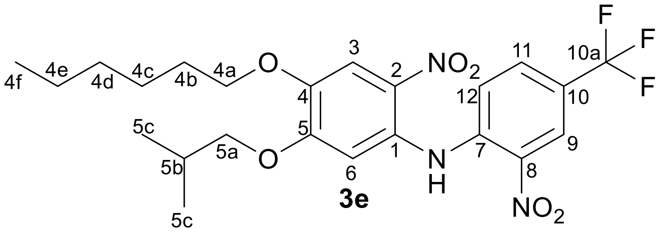

3e

^{1}H NMR ($CDCl_3$, 300 MHz, δ ppm): 11.07 (s, 1H, $H_{N–H}$), 8.51 (d, $^{4}J_{H9–H11}$ = 2.20 Hz, 1H, H_9), 7.69 (s, 1H, H_3), 7.66 (dd, $^{3}J_{H11–H12}$ = 8.90 Hz, $^{4}J_{H9–H11}$ = 2.20 Hz, 1H, H_{11}), 7.51 (d, $^{3}J_{H11–H12}$ = 8.90 Hz, 1H, H_{12}), 6.91 (s, 1H, H_6), 4.06 (t, $^{3}J_{H4a–H4b}$ = 6.48 Hz, 2H, H_{4a}), 3.74 (d, $^{3}J_{H5a–H5b}$ = 6.41 Hz, 2H, H_{5a}), 2.17 (m, 1H, H_{5b}), 1.91 – 1.80 (m, 2H, H_{4b}), 1.57 – 1.46 (m, 2H, H_{4c}), 1.41 – 1.32 (m, 4H, $H_{4d,4e}$), 1.05 (d, $^{3}J_{H5b–H5c}$ = 6.76 Hz, 6H, H_{5c}), 0.92 (t, $^{3}J_{H4e–H4f}$ = 6.88 Hz, 3H, H_{4f}).

$^{13}C\{^{1}H\}$ NMR ($CDCl_3$, 75 MHz, δ ppm): 155.7 (C_5), 146.4 (C_4), 142.4 (C_7), 136.3 (C_8), 133.9 (C_2), 131.9 (q, $^{3}J_{C–F}$ = 3 Hz, C_{11}), 130.6 (C_1), 125.5 (q, $^{3}J_{C–F}$ = 2 Hz C_9), 123.3 – 122.4 (m, C_{10}), 118.8 (C_{12}), 110.3 (C_3), 105.9 (C_6), 76.4 (C_{5a}), 70.3 (C_{4a}), 32.1 (C_{4d}), 29.6 (C_{4b}), 28.9 (C_{5b}), 26.2 (C_{4c}), 23.2 (C_{4e}), 19.7 (C_{5c}), 14.6 (C_{4f}), signal from C_{10a} is missing.

^{19}F NMR ($CDCl_3$, 282 MHz, δ ppm): –63.43 (s, 3F, F_{CF3}).

FT-IR (ATR, ν_{max}, (neat)/cm^{-1}): 3323, 3093, 2978, 2956, 2934, 2871, 2856, 1634, 1607, 1581, 1571, 1542, 1519, 1500, 1467, 1435, 1397, 1360, 1341, 1323, 1282, 1261, 1234, 1215, 1195, 1169, 1147, 1108, 1082, 1068, 1038, 1008, 986, 938, 917, 892, 874, 837, 825, 802, 781, 763, 741, 683.

HRMS (ESI) *m/z* Calculated for $C_{23}H_{29}N_3O_6F_3$ $[M+H]^+$, 500.2003; found: 500.2005.

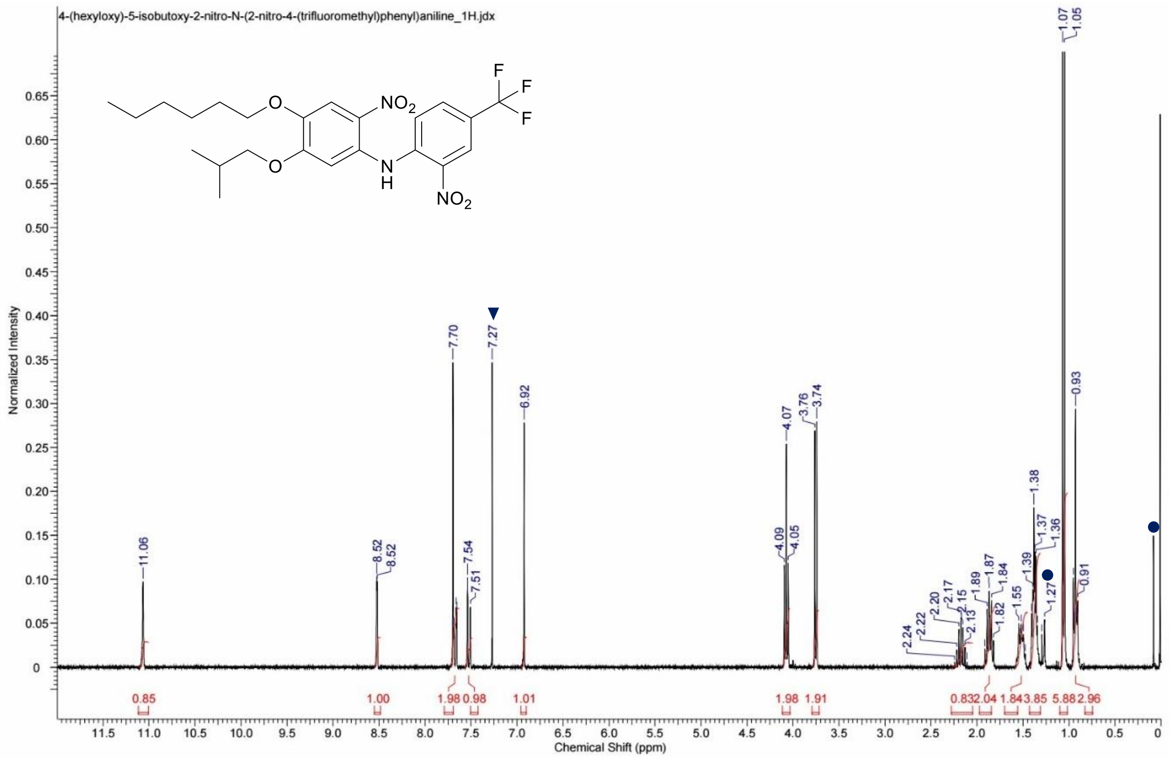

Figure S57. ^{1}H NMR ($CDCl_3$, 300 MHz, δ ppm) spectrum of **3e**.

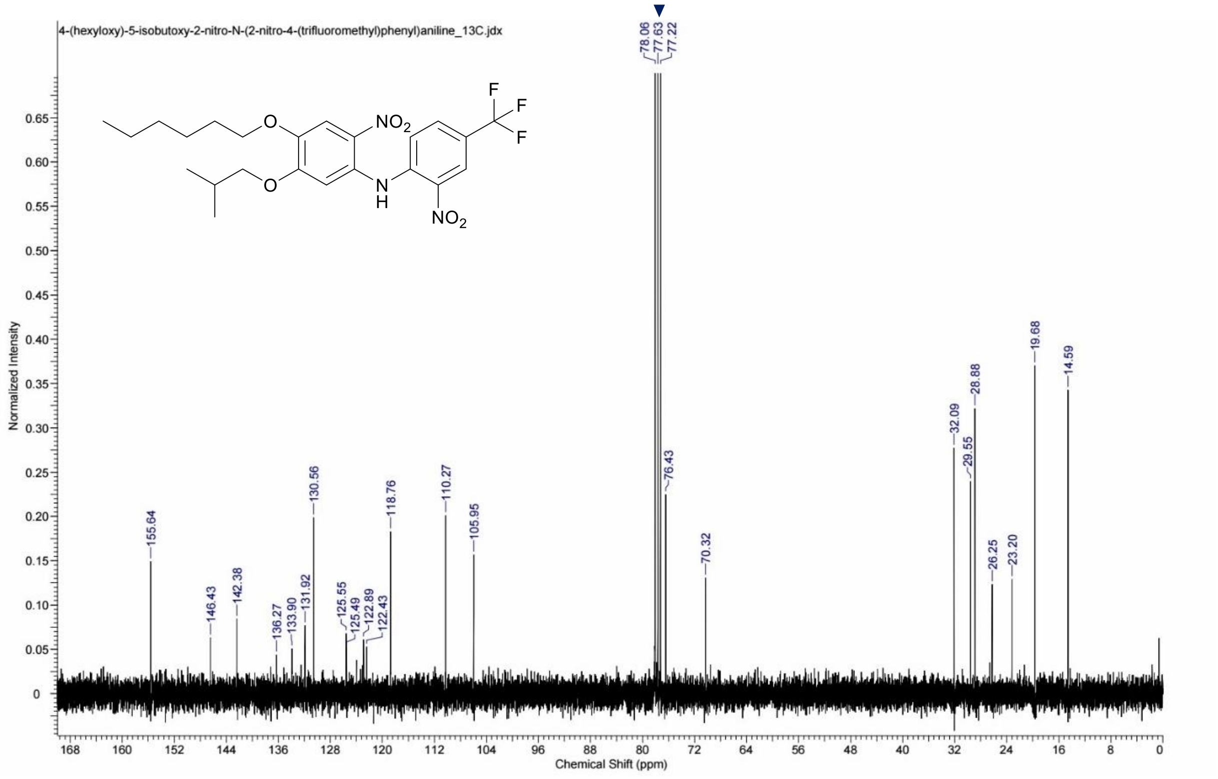

Figure S58. $^{13}C\{^{1}H\}$ NMR ($CDCl_3$, 75 MHz, δ ppm) NMR spectrum of **3e**.

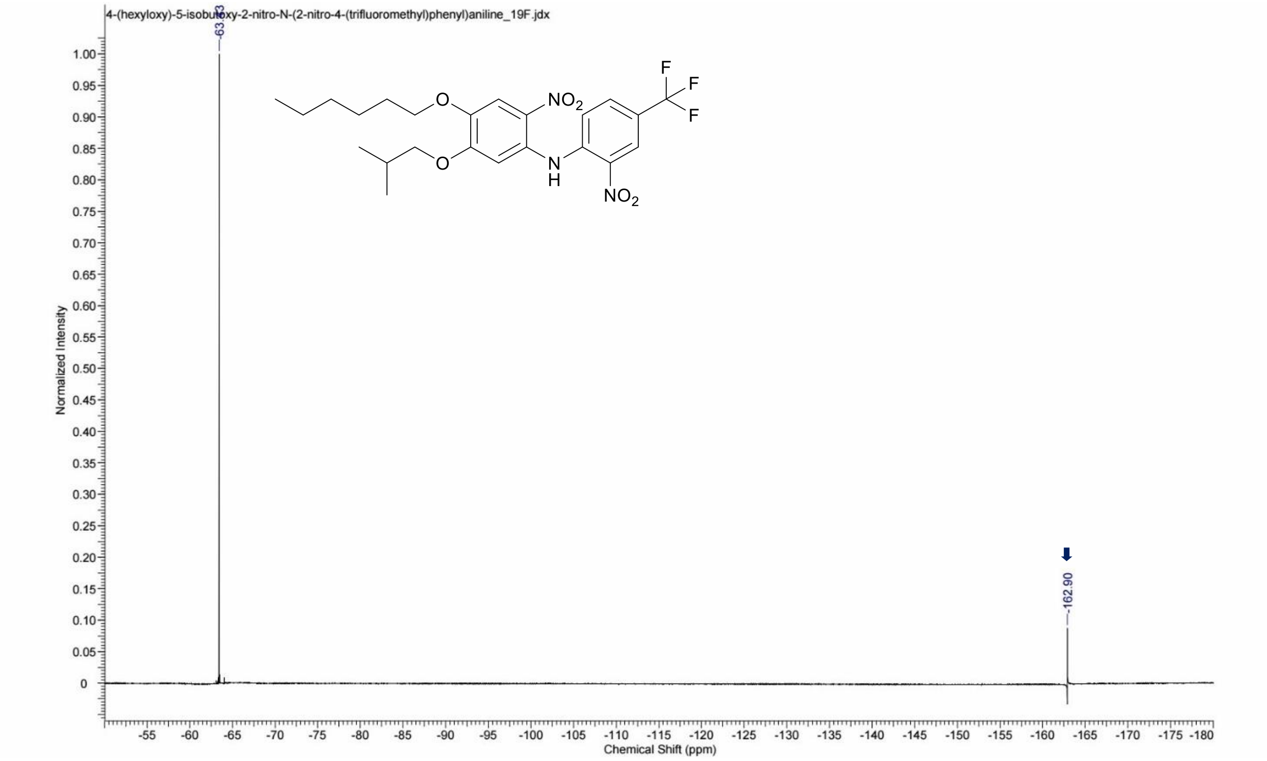

Figure S59. ^{19}F NMR ($CDCl_3$, 282 MHz, δ ppm) spectrum of **3e**.

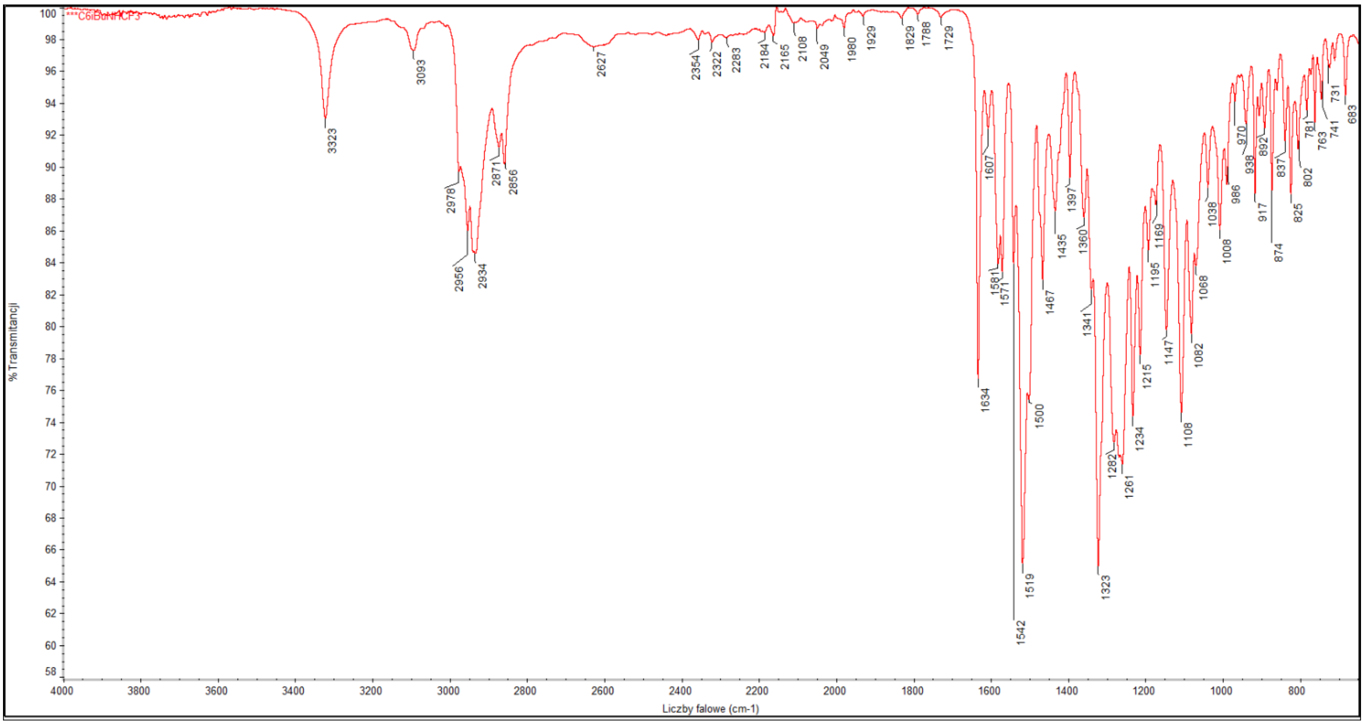

Figure S60. IR spectrum of **3e**.

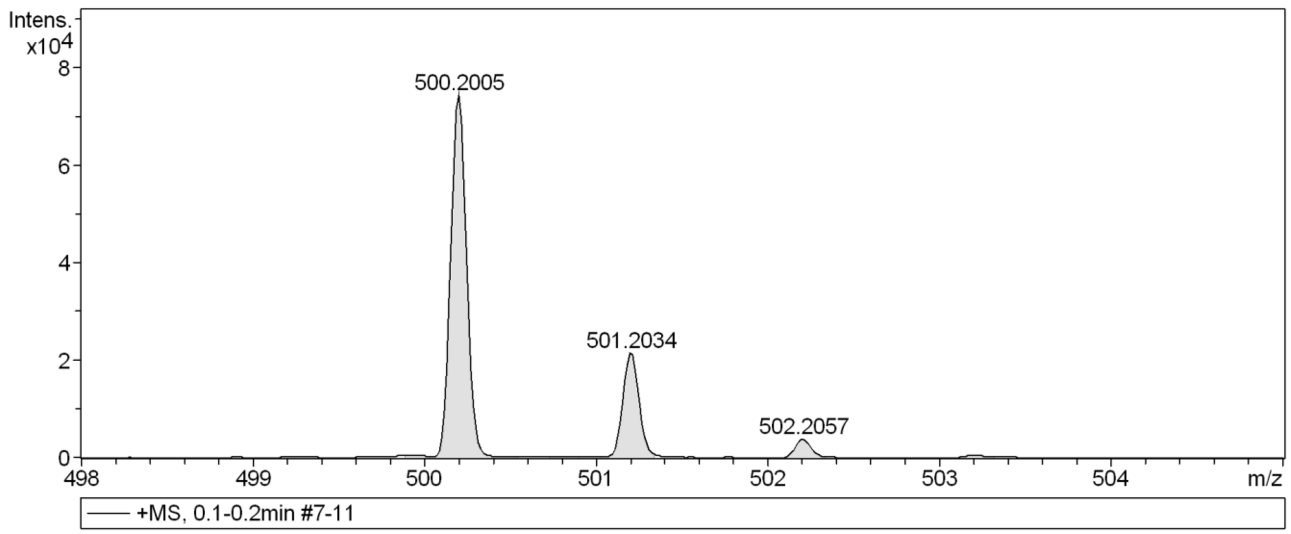

Figure S61. HRMS (ESI) spectrum of **3e**.

5-isobutoxy-2-nitro-*N*-(2-nitro-4-(trifluoromethyl)phenyl)-4-(octyloxy)aniline (3f).

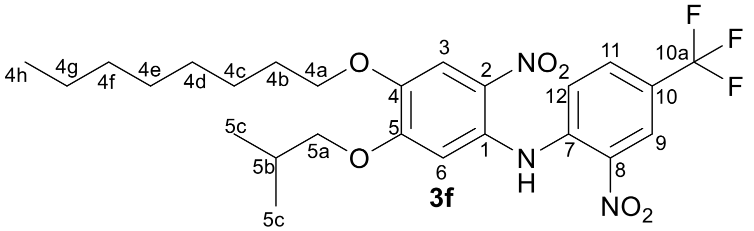

3f

^{1}H NMR ($CDCl_3$, 300 MHz, δ ppm): 11.06 (s, 1H, H_{N-H}), 8.50 (d, $^{4}J_{H9-H11}$ = 2.18 Hz, 1H, H_9), 7.68 (s, 1H, H_3), 7.67 (dd, $^{3}J_{H11-H12}$ = 8.90 Hz, $^{4}J_{H9-H11}$ = 2.18 Hz, 1H, H_{11}), 7.53 (d, $^{3}J_{H11-H12}$ = 8.90 Hz, 1H, H_{12}), 6.94 (s, 1H, H_6), 4.07 (t, $^{3}J_{4a-4b}$ = 6.53 Hz, 2H, H_{4a}), 3.76 (d, $^{3}J_{H5a-H5b}$ = 6.53 Hz, 2H, H_{5a}), 2.18 (m, 1H, H_{5b}), 1.92 – 1.81 (m, 2H, H_{4b}), 1.57 – 1.45 (m, 2H, C_{4c}), 1.44 – 1.24 (m, 8H, $H_{4d,4e,4f,4g}$), 1.06 (d, $^{3}J_{H5b-H5c}$ = 6.91 Hz, 6H, H_{5c}), 0.90 (t, $^{3}J_{H4g-H4h}$ = 6.91 Hz, 3H, H_{4h}).

^{13}C{^{1}H} NMR ($CDCl_3$, 75 MHz, δ ppm): 155.7 (C_5), 146.4 (C_4), 142.4 (C_7), 136.2 (C_8), 133.8 (C_2), 132.0 (q, $^{3}J_{C-F}$ = 3 Hz, C_{11}), 130.6 (C_1), 125.5 (q, $^{3}J_{C-F}$ = 4 Hz C_9), 122.9 – 121.9 (m, C_{10}), 118.8 (C_{12}), 110.3 (C_3), 105.9 (C_6), 76.4 (C_{5a}), 70.3 (C_{4a}), 32.4 (C_{4f}), 29.9 – 29.8 (m, 2C, $C_{4b,4d}$), 29.6 (C_{4e}), 28.9 (C_{5b}), 26.6 (C_{4c}), 23.3 (C_{4g}), 19.7 (C_{5c}), 14.7 (C_{4h}), signal from C_{10a} is missing.

^{19}F NMR ($CDCl_3$, 282 MHz, δ ppm): –63.42 (s, 3F, F_{CF3}).

FT-IR (ATR, ν_{max}, (neat)/cm^{-1}): 3320, 3102, 2954, 2926, 2869, 2856, 1634, 1585, 1572, 1543, 1520, 1471, 1436, 1398, 1358, 1339, 1324, 1282, 1235, 1217, 1199, 1173, 1147, 1105, 1086, 1066, 1010, 988, 968, 917, 869, 874, 835, 822, 816, 783, 763, 746, 683.

HRMS (ESI) *m/z* Calculated for $C_{25}H_{32}N_3O_6F_3Na$ [M+Na]$^{+}$, 550.2136; found: 550.2132.

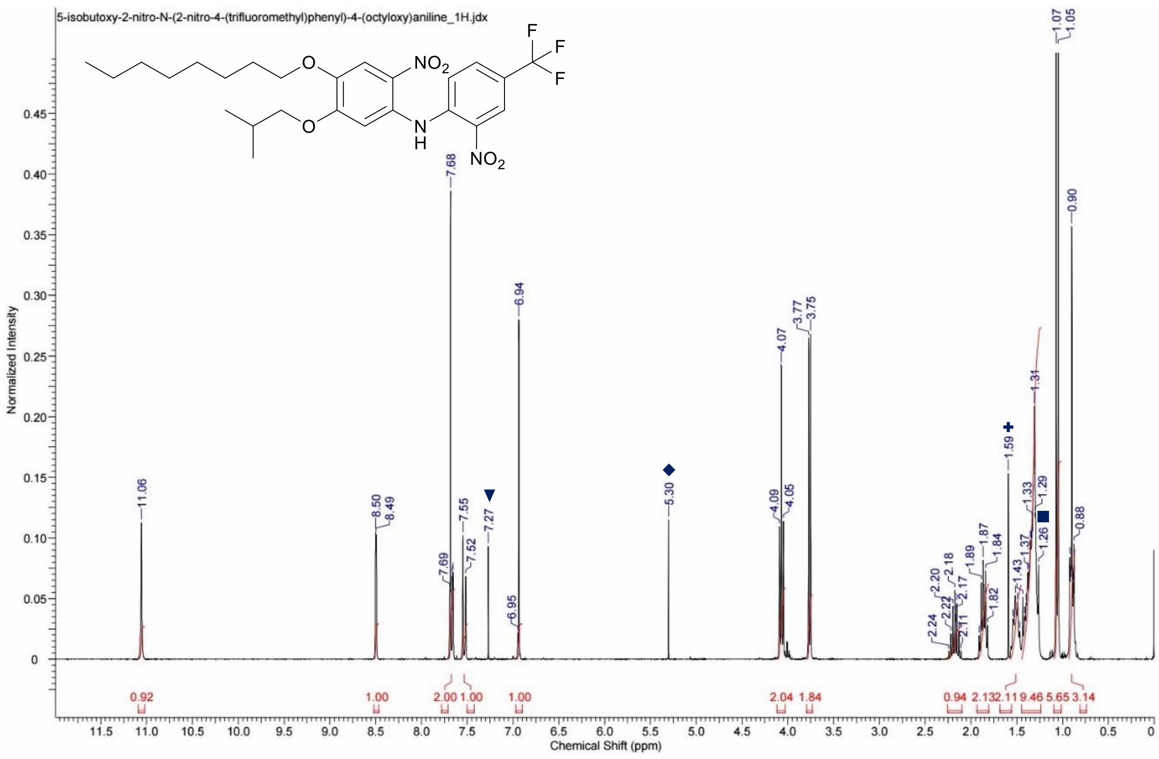

Figure S62. ^{1}H NMR ($CDCl_3$, 300 MHz, δ ppm) spectrum of **3f**.

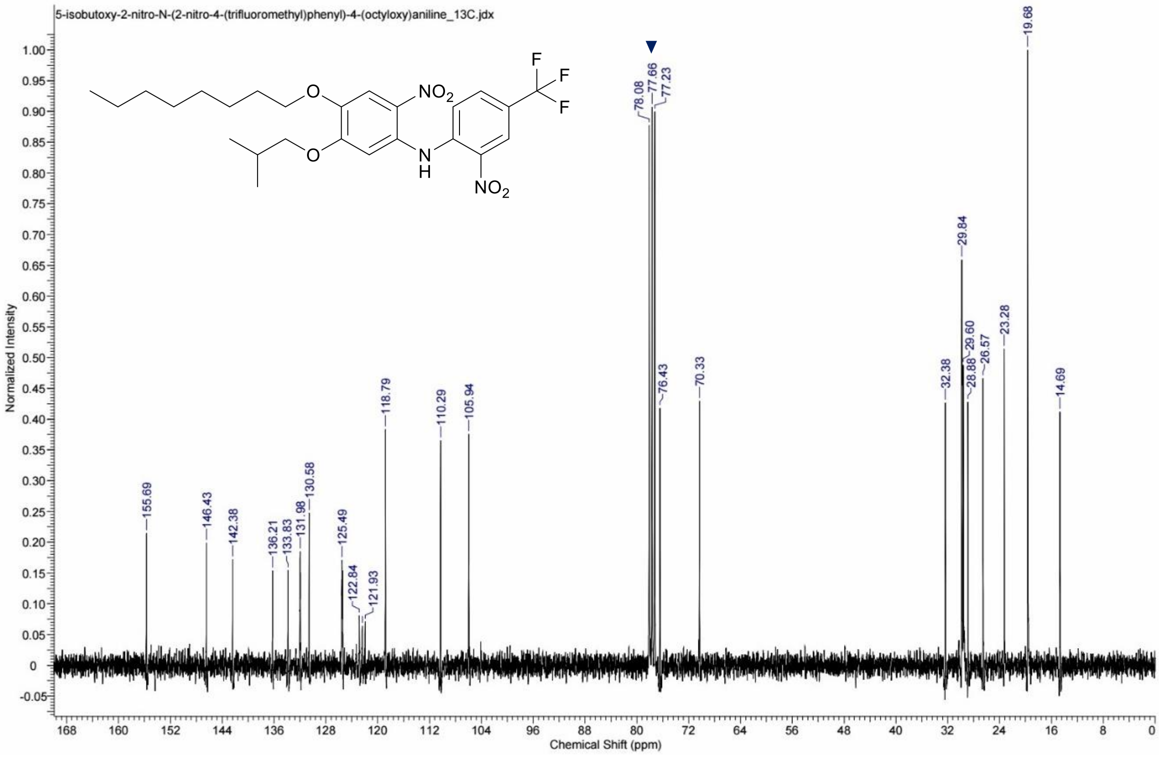

Figure S63. $^{13}C\{^{1}H\}$ NMR ($CDCl_3$, 75 MHz, δ ppm) NMR spectrum of **3f**.

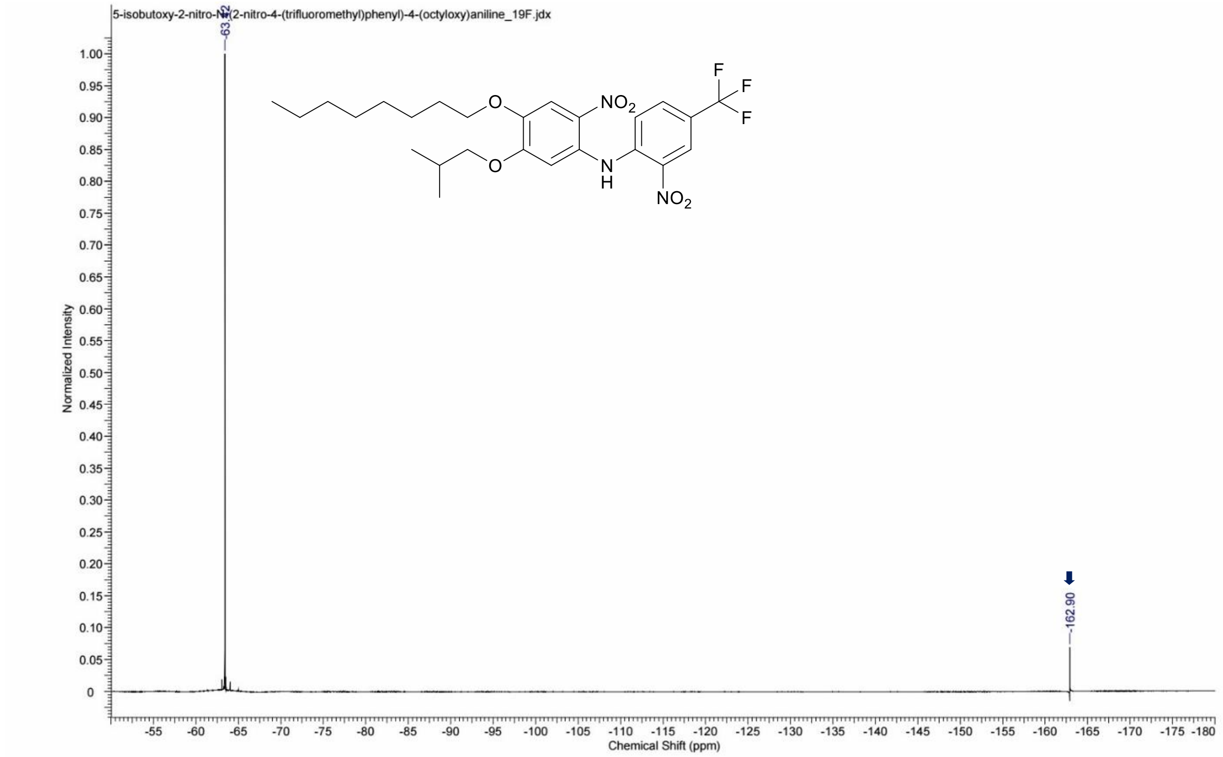

Figure S64. ^{19}F NMR ($CDCl_3$, 282 MHz, δ ppm) spectrum of **3f**.

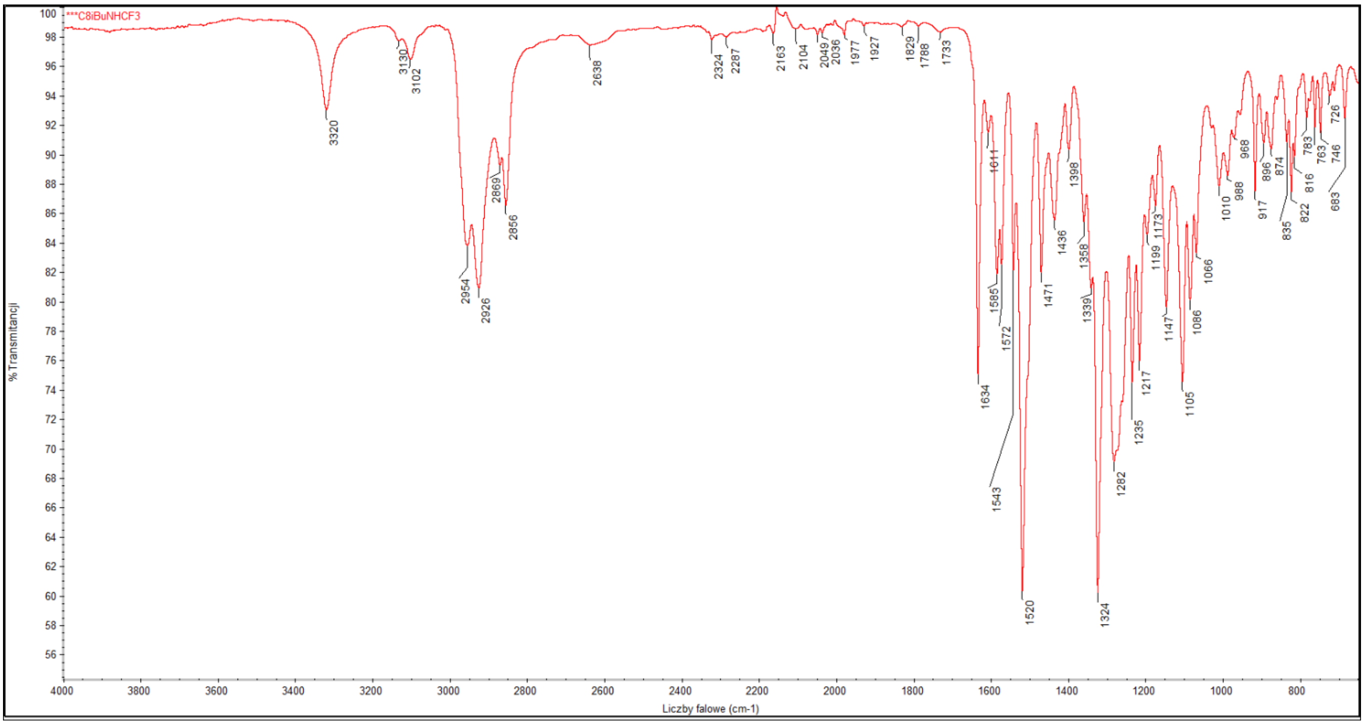

Figure S65. IR spectrum of **3f**.

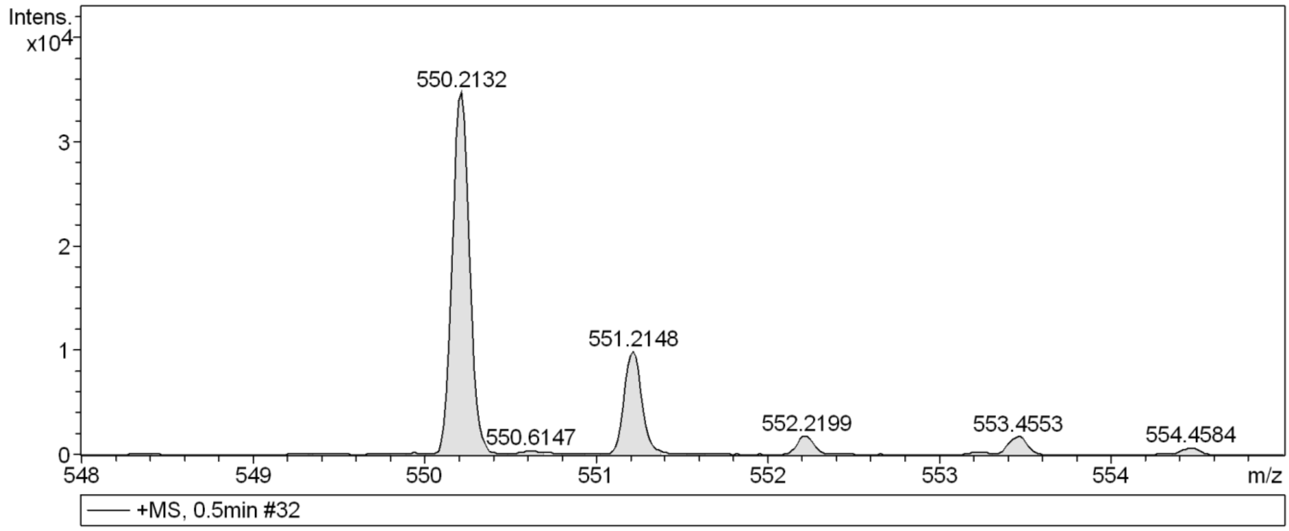

Figure S66. HRMS (ESI) spectrum of **3f**.

4-(decyloxy)-5-isobutoxy-2-nitro-*N*-(2-nitro-4-(trifluoromethyl)phenyl)aniline (3g).

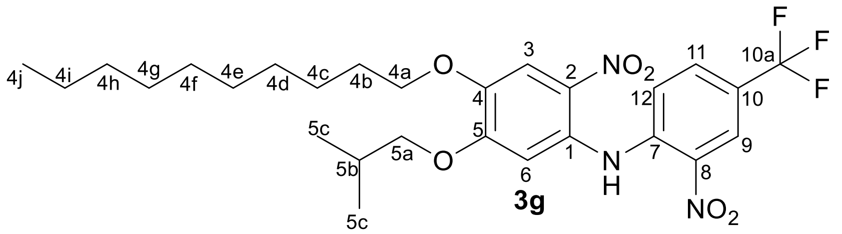

3g

^{1}H NMR ($CDCl_3$, 300 MHz, δ ppm): 11.06 (s, 1H, H_{N-H}), 8.51 (d, $^4J_{H9-H11}$ = 2.05 Hz, 1H, H_9), 7.69 (s, 1H, H_3), 7.67 (dd, $^3J_{H11-H12}$ = 8.97 Hz, $^4J_{H9-H11}$ = 2.05 Hz, 1H, H_{11}), 7.52 (d, $^3J_{H11-H12}$ = 8.97 Hz, 1H, H_{12}), 6.92 (s, 1H, H_6), 4.07 (t, $^3J_{H4a-H4b}$ = 6.42 Hz, 2H, H_{4a}), 3.75 (d, $^3J_{H5a-H5b}$ = 6.52 Hz, 2H, H_{5a}), 2.18 (m, 1H, H_{5b}), 1.92 – 1.81 (m, 2H, H_{4b}), 1.57 – 1.45 (m, 2H, C_{4c}), 1.43 – 1.23 (m, 12H, $H_{4d,4e,4f,4g,4h,4i}$), 1.06 (d, $^3J_{H5b-H5c}$ = 6.66 Hz, 6H, H_{5c}), 0.89 (t, $^3J_{4Hi-4Hj}$ = 6.90 Hz, 3H, H_{4j}).

^{13}C{^{1}H} NMR ($CDCl_3$, 75 MHz, δ ppm): 155.6 (C_5), 146.5 (C_4), 142.4 (C_7), 136.3 (C_8), 133.9 (C_2), 132.0 (q, $^3J_{C-F}$ = 3 Hz, C_{11}), 130.6 (C_1), 125.5 (q, $^3J_{C-F}$ = 3 Hz C_9), 122.8 – 121.9 (m, C_{10}), 118.8 (C_{12}), 110.3 (C_3), 106.0 (C_6), 76.4 (C_{5a}), 70.3 (C_{4a}), 32.5 (C_{4h}), 30.2 – 29.8 (m, 4C, $C_{4b,4d,4e,4f}$), 29.6 (C_{4g}), 28.9 (C_{5b}), 26.6 (C_{4c}), 23.3 (C_{4i}), 19.7 (C_{5c}), 14.7 (C_{4j}), signal from C_{10a} is missing.

^{19}F NMR ($CDCl_3$, 282 MHz, δ ppm): –63.43 (s, 3F, F_{CF3}).

FT-IR (ATR, ν_{max}, (neat)/cm^{-1}): 3317, 3096, 2952, 2922, 2873, 2855, 1634, 1609, 1584, 1570, 1542, 1520, 1472, 1435, 1404, 1356, 1324, 1282, 1235, 1216, 1195, 1175, 1147, 1109, 1082, 1066, 1042, 1007, 975, 917, 894, 876, 837, 823, 816, 783, 759.

HRMS (ESI) *m/z* Calculated for $C_{27}H_{36}N_3O_6F_3Na$ [M+Na]$^+$, 578.2449; found: 578.2449.

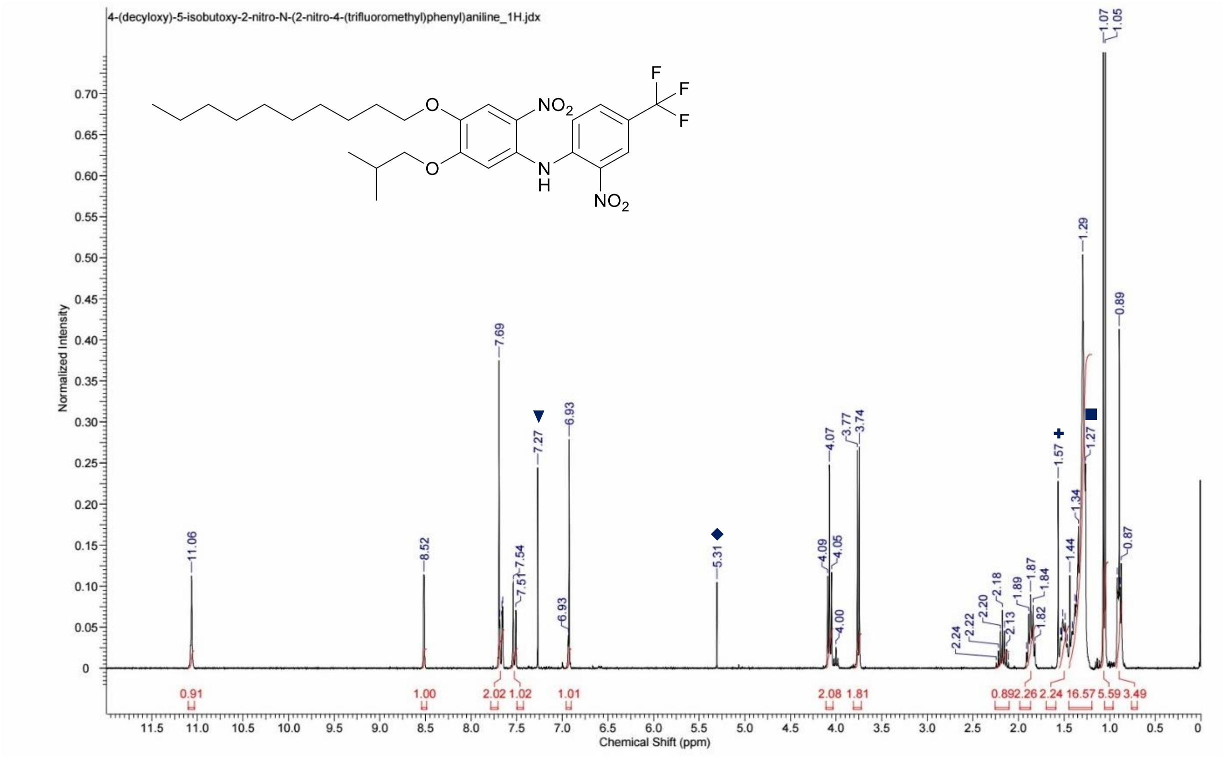

Figure S67. ^{1}H NMR ($CDCl_3$, 300 MHz, δ ppm) spectrum of **3g**.

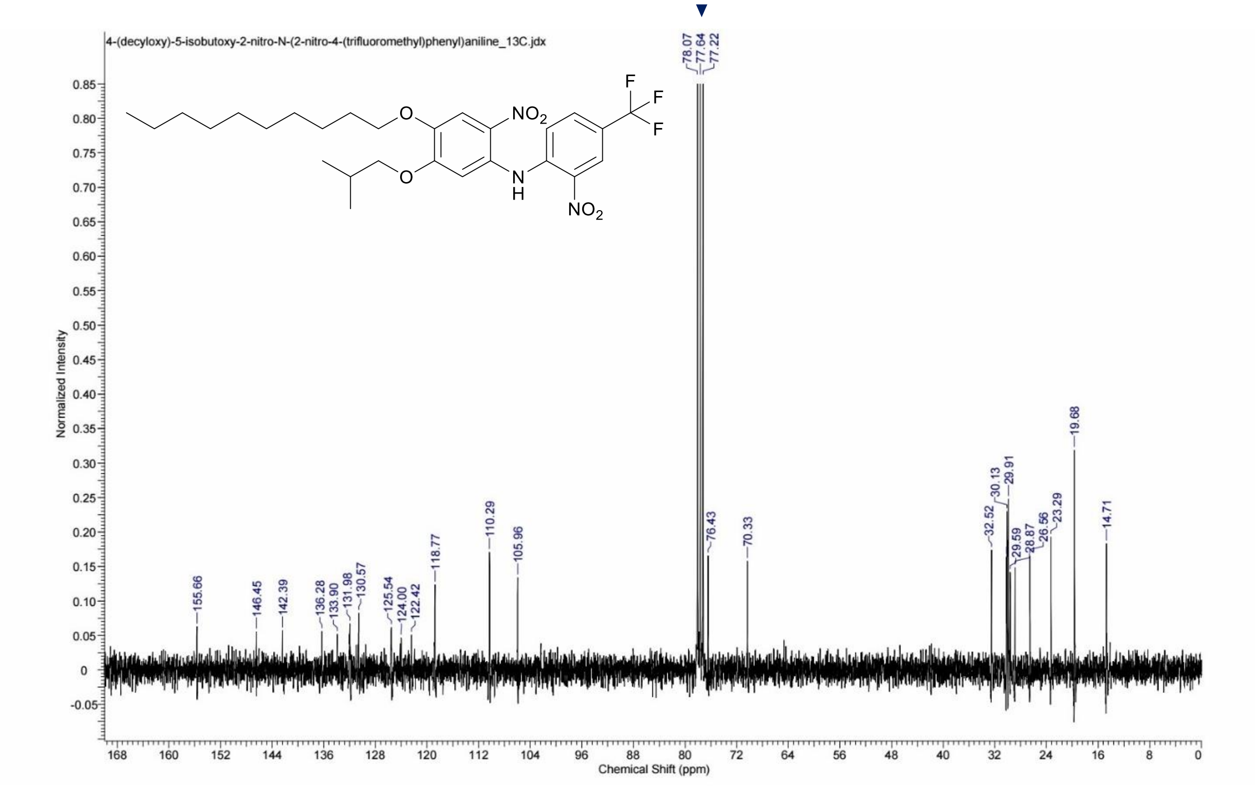

Figure S68. $^{13}C\{^{1}H\}$ NMR ($CDCl_3$, 75 MHz, δ ppm) NMR spectrum of **3g**.

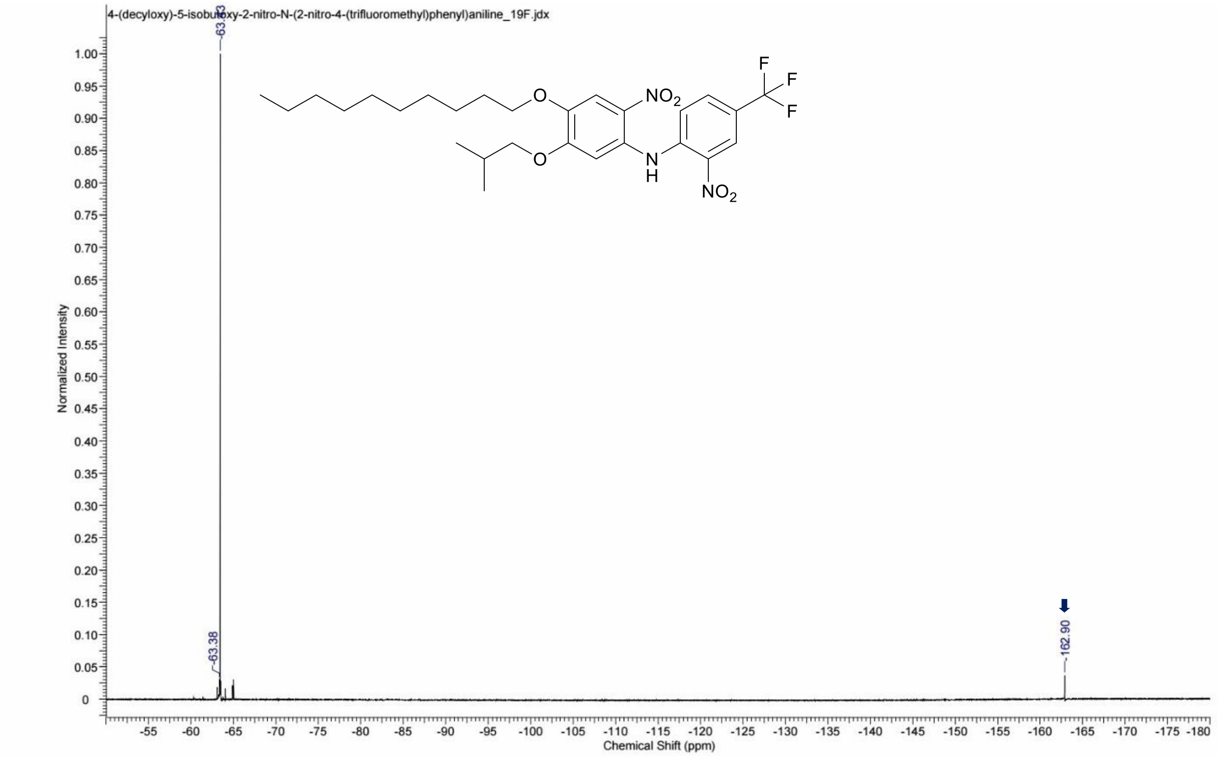

Figure S69. ^{19}F NMR ($CDCl_3$, 282 MHz, δ ppm) spectrum of **3g**.

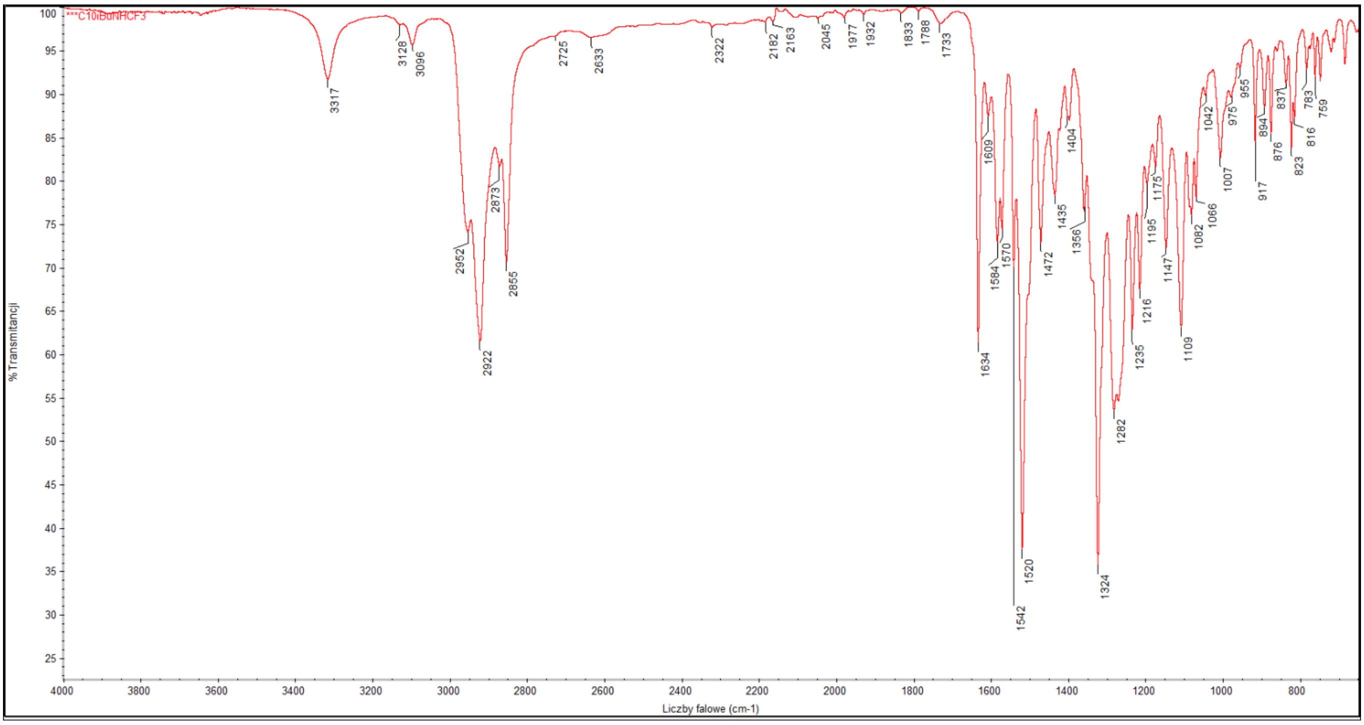

Figure S70. IR spectrum of **3g**.

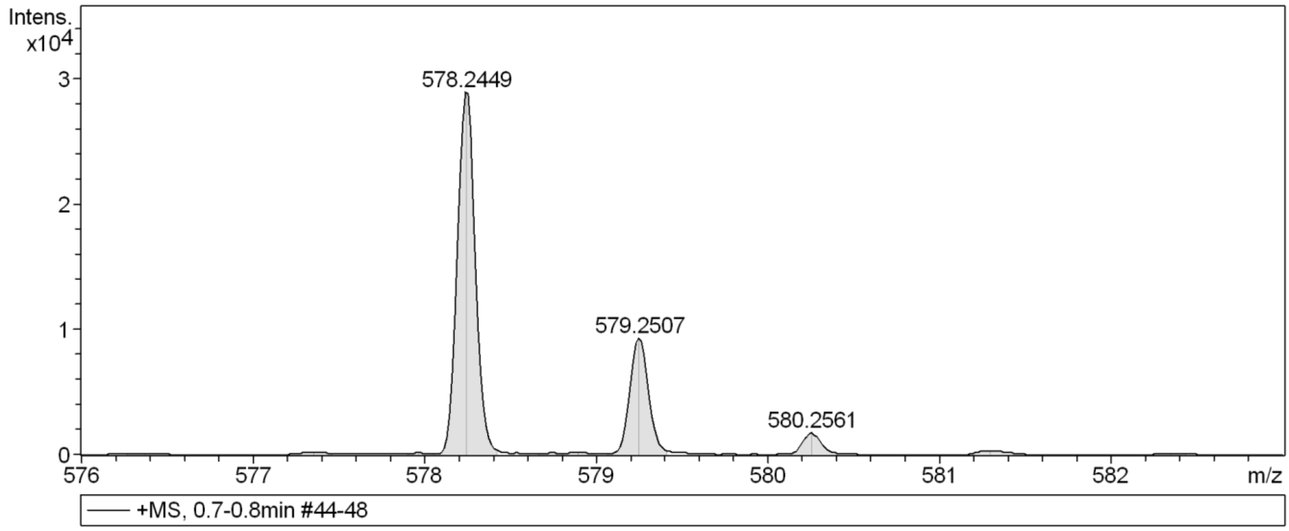

Figure S71. HRMS (ESI) spectrum of **3g**.

N-(4,5-bis(hexyloxy)-2-nitrophenyl)-4,5-dimethoxy-2-nitroaniline (4)

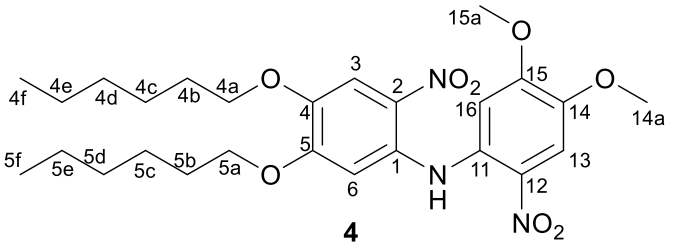

4

^{1}H NMR ($CDCl_3$, 300 MHz, δ ppm): 11.13 (s, 1H, $H_{N–H}$), 7.68 and 7.67 (s, 2x1H, $H_{3,13}$), 6.93 and 6.92 (s, 2x1H, $H_{6,16}$), 4.03 (t, $^3J_{H4a–H4b}$ = 6.60 Hz, 2H, H_{4a}), 3.94 (t, $^3J_{H5a–H5b}$ = 6.60 Hz, 2H, H_{5a}), 3.94 (s, 3H, H_{14a}), 3.85 (s, 3H, H_{15a}), 1.90 – 1.77 (m, 4H, $H_{4b,5b}$), 1.55 – 1.41 (m, 4H, $H_{4c,5c}$), 1.41 – 1.28 (m, 8H, $H_{4d,4e,5d,5e}$), 0.95 – 0.85 (m, 6H, $H_{4f,5f}$).

^{13}C{^{1}H} NMR ($CDCl_3$, 75 MHz, δ ppm): 155.9 and 155.7 ($C_{14,15}$), 144.6 and 144.5 ($C_{4,5}$), 134.8 and 133.9 ($C_{2,12}$), 131.6 and 131.2 ($C_{1,11}$), 110.2 and 108.6 ($C_{3,13}$), 103.2 and 101.8 ($C_{6,16}$), 70.3 and 70.2 ($C_{4a,5a}$), 57.1 ($C_{14a,15a}$), 32.1 and 32.0 ($C_{4d,5d}$), 29.6 and 29.4 ($C_{4b,5b}$), 26.3 and 26.2 ($C_{4c,5c}$), 23.2 and 23.1 ($C_{4e,5e}$), 14.6 and 14.5 ($C_{4f,5f}$).

FT-IR (ATR, ν_{max}, (neat)/cm^{-1}): 3462, 3335, 3284, 3104, 2952, 2928, 2869, 2856, 1622, 1579, 1506, 1464, 1405, 1391, 1371, 1318, 1253, 1227, 1186, 1078, 1067, 1026, 995, 950, 926, 897, 859, 848, 820, 799, 779, 755.

HRMS (ESI) *m/z* Calculated for $C_{26}H_{38}N_3O_8$ [M+H]$^+$, 520.2654; found: 520.2655.

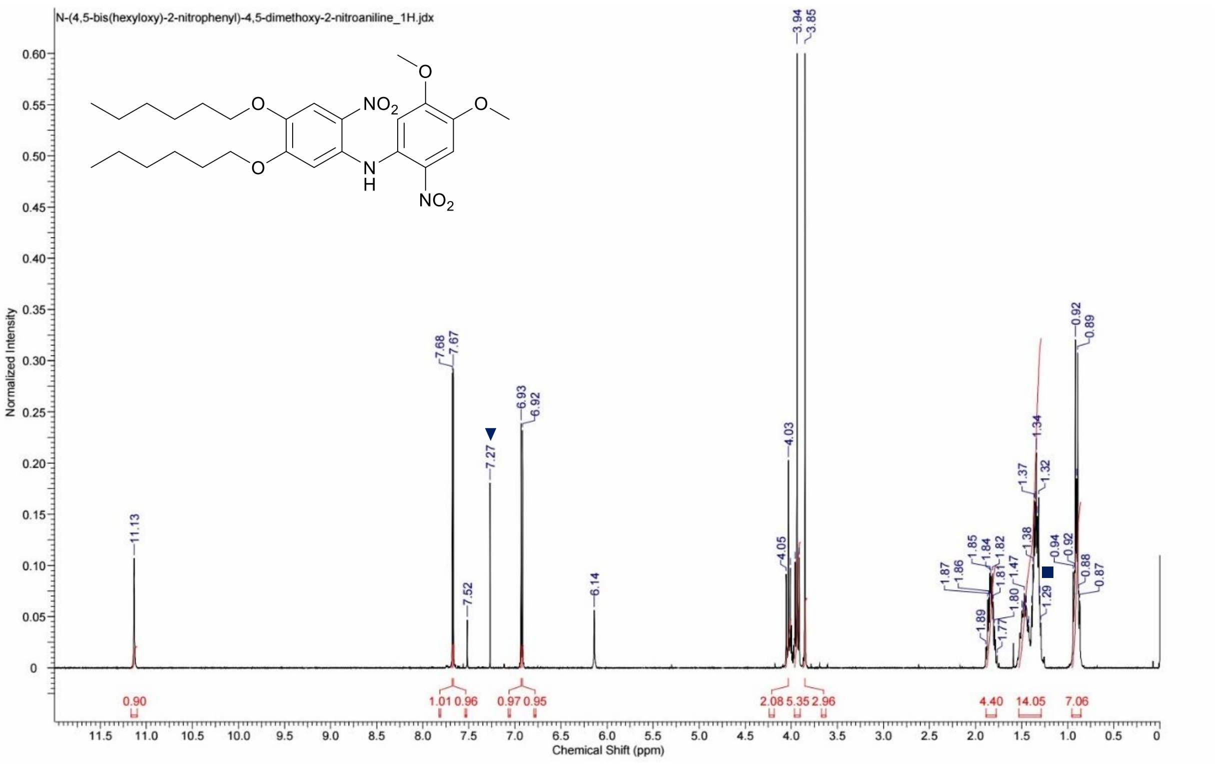

Figure S72. ^{1}H NMR ($CDCl_3$, 300 MHz, δ ppm) spectrum of **4**.

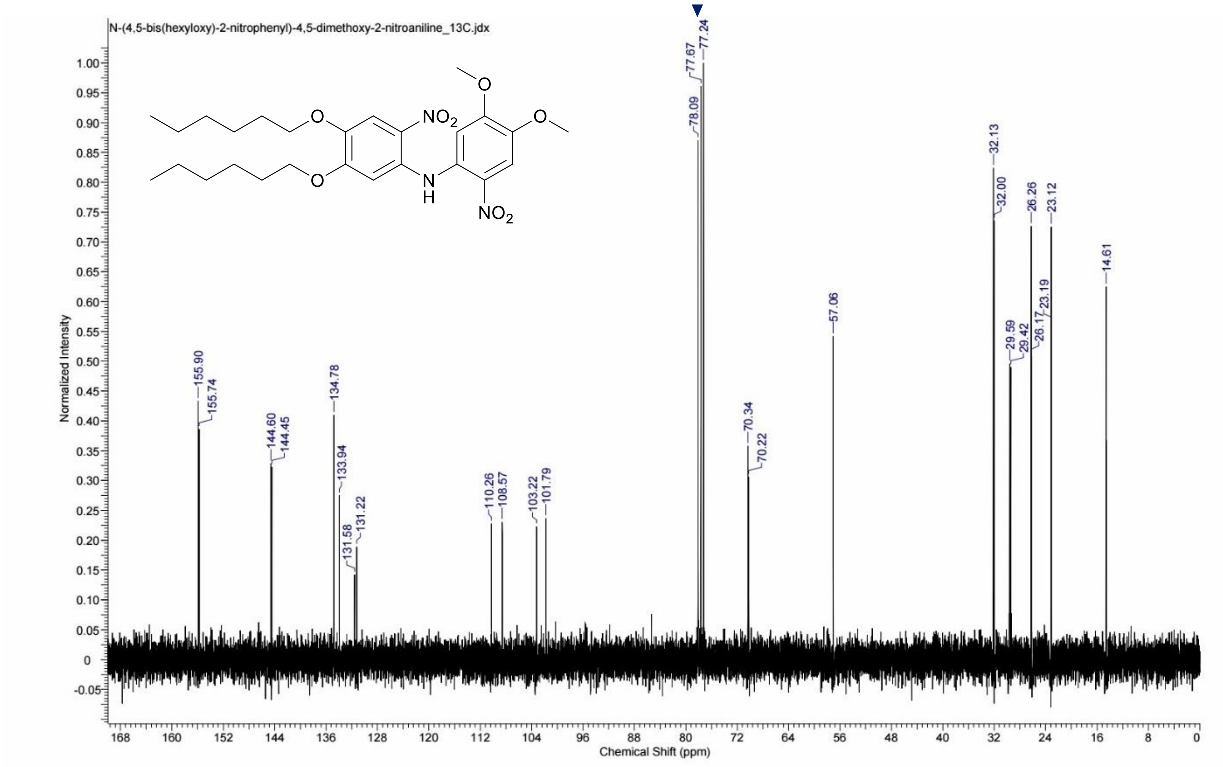

Figure S73. $^{13}C\{^{1}H\}$ NMR ($CDCl_3$, 75 MHz, δ ppm) NMR spectrum of **4**.

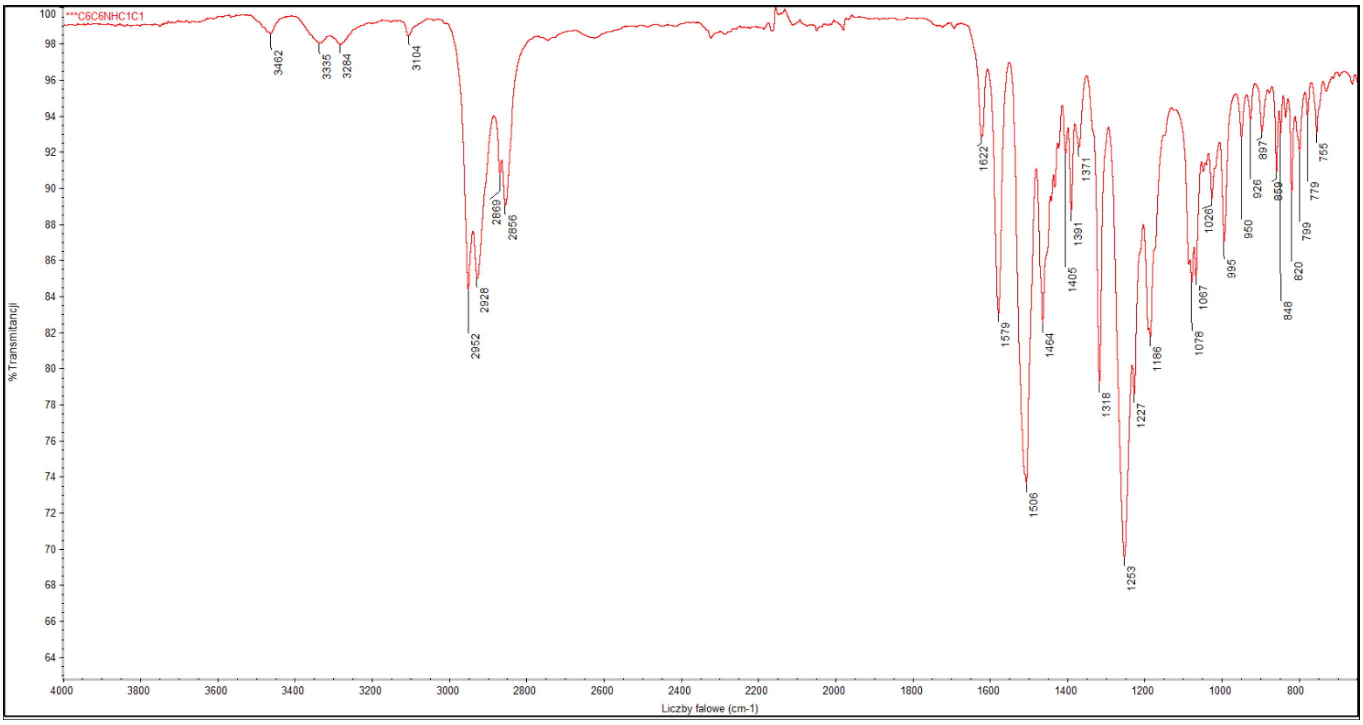

Figure S74. IR spectrum of **4**.

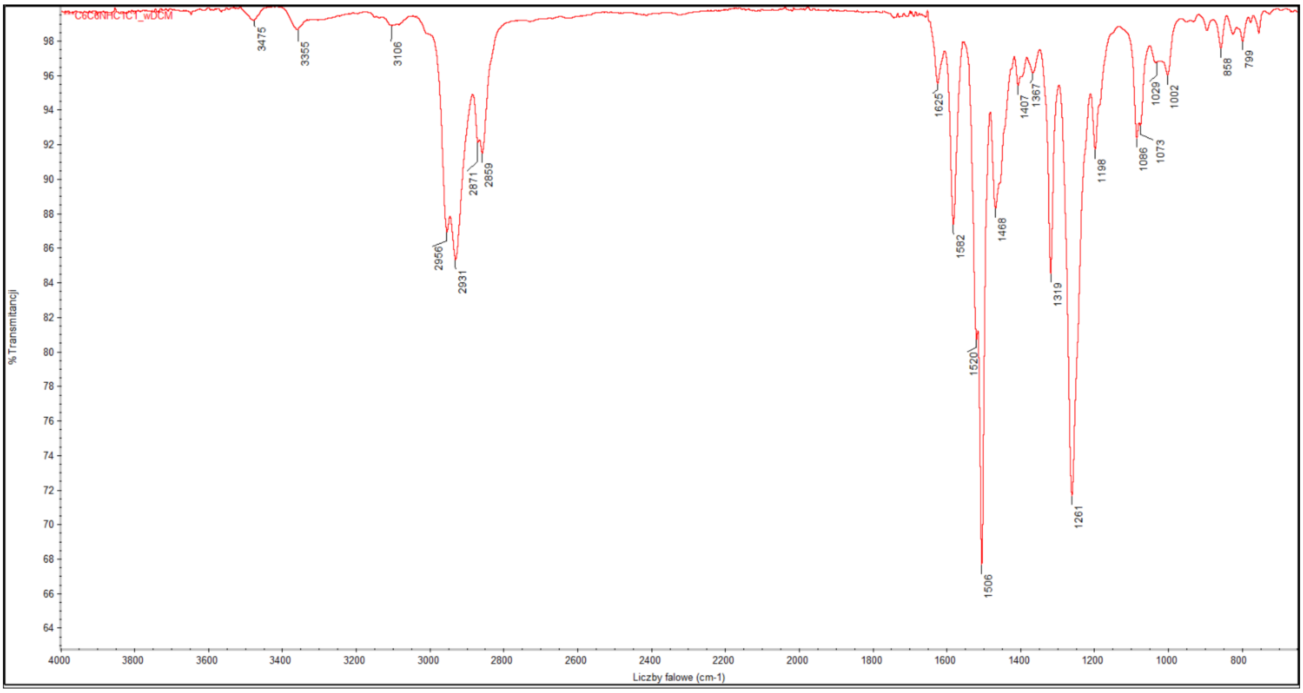

Figure S75. IR spectrum of **4** in solution.

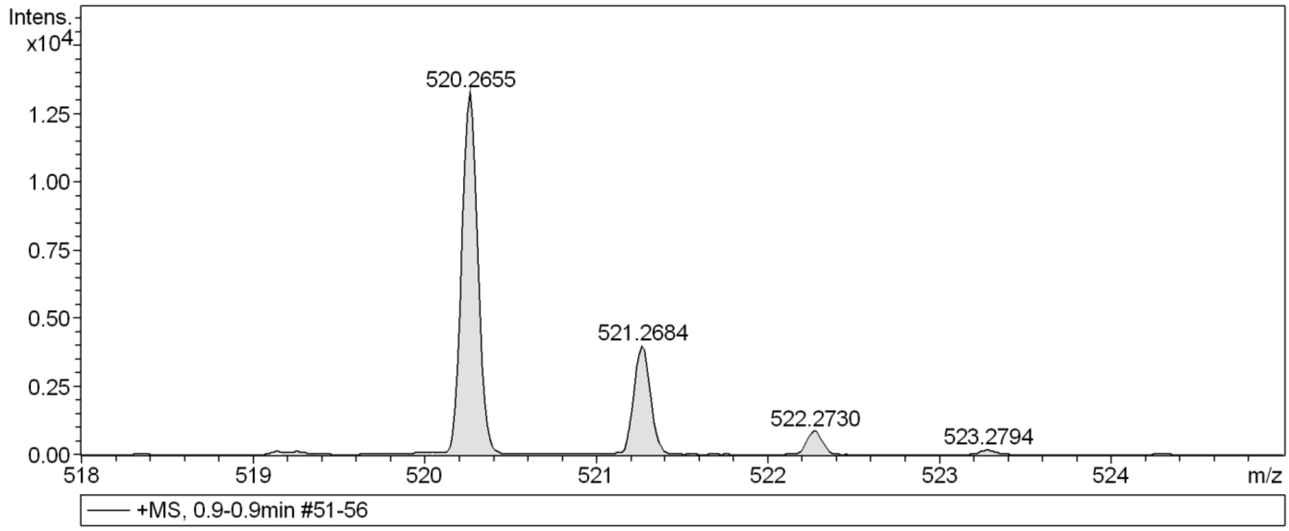

Figure S76. HRMS (ESI) spectrum of **4**.

7-(*tert*-butyl)-3-isobutoxy-2-methoxyphenazine (5a).

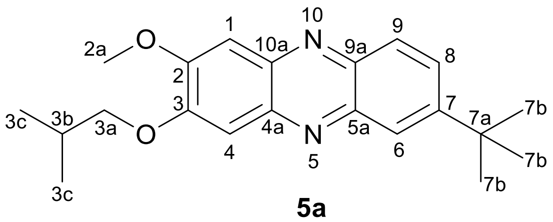

5a

^{1}H NMR ($CDCl_3$, 300 MHz, δ ppm): 8.08 (d, $^3J_{H8–H9}$ = 9.43 Hz, 1H, H_9), 8.06 (d, $^4J_{H6–H8}$ = 2.13 Hz, 1H, H_6), 7.85 (dd, $^3J_{H8–H9}$ = 9.43 Hz, $^4J_{H8–H6}$ = 2.13 Hz, 1H, H_8), 7.39 (s, 1H, H_4), 7.36 (s, 1H, H_1), 4.09 (s, 3H, H_{2a}), 4.02 (d, $^3J_{H3a–H3b}$ = 6.53 Hz, 2H, H_{3a}), 2.37 – 2.28 (m, 1H, H_{3b}), 1.47 (s, 9H, H_{7b}), 1.10 (d, $^3J_{H3b–H3c}$ = 6.64 Hz, 6H, H_{3c}).

^{13}C{^{1}H} NMR ($CDCl_3$, 75 MHz, δ ppm): 155.2 and 154.6 ($C_{2,3}$), 152.9 (C_{5a}), 142.6 and 142.0 ($C_{4a,10a}$), 142.5 (C_{9a}), 141.2 (C_7), 129.1 (C_8), 128.8 (C_9), 124.1 (C_6), 106.4 and 105.9 ($C_{1,4}$), 76.1 (C_{3a}), 57.0 (C_{2a}), 35.9 (C_{7a}), 31.6 (C_{7b}), 28.4 (C_{3b}), 19.9 (C_{3c}).

FT-IR (ATR, ν_{max}, (neat)/cm^{-1}): 3252, 3087, 3061, 3002, 2958, 2928, 2904, 2867, 1636, 1608, 1566, 1517, 1488, 1463, 1437, 1419, 1392, 1364, 1328, 1308, 1251, 1211, 1197, 1177, 1159, 1136, 1086, 1031, 1013, 966, 950, 905, 879, 855, 833, 818, 783.

HRMS (ESI) *m/z* Calculated for $C_{21}H_{27}N_2O_2$ [M+H]$^+$, 339.2068; found: 339.2065.

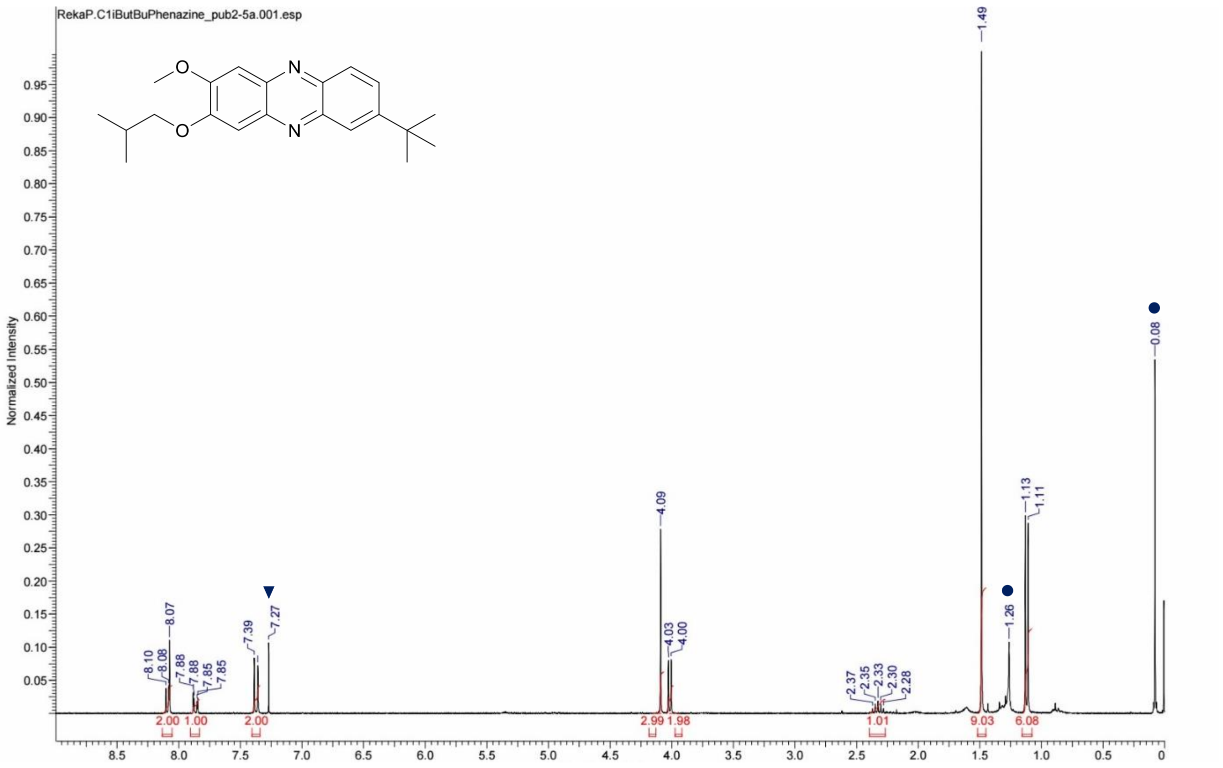

Figure S77. ^{1}H NMR ($CDCl_3$, 300 MHz, δ ppm) spectrum of **5a**.

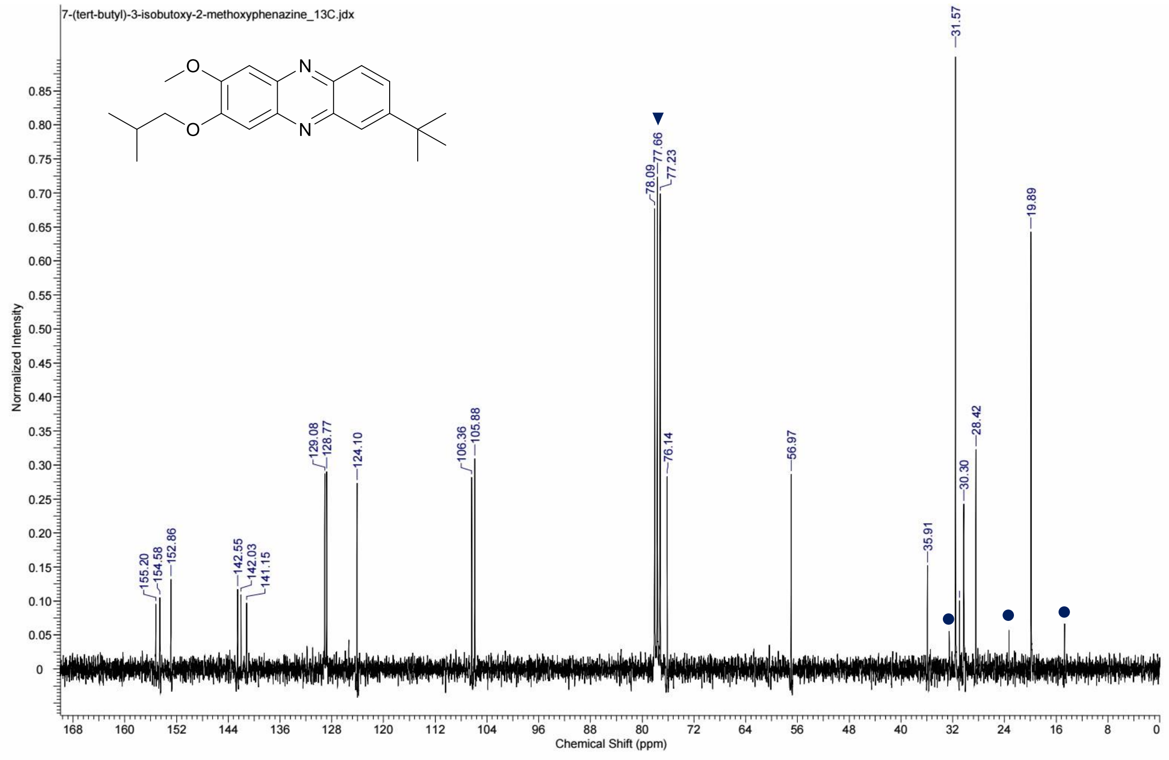

Figure S78. $^{13}C\{^{1}H\}$ NMR ($CDCl_3$, 75 MHz, δ ppm) NMR spectrum of **5a**.

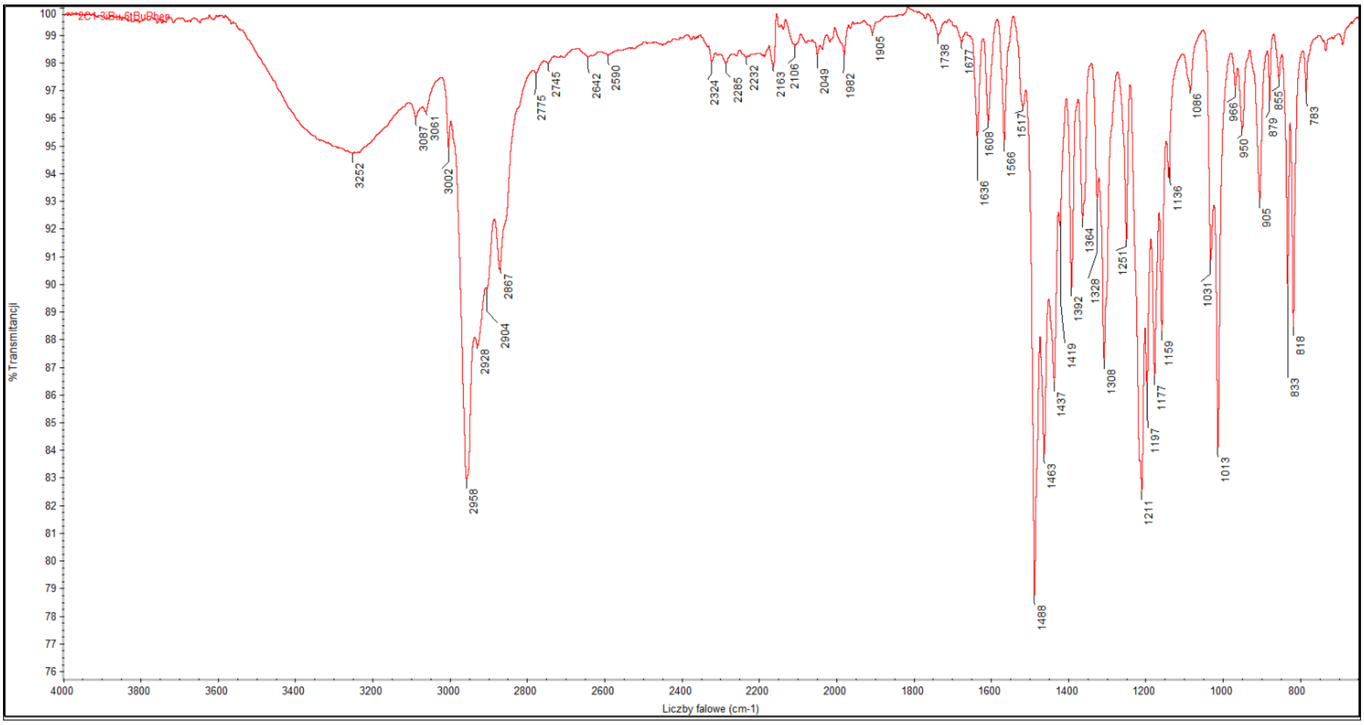

Figure S79. IR spectrum of **5a**.

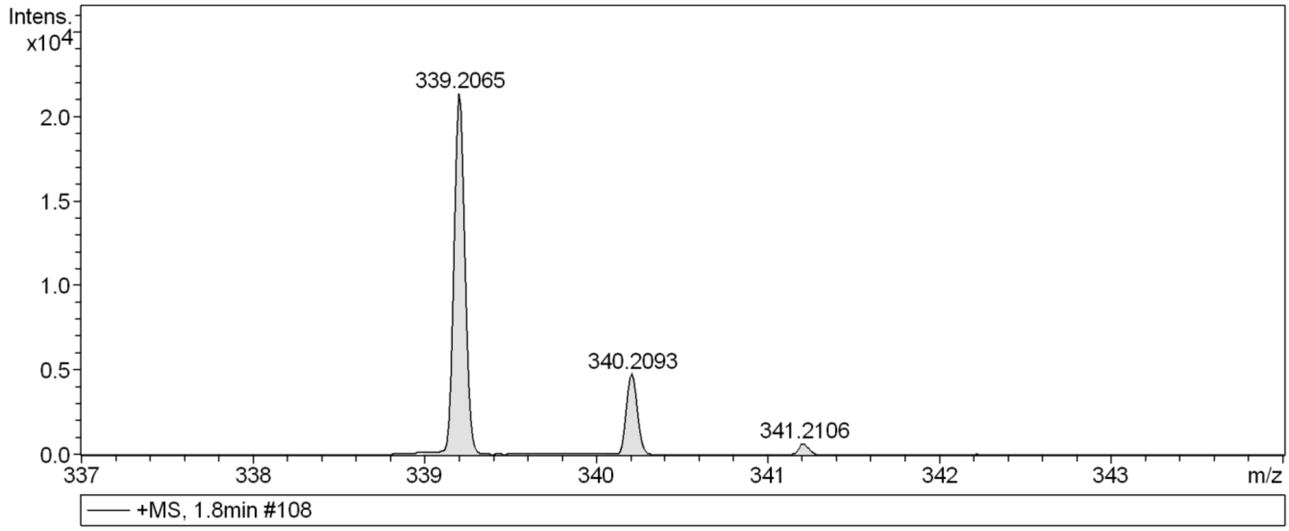

Figure S80. HRMS (ESI) spectrum of **5a**.

7-(*tert*-butyl)-2-ethoxy-3-isobutoxyphenazine (5b).

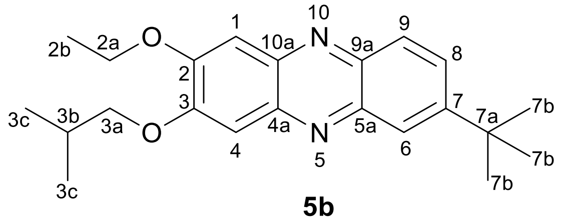

5b

^{1}H NMR ($CDCl_3$, 300 MHz, δ ppm): 8.08 (d, $^3J_{H8–H9}$ = 9.17 Hz, 1H, H_9), 8.06 (d, $^4J_{H6–H8}$ = 2.05 Hz, 1H, H_6), 7.84 (dd, $^3J_{H8–H9}$ = 9.17 Hz, $^4J_{H8–H6}$ = 2.05 Hz, 1H, H_8), 7.35 (s, 1H, H_4), 7.33 (s, 1H, H_1), 4.30 (q, $^3J_{H2a–H2b}$ = 6.98 Hz, 2H, H_{2a}), 3.99 (d, $^3J_{H3a–H3b}$ = 6.71 Hz, 2H, H_{3a}), 2.37 – 2.23 (m, 1H, H_{3b}), 1.57 (t, $^3J_{H2b–H2a}$ = 6.98 Hz, 3H, H_{2b}), 1.47 (s, 9H, H_{7b}), 1.11 (d, $^3J_{H3b–H3c}$ = 6.71 Hz, 6H, H_{3c}).

^{13}C{^{1}H} NMR ($CDCl_3$, 75 MHz, δ ppm): 154.8 and 154.6 ($C_{2,3}$), 152.7 (C_{5a}), 142.5 (C_{9a}), 142.1 ($C_{4a,10a}$), 141.1 (C_7), 129.0 (C_8), 128.7 (C_9), 125.4 (C_6), 106.3 ($C_{1,4}$), 76.0 (C_{3a}), 65.3 (C_{2a}), 35.9 (C_{7a}), 31.6 (C_{7b}), 28.5 (C_{3b}), 19.8 (C_{3c}), 15.1 (C_{2a}).

FT-IR (ATR, ν_{max}, (neat)/cm^{-1}): 3332, 2976, 2955, 2926, 2872, 2855, 1743, 1696, 1635, 1608, 1569, 1522, 1489, 1478, 1468, 1441, 1394, 1368, 1328, 1315, 1255, 1226, 1217, 1199, 1182, 1109, 1092, 1043, 1029, 959, 932, 894, 860, 853, 841, 823, 787.

HRMS (ESI) *m/z* Calculated for $C_{22}H_{29}N_2O_2$ [M+H]$^+$, 353.2224; found: 353.2223.

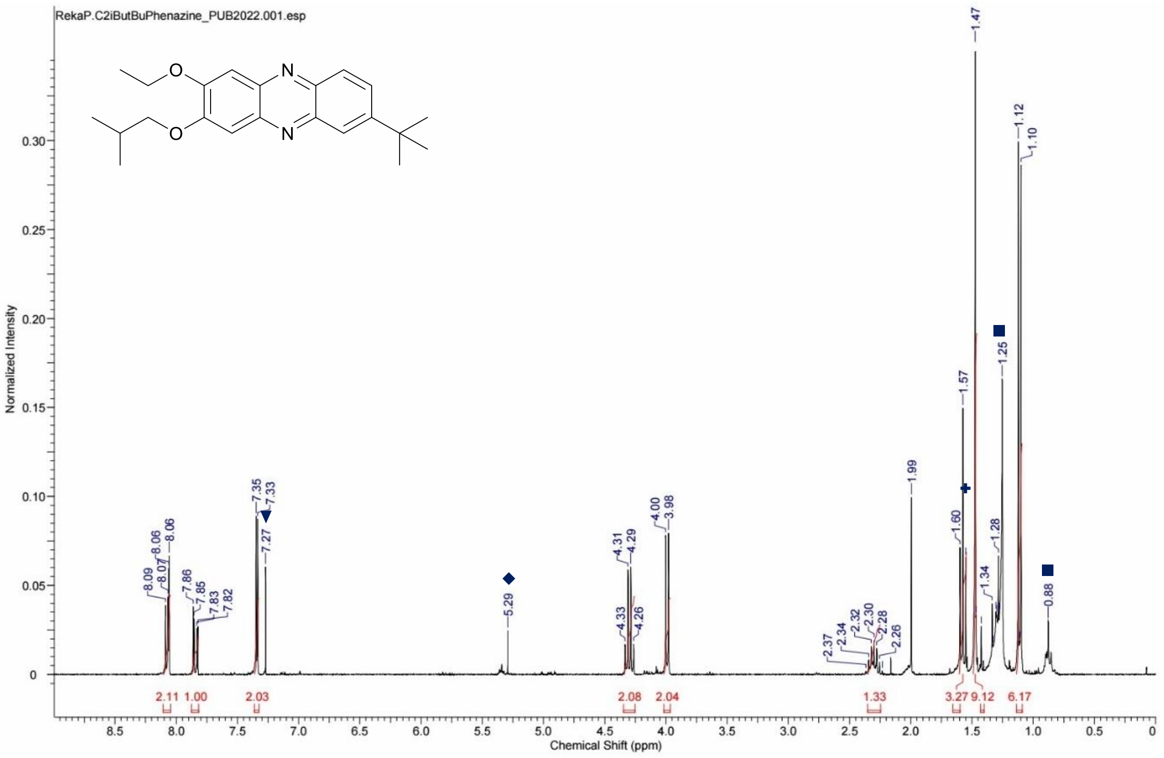

Figure S81. ^{1}H NMR ($CDCl_3$, 300 MHz, δ ppm) spectrum of **5b**.

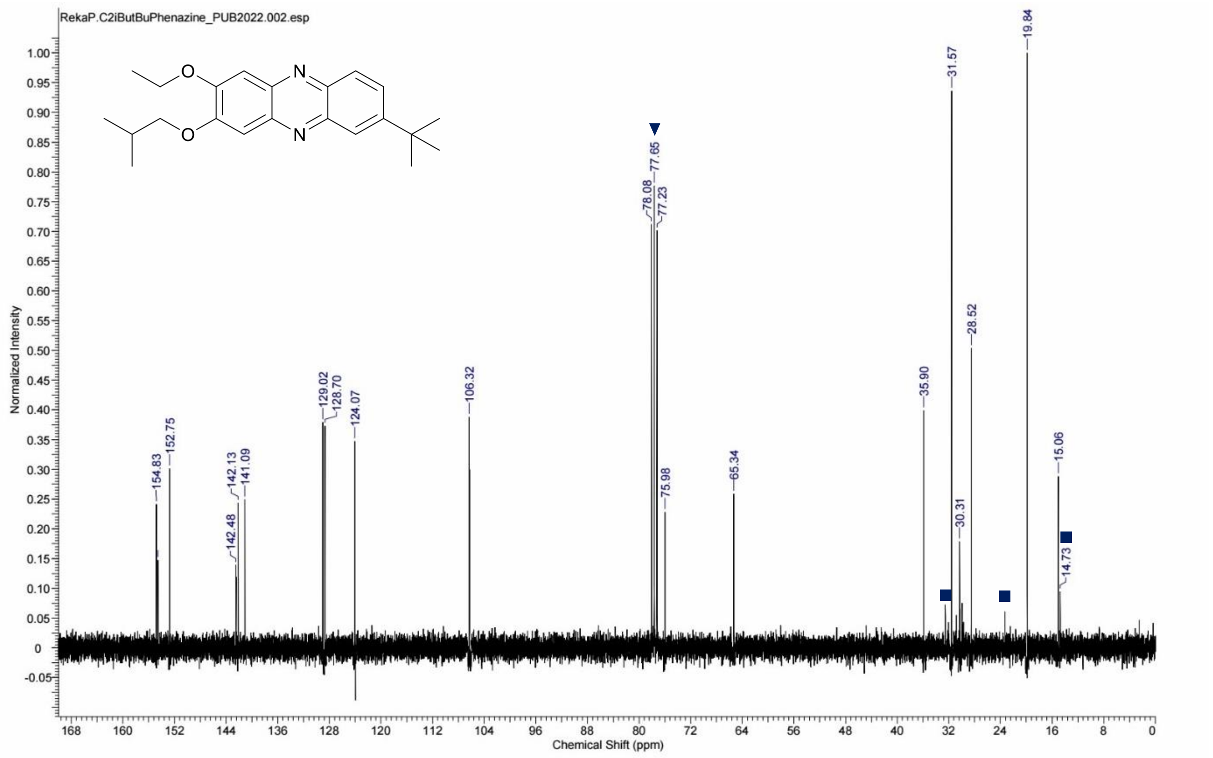

Figure S82. $^{13}C\{^{1}H\}$ NMR ($CDCl_3$, 75 MHz, δ ppm) NMR spectrum of **5b.**

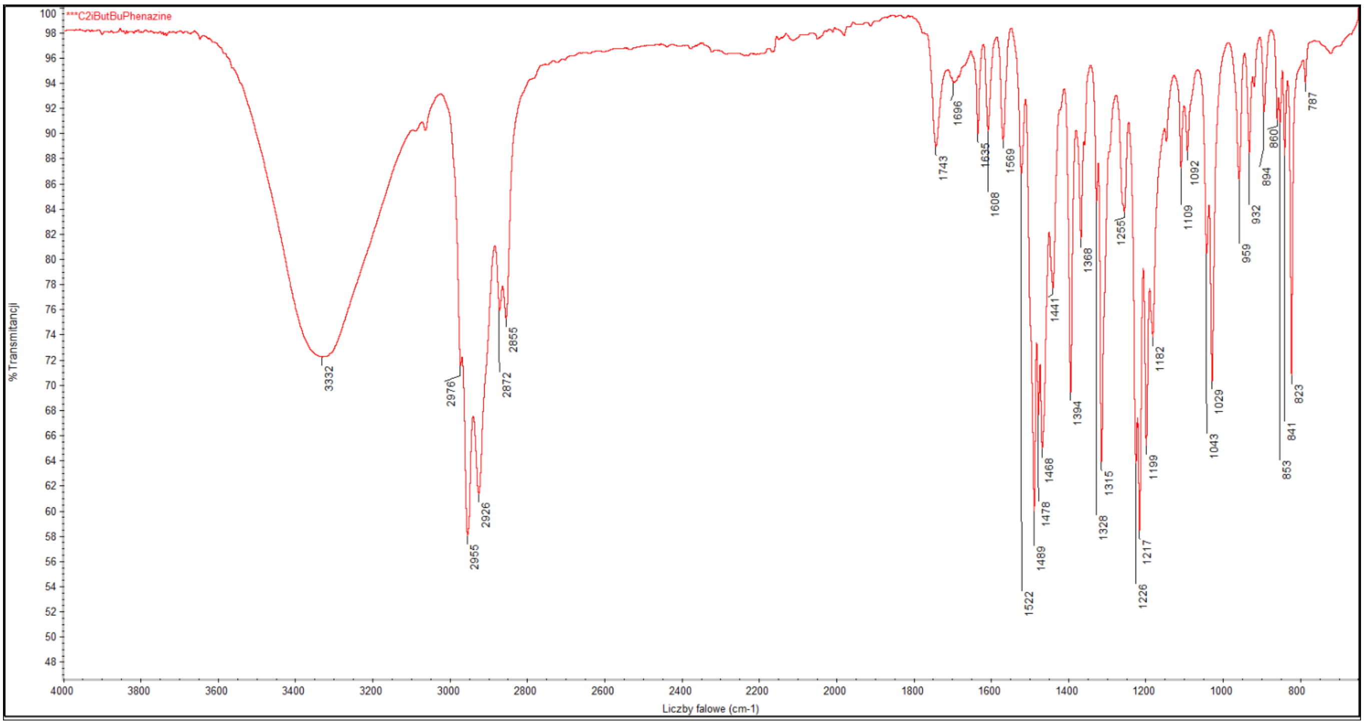

Figure S83. IR spectrum of **5b.**

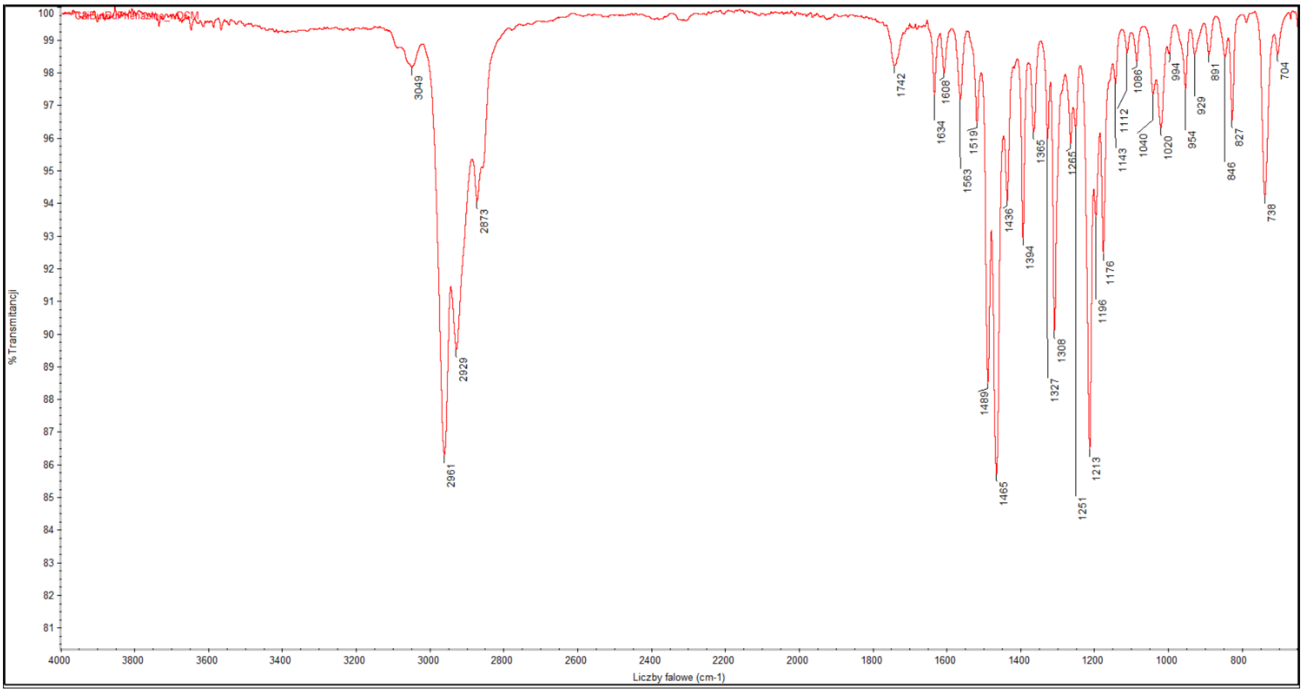

Figure S84. IR spectrum of **5b** in solution.

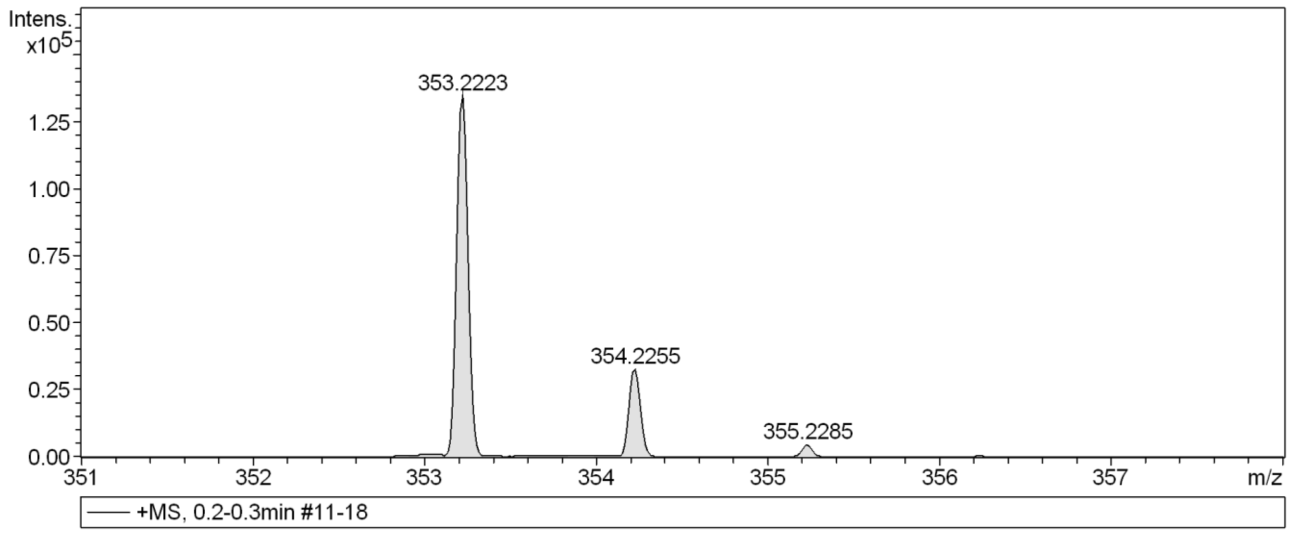

Figure S85. HRMS (ESI) spectrum of **5b**.

7-(*tert*-butyl)-3-ethoxy-2-isobutoxyphenazine (5c).

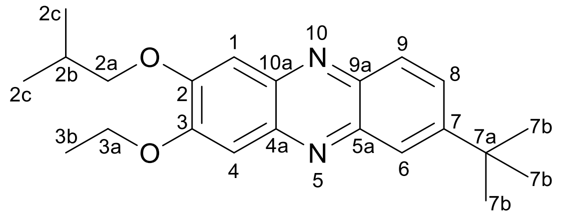

5c

^{1}H NMR ($CDCl_3$, 300 MHz, δ ppm): 8.07 (d, $^{3}J_{H8-H9}$ = 9.07 Hz, 1H, H_9), 8.06 (d, $^{4}J_{H6-H8}$ = 1.96 Hz, 1H, H_6), 7.85 (dd, $^{3}J_{H8-H9}$ = 9.07 Hz, $^{4}J_{H8-H6}$ = 1.96 Hz, 1H, H_8), 7.33 (s, 2H, $H_{1,4}$), 4.30 (q, $^{3}J_{H3a-H3b}$ = 6.97 Hz, 2H, H_{3a}), 3.99 (d, $^{3}J_{H2a-H2b}$ = 6.76 Hz, 2H, H_{2a}), 2.26 – 2.35 (m, 1H, H_{2b}), 1.59 (t, $^{3}J_{H3a-H3b}$ = 6.98 Hz, 3H, H_{3b}), 1.48 (s, 9H, H_{7b}), 1.12 (d, $^{3}J_{H2b-H2c}$ = 6.78 Hz, 6H, H_{2c}).

^{13}C{^{1}H} NMR ($CDCl_3$, 75 MHz, δ ppm): 154.8 and 154.6 ($C_{2,3}$), 152.8 (C_{5a}), 142.5 (C_{9a}), 142.4 and 142.1 ($C_{4a,10a}$), 141.1 (C_7), 129.0 (C_8), 128.7 (C_9), 124.0 (C_6), 106.3 and 106.2 ($C_{1,4}$), 76.0 (C_{2a}), 65.3 (C_{3a}), 35.9 (C_{7a}), 31.6 (C_{7b}), 28.5 (C_{2b}), 19.8 (C_{2c}), 15.0 (C_{3b}).

FT-IR (ATR, ν_{max}, (neat)/cm^{-1}): 3401, 3243, 2960, 2928, 2874, 2854, 1743, 1636, 1612, 1572, 1523, 1490, 1470, 1441, 1394, 1369, 1330, 1314, 1256, 1229, 1216, 1197, 1186, 1147, 1110, 1089, 1045, 1024, 996, 966, 937, 893, 839, 819.

HRMS (ESI) *m/z* Calculated for $C_{22}H_{29}N_2O_2$ $[M+H]^+$, 353.2224; found: 353.2222.

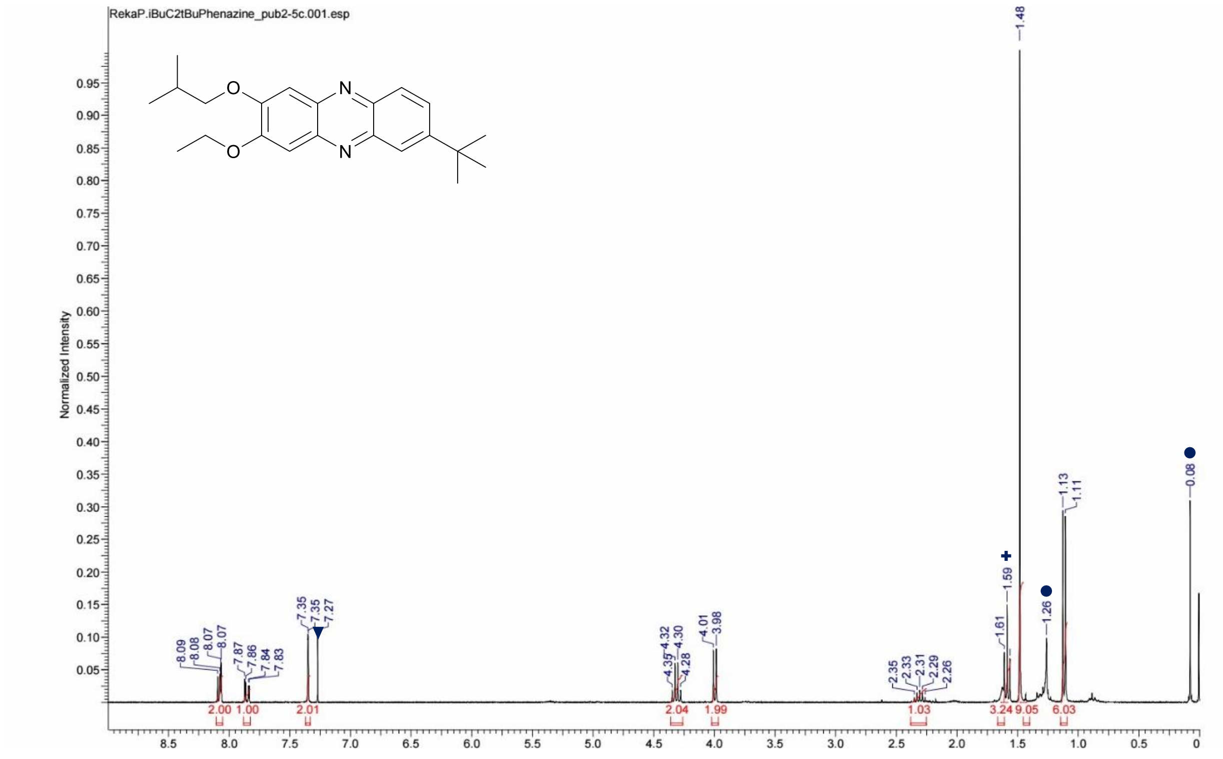

Figure S86. ^{1}H NMR ($CDCl_3$, 300 MHz, δ ppm) spectrum of **5c.**

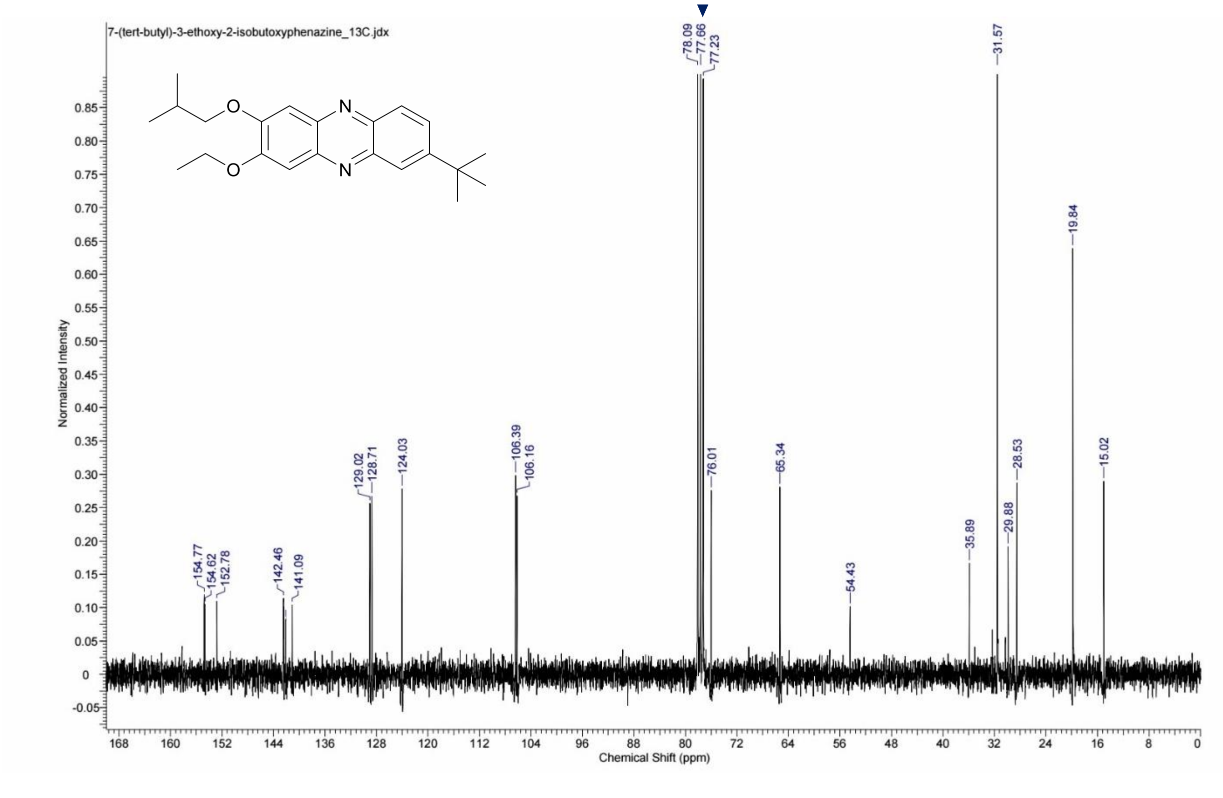

Figure S87. $^{13}C\{^{1}H\}$ NMR ($CDCl_3$, 75 MHz, δ ppm) NMR spectrum of **5c.**

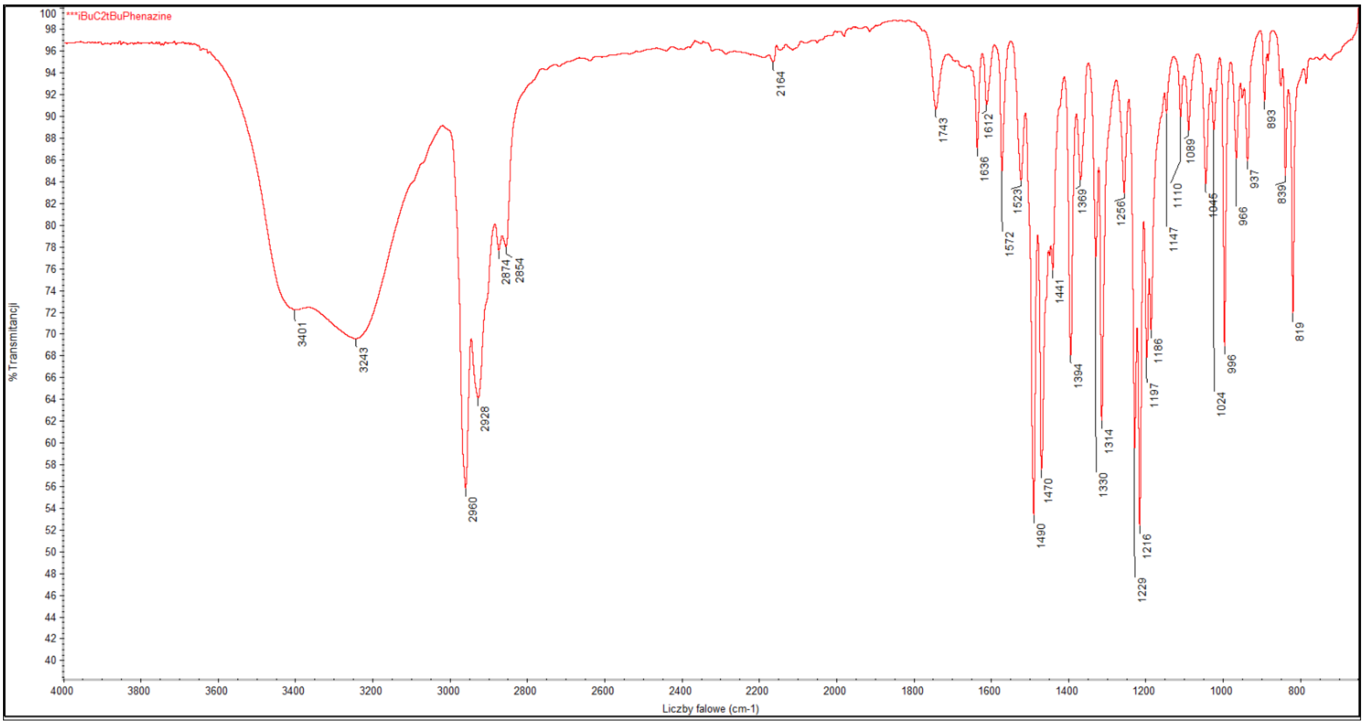

Figure S88. IR spectrum of **5c.**

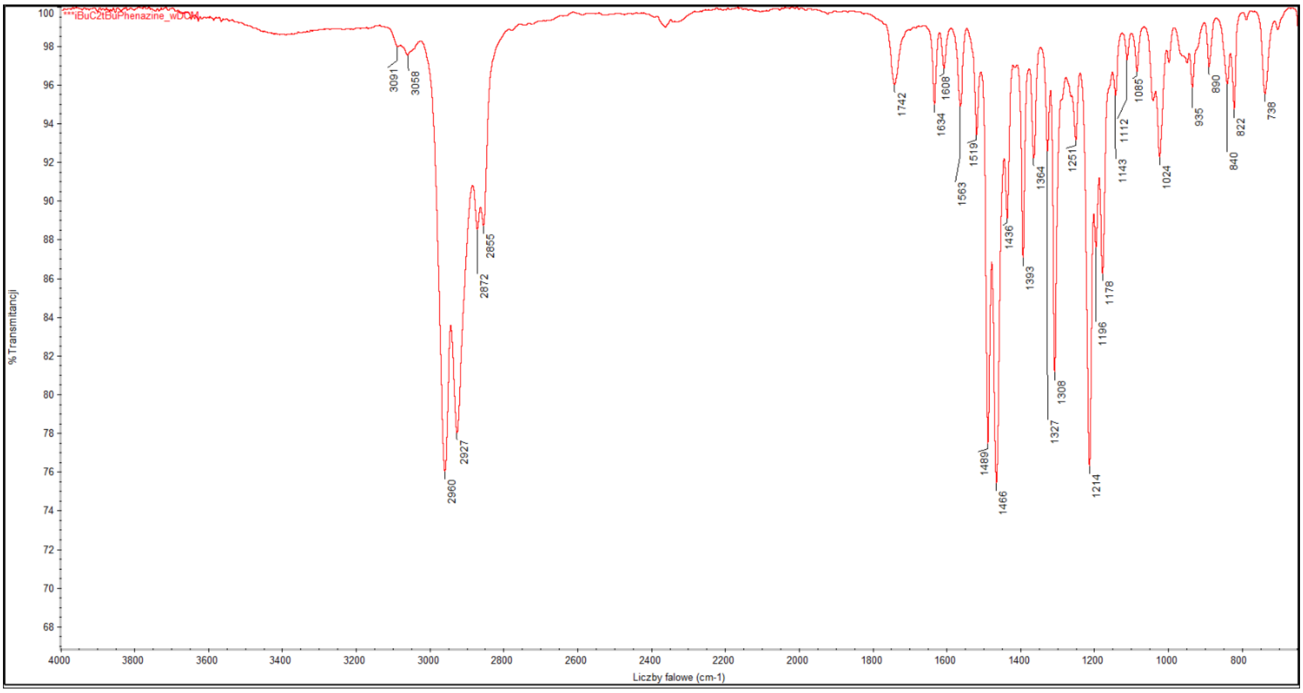

Figure S89. IR spectrum of **5c** in solution.

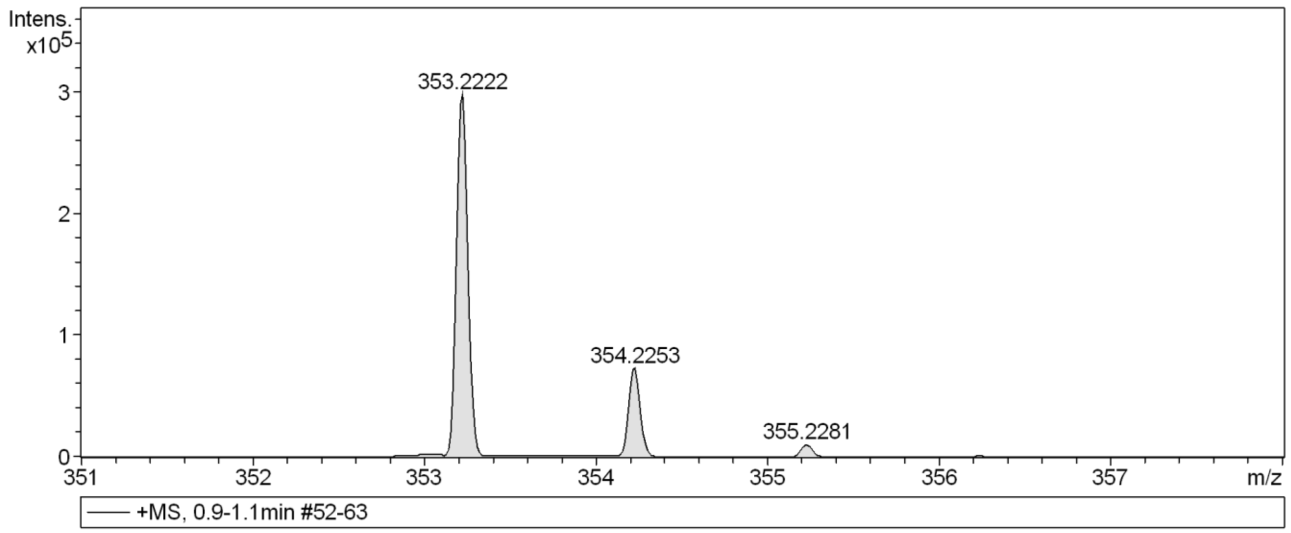

Figure S90. HRMS (ESI) spectrum of **5c**.

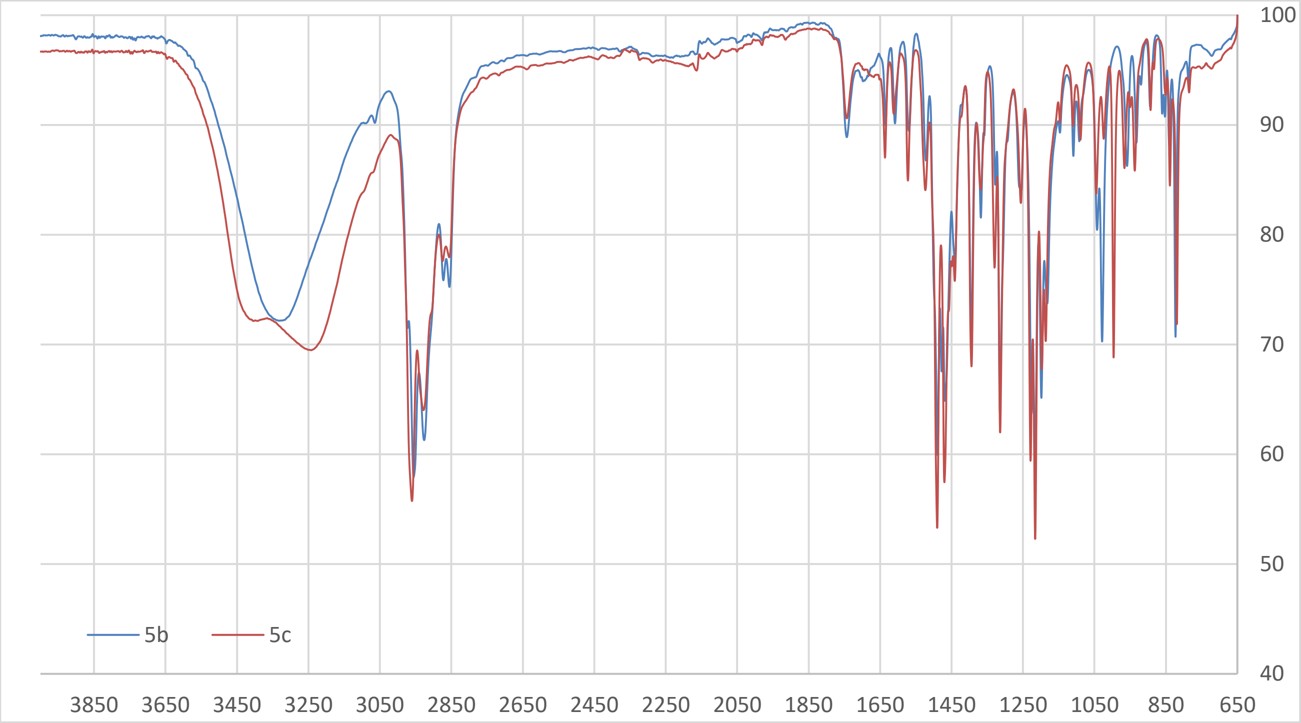

Figure S91. Comparison of IR spectra of compounds **5b** and **5c**.

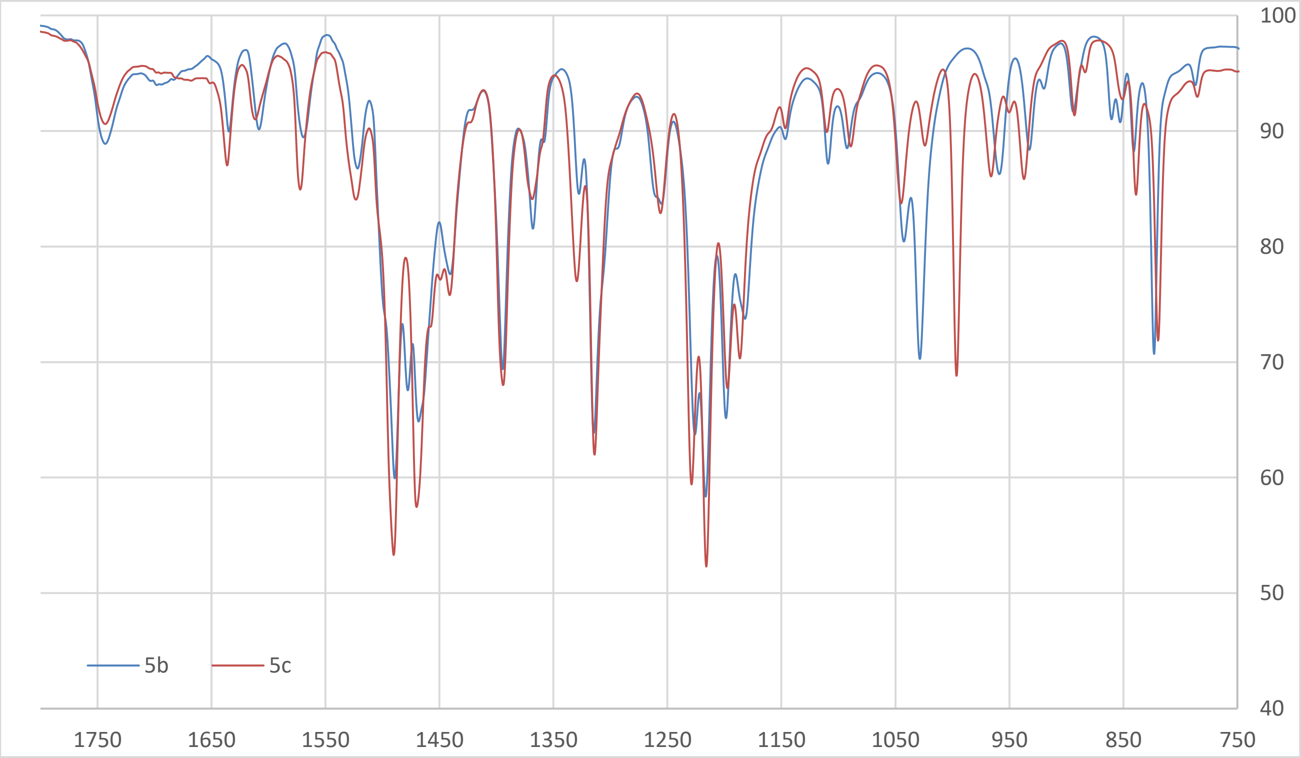

Figure S92. Comparison of IR spectra of compounds **5b** and **5c**, zoomed region.

2-butoxy-7-(*tert*-butyl)-3-isobutoxyphenazine (5d).

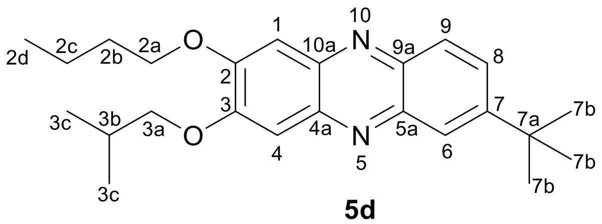

5d

^{1}H NMR (CDCl$_3$, 300 MHz, δ ppm): 8.08 (d, $^3J_{H8-H9}$ = 9.17 Hz, 1H, H$_9$), 8.07 (d, $^4J_{H6-H8}$ = 2.20 Hz, 1H, H$_6$), 7.85 (dd, $^3J_{H8-H9}$ = 9.17 Hz, $^4J_{H8-H6}$ = 2.20 Hz, 1H, H$_8$), 7.35 (s, 1H, H$_4$), 7.33 (s, 1H, H$_1$), 4.24 (t, $^3J_{H2a-H2b}$ = 6.42 Hz, 2H, H$_{2a}$), 3.99 (d, $^3J_{H3a-H3b}$ = 6.52 Hz, 2H, H$_{3a}$), 2.29 (m, 1H, H$_{3b}$), 2.01 – 1.89 (m, 2H, H$_{2b}$), 1.65 – 1.51 (m, 2H, H$_{2c}$), 1.49 (s, 9H, H$_{7b}$), 1.12 (d, $^3J_{H3b-H3c}$ = 6.55 Hz, 6H, H$_{3c}$), 1.04 (t, $^3J_{H2c-H2d}$ = 7.49 Hz, 3H, H$_{2d}$).

^{13}C{^{1}H} NMR (CDCl$_3$, 75 MHz, δ ppm): 155.6 and 154.9 (C$_{2,3}$), 152.7 (C$_{5a}$), 142.5 (C$_{9a}$), 142.5 and 142.2 (C$_{4a,10a}$), 141.1 (C$_7$), 129.0 (C$_8$), 128.7 (C$_9$), 124.1 (C$_6$), 106.3 and 106.2 (C$_{1,4}$), 75.9 (C$_{3a}$), 69.5 (C$_{2a}$), 35.9 (C$_{7a}$), 31.6 (C$_{7b}$), 31.5 (C$_{2b}$), 28.6 (C$_{3b}$), 19.9 (C$_{2c}$), 19.8 (C$_{3c}$), 14.5 (C$_{2d}$).

FT-IR (ATR, ν_{max}, (neat)/cm^{-1}): 2958, 2925, 2873, 2854, 1744, 1634, 1608, 1565, 1519, 1488, 1463, 1436, 1393, 1364, 1327, 1308, 1250, 1213, 1196, 1176, 1143, 1083, 1022, 969, 914, 826, 722.

HRMS (ESI) *m/z* Calculated for $C_{24}H_{33}N_2O_2$ [M+H]$^+$, 381.2537; found: 381.2538.

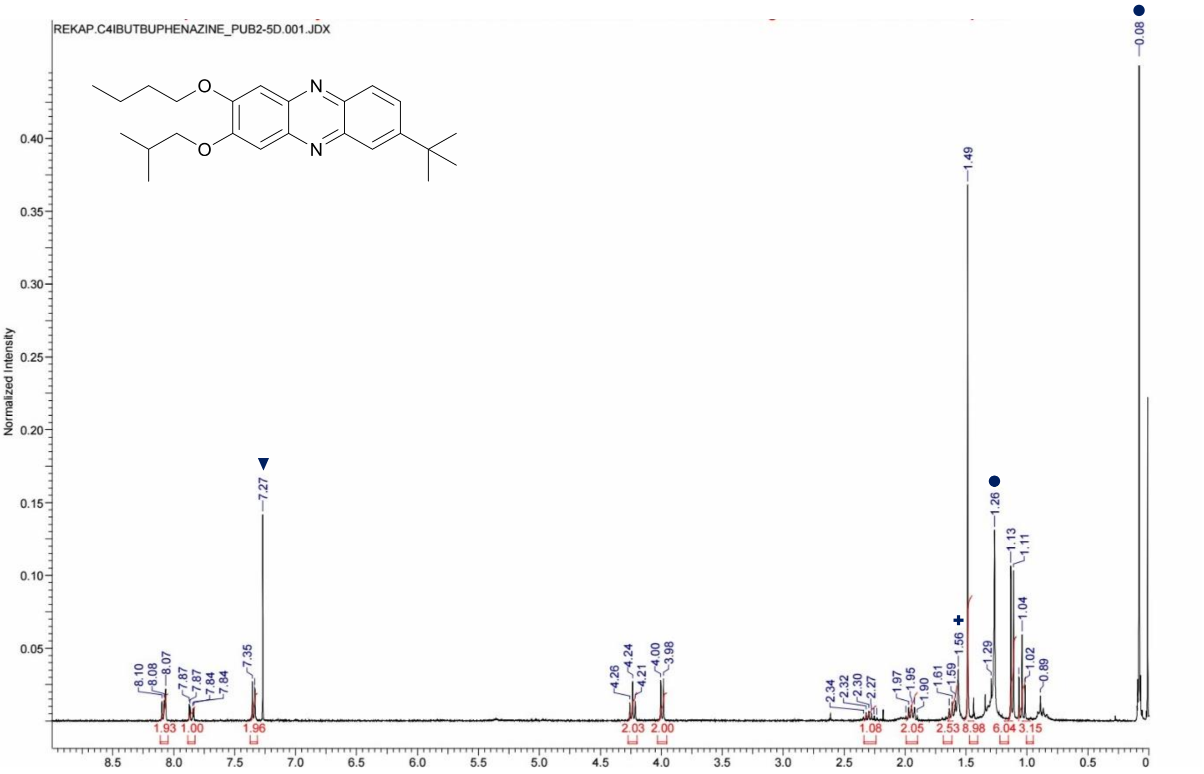

Figure S93. ^{1}H NMR ($CDCl_3$, 300 MHz, δ ppm) spectrum of **5d.**

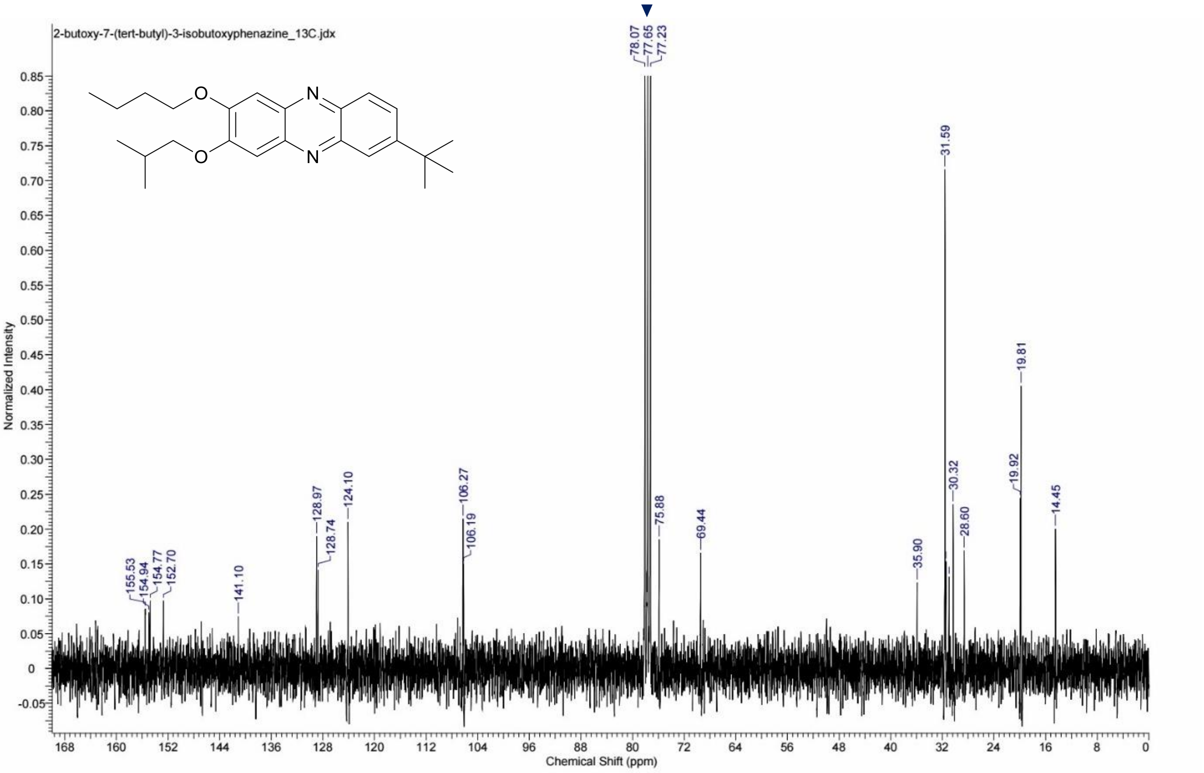

Figure S94. $^{13}C\{^1H\}$ NMR ($CDCl_3$, 75 MHz, δ ppm) NMR spectrum of **5d.**

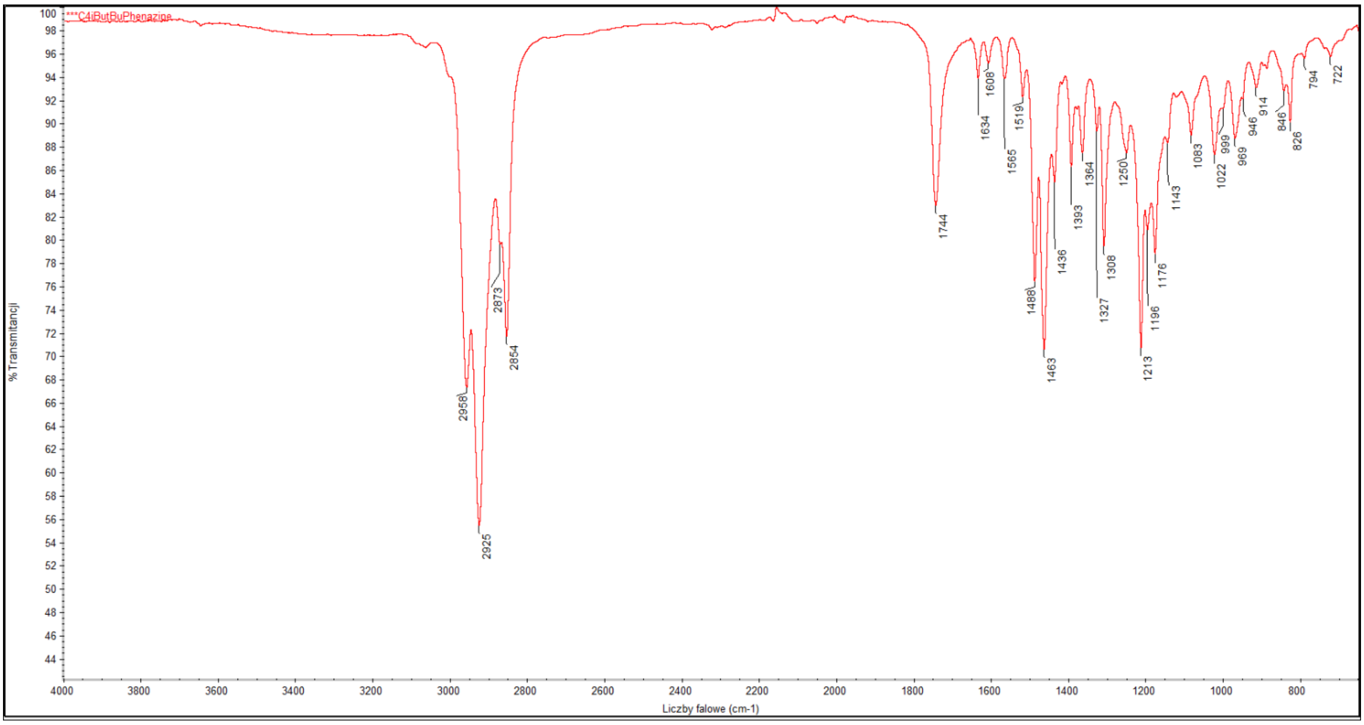

Figure S95. IR spectrum of **5d.**

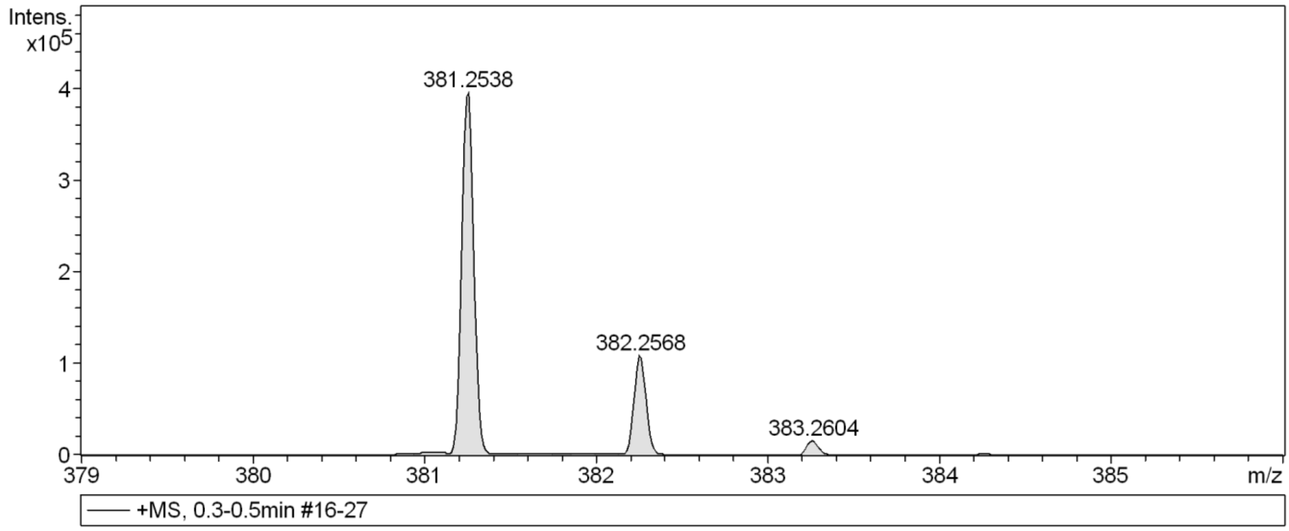

Figure S96. HRMS (ESI) spectrum of **5d**.

7-(*tert*-butyl)-2-(hexyloxy)-3-isobutoxyphenazine (5e).

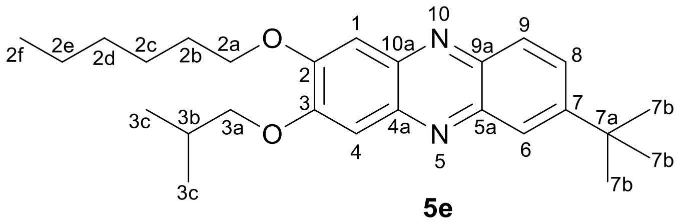

5e

^{1}H NMR ($CDCl_3$, 300 MHz, δ ppm): 8.08 (d, $^{3}J_{H8-H9}$ = 9.17 Hz, 1H, H_9), 8.07 (d, $^{4}J_{H6-H8}$ = 2.05 Hz, 1H, H_6), 7.85 (dd, $^{3}J_{H8-H9}$ = 9.17 Hz, $^{4}J_{H8-H6}$ = 2.05 Hz, 1H, H_8), 7.35 (s, 1H, H_4), 7.33 (s, 1H, H_1), 4.22 (t, 2H, H_{2a}), 3.99 (d, $^{3}J_{H3a-H3b}$ = 6.71 Hz, 2H, H_{3a}), 2.35 – 2.24 (m, 1H, H_{3b}), 2.00 – 1.90 (m, 2H, H_{2b}), 1.63 – 1.52 (m, 2H, H_{2c}), 1.49 (s, 9H, H_{7b}), 1.44 – 1.32 (m, 4H, $H_{2d,2e}$), 1.12 (d, $^{3}J_{H3b-H3c}$ = 6.71 Hz, 6H, H_{3c}), 0.93 (t, $^{3}J_{H2e-H2f}$ = 6.98 Hz, 3H, H_{2f}).

^{13}C{^{1}H} NMR ($CDCl_3$, 75 MHz, δ ppm): 155.0 ($C_{2,3}$), 152.7 (C_{5a}), 142.5 (C_{9a}), 142.4 and 142.2 ($C_{4a,10a}$), 141.1 (C_7), 129.0 (C_8), 128.7 (C_9), 124.1 (C_6), 106.2 and 106.1 ($C_{1,4}$), 75.9 (C_{3a}), 69.7 (C_{2a}), 35.9 (C_{7a}), 32.1 (C_{2d}), 31.6 (C_{7b}), 30.3 (C_{2b}), 29.4 (C_{3b}), 26.4 (C_{2c}), 23.2 (C_{2e}), 19.8 (C_{3c}), 14.6 (C_{2f}).

FT-IR (ATR, ν_{max}, (neat)/cm^{-1}): 2957, 2926, 2871, 2855, 1743, 1634, 1608, 1565, 1519, 1488, 1464, 1436, 1393, 1365, 1327, 1308, 1250, 1213, 1196, 1176, 1143, 1084, 1022.

HRMS (ESI) *m/z* Calculated for $C_{26}H_{37}N_2O_2$ $[M+H]^+$, 409.2850; found: 409.2849.

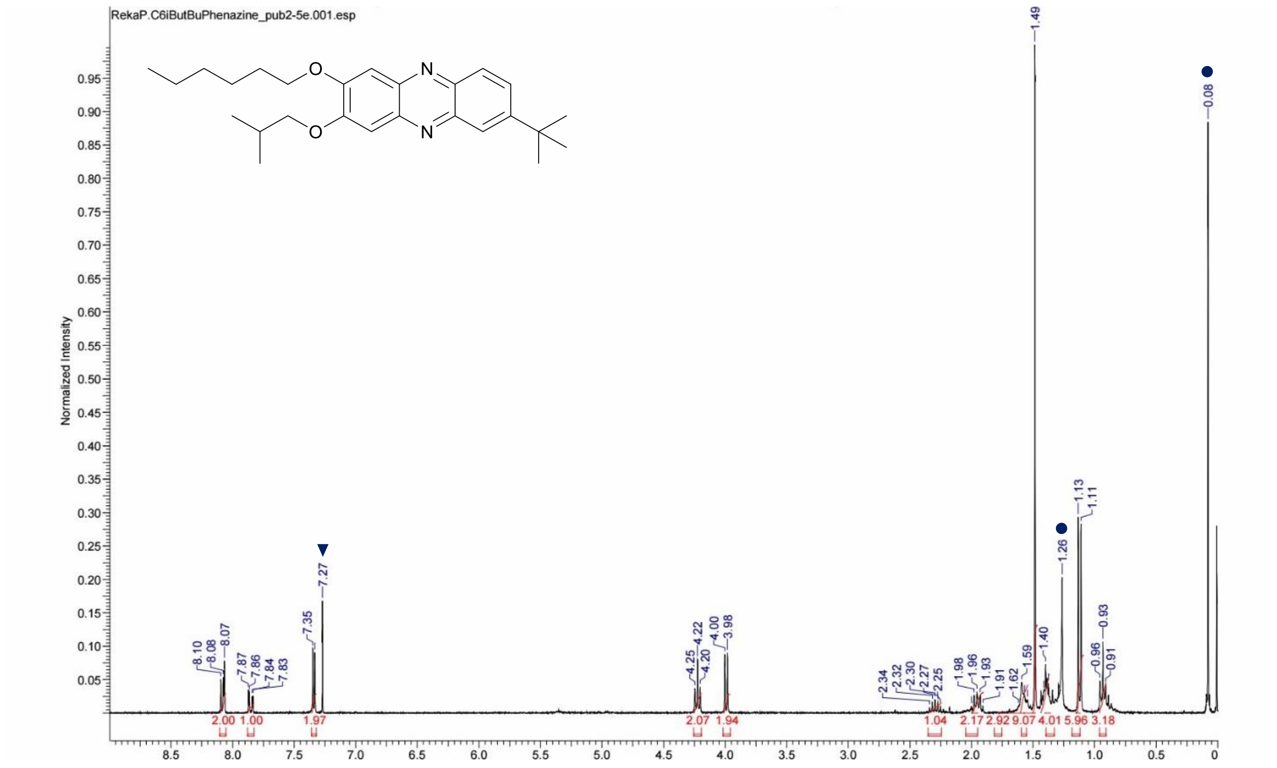

Figure S97. ^{1}H NMR ($CDCl_3$, 300 MHz, δ ppm) spectrum of **5e.**

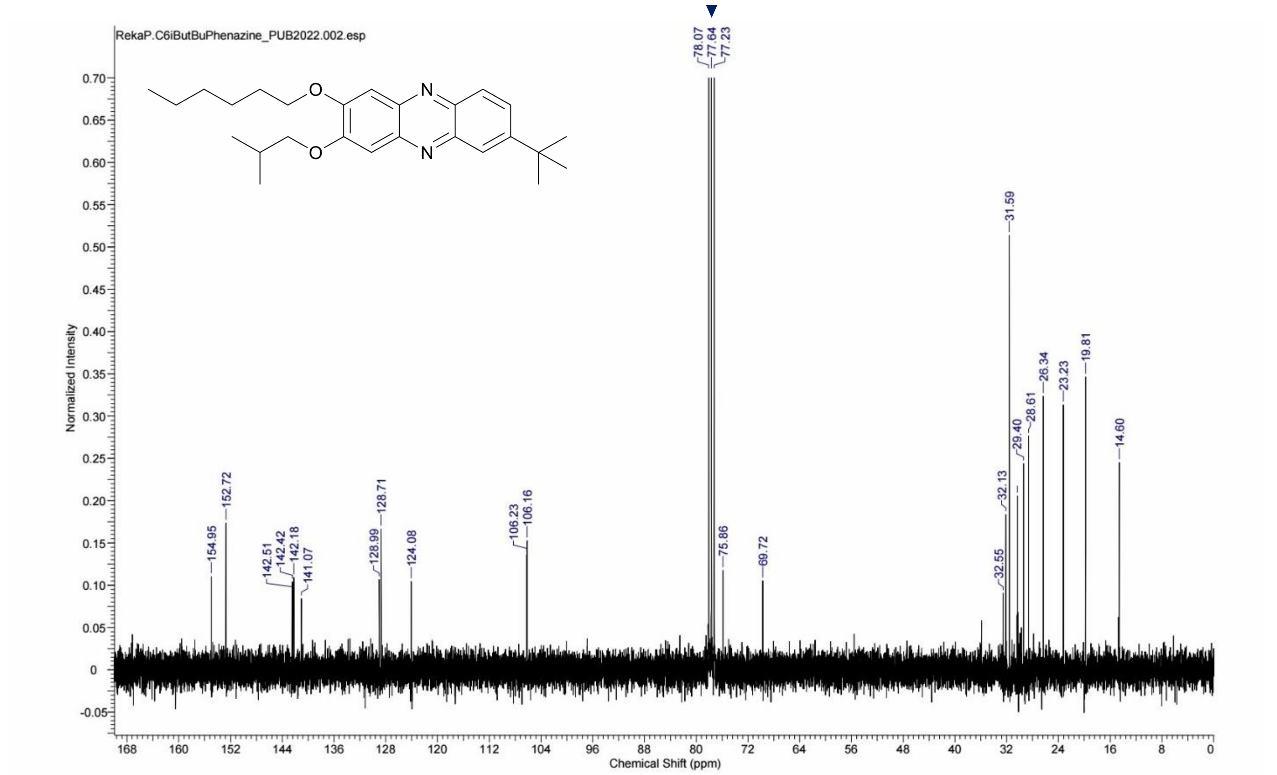

Figure S98. $^{13}C\{^{1}H\}$ NMR ($CDCl_3$, 75 MHz, δ ppm) NMR spectrum of **5e.**

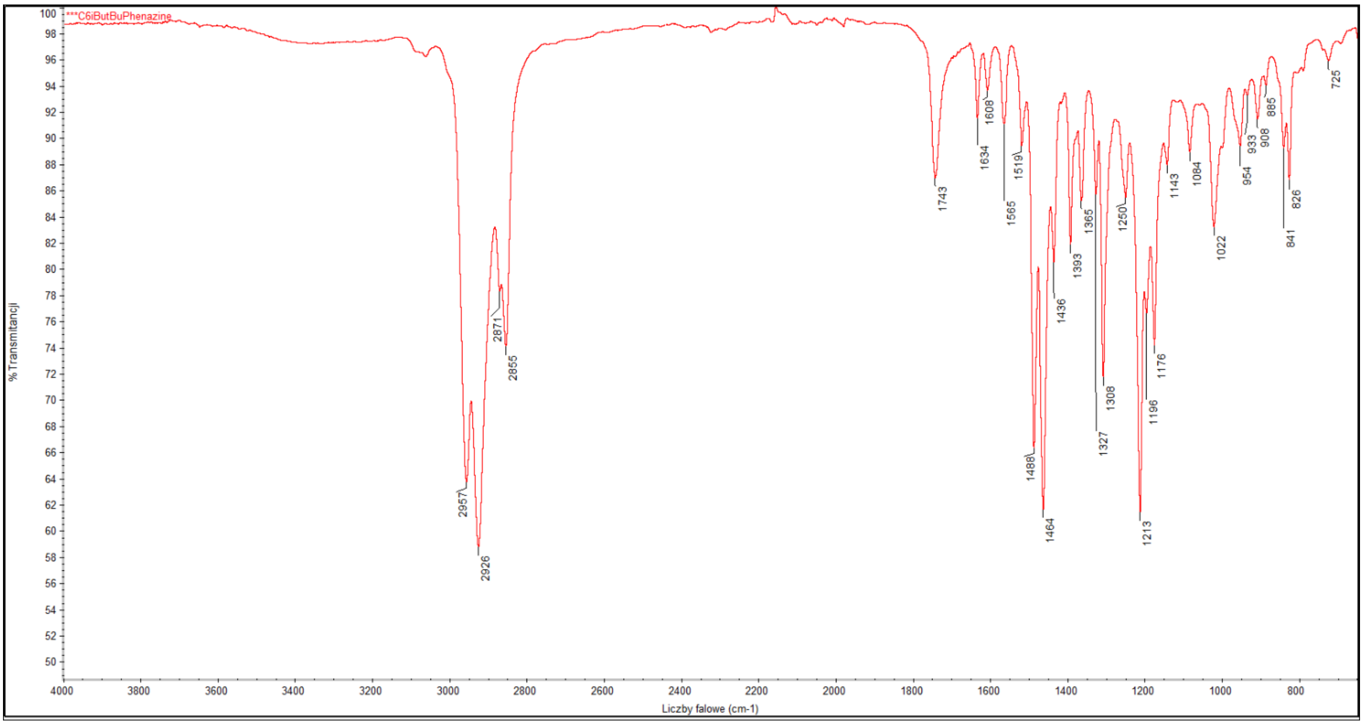

Figure S99. IR spectrum of **5e.**

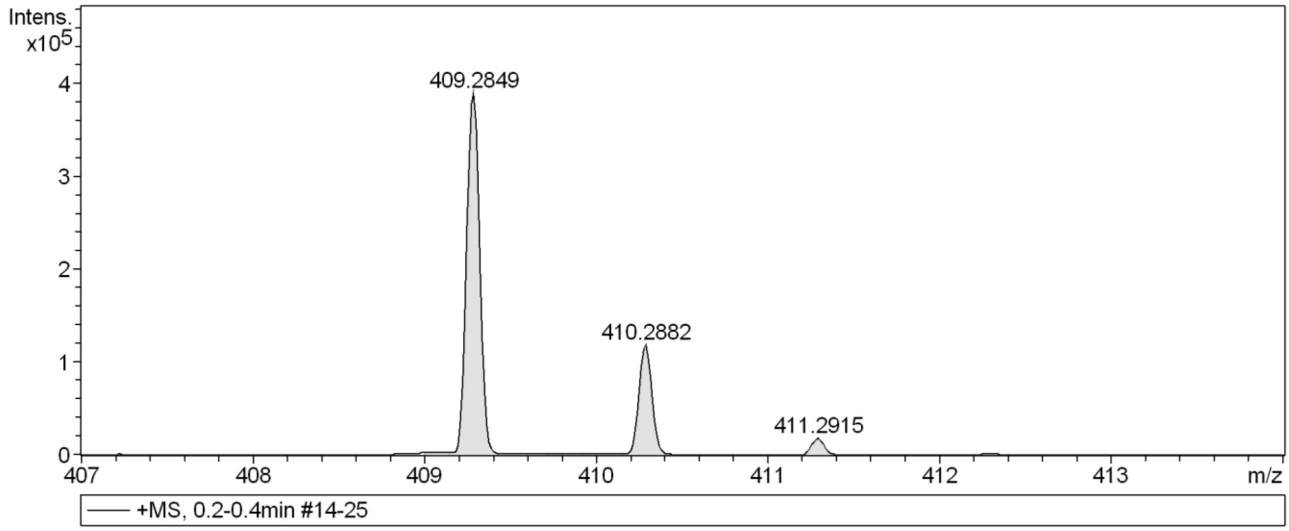

Figure S100. HRMS (ESI) spectrum of **5e**.

7-(*tert*-butyl)-3-isobutoxy-2-(octyloxy)phenazine (5f).

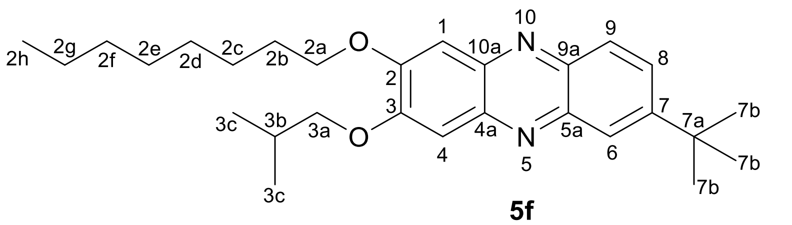

5f

^{1}H NMR ($CDCl_3$, 300 MHz, δ ppm): 8.07 (d, $^{3}J_{H8-H9}$ = 9.22 Hz, 1H, H_9), 8.06 (d, $^{3}J_{H6-H8}$ = 2.22 Hz, 1H, H_6), 7.85 (dd, $^{3}J_{H8-H9}$ = 9.20 Hz, $^{3}J_{H8-H6}$ = 2.22 Hz, 1H, H_8), 7.34 (s, 1H, H_4), 7.33 (s, 1H, H_1), 4.22 (t, $^{3}J_{H2a-H2b}$ = 6.51 Hz, 2H, H_{2a}), 3.99 (d, $^{3}J_{H3a-H3b}$ = 6.62 Hz, 2H, H_{3a}), 2.28 (m, 1H, H_{3b}), 2.06 – 1.88 (m, 2H, H_{2b}), 1.60 – 1.49 (m, 2H, H_{2c}), 1.48 (s, 9H, H_{7b}), 1.45 – 1.25 (m, 8H, $H_{2d,2e,2f,2g}$), 1.12 (d, $^{3}J_{H3b-H3c}$ = 6.87 Hz, 6H, H_{3c}), 0.90 (t, $^{3}J_{H2g-H2h}$ = 6.87 Hz, 3H, H_{2h}).

$^{13}C\{^{1}H\}$ NMR ($CDCl_3$, 75 MHz, δ ppm): 154.9 and 154.8 ($C_{2,3}$), 152.7 (C_{5a}), 142.5 (C_{9a}), 142.4 and 142.2 ($C_{4a,10a}$), 141.1 (C_7), 129.0 (C_8), 128.7 (C_9), 124.1 (C_6), 106.3 and 106.2 ($C_{1,4}$), 75.9 (C_{3a}), 69.7 (C_{2a}), 35.9 (C_{7a}), 32.4 (C_{2f}), 31.6 (C_{7b}), 30.3 (C_{2b}), 29.9 (C_{2d}), 29.5 (C_{2e}), 28.6 (C_{3b}), 26.7 (C_{2c}), 23.3 (C_{2g}), 19.8 (C_{3c}), 14.7 (C_{2h}).

FT-IR (ATR, ν_{max}, (neat)/cm^{-1}): 3085, 3056, 1976, 2954, 2921, 2871, 2855, 1744, 1634, 1607, 1562, 1519, 1489, 1466, 1434, 1392, 1364, 1327, 1307, 1297, 1249, 1213, 1195, 1177, 1140, 1084, 1038, 1022, 997, 948, 908, 885, 843, 829, 725.

HRMS (ESI) *m/z* Calculated for $C_{28}H_{41}N_2O_2$ $[M+H]^+$, 437.3163; found: 437.3162.

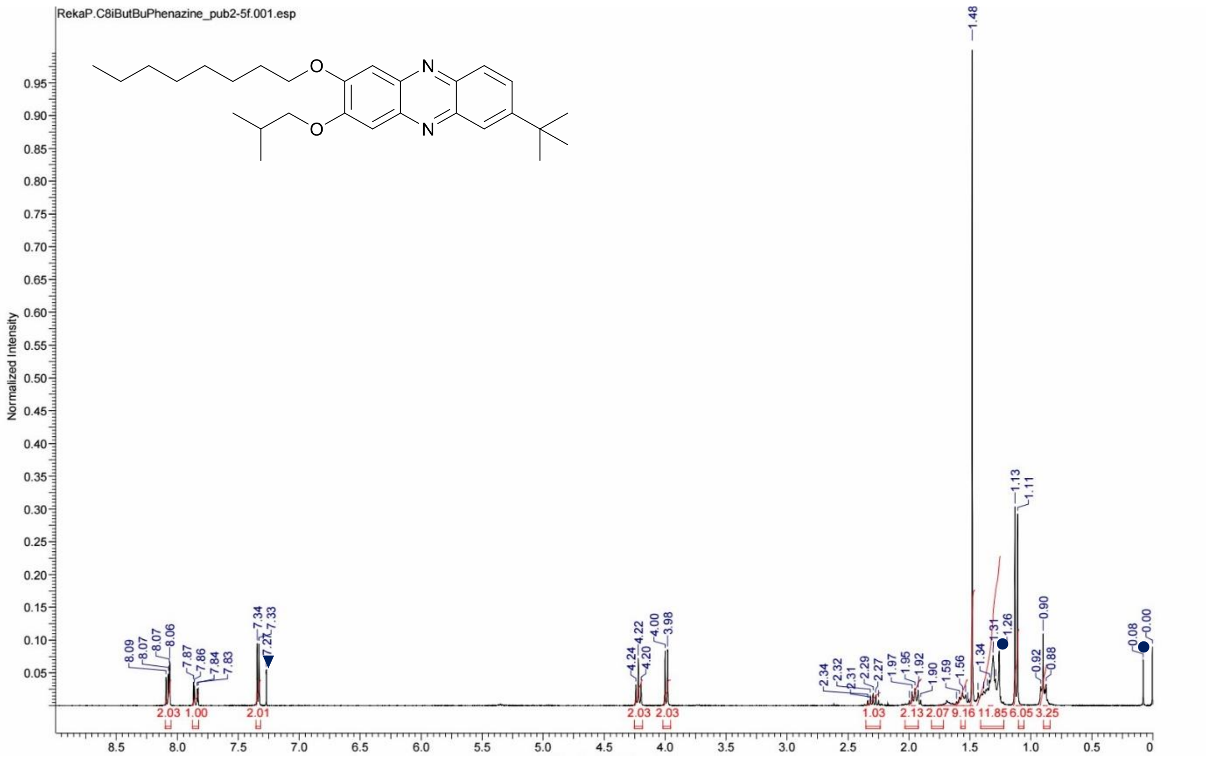

Figure S101. ^{1}H NMR ($CDCl_3$, 300 MHz, δ ppm) spectrum of **5f.**

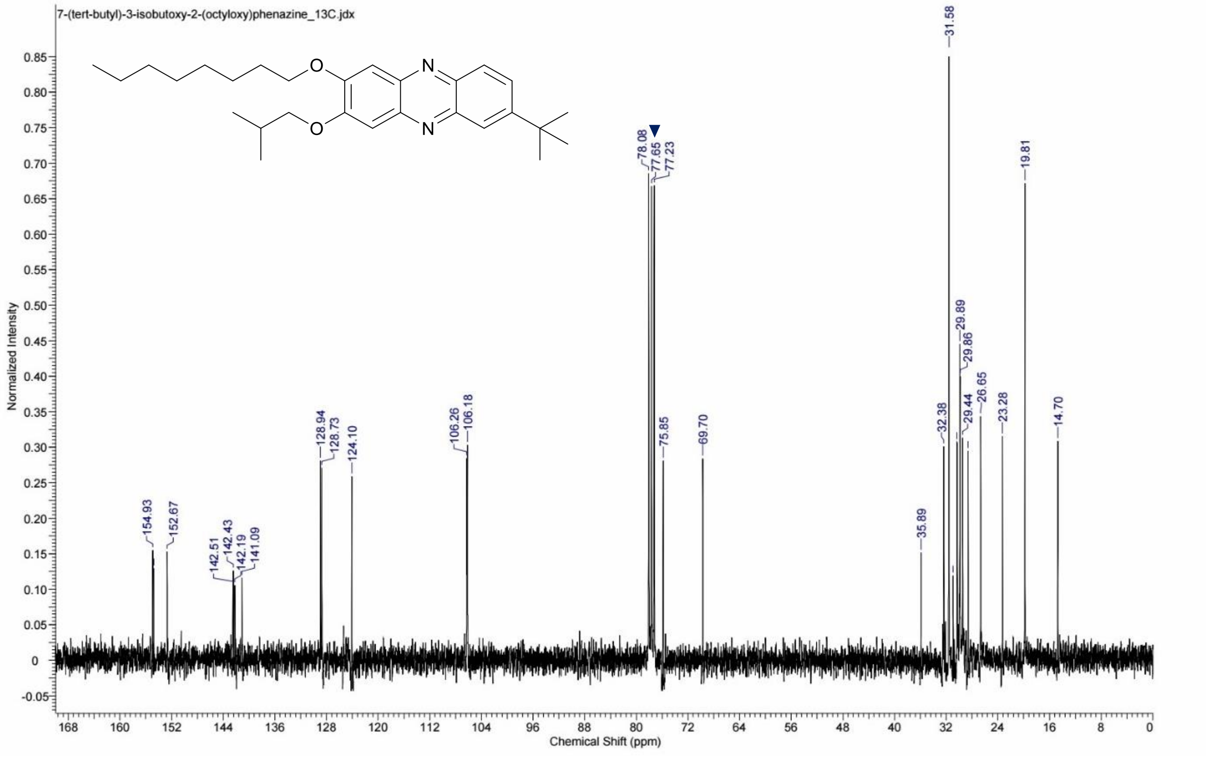

Figure S102. $^{13}C\{^1H\}$ NMR ($CDCl_3$, 75 MHz, δ ppm) NMR spectrum of **5f.**

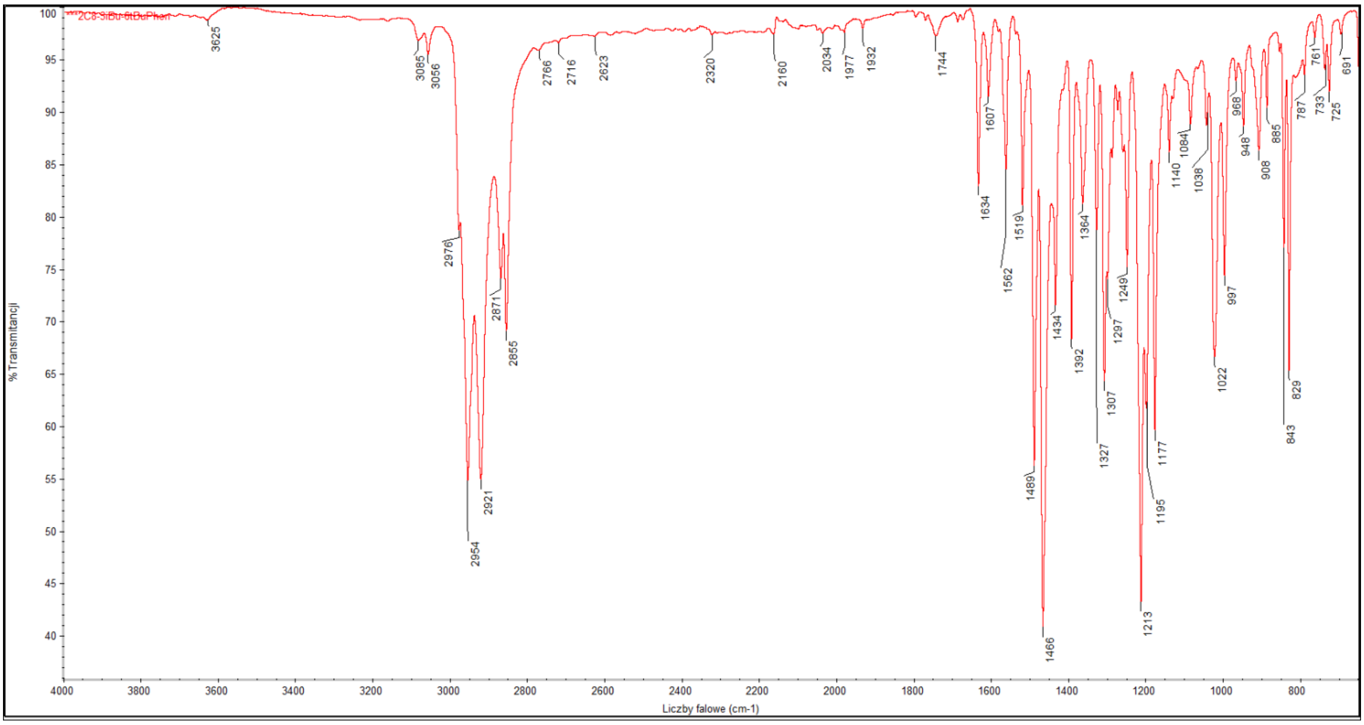

Figure S103. IR spectrum of **5f.**

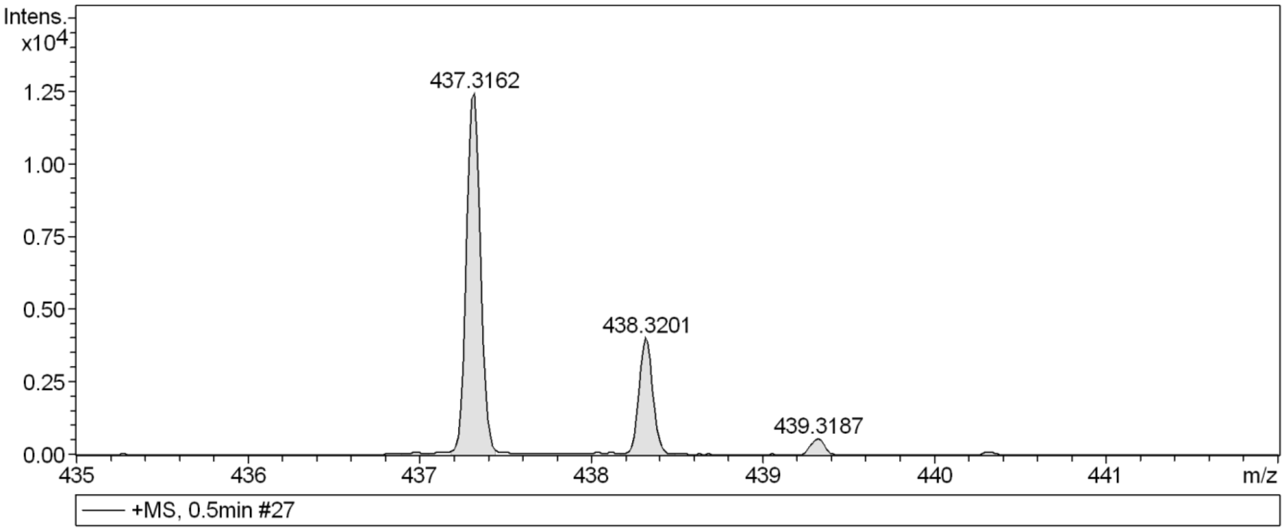

Figure S104. HRMS (ESI) spectrum of **5f**.

7-(*tert*-butyl)-2-(decyloxy)-3-isobutoxyphenazine (5g).

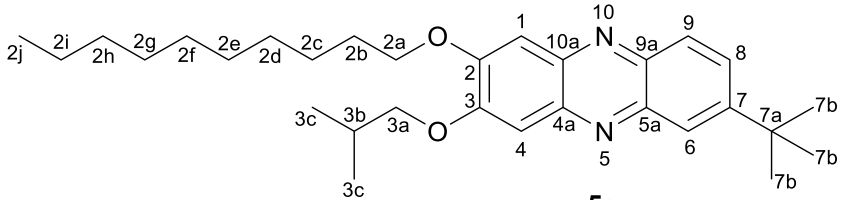

5g

^{1}H NMR ($CDCl_3$, 300 MHz, δ ppm): 8.07 (d, $^{3}J_{H8–H9}$ = 9.18 Hz, 1H, H_9), 8.06 (d, $^{4}J_{H6–H8}$ = 2.26 Hz, 1H, H_6), 7.84 (dd, $^{3}J_{H8–H9}$ = 9.18 Hz, $^{4}J_{H8–H6}$ = 2.26 Hz, 1H, H_8), 7.34 (s, 1H, H_4), 7.32 (s, 1H, H_1), 4.21 (t, $^{3}J_{H2a–H2b}$ = 6.45 Hz, 2H, H_{2a}), 3.98 (d, $^{3}J_{H3a–H3b}$ = 6.70 Hz, 2H, H_{3a}), 2.28 (m, 1H, H_{3b}), 2.00 – 1.88 (m, 2H, H_{2b}), 1.60 – 1.49 (m, 2H, H_{2c}), 1.48 (s, 9H, H_{7b}), 1.45 – 1.22 (m, 12H, $H_{2d,2e,2f,2g,2h,2i}$), 1.11 (d, $^{3}J_{H3b–H3c}$ = 6.93 Hz, 6H, H_{3c}), 0.89 (t, $^{3}J_{H2i–H2j}$ = 6.66 Hz, 3H, H_{2j}).

^{13}C{^{1}H} NMR ($CDCl_3$, 75 MHz, δ ppm): 154.9 and 154.7 ($C_{2,3}$), 152.7 (C_{5a}), 142.5 (C_{9a}), 142.4 and 142.2 ($C_{4a,10a}$), 141.1 (C_7), 128.9 (C_8), 128.7 (C_9), 124.1 (C_6), 106.3 and 106.2 ($C_{1,4}$), 75.8 (C_{3a}), 69.7 (C_{2a}), 35.9 (C_{7a}), 32.4 (C_{2h}), 31.6 (C_{7b}), 30.4 – 29.8 (m, 4C, $C_{2b,2d,2e,2f}$), 29.4 (C_{2g}), 28.6 (C_{3b}), 26.7 (C_{2c}), 23.3 (C_{2i}), 19.8 (C_{3c}), 14.7 (C_{2j}).

FT-IR (ATR, ν_{max}, (neat)/cm^{-1}): 3087, 3061, 2957, 2926, 2871, 2855, 1634, 1607, 1565, 1519, 1487, 1463, 1436, 1393, 1364, 1327, 1308, 1250, 1212, 1196, 1175, 1142, 1084, 1022, 994, 950, 911, 885, 840, 826, 792, 765, 750.

HRMS (ESI) *m/z* Calculated for $C_{30}H_{45}N_2O_2$ $[M+H]^+$, 465.3476; found: 465.3475.

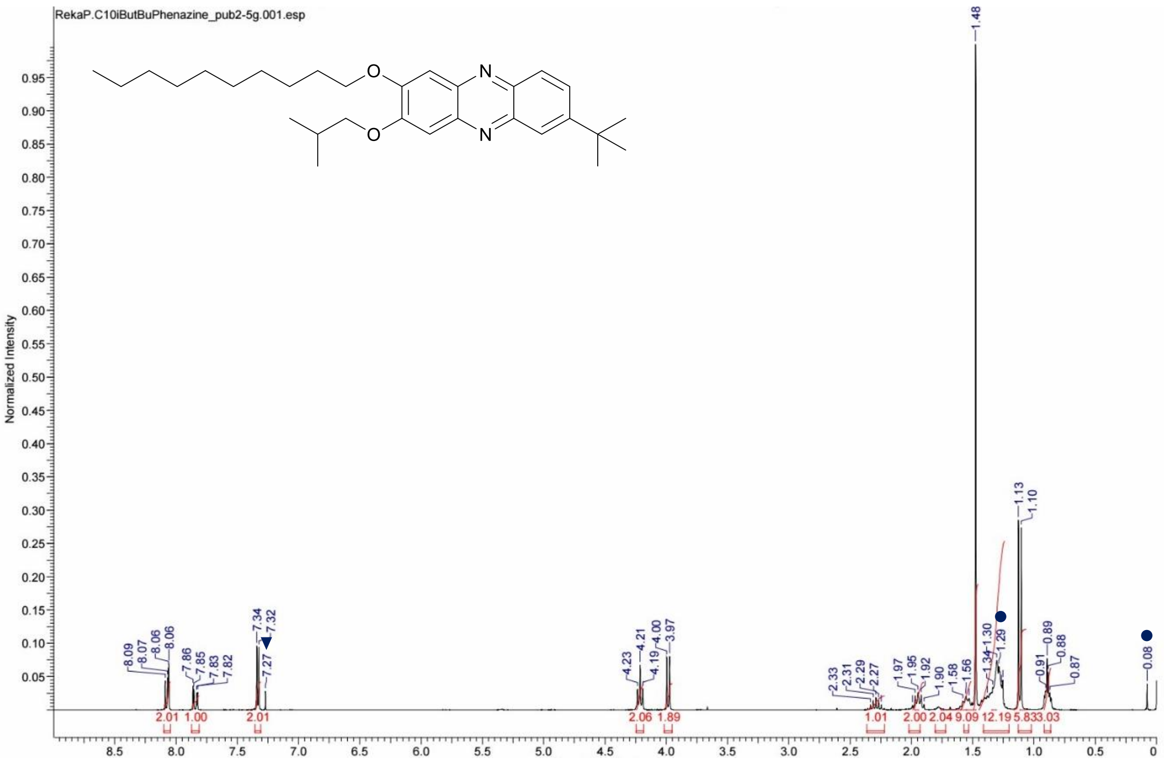

Figure S105. ^{1}H NMR ($CDCl_3$, 300 MHz, δ ppm) spectrum of **5g.**

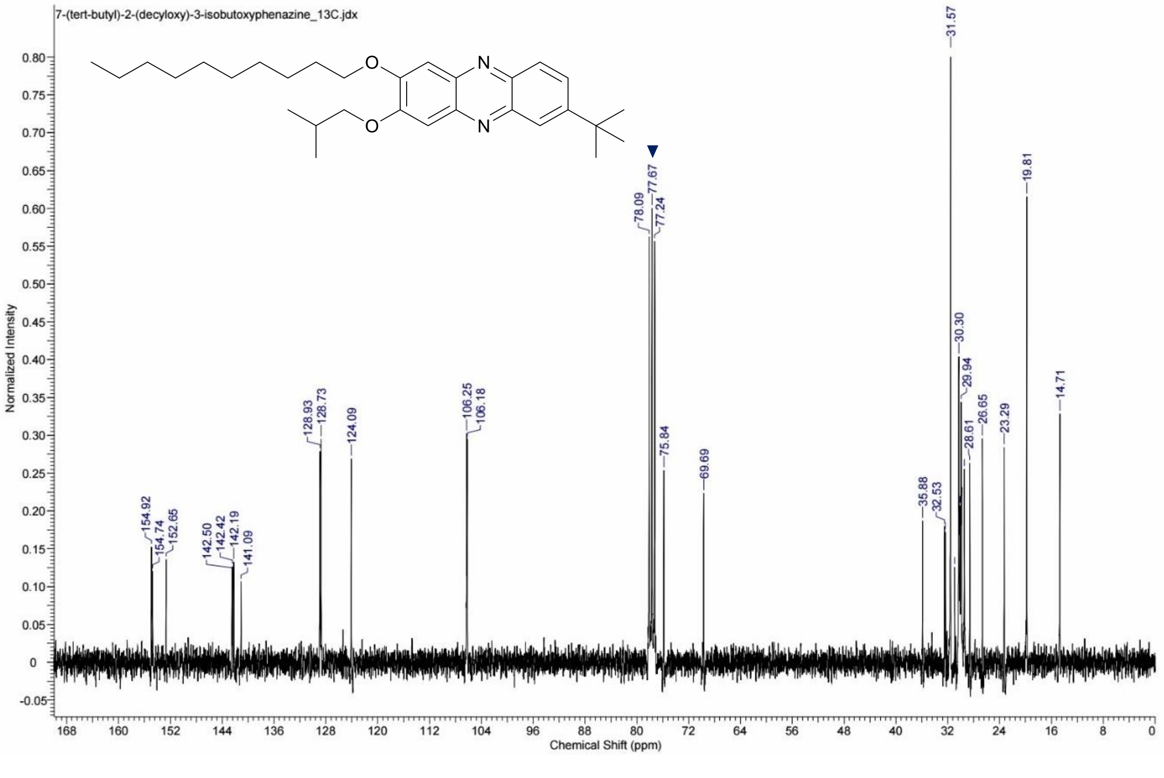

Figure S106. $^{13}C\{^{1}H\}$ NMR ($CDCl_3$, 75 MHz, δ ppm) NMR spectrum of **5g.**

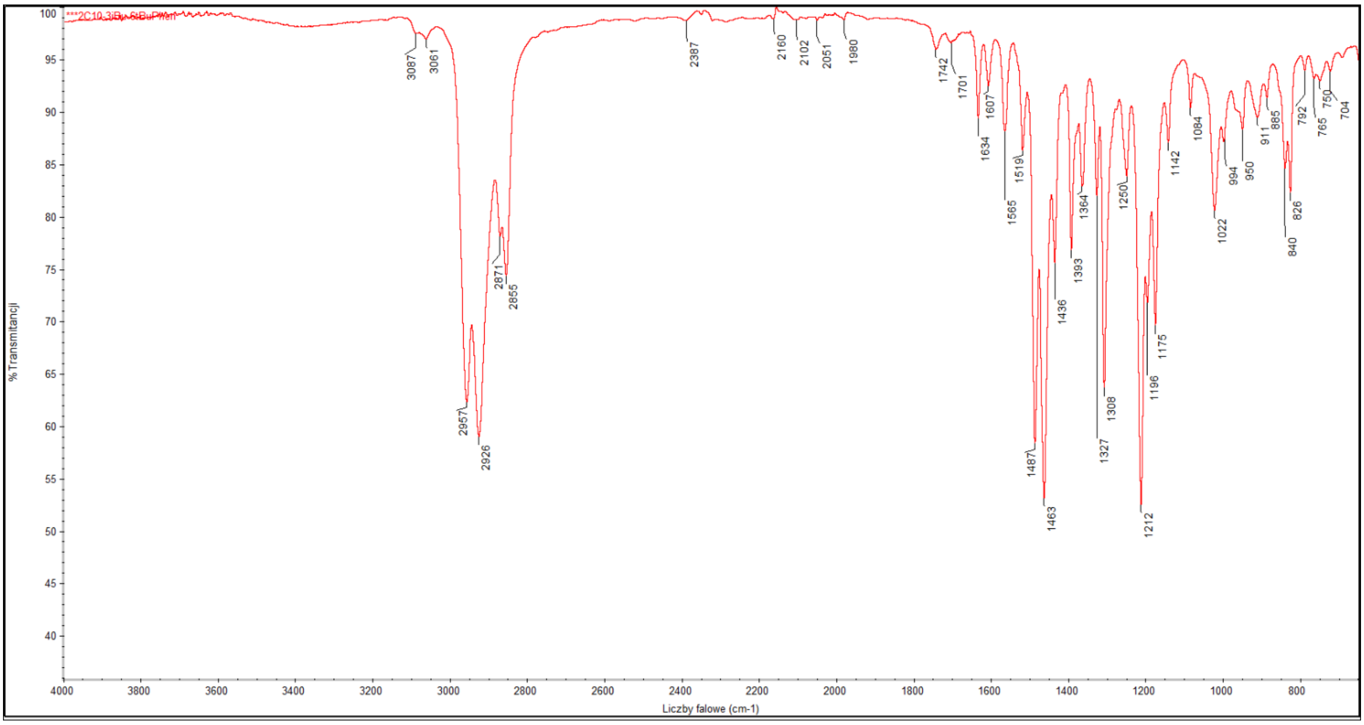

Figure S107. IR spectrum of **5g.**

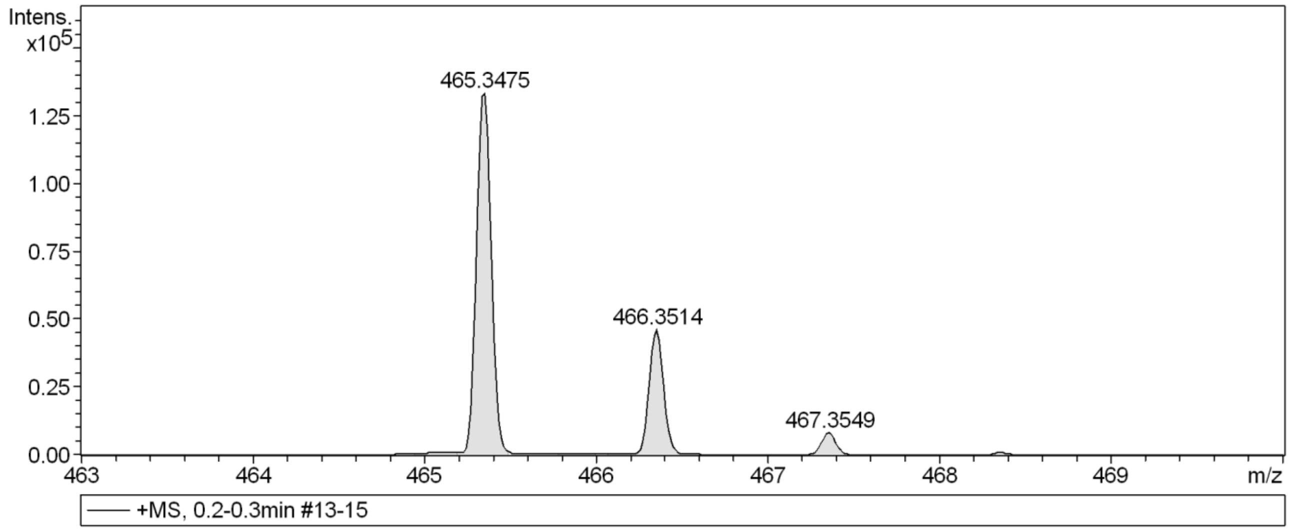

Figure S108. HRMS (ESI) spectrum of **5g**.

2-isobutoxy-3-methoxy-7-(trifluoromethyl)phenazine (6a).

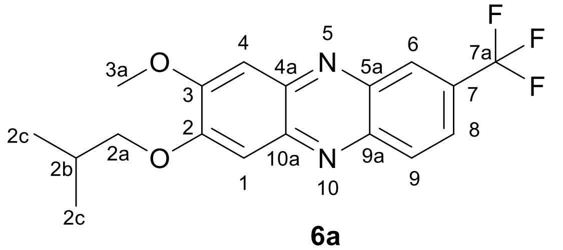

6a

^{1}H NMR ($CDCl_3$, 300 MHz, δ ppm): 8.48 (d, $^{4}J_{H6–H8}$ = 2.11 Hz, 1H, H_6), 8.25 (d, $^{3}J_{H8–H9}$ = 9.06 Hz, 1H, H_9), 7.88 (dd, $^{3}J_{H8–H9}$ = 9.06 Hz, $^{4}J_{H8–H6}$ = 2.11 Hz, 1H, H_8), 7.39 (s, 2H, $H_{1,4}$), 4.12 (s, 3H, H_{3a}), 4.03 (d, $^{3}J_{H2a–H2b}$ = 6.83 Hz, 2H, H_{2a}), 2.38 – 2.29 (m, 1H, H_{2b}), 1.13 (d, $^{3}J_{H2b–H2c}$ = 6.69 Hz, 6H, H_{2c}).

^{13}C{^{1}H} NMR ($CDCl_3$, 75 MHz, δ ppm): 156.4 (C_2), 156.0 (C_3), 144.0 and 143.5 ($C_{4a,10a}$), 143.1 (C_7), 141.0 (C_{9a}), 130.8 (C_9), 127.7 (q, $^{3}J_{C–F}$ = 4 Hz, C_6), 124.7 (q, $^{3}J_{C–F}$ = 3 Hz, C_8), 106.1 (C_1), 105.6 (C_4), 76.4 (C_{2a}), 57.2 (C_{3a}), 28.5 (C_{2b}), 19.6 (C_{2c}), signals from C_{7a} and C_{5a} are missing.

^{19}F NMR ($CDCl_3$, 282 MHz, δ ppm): –63.84 (s, 3F, F_{CF3}).

FT-IR (ATR, ν_{max}, (neat)/cm^{-1}): 3092, 3031, 2975, 2963, 2917, 2875, 2851, 2836, 1642, 1612, 1567, 1527, 1488, 1464, 1448, 1429, 1417, 1394, 1369, 1341, 1327, 1283, 1269, 1255, 1221, 1194, 1162, 1141, 1111, 1052, 1014, 972, 959, 942, 905, 893, 837, 825, 790, 750, 735.

HRMS (ESI) *m/z* Calculated for $C_{18}H_{18}N_2O_2F_3$ [M+H]$^+$, 351.1315; found: 351.1313.

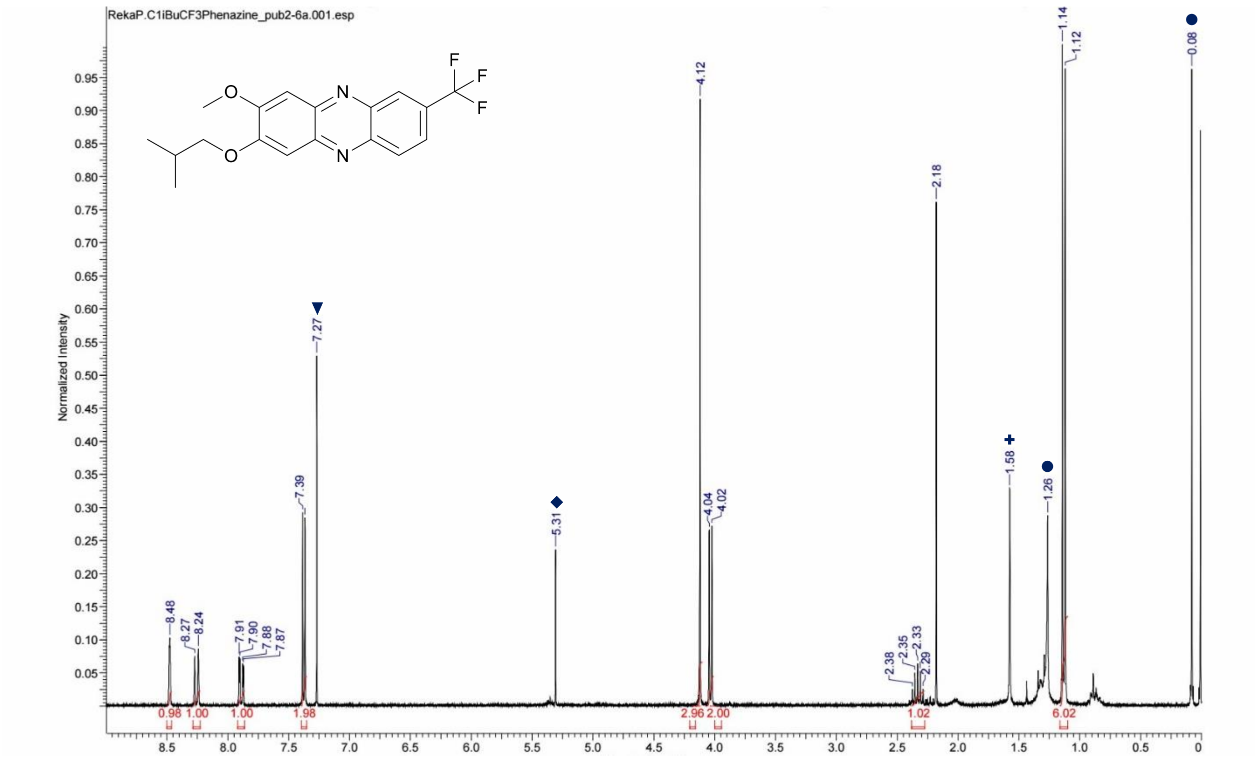

Figure S109. ^{1}H NMR ($CDCl_3$, 300 MHz, δ ppm) spectrum of **6a.**

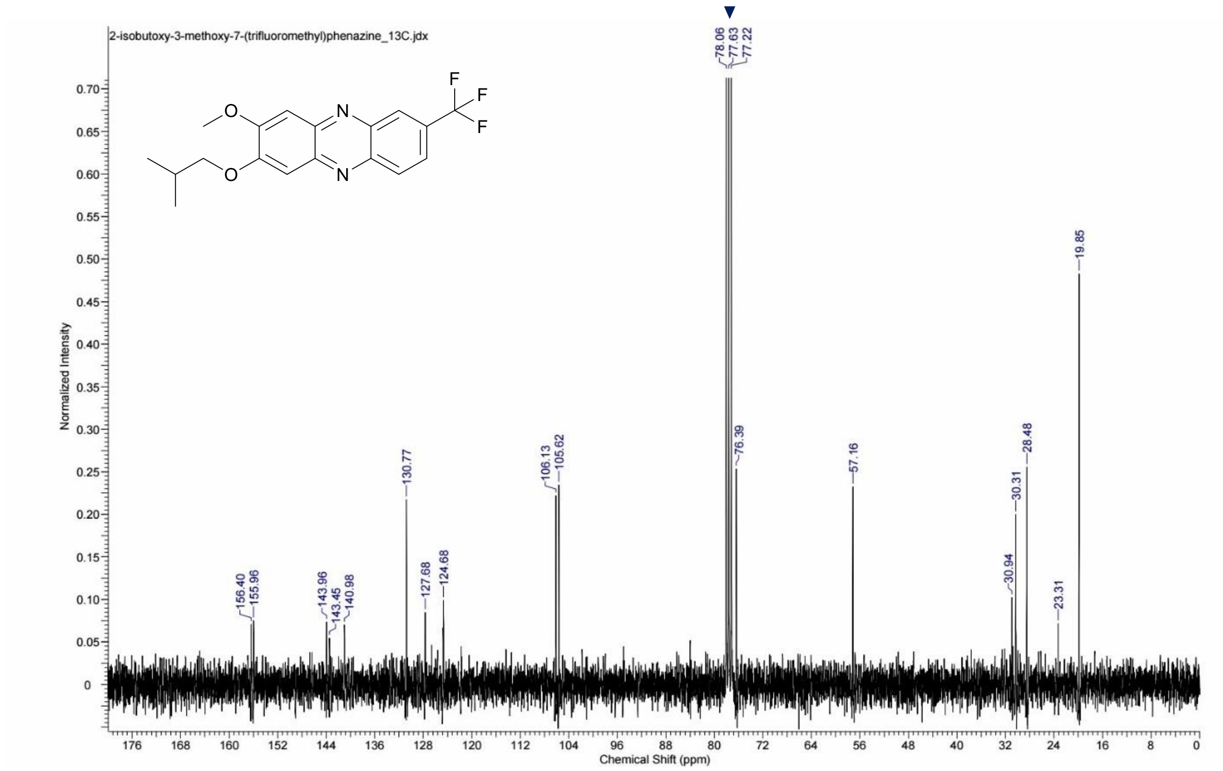

Figure S110. $^{13}C\{^1H\}$ NMR ($CDCl_3$, 75 MHz, δ ppm) NMR spectrum of **6a.**

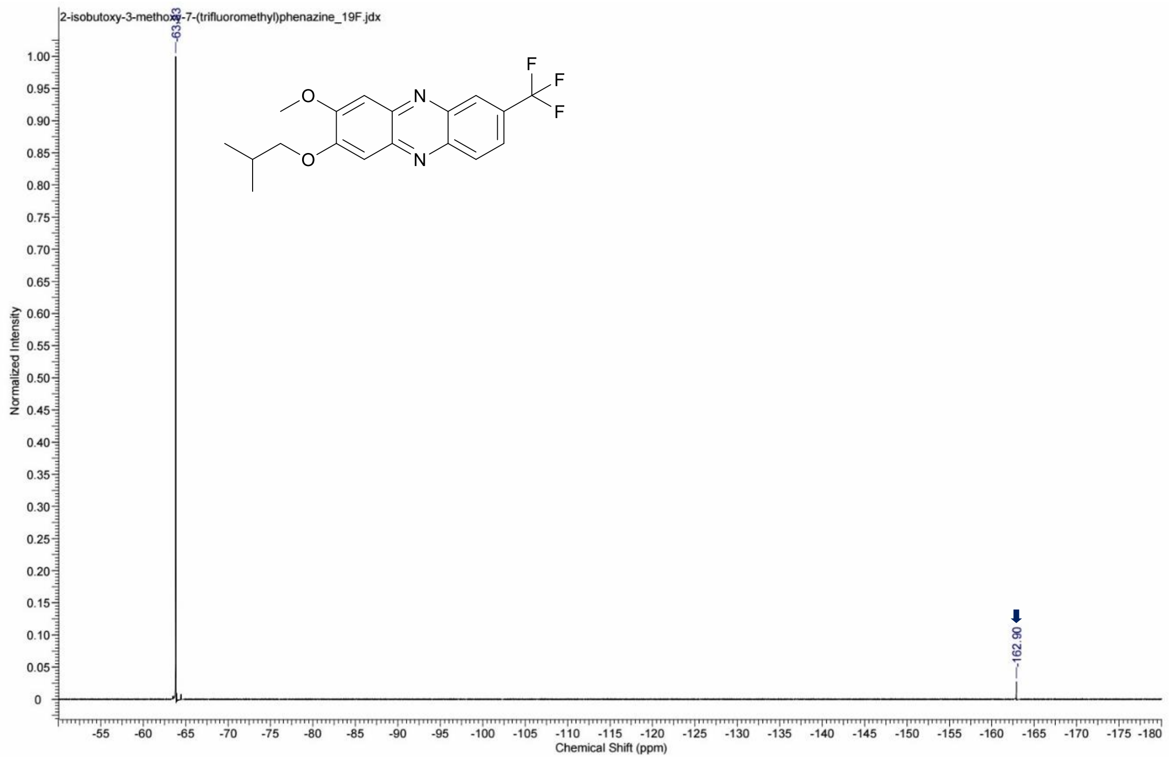

Figure S111. ^{19}F ($CDCl_3$, 282 MHz, δ ppm) NMR spectrum of **6a.**

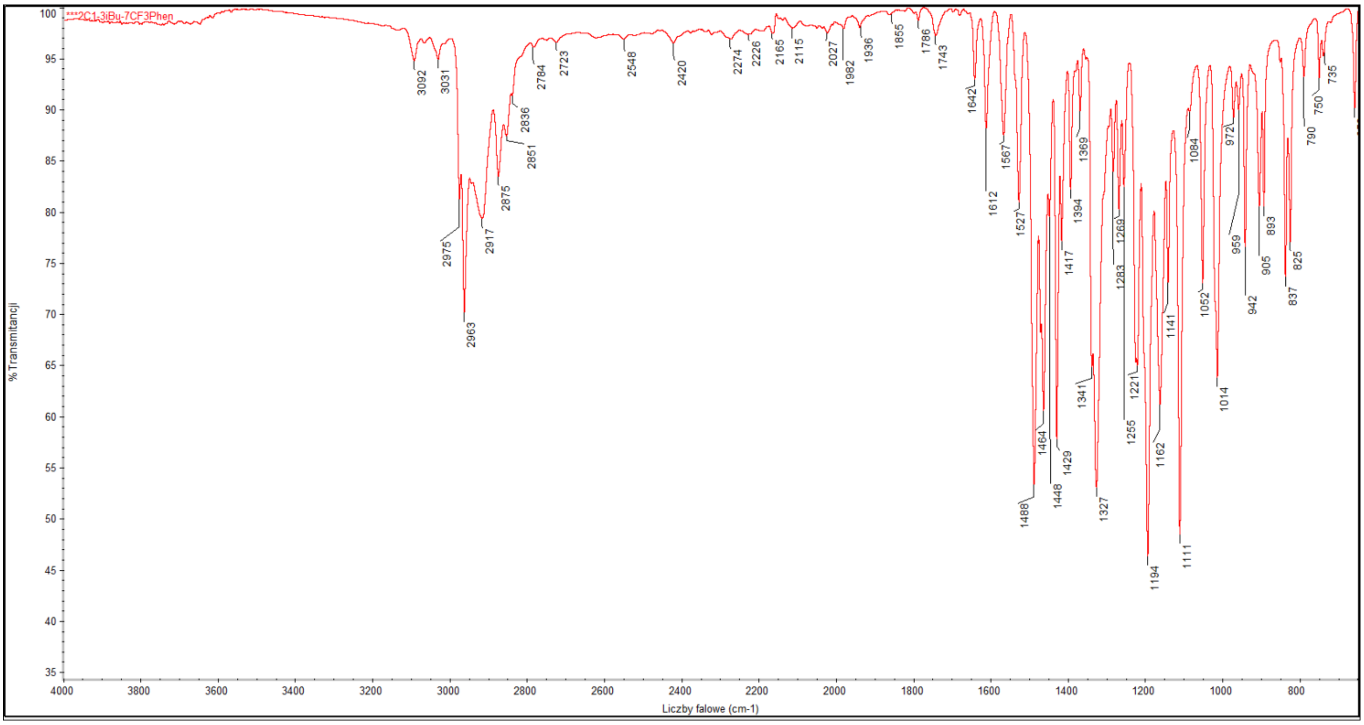

Figure S112. IR spectrum of **6a.**

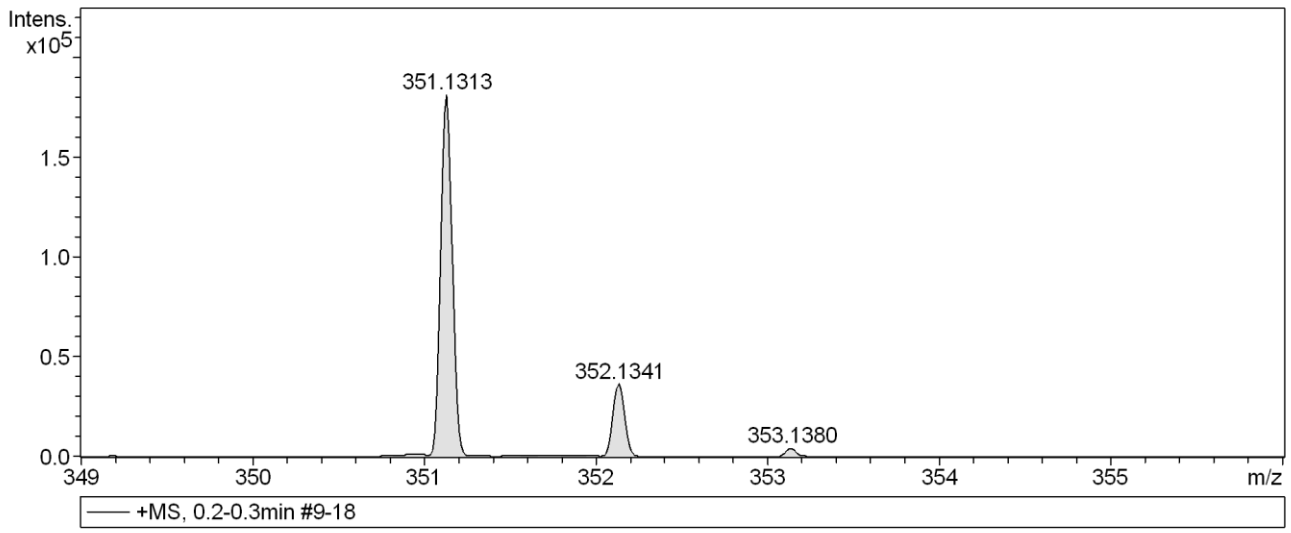

Figure S113. HRMS (ESI) spectrum of **6a**.

3-ethoxy-2-isobutoxy-7-(trifluoromethyl)phenazine (6b).

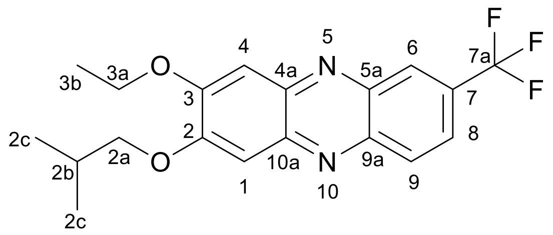

6b

^{1}H NMR ($CDCl_3$, 300 MHz, δ ppm): 8.46 (d, $^{4}J_{H6–H8}$ = 2.09 Hz, 1H, H_6), 8.23 (d, $^{3}J_{H8–H9}$=9.00Hz, 1H, H_9), 7.87 (dd, $^{3}J_{H8–H9}$ = 9.00 Hz, $^{4}J_{H8–H6}$ = 2.09 Hz, 1H, H_8), 7.33 (s, 2H, $H_{1,4}$), 4.33 (q, $^{3}J_{H3a–H3b}$ = 6.95 Hz, 2H, H_{3a}), 4.01 (d, $^{3}J_{H2a–H2b}$ = 6.68 Hz, 2H, H_{2a}), 2.31 (m, 1H, H_{2b}), 1.60 (t, $^{3}J_{H3a–H2b}$ = 6.89 Hz, 3H, H_{3b}), 1.13 (d, $^{3}J_{H2b–H2c}$ = 6.83 Hz, 6H, H_{2c}).

^{13}C{^{1}H} NMR ($CDCl_3$, 75 MHz, δ ppm): 156.2 (C_2), 155.8 (C_3), 143.9 and 143.6 ($C_{4a,10a}$), 143.0 (C_7), 141.0 (C_{9a}), 130.8 (C_9), 127.7 (q, $^{3}J_{C–F}$ = 4 Hz, C_6), 124.6 (q, $^{3}J_{C–F}$ = 3 Hz, C_8), 106.1 ($C_{1,4}$), 76.2 (C_{2a}), 65.6 (C_{3a}), 28.6 (C_{2b}), 19.8 (C_{2c}), 15.0 (C_{3b}), signals from C_{7a} and C_{5a} are missing.

^{19}F NMR ($CDCl_3$, 282 MHz, δ ppm): –63.84 (s, 3F, F_{CF3}).

FT-IR (ATR, ν_{max}, (neat)/cm^{-1}): 3109, 3032, 3015, 2960, 2927, 2875, 2851, 1640, 1611, 1566, 1525, 1490, 1466, 1451, 1417, 1392, 1367, 1337, 1325, 1283, 1267, 1253, 1219, 1201, 1184, 1153, 1142, 1110, 1058, 1044, 1016, 972, 943, 933, 908, 889, 850, 822, 789, 751.

HRMS (ESI) *m/z* Calculated for $C_{19}H_{20}N_2O_2F_3$ [M+H]$^+$, 365.1472; found: 365.1469.

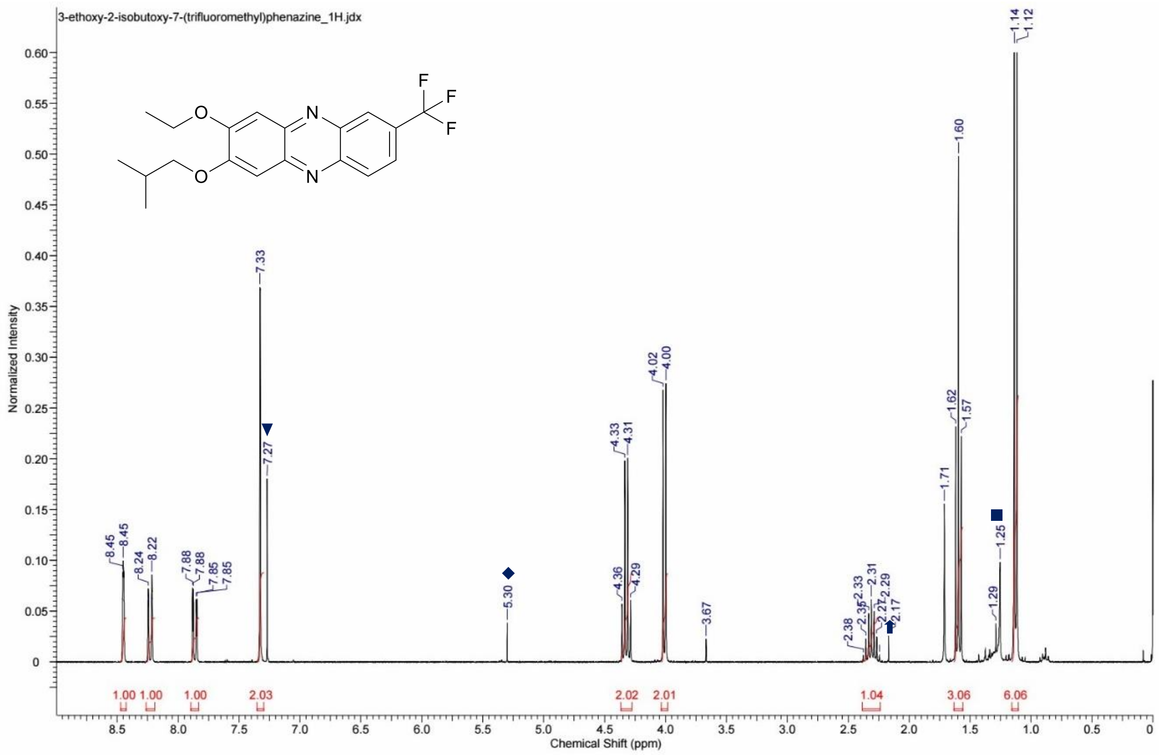

Figure S114. ^{1}H NMR ($CDCl_3$, 300 MHz, δ ppm) spectrum of **6b.**

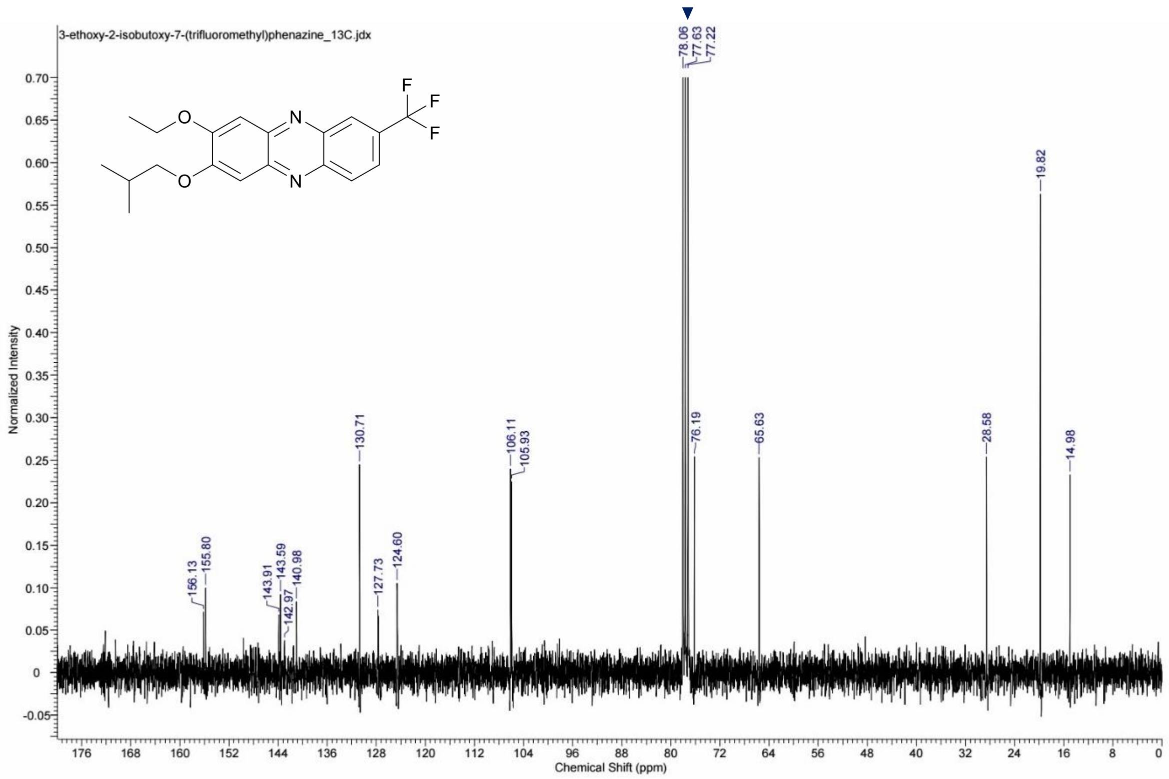

Figure S115. $^{13}C\{^{1}H\}$ NMR ($CDCl_3$, 75 MHz, δ ppm) NMR spectrum of **6b.**

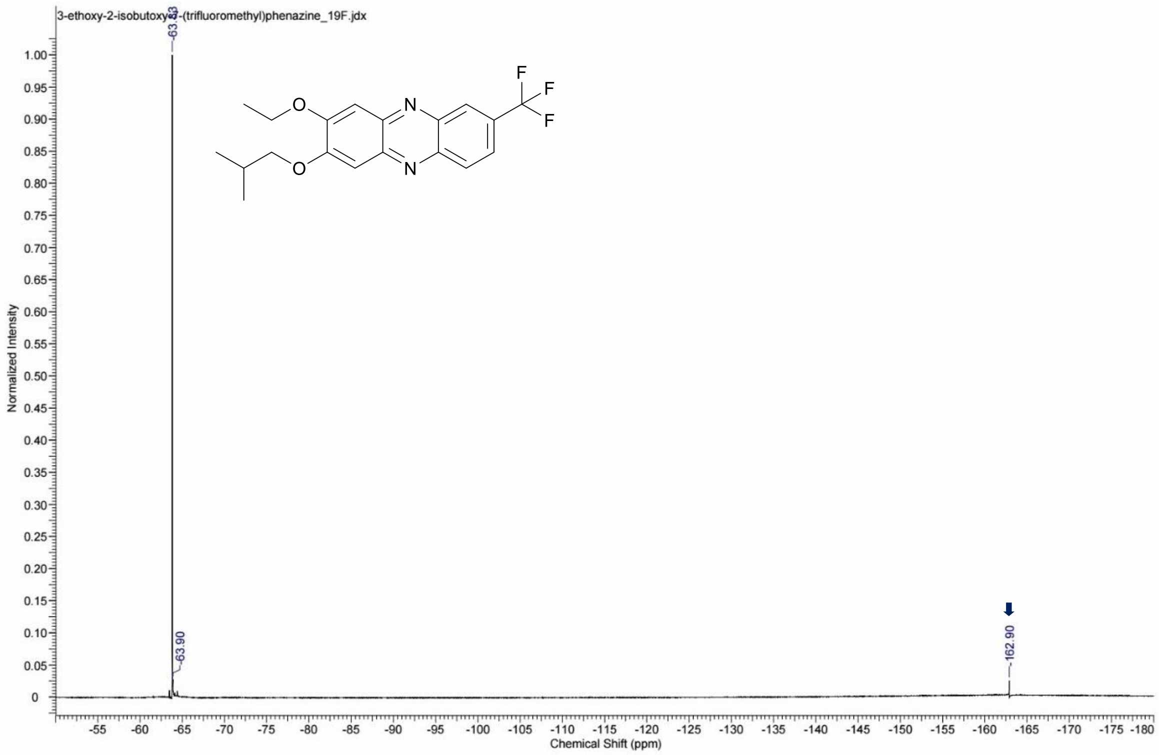

Figure S116. ^{19}F ($CDCl_3$, 282 MHz, δ ppm) NMR spectrum of **6b.**

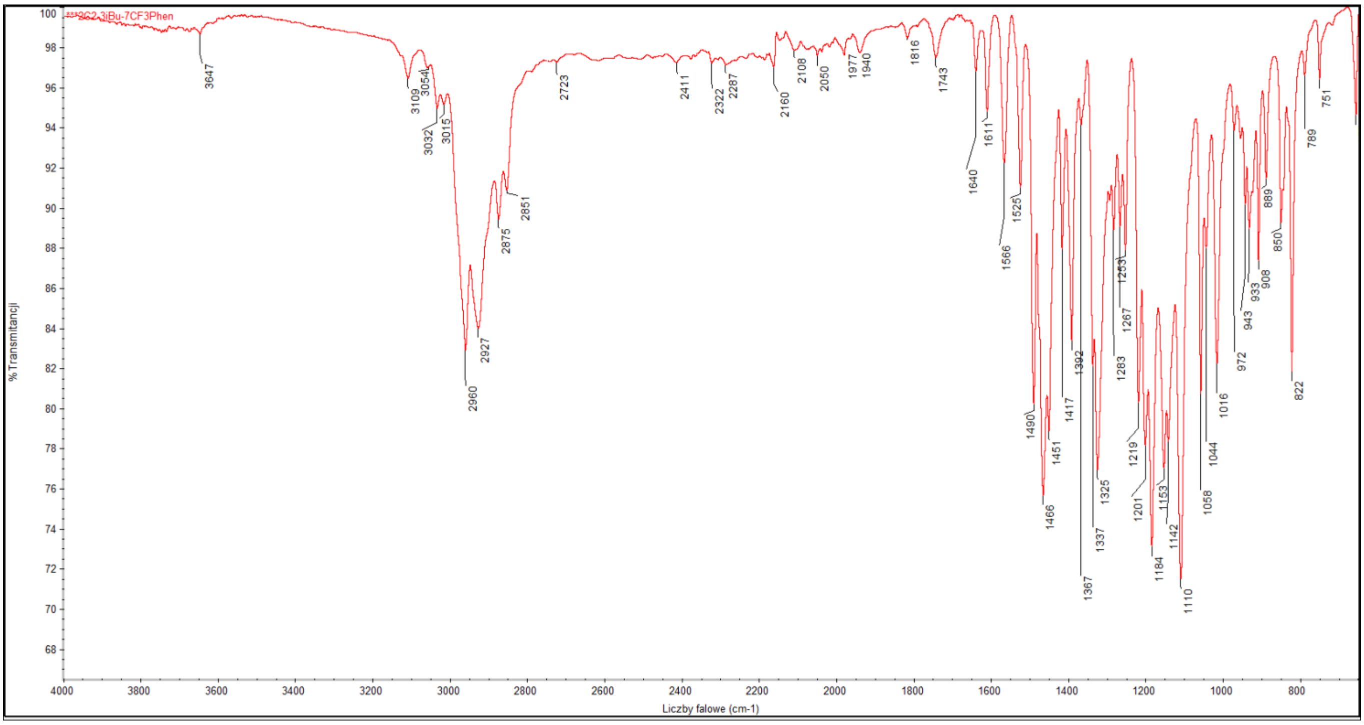

Figure S117. IR spectrum of **6b.**

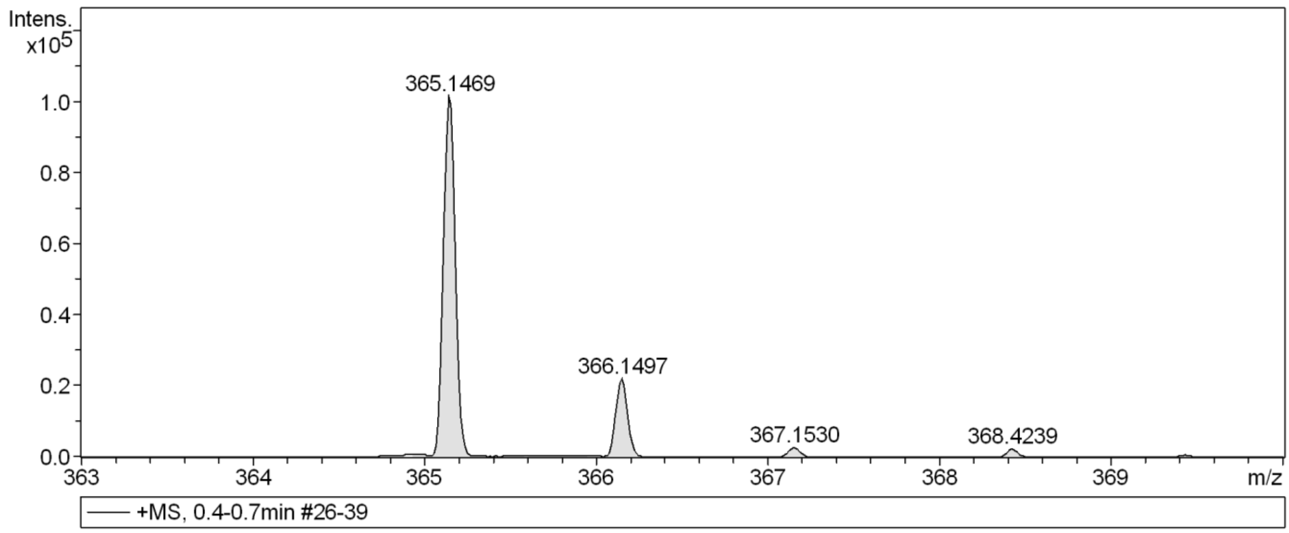

Figure S118. HRMS (ESI) spectrum of **6b**.

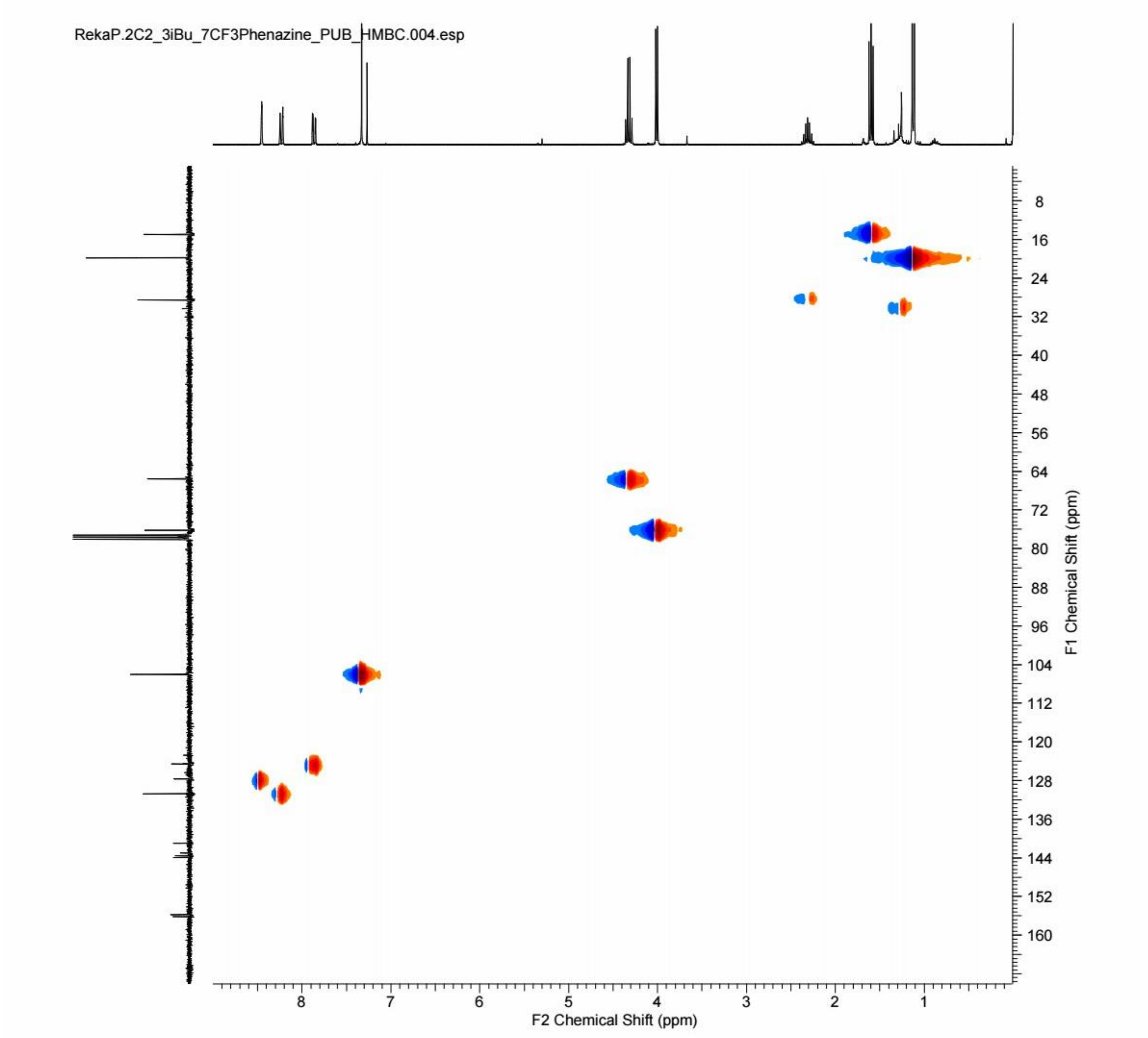

Figure S119. HSQC spectrum of **6b.**

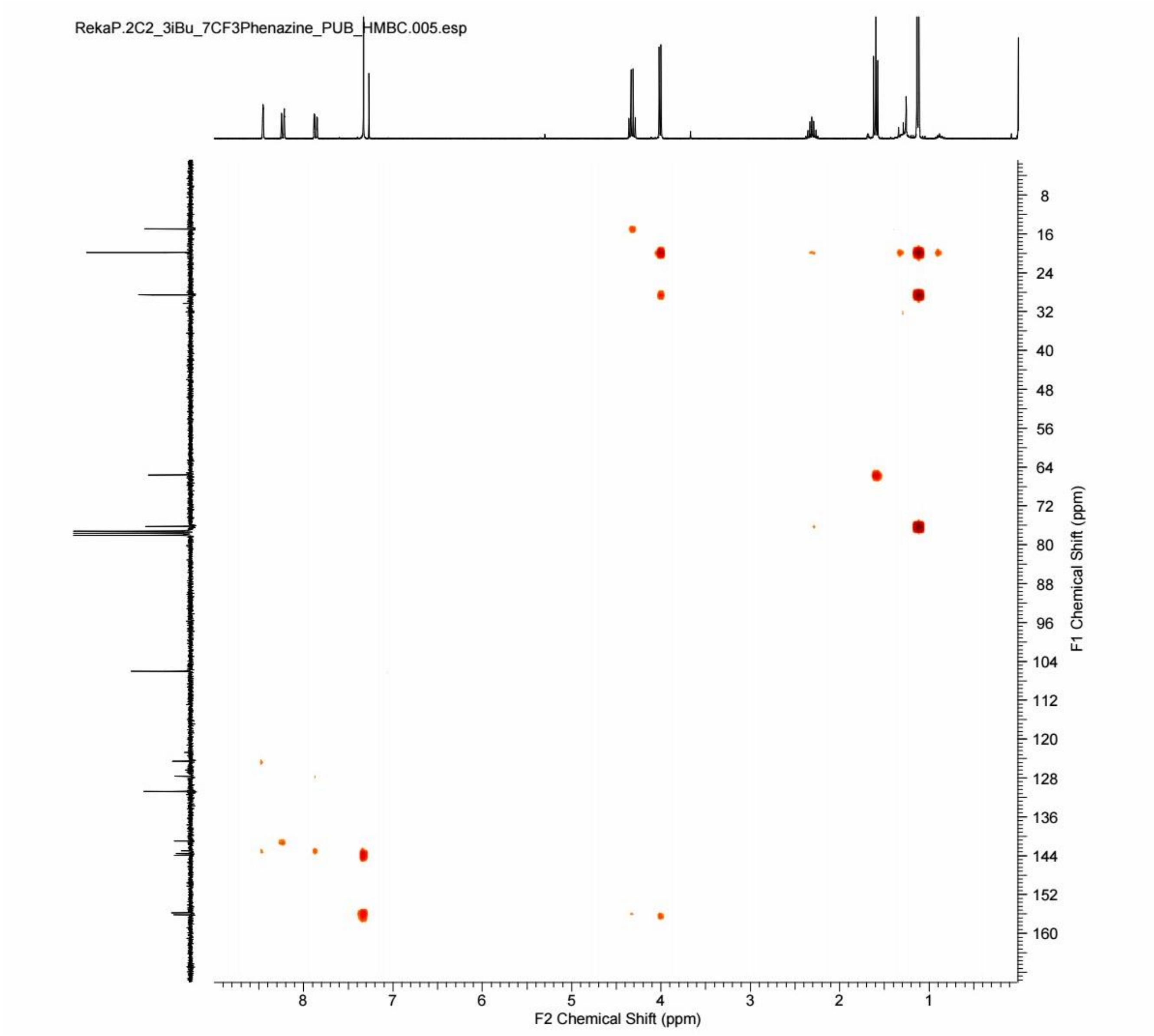

Figure S120. HMBC spectrum of **6b.**

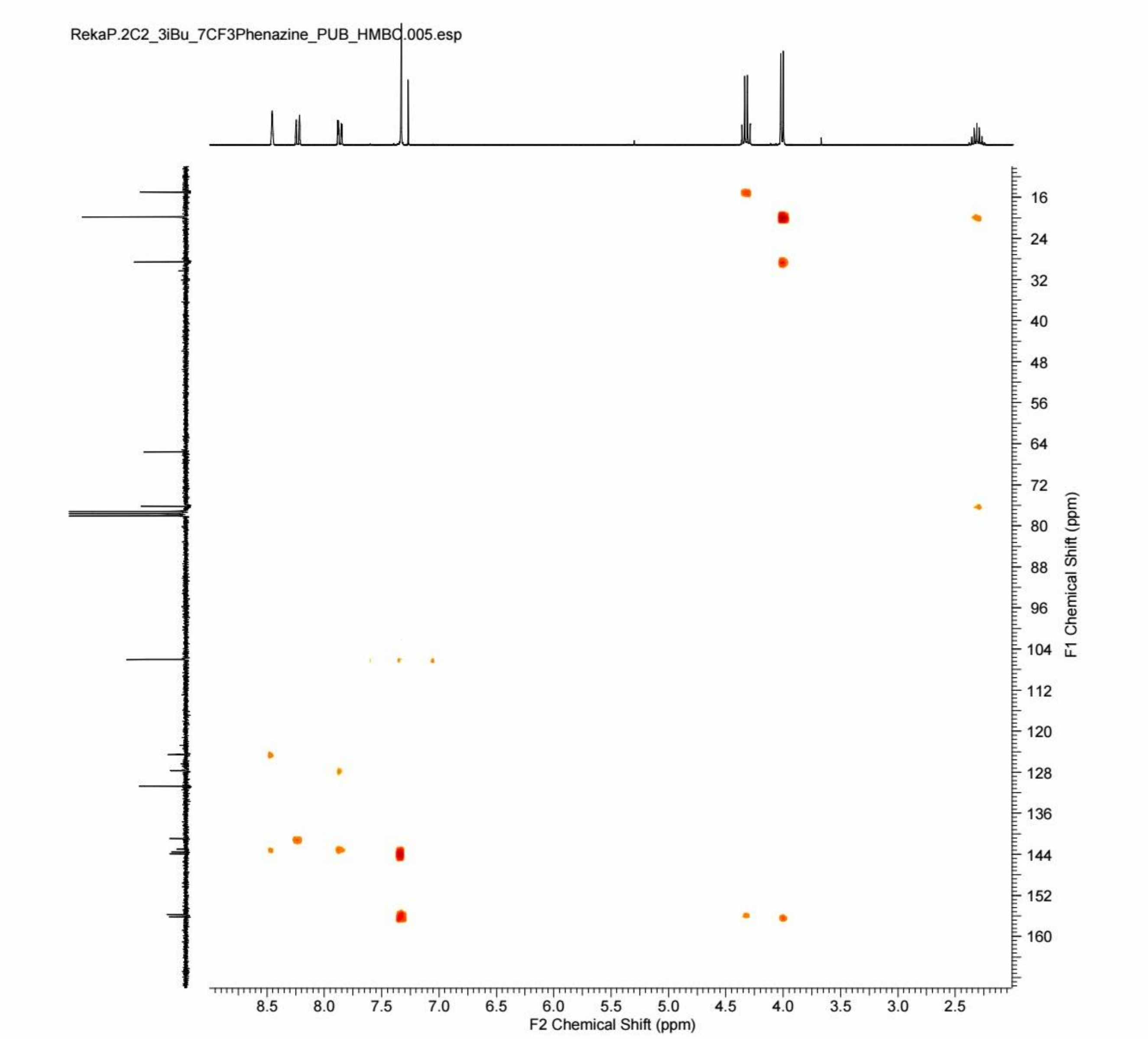

Figure S121. Zoomed region of HMBC spectrum of **6b.**

2-ethoxy-3-isobutoxy-7-(trifluoromethyl)phenazine (6c).

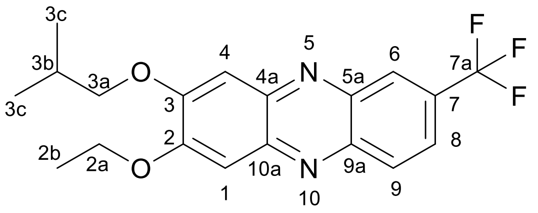

6c

^{1}H NMR ($CDCl_3$, 300 MHz, δ ppm): 8.46 (d, $^{4}J_{H6–H8}$ = 2.09 Hz, 1H, H_6), 8.23 (d, $^{3}J_{H8–H9}$ = 9.00 Hz, 1H, H_9), 7.87 (dd, $^{3}J_{H8–H9}$ = 9.00 Hz, $^{4}J_{H8–H6}$ = 2.09 Hz, 1H, H_8), 7.33 (s, 2H, $H_{1,4}$), 4.33 (q, $^{3}J_{H2a–H2b}$ = 6.95 Hz, 2H, H_{2a}), 4.01 (d, $^{3}J_{H3a–H3b}$ = 6.68 Hz, 2H, H_{3a}), 2.31 (m, 1H, H_{3b}), 1.60 (t, $^{3}J_{H2a–H2b}$ = 6.89 Hz, 3H, H_{2b}), 1.13 (d, $^{3}J_{H3b–H3c}$ = 6.83 Hz, 6H, H_{3c}).

^{13}C{^{1}H} NMR ($CDCl_3$, 75 MHz, δ ppm): 156.2 (C_2), 155.8 (C_3), 143.9 and 143.6 ($C_{4a,10a}$), 143.0 (C_7), 141.0 (C_{9a}), 130.8 (C_9), 127.7 (q, $^{3}J_{C–F}$ = 4 Hz, C_6), 124.6 (q, $^{3}J_{C–F}$ = 3 Hz, C_8), 106.1 ($C_{1,4}$), 76.2 (C_{3a}), 65.6 (C_{2a}), 28.6 (C_{3b}), 19.8 (C_{3c}), 15.0 (C_{2b}), signals from C_{7a} and C_{5a} are missing.

^{19}F NMR ($CDCl_3$, 282 MHz, δ ppm): –63.83 (s, 3F, F_{CF3}).

FT-IR (ATR, ν_{max}, (neat)/cm^{-1}): 3105, 3039, 2965, 2936, 2897, 2875, 1641, 1611, 1572, 1524, 1490, 1468, 1451, 1417, 1393, 1368, 1338, 1326, 1301, 1283, 1283, 1267, 1251, 1220, 1189, 1178, 1150, 1141, 1102, 1057, 1043, 1024, 1000, 946, 928, 904, 890, 849, 928, 904, 890, 849, 830, 787, 751.

HRMS (ESI) *m/z* Calculated for $C_{19}H_{20}N_2O_2F_3$ $[M+H]^+$, 365.1472; found: 365.1471.

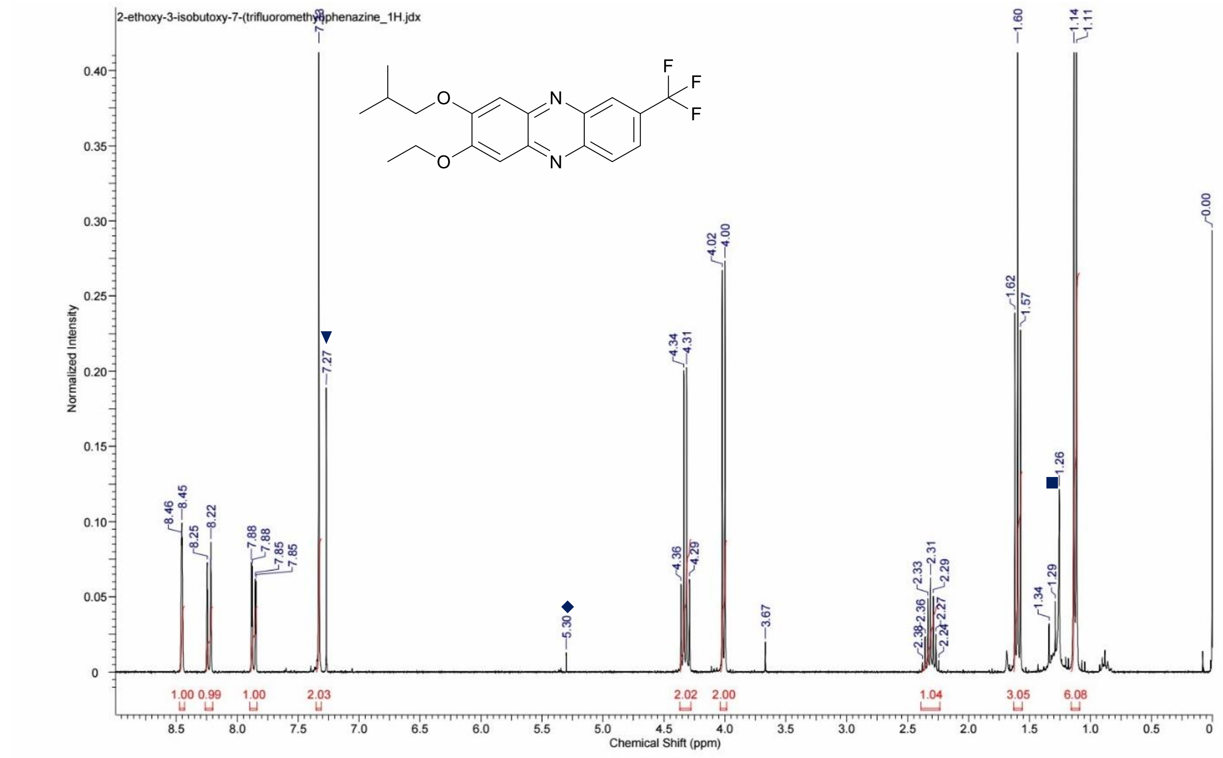

Figure S122. ^{1}H NMR ($CDCl_3$, 300 MHz, δ ppm) spectrum of **6c.**

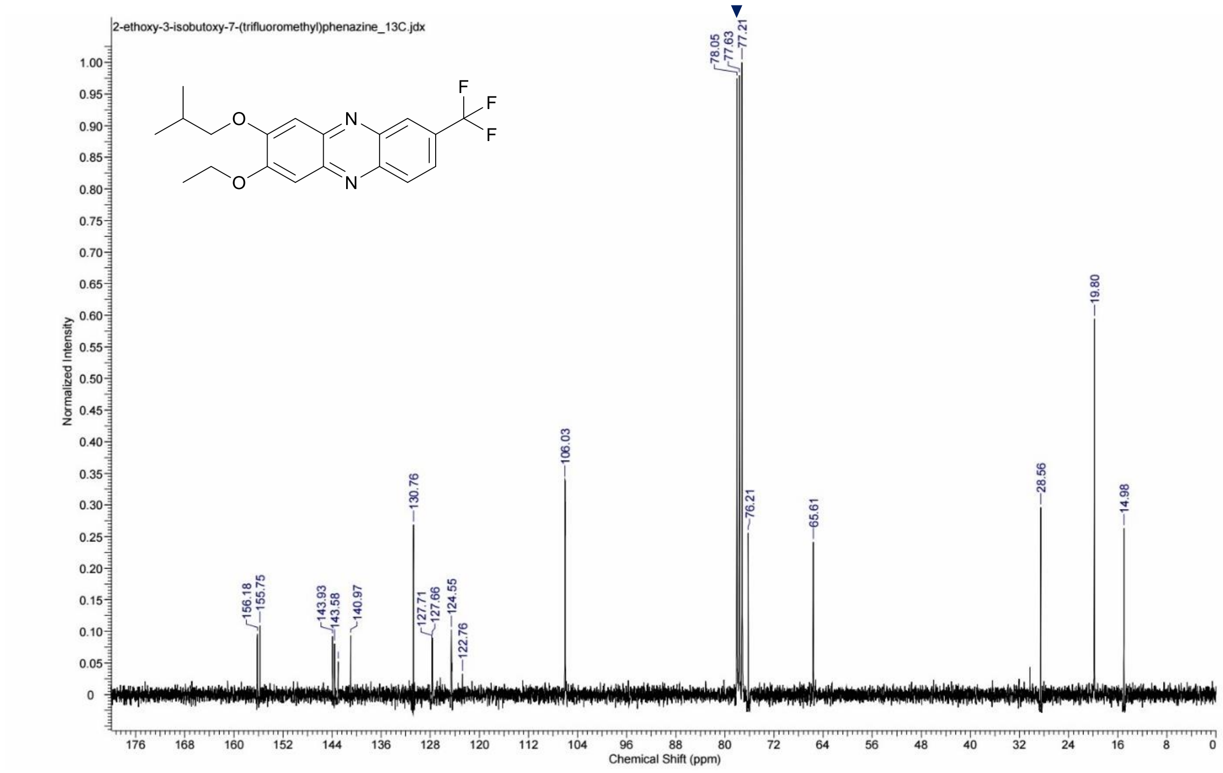

Figure S123. $^{13}C\{^{1}H\}$ NMR ($CDCl_3$, 75 MHz, δ ppm) NMR spectrum of **6c.**

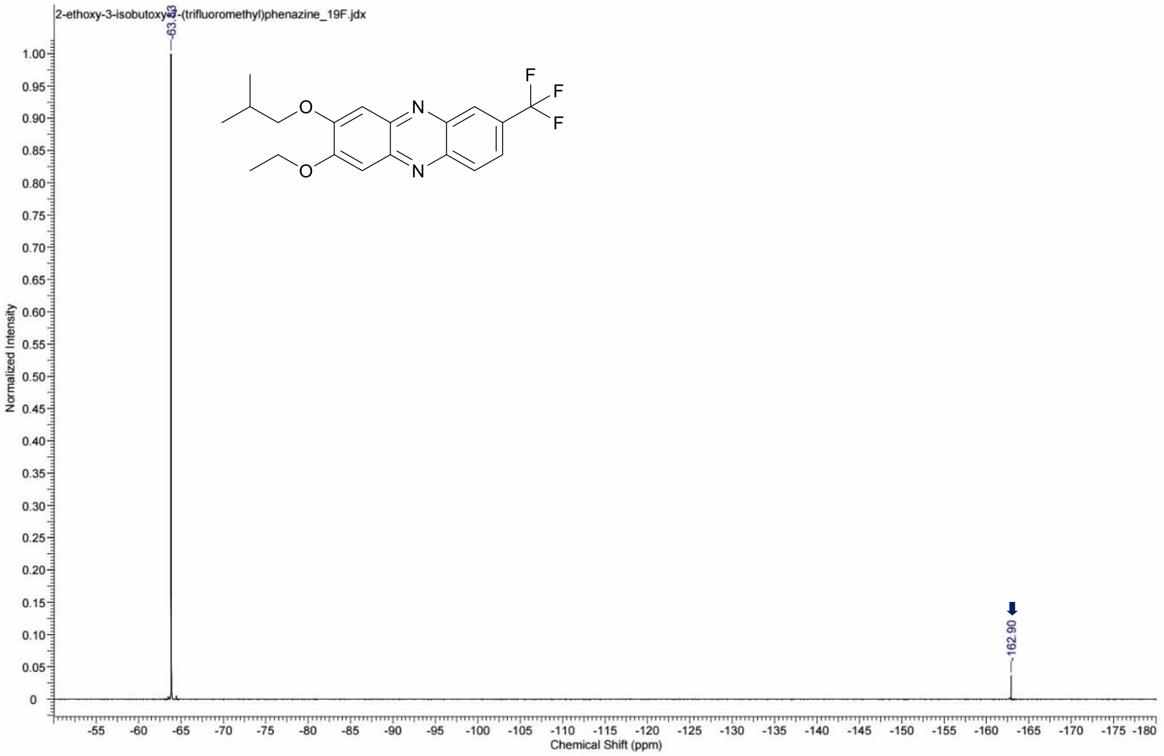

Figure S124. ^{19}F NMR ($CDCl_3$, 282 MHz, δ ppm) spectrum of **6c.**

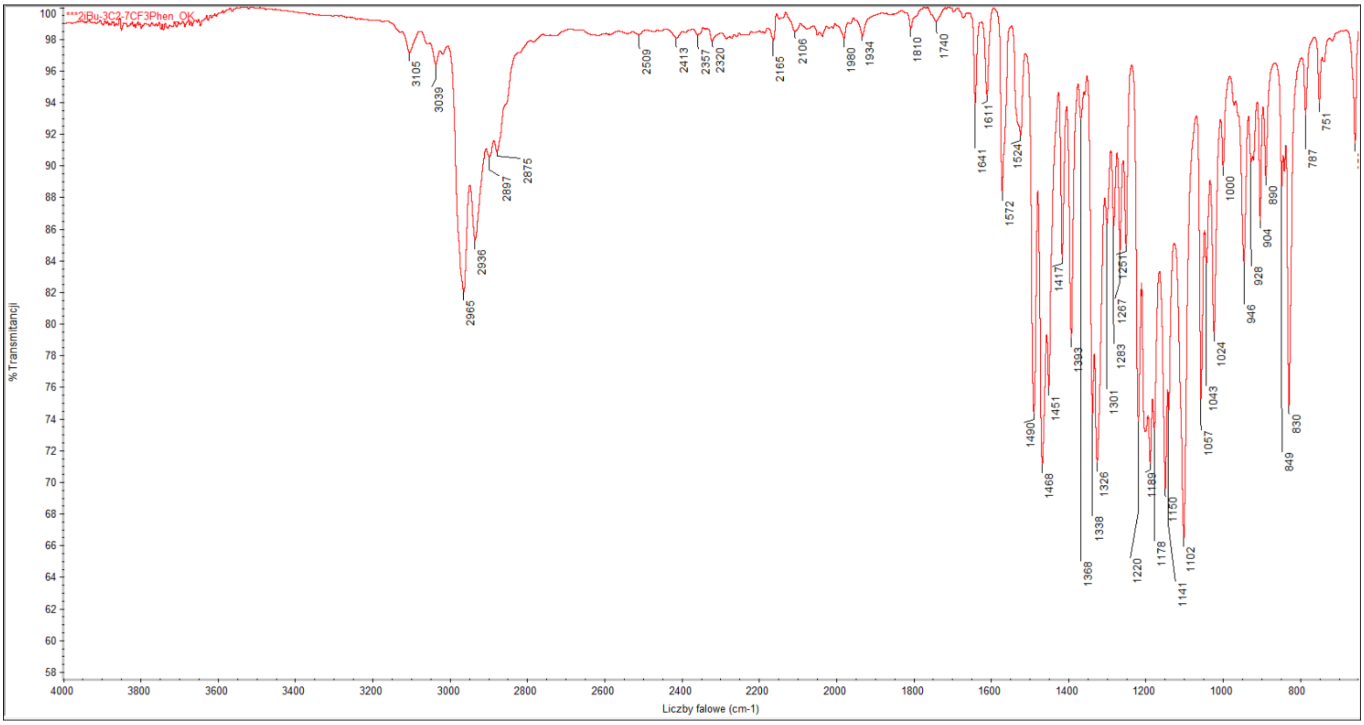

Figure S125. IR spectrum of **6c.**

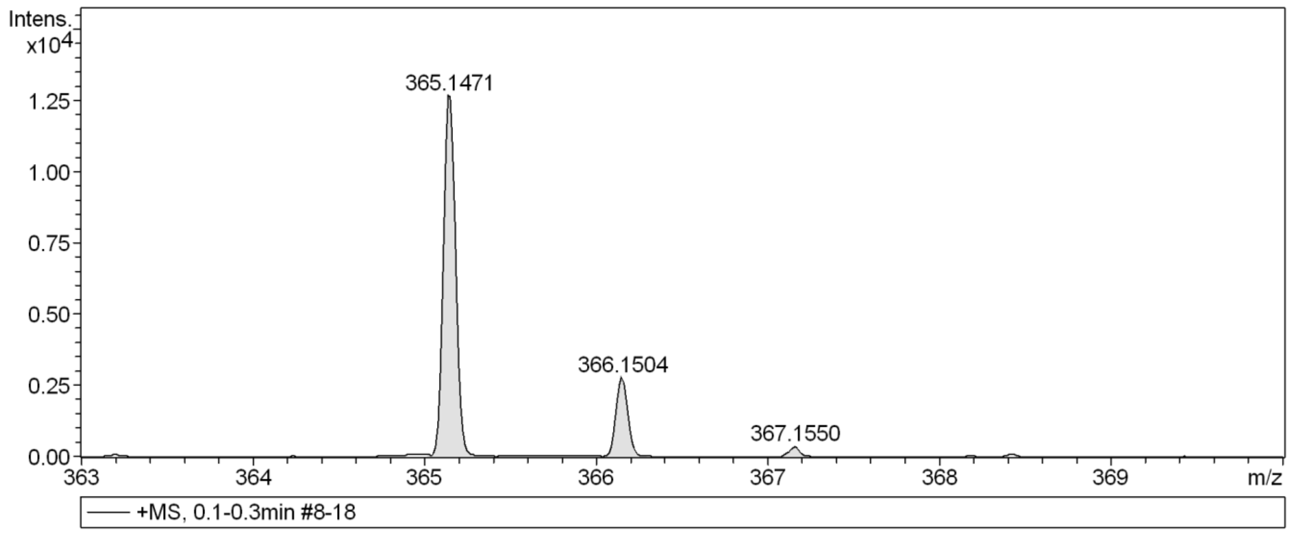

Figure S126. HRMS (ESI) spectrum of **6c**.

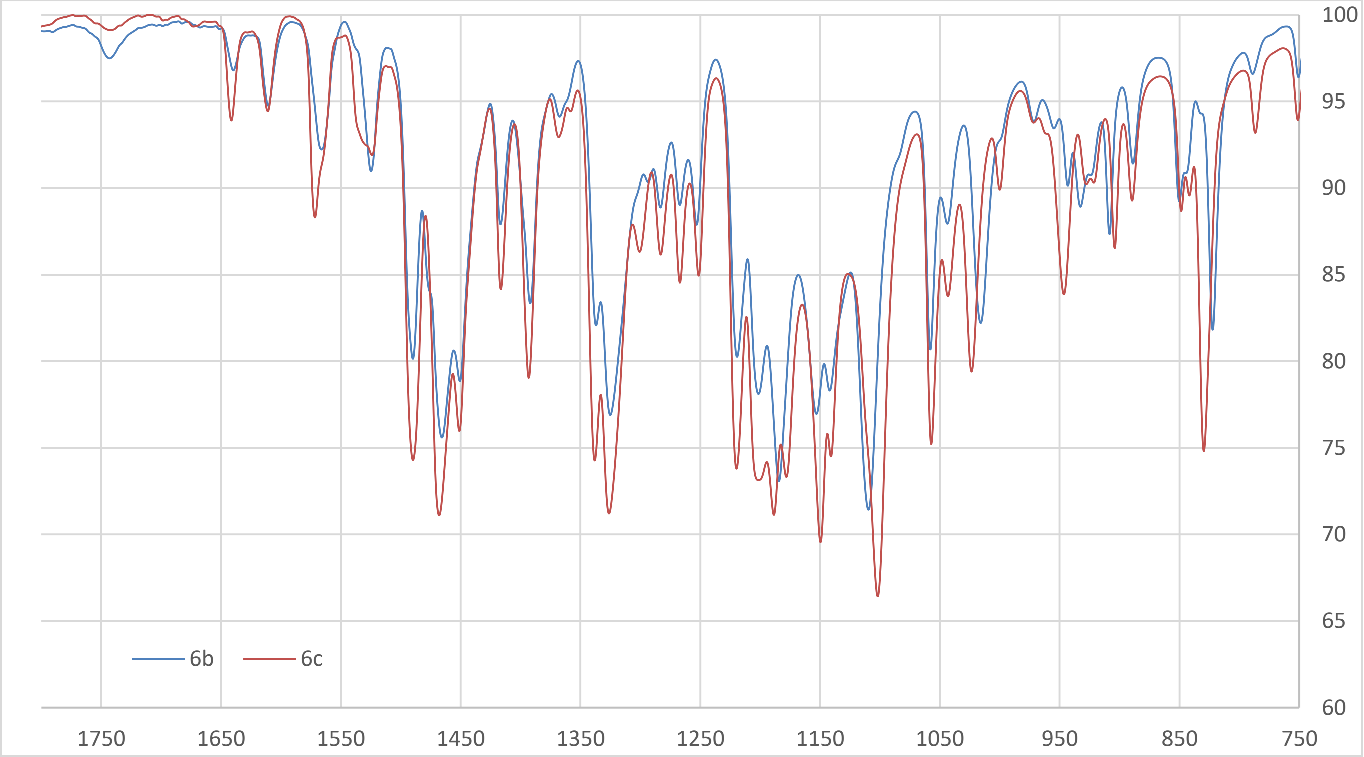

Figure S127. Comparison of IR spectra of compounds **6b** and **6c**.

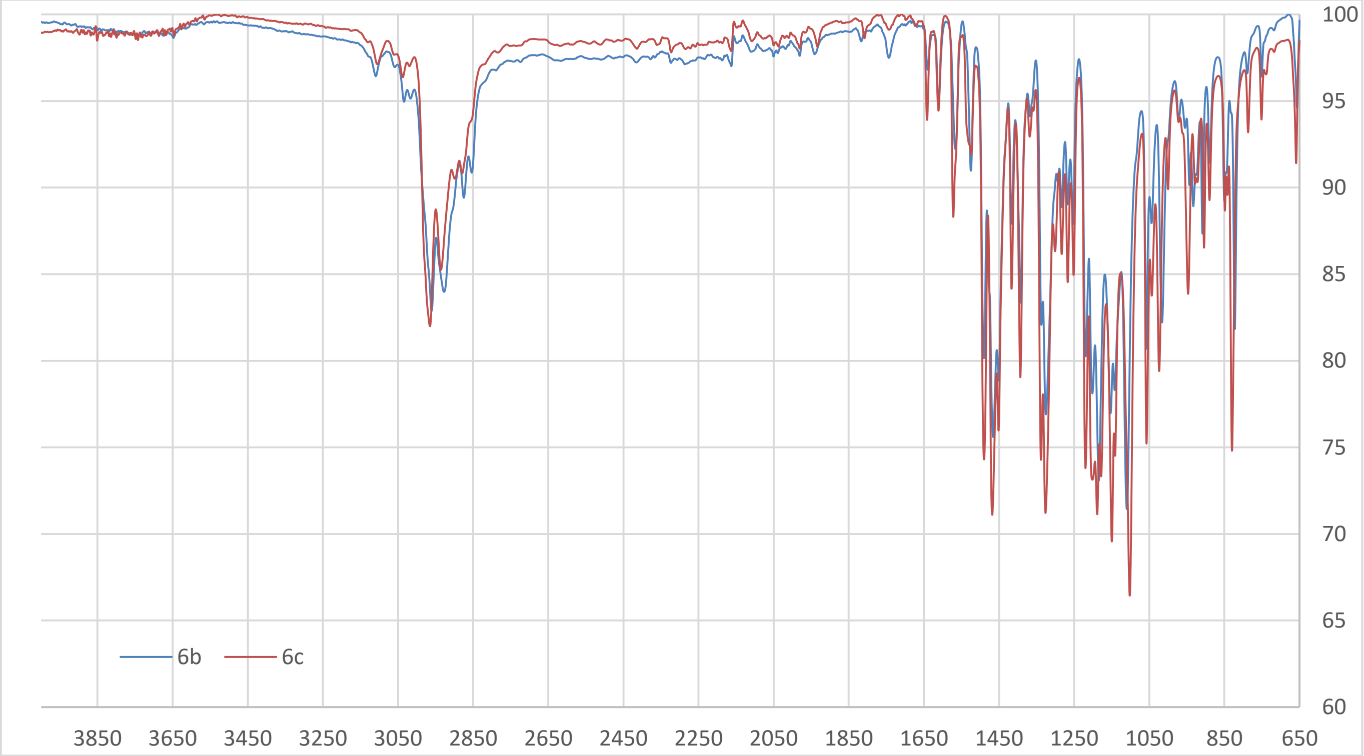

Figure S128. Comparison of IR spectra of compounds **6b** and **6c**, zoomed region.

3-butoxy-2-isobutoxy-7-(trifluoromethyl)phenazine (6d).

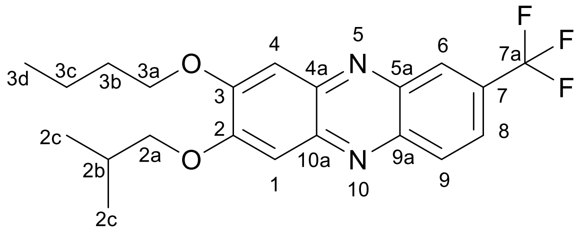

6d

^{1}H NMR ($CDCl_3$, 300 MHz, δ ppm): 8.46 (d, $^4J_{H6-H8}$ = 2.04 Hz, 1H, H_6), 8.24 (d, $^3J_{H8-H9}$ = 9.17 Hz, 1H, H_9), 7.87 (dd, $^3J_{H8-H9}$ = 9.17 Hz, $^4J_{H8-H6}$ = 2.04 Hz, 1H, H_8), 7.33 (2x s, 2x1H, $H_{1,4}$), 4.33 (q, $^3J_{H3a-H3b}$ = 6.95 Hz, 2H, H_{3a}), 4.01 (d, $^3J_{H2a-H2b}$ = 6.68 Hz, 2H, H_{2a}), 2.30 (m, 1H, H_{2b}), 2.01– 1.90 (m, 2H, H_{3b}), 1.64 – 1.54 (m, 2H, H_{3c}), 1.13 (d, $^3J_{H2b-H2c}$ = 6.78 Hz, 6H, H_{2c}), 1.05 (t, $^3J_{H3c-H3d}$ = 7.32 Hz, 3H, H_{3d}).

^{13}C{^{1}H} NMR ($CDCl_3$, 75 MHz, δ ppm): 156.3 (C_2), 156.0 (C_3), 144.0 and 143.6 ($C_{4a,10a}$), 143.0 (C_7), 141.0 (C_{9a}), 130.8 (C_9), 127.7 (q, $^3J_{C-F}$ = 4 Hz, C_6), 124.6 (q, $^3J_{C-F}$ = 3 Hz, C_8), 106.0 ($C_{4,1}$), 76.1 (C_{2a}), 69.7 (C_{3a}), 31.4 (C_{3b}), 28.6 (C_{2b}), 19.9 (C_{3c}), 19.8 (C_{2c}), 14.4 (C_{3d}), signals from C_{7a} and C_{5a} are missing.

^{19}F NMR ($CDCl_3$, 282 MHz, δ ppm): –63.81 (s, 3F, F_{CF3}).

FT-IR (ATR, ν_{max}, (neat)/cm^{-1}): 3106, 3034, 3015, 2957, 2932, 2874, 1640, 1611, 1570, 1524, 1489, 1466, 1451, 1417, 1392, 1367, 1341, 1327, 1283, 1267, 1254, 1219, 1203, 1187, 1152, 1141, 1103, 1058, 1023, 1005, 967, 942, 920, 908, 852, 836, 824, 787, 751.

HRMS (ESI) *m/z* Calculated for $C_{21}H_{24}N_2O_2F_3$ [M+H]$^+$, 393.1785; found: 393.1782.

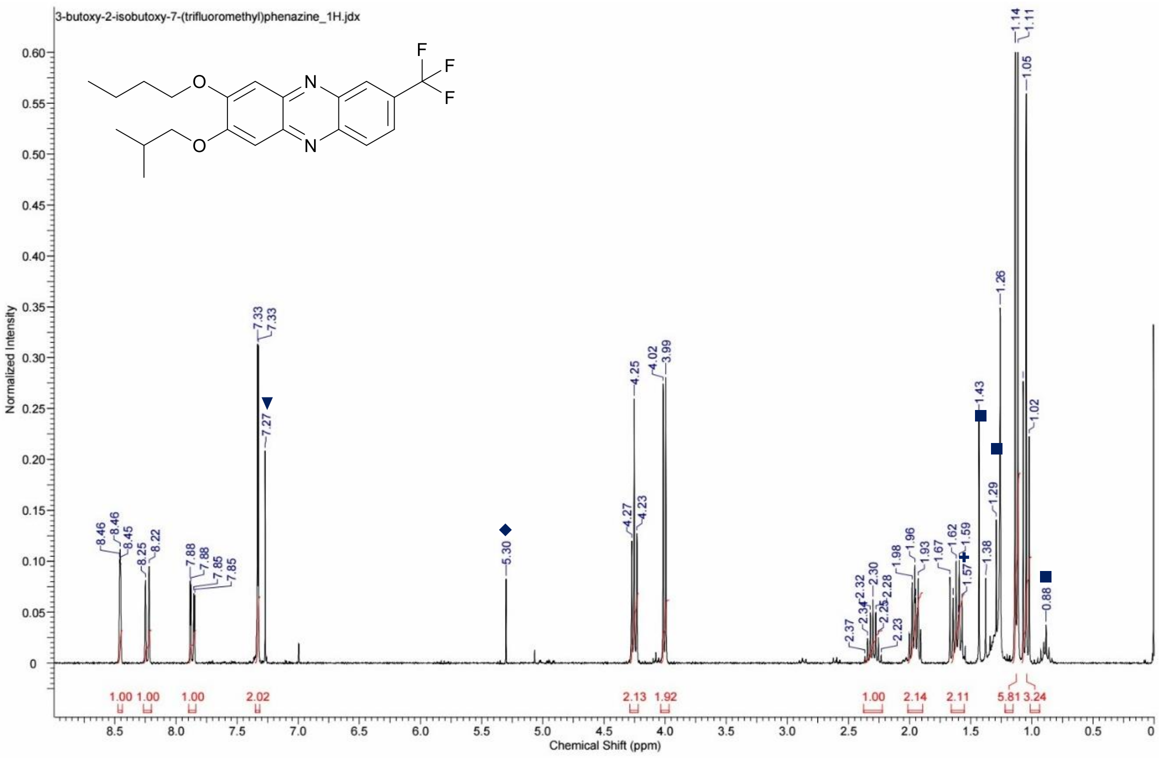

Figure S129. ^{1}H NMR ($CDCl_3$, 300 MHz, δ ppm) spectrum of **6d.**

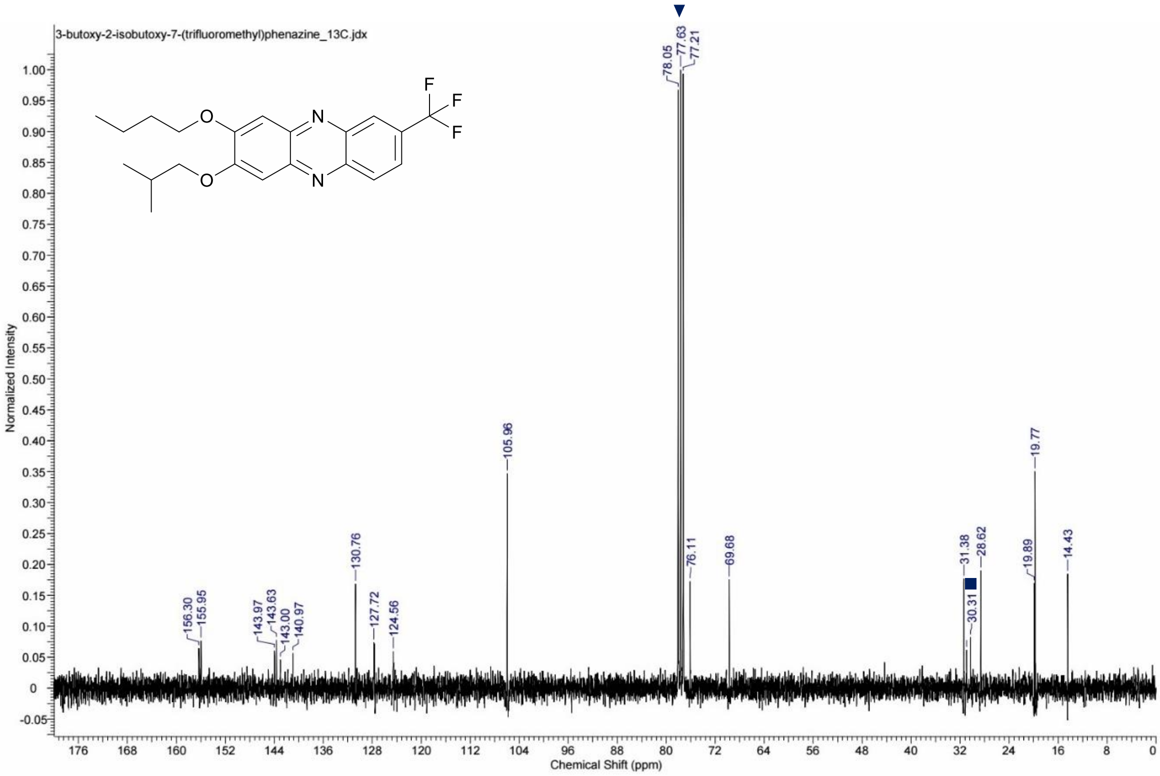

Figure S130. $^{13}C\{^{1}H\}$ NMR ($CDCl_3$, 75 MHz, δ ppm) NMR spectrum of **6d.**

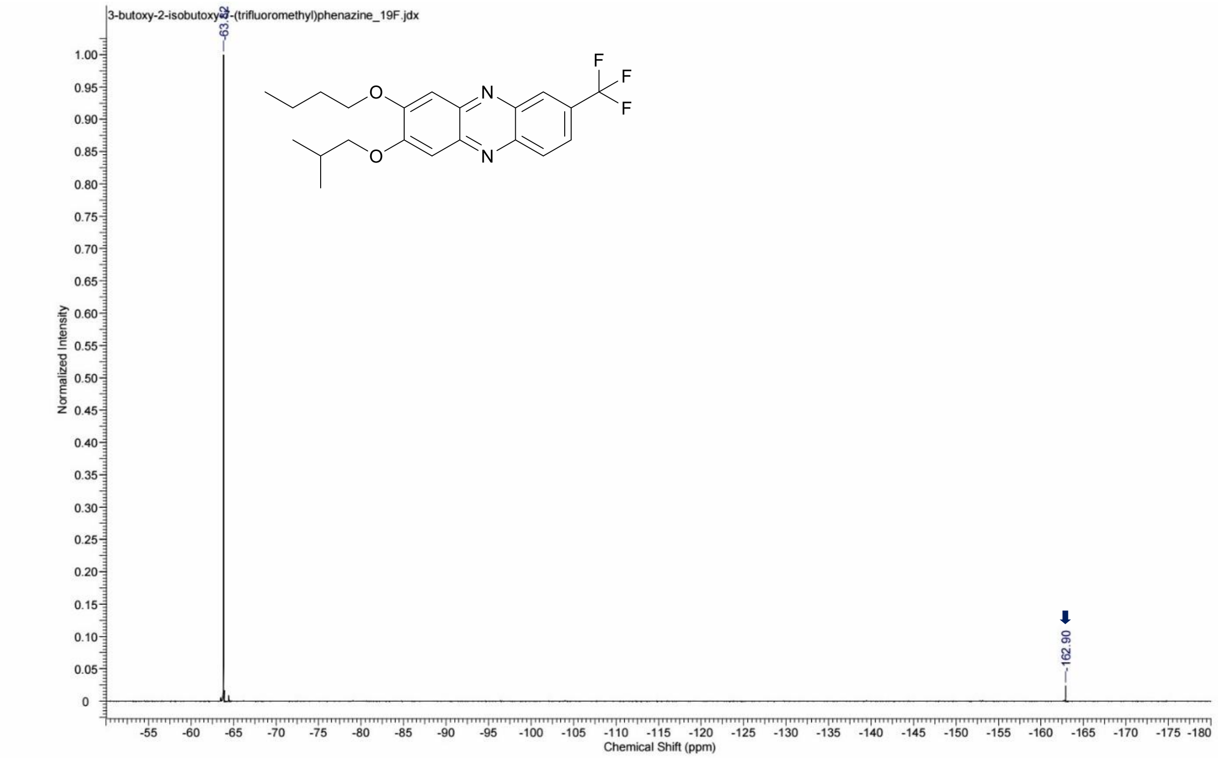

Figure S131. ^{19}F NMR ($CDCl_3$, 282 MHz, δ ppm) spectrum of **6d.**

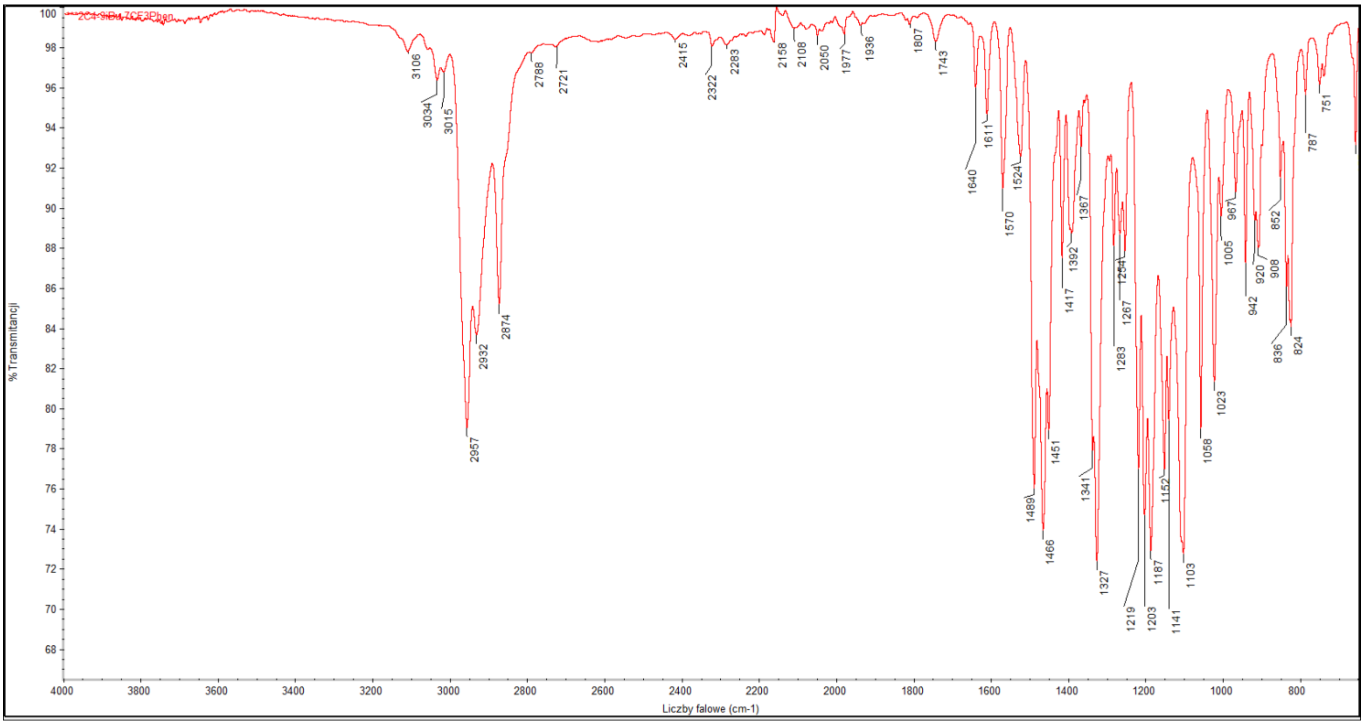

Figure S132. IR spectrum of **6d.**

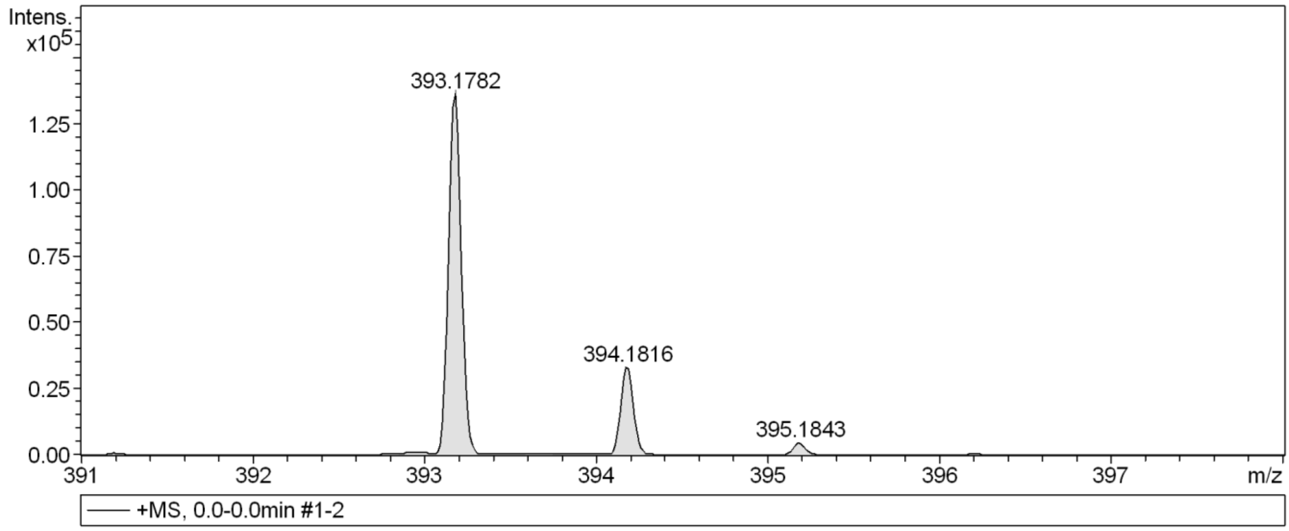

Figure S133. HRMS (ESI) spectrum of **6d**.

3-(hexyloxy)-2-isobutoxy-7-(trifluoromethyl)phenazine (6e).

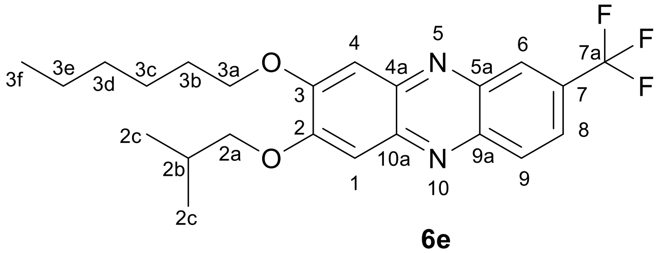

6e

^{1}H NMR ($CDCl_3$, 300 MHz, δ ppm): 8.46 (d, $^4J_{H6–H8}$ = 2.23 Hz, 1H, H_6), 8.24 (d, $^3J_{H8–H9}$ = 9.07 Hz, 1H, H_9), 7.87 (dd, $^3J_{H8–H9}$ = 9.07 Hz, $^4J_{H8–H6}$ = 2.23 Hz, 1H, H_8), 7.34 (s, 1H, H_1), 7.33 (s, 1H, H_4), 4.24 (t, $^3J_{H3a–H3b}$ = 6.36 Hz, 2H, H_{3a}), 4.01 (d, $^3J_{H2a–H2b}$ = 6.55 Hz, 2H, H_{2a}), 2.30 (m, 1H, H_{2b}), 2.02 – 1.91 (m, 2H, H_{3b}), 1.63 – 1.52 (m, 2H, H_{3c}), 1.45 – 1.36 (m, 4H, $H_{3d,3e}$), 1.13 (d, $^3J_{H2b–H2c}$ = 6.77 Hz, 6H, H_{2c}), 0.94 (t, $^3J_{H3e–H3f}$ = 7.09 Hz, 3H, H_{3f}).

^{13}C{^{1}H} NMR ($CDCl_3$, 75 MHz, δ ppm): 156.3 (C_2), 156.0 (C_3), 144.0 and 143.6 ($C_{4a,10a}$), 143.0 (C_7), 141.0 (C_{9a}), 130.7 (C_9), 127.7 (q, $^3J_{C–F}$ = 4 Hz, C_6), 124.5 (q, $^3J_{C–F}$ = 3 Hz, C_8), 106.1 (C_1), 105.6 (C_4), 76.1 (C_{2a}), 70.0 (C_{3a}), 32.1 (C_{3d}), 29.3 (C_{3b}), 28.6 (C_{2b}), 26.3 (C_{3c}), 23.2 (C_{3e}), 19.8 (C_{2c}), 14.6 (C_{3f}), signals from C_{7a} and C_{5a} are missing.

^{19}F NMR ($CDCl_3$, 282 MHz, δ ppm): –63.82 (s, 3F, F_{CF3}).

FT-IR (ATR, ν_{max}, (neat)/cm^{-1}): 3106, 3033, 3017, 2958, 2931, 2873, 2858, 1640, 1611, 1565, 1525, 1488, 1464, 1452, 1417, 1397, 1384, 1338, 1327, 1284, 1268, 1255, 1219, 1202, 1185, 1155, 1139, 1111, 1058, 1022, 997, 940, 909, 851, 825.

HRMS (ESI) *m/z* Calculated for $C_{23}H_{28}N_2O_2F_3$ [M+H]$^+$, 421.2098; found: 421.2096.

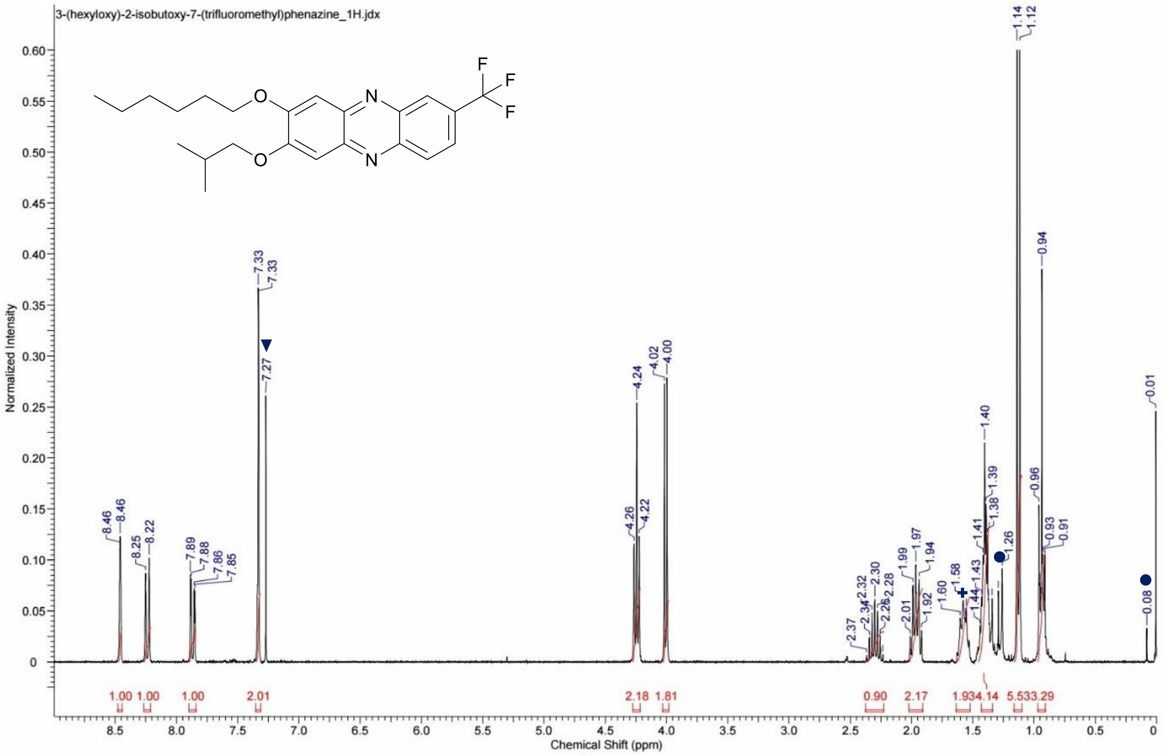

Figure S134. ^{1}H NMR ($CDCl_3$, 300 MHz, δ ppm) spectrum of **6e.**

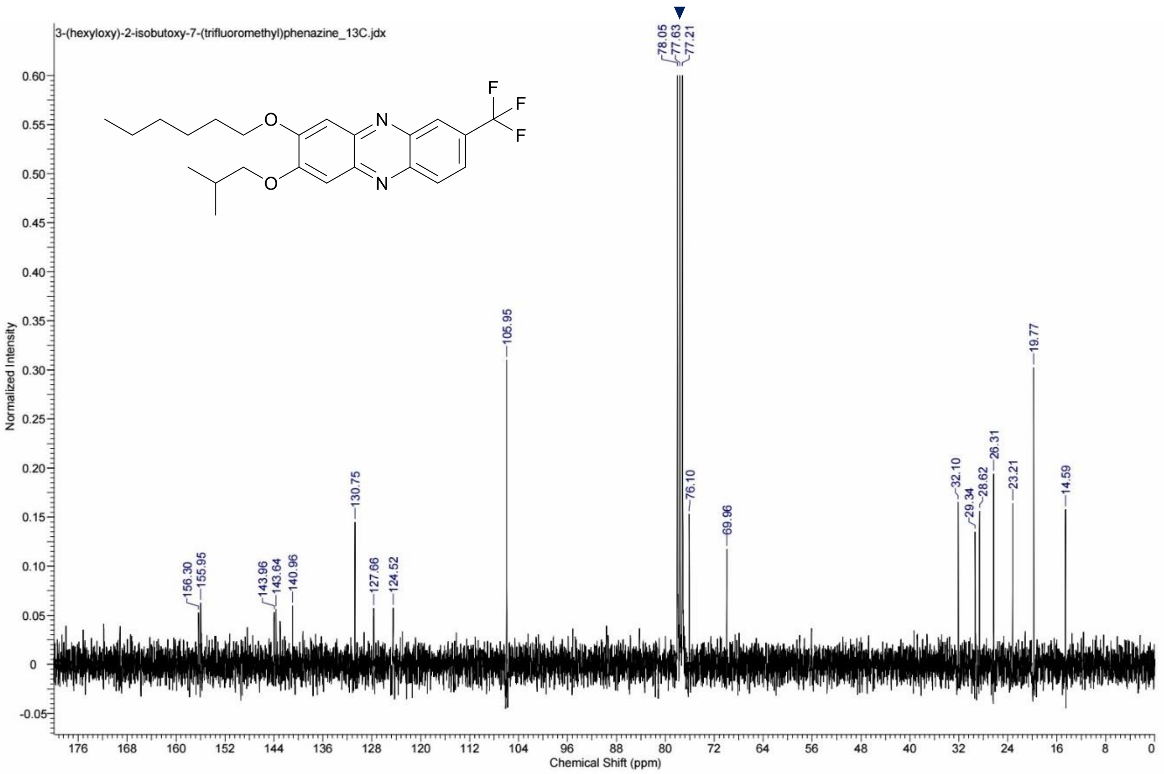

Figure S135. $^{13}C\{^{1}H\}$ NMR ($CDCl_3$, 75 MHz, δ ppm) NMR spectrum of **6e.**

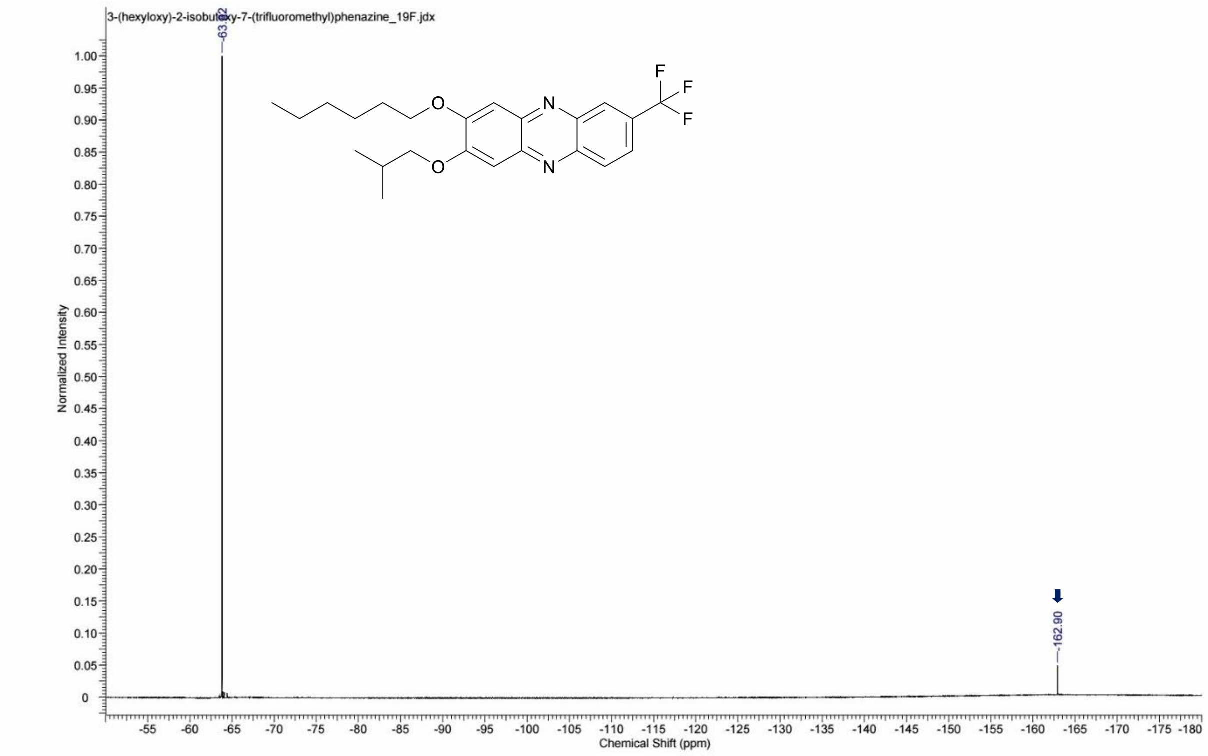

Figure S136. ^{19}F NMR ($CDCl_3$, 282 MHz, δ ppm) spectrum of **6e.**

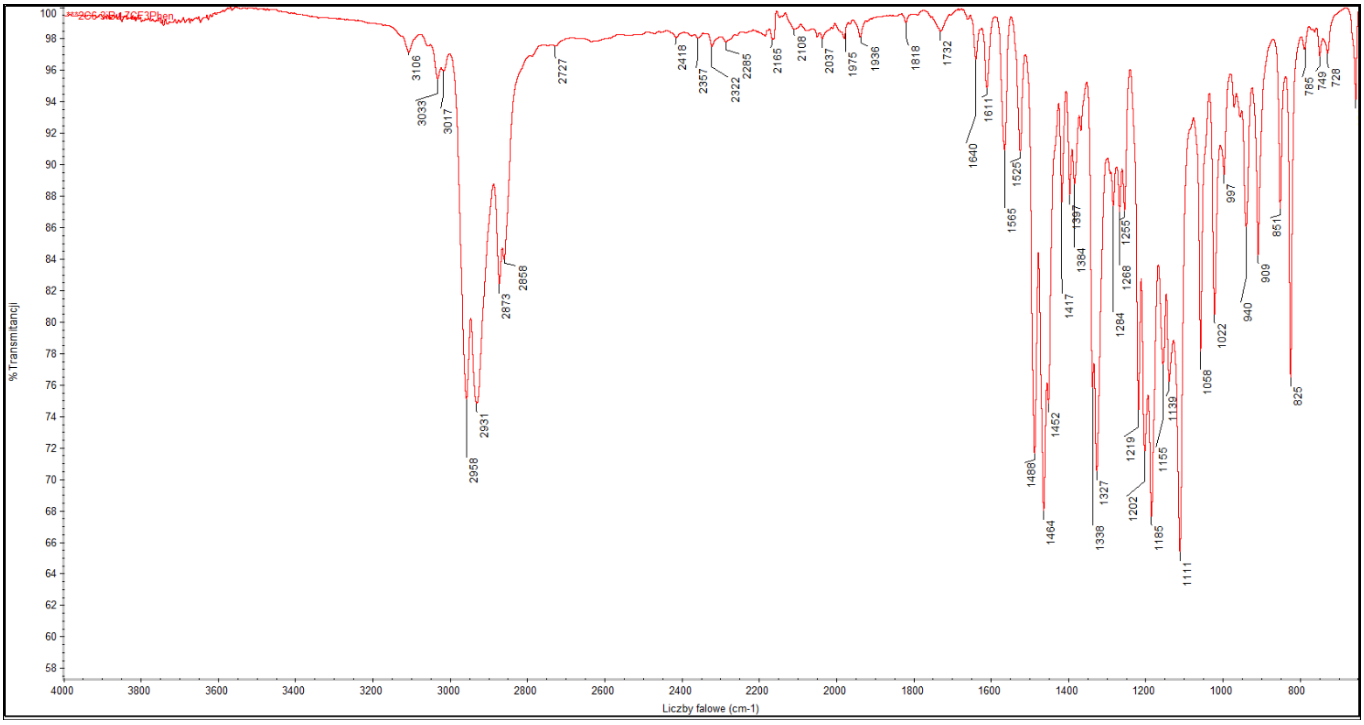

Figure S137. IR spectrum of **6e.**

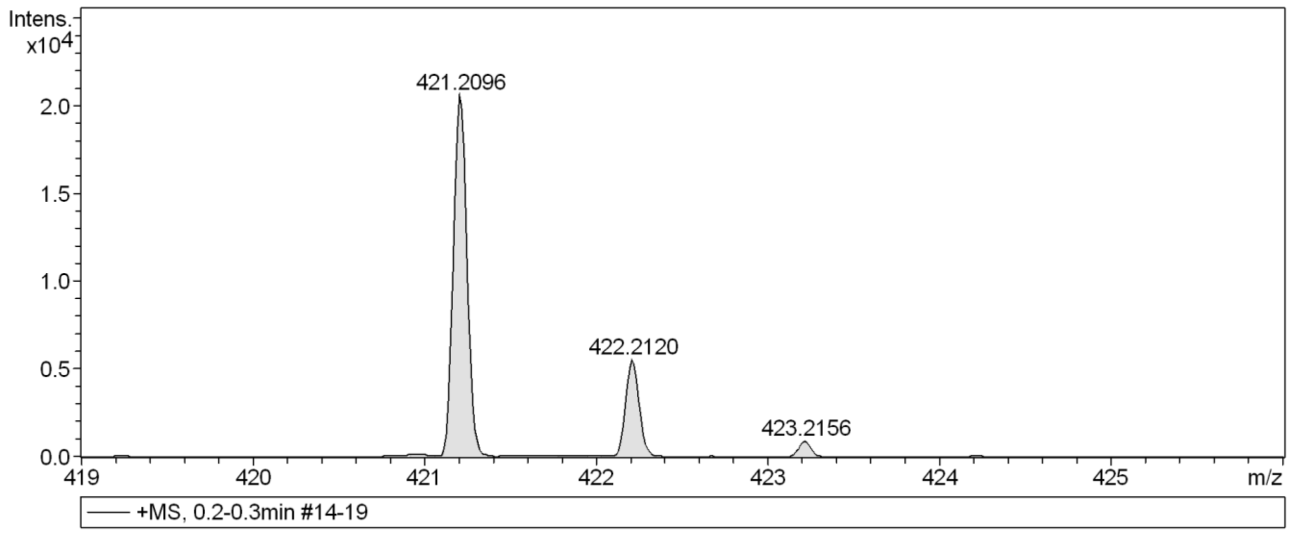

Figure S138. HRMS (ESI) spectrum of **6e**.

2-isobutoxy-3-(octyloxy)-7-(trifluoromethyl)phenazine (6f).

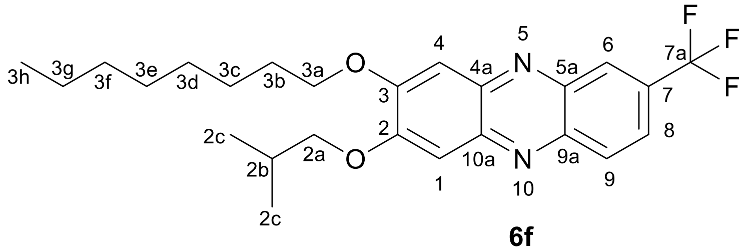

6f

^{1}H NMR ($CDCl_3$, 300 MHz, δ ppm): 8.46 (d, $^4J_{H6-H8}$ = 2.11 Hz, 1H, H_6), 8.24 (d, $^3J_{H8-H9}$ = 9.03 Hz, 1H, H_9), 7.87 (dd, $^3J_{H8-H9}$ = 9.03 Hz, $^4J_{H8-H6}$ = 2.11 Hz, 1H, H_8), 7.33 (2x s, 2x1H, $H_{1,4}$), 4.24 (t, $^3J_{H3a-H3b}$ = 6.45 Hz, 2H, H_{3a}), 4.01 (d, $^3J_{H2a-H2b}$ = 6.64 Hz, 2H, H_{2a}), 2.30 (m, 1H, H_{2b}), 2.06 – 1.92 (m, 2H, H_{3b}), 1.68 – 1.50 (m, 2H, H_{3c}), 1.49 – 1.21 (m, 8H, $H_{3d,3e,3f,3g}$), 1.13 (d, $^3J_{H2b-H2c}$ = 6.64 Hz, 6H, H_{2c}), 0.90 (t, $^3J_{H3g-H3h}$ = 7.40 Hz, 3H, H_{3h}).

^{13}C{1H} NMR ($CDCl_3$, 75 MHz, δ ppm): 156.3 (C_2), 156.0 (C_3), 144.0 and 143.6 ($C_{4a,10a}$), 143.0 (C_7), 141.0 (C_{9a}), 130.8 (C_9), 127.7 (q, $^3J_{C-F}$ = 4 Hz, C_6), 124.5 (q, $^3J_{C-F}$ = 3 Hz, C_8), 106.0 ($C_{1,4}$), 76.1 (C_{2a}), 70.0 (C_{3a}), 32.4 (C_{3f}), 30.9 (C_{3d}), 30.3 (C_{3b}), 29.9 (C_{3e}), 28.6 (C_{2b}), 26.4 (C_{3c}), 23.3 (C_{3g}), 19.8 (C_{2c}), 14.7 (C_{3h}), signals from C_{7a} and C_{5a} are missing.

^{19}F NMR ($CDCl_3$, 282 MHz, δ ppm): –63.82 (s, 3F, F_{CF3}).

FT-IR (ATR, ν_{max}, (neat)/cm^{-1}): 3108, 3033, 3015, 2959, 2927, 2873, 2857, 1640, 1611, 1565, 1524, 1488, 1465, 1524, 1488, 1465, 1452, 1417, 1397, 1304, 1367, 1338, 1327, 1285, 1268, 1256, 1220, 1204, 1185, 1155, 1140, 1112, 1059, 1022, 970, 943, 910, 852, 825, 792, 752, 725.

HRMS (ESI) *m/z* Calculated for $C_{25}H_{32}N_2O_2F_3$ $[M+H]^+$, 449.2411; found: 449.2408.

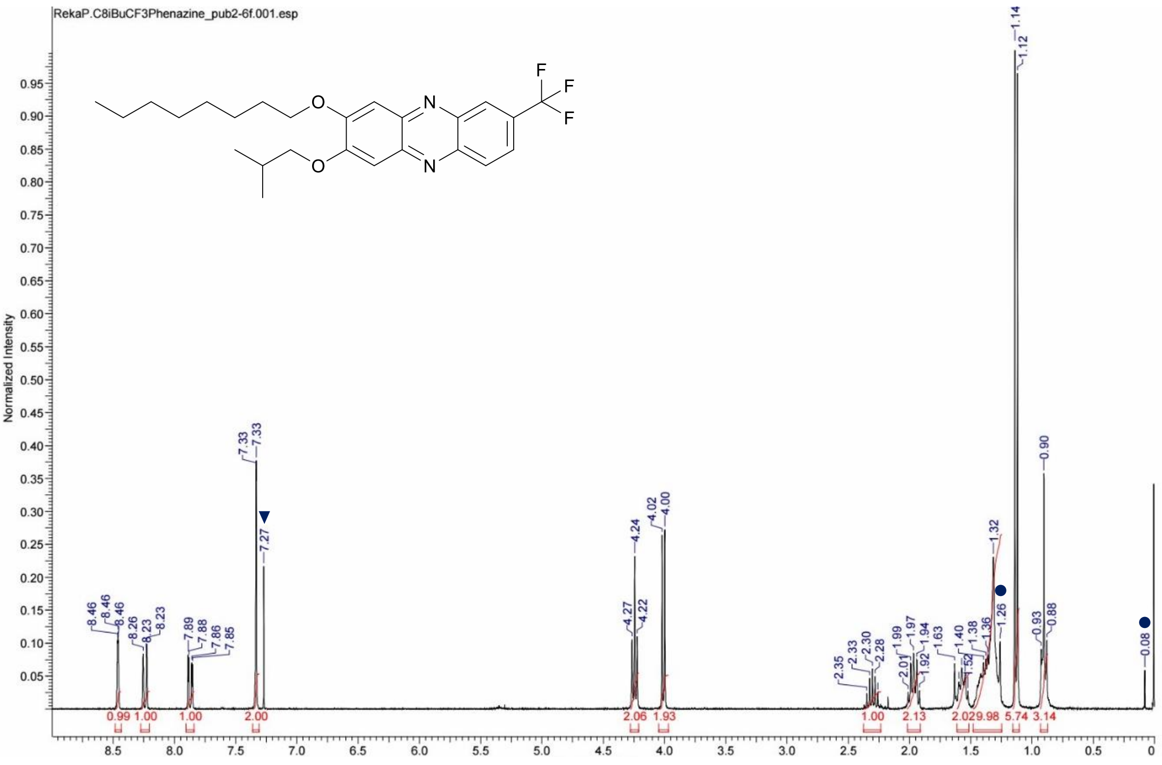

Figure S139. ^{1}H NMR ($CDCl_3$, 300 MHz, δ ppm) spectrum of **6f.**

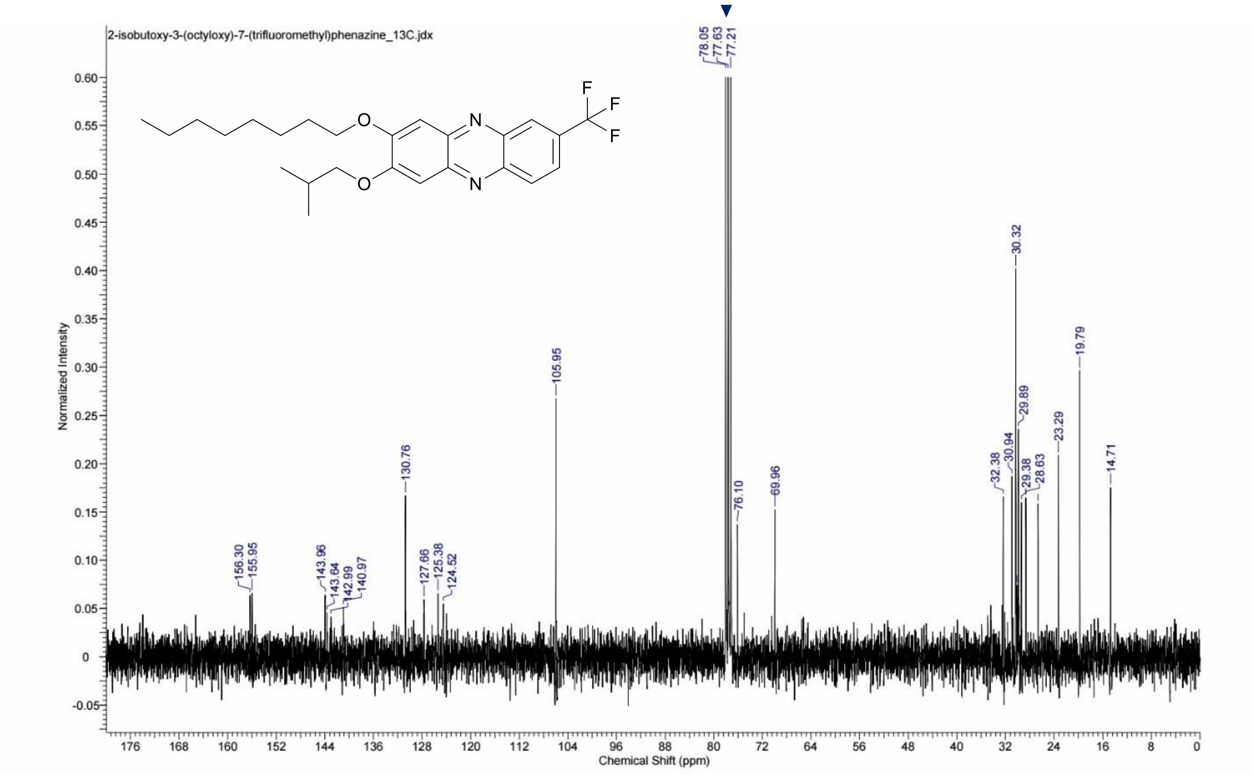

Figure S140. $^{13}C\{^{1}H\}$ NMR ($CDCl_3$, 75 MHz, δ ppm) NMR spectrum of **6f.**

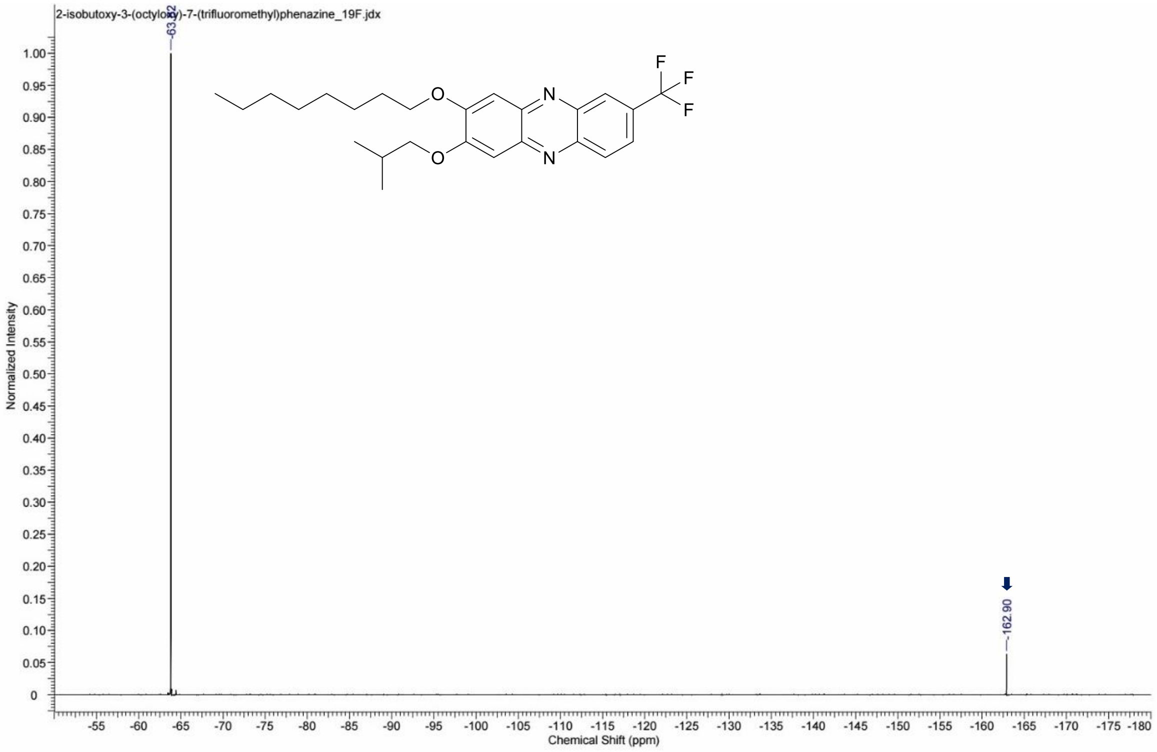

Figure S141. ^{19}F NMR ($CDCl_3$, 282 MHz, δ ppm) spectrum of **6f.**

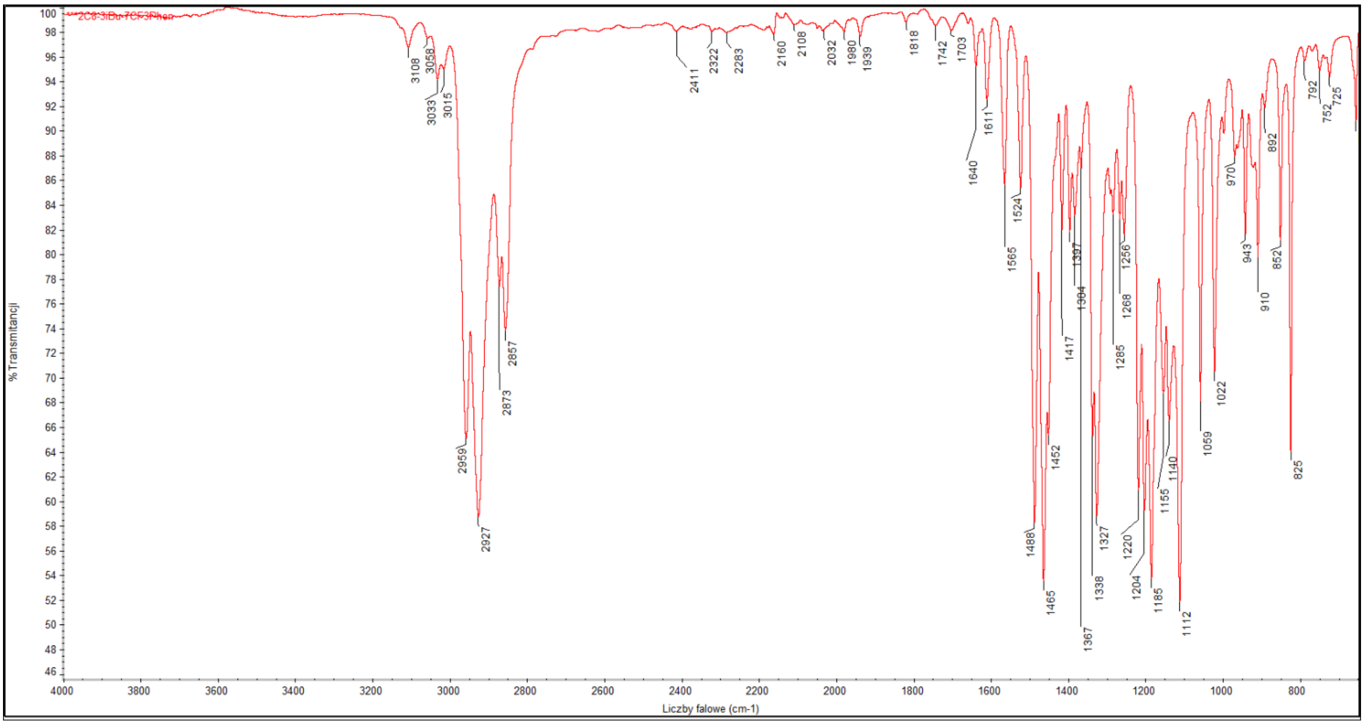

Figure S142. IR spectrum of **6f.**

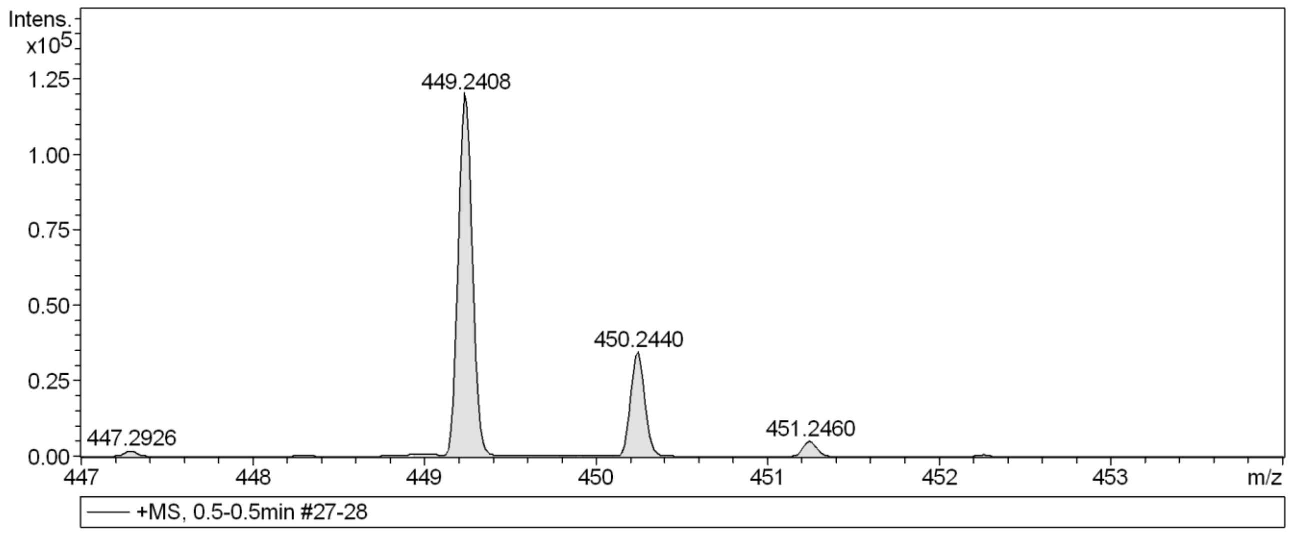

Figure S143. HRMS (ESI) spectrum of **6f**.

3-(decyloxy)-2-isobutoxy-7-(trifluoromethyl)phenazine (6g).

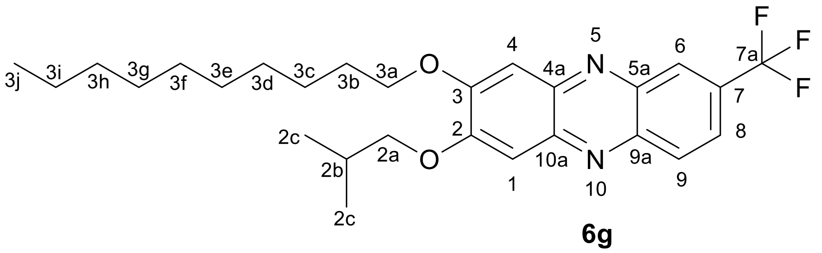

6g

^{1}H NMR ($CDCl_3$, 300 MHz, δ ppm): 8.46 (d, $^{4}J_{H6-H8}$ = 2.03 Hz, 1H, H_6), 8.24 (d, $^{3}J_{H8-H9}$ = 8.96 Hz, 1H, H_9), 7.87 (dd, $^{3}J_{H8-H9}$ = 8.96 Hz, $^{3}J_{H8-H6}$ = 2.03 Hz, 1H, H_8), 7.34 (s, 1H, H_1), 7.33 (s, 1H, H_4), 4.25 (t, $^{3}J_{H3a-H3b}$ = 6.56 Hz, 2H, H_{3a}), 4.01 (d, $^{3}J_{H2a-H2b}$ = 6.56 Hz, 2H, H_{2a}), 2.30 (m, 1H, H_{2b}), 2.08 – 1.91 (m, 2H, H_{3b}), 1.65 – 1.50 (m, 2H, H_{3c}), 1.48 – 1.24 (m, 12H, $H_{3d,3e,3f,3g,3h,3i}$), 1.13 (d, $^{3}J_{H2b-H2c}$ = 6.74 Hz, 6H, H_{2c}), 0.89 (t, $^{3}J_{H3i-H3j}$ = 6.42 Hz, 3H, H_{3j}).

$^{13}C\{^{1}H\}$ NMR ($CDCl_3$, 75 MHz, δ ppm): 156.3 (C_2), 156.0 (C_3), 144.0 and 143.7 ($C_{4a,10a}$), 143.0 (C_7), 141.0 (C_{9a}), 130.7 (C_9), 127.7 (q, $^{3}J_{C-F}$ = 4 Hz, C_6), 124.6 (q, $^{3}J_{C-F}$ = 3 Hz, C_8), 106.0 ($C_{1,4}$), 76.1 (C_{2a}), 70.0 (C_{3a}), 32.5 (C_{3h}), 30.4 – 30.0 ($C_{3b,3d,3e,3f,3g}$), 28.6 (C_{2b}), 26.6 (C_{3c}), 23.3 (C_{3i}), 19.8 (C_{2c}), 14.7 (C_{3j}), signals from C_{7a} and C_{5a} are missing.

^{19}F NMR ($CDCl_3$, 282 MHz, δ ppm): –63.82 (s, 3F, F_{CF3}).

FT-IR (ATR, ν_{max}, (neat)/cm^{-1}): 3106, 3056, 3033, 3013, 2957, 2924, 2871, 2853, 1640, 1611, 1565, 1524, 1488, 1464, 1448, 1417, 1397, 1384, 1365, 1338, 1327, 1284, 1268, 1256, 1219, 1204, 1186, 1155, 1139, 1112, 1058, 1022, 942, 910, 852, 825, 787, 748, 722.

HRMS (ESI) *m/z* Calculated for $C_{27}H_{36}N_2O_2F_3$ $[M+H]^+$, 477.2724; found: 477.2720.

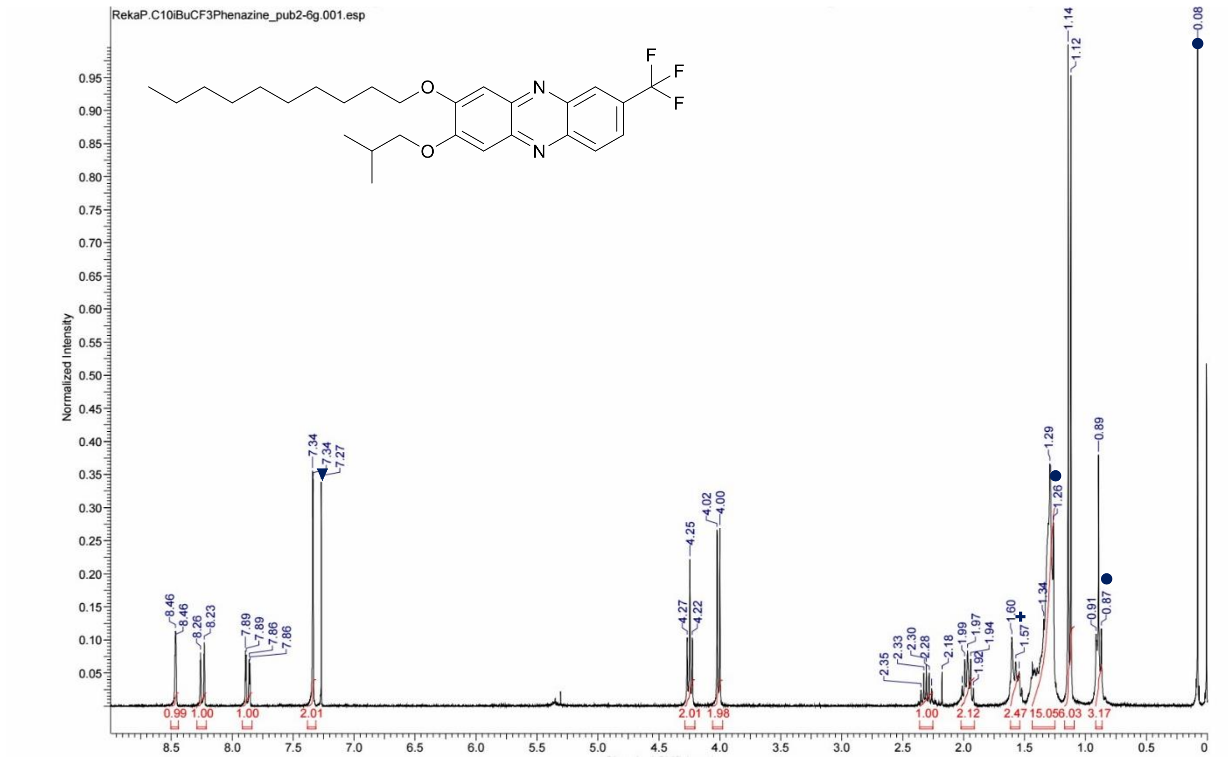

Figure S144. ^{1}H NMR ($CDCl_3$, 300 MHz, δ ppm) spectrum of **6g.**

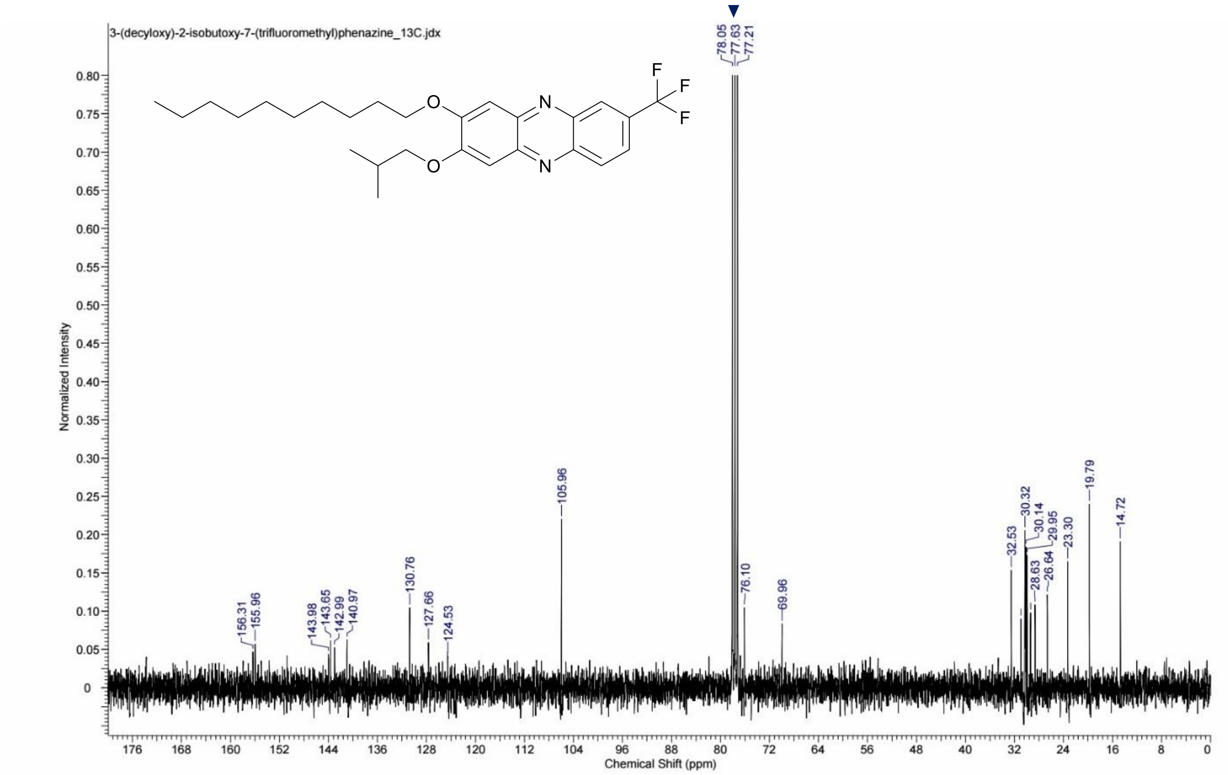

Figure S145. $^{13}C\{^{1}H\}$ NMR ($CDCl_3$, 75 MHz, δ ppm) NMR spectrum of **6g.**

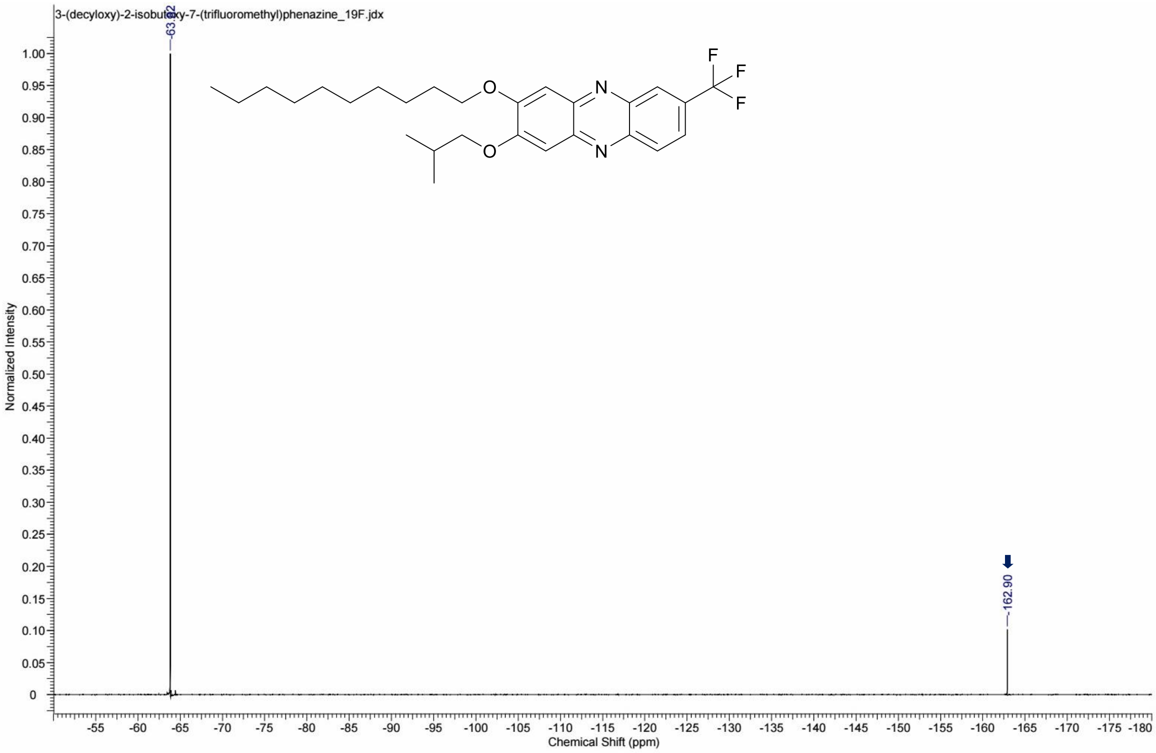

Figure S146. ^{19}F ($CDCl_3$, 282 MHz, δ ppm) NMR spectrum of **6g.**

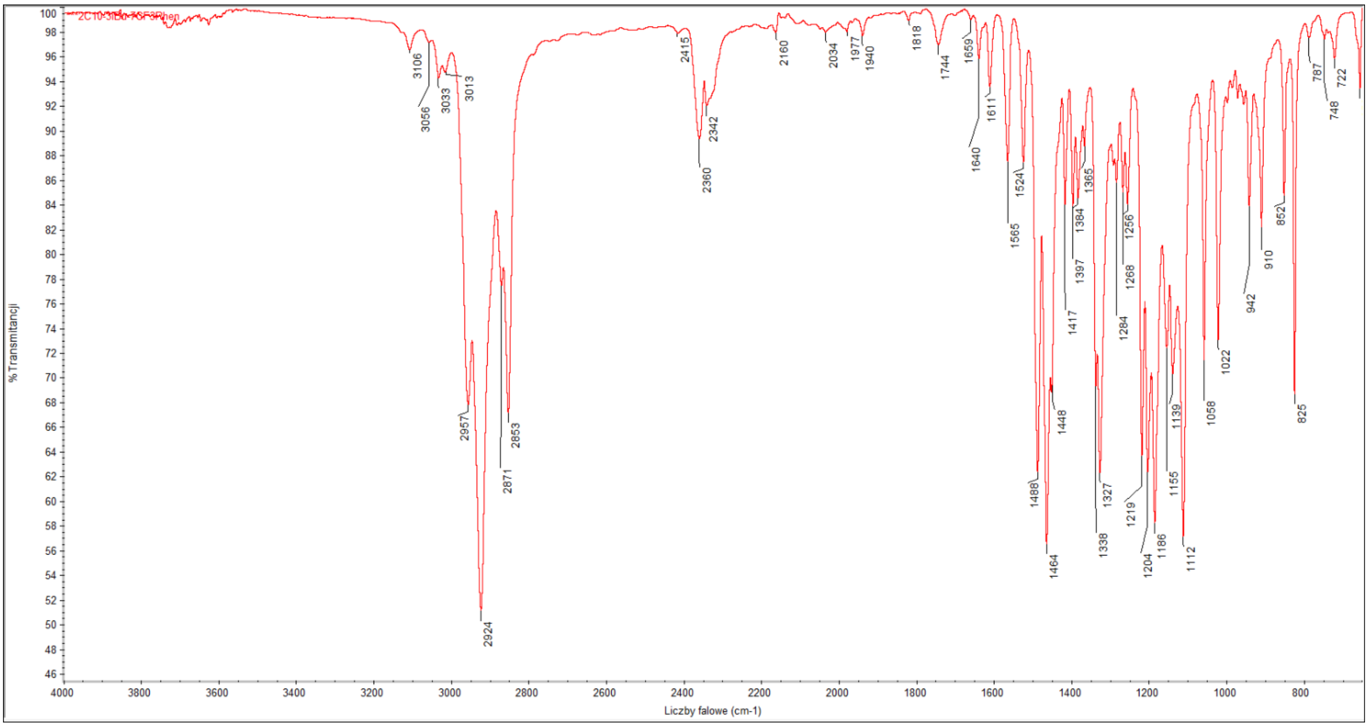

Figure S147. IR spectrum of **6g.**

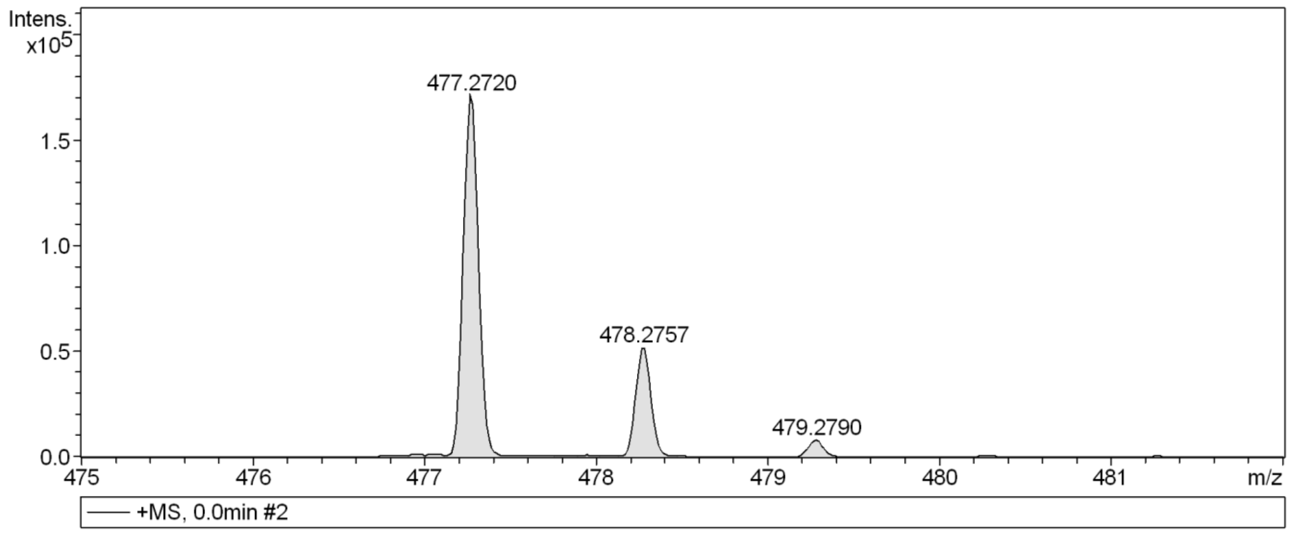

Figure S148. HRMS (ESI) spectrum of **6g**.

2,3-bis(hexyloxy)-7,8-dimethoxyphenazine (7).

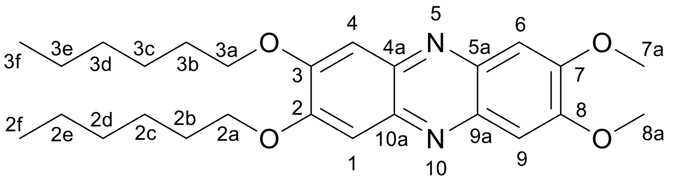

7

^{1}H NMR ($CDCl_3$, 300 MHz, δ ppm): 7.35 (s, 2H, $H_{6,9}$), 7.31 (s, 2H, $H_{1,4}$), 4.21 (t, $^{3}J_{H2a–H2b,H3a–H3b}$ = 6.61 Hz, 4H, $H_{2a,3a}$), 4.08 (s, 6H, $H_{7a,8a}$), 2.00 – 1.89 (m, 4H, $H_{2b,3b}$), 1.61 – 1.48 (m, 4H, $H_{2c,3c}$), 1.43 – 1.34 (m, 8H, $H_{2d,2e,3d,3e}$), 0.93 (t, $^{3}J_{H2e–H2f,H3e–H3f}$ = 7.10 Hz, 6H, $H_{2f,3f}$).

^{13}C{^{1}H} NMR ($CDCl_3$, 75 MHz, δ ppm): 153.8 ($C_{2,3}$), 153.6 ($C_{7,8}$), 140.5 ($C_{4a,10a}$), 140.2 ($C_{5a,9a}$), 106.4 ($C_{6,9}$), 106.0 ($C_{1,4}$), 69.8 ($C_{2a,3a}$), 56.9 ($C_{7a,8a}$), 32.2 ($C_{2d,3d}$), 29.4 ($C_{2b,3b}$), 26.4 ($C_{2c,3c}$), 23.2 ($C_{2e,3e}$), 14.6 ($C_{2f,3f}$).

FT-IR (ATR, ν_{max}, (neat)/cm^{-1}): 3093, 3076, 3011, 3002, 2950, 2926, 2864, 2855, 2830, 1738, 1671, 1635, 1593, 1528, 1485, 1464, 1436, 1422, 1387, 1363, 1287, 1267, 1237, 1206, 1186, 1154, 1074, 1043, 1032, 1009, 992, 951, 928, 916, 842, 831, 765, 737, 723.

HRMS (ESI) *m/z* Calculated for $C_{19}H_{20}N_2O_2F_3$ $[M+H]^+$, 441.2748; found: 441.2748.

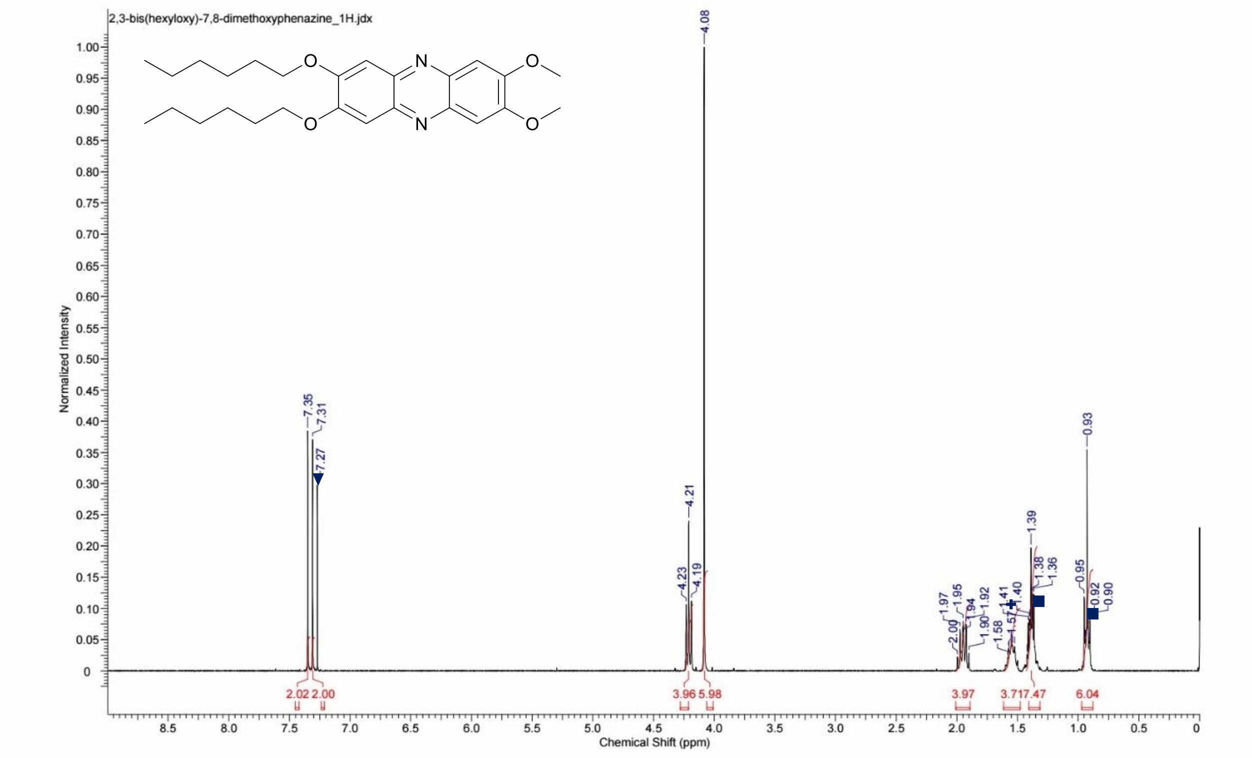

Figure S149. ^{1}H NMR ($CDCl_3$, 300 MHz, δ ppm) spectrum of **7.**

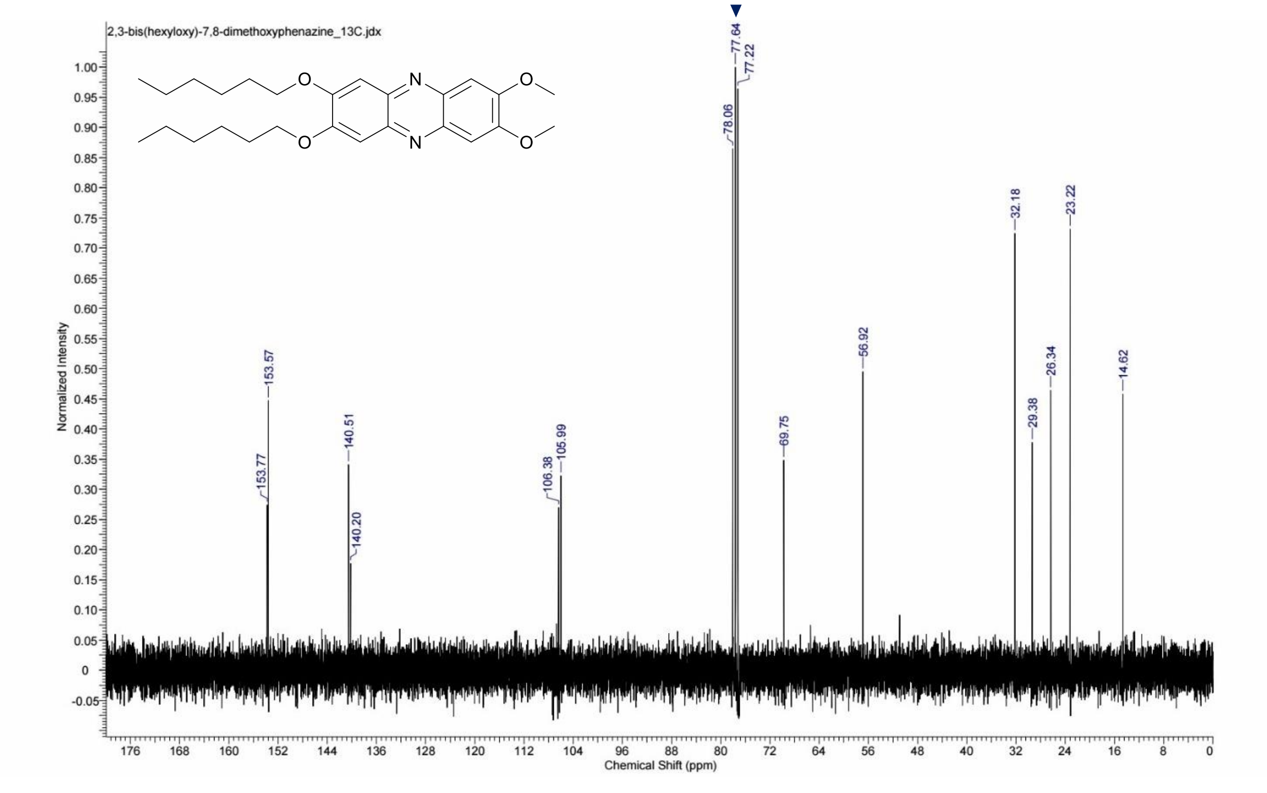

Figure S150. $^{13}C\{^{1}H\}$ NMR ($CDCl_3$, 75 MHz, δ ppm) NMR spectrum of **7.**

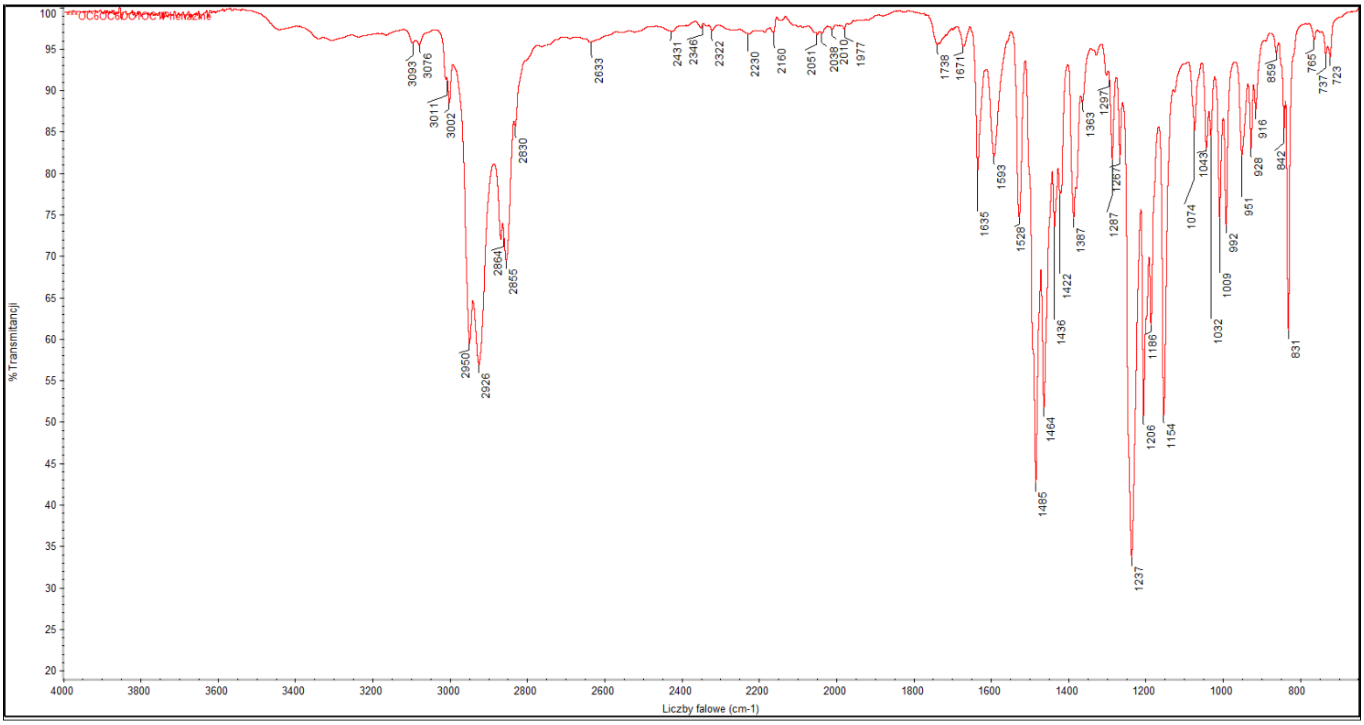

Figure S151. IR spectrum of **7.**

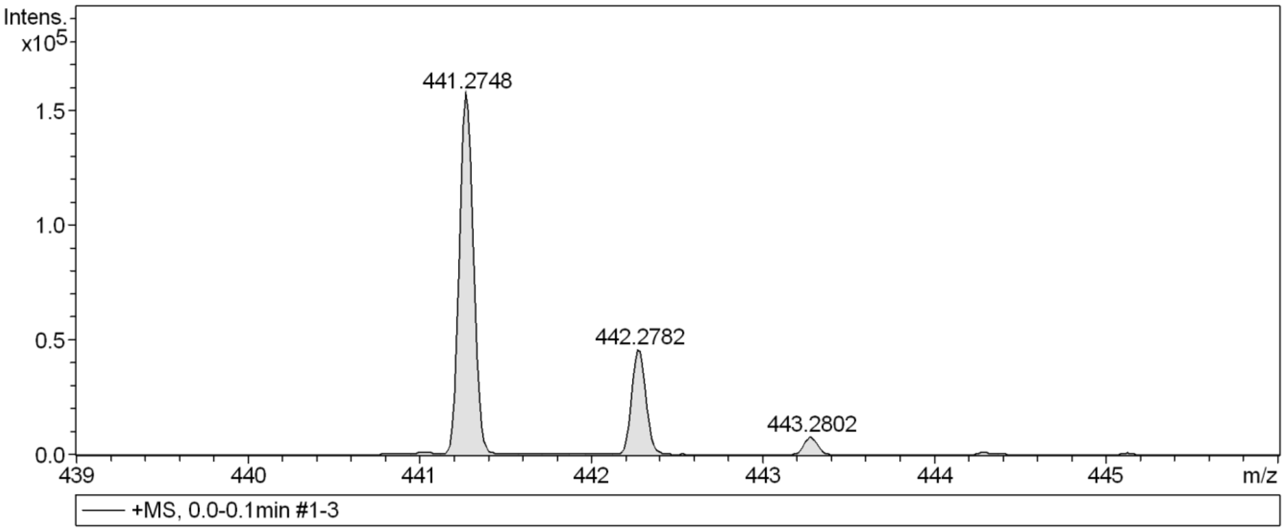

Figure S152. HRMS (ESI) spectrum of **7**.

Crystallographic data.

Table S1. Crystal data, intensity measurement conditions and structure refinement details for **5c hydrate** and **6b** at T=100 K and for **6b solvate** at T=120 K

**Identification code**	**5c hydrate**	**6b**	**6b solvate**
**Crystal data**			
Chemical formula sum	$C_{22}H_{33.15}N_2O_{4.57}$	$C_{228}H_{228}F_{36}N_{24}O_{24}$	$C_{21}H_{23}Br_2F_3N_2O_2$
Chemical formula	$C_{22}H_{28}N_2O_2$	$C_{19}H_{19}F_3N_2O_2$	$C_{19}H_{19}F_3N_2O_2$
Solvents	2.575 H_2O	none	$C_2H_4Br_2$
Mr	398.85	364.36	552.23
Wavelength (Å)	1.54184	1.54184	0.71073
Temperature (K)	100.0 (1)	100.0 (1)	119.9 (2)
Crystal system	Monoclinic	Triclinic	Triclinic
Space group	*P* 2_1/c	*P* -1	*P* -1
Unit cell dimensions (Å, °)	*a* = 14.7376 (1)	*a* = 13.1641 (3)	*a* = 9.2360 (2)
	b = 22.5390 (2)	*b* = 23.2671 (4)	*b* = 11.0079 (2)
	c = 6.90584 (4)	*c* = 35.5423 (9)	*c* = 12.8655 (3)
	α = 90.000	*α* = 94.735 (2)	*α* = 90.075 (2)
	β = 96.7553 (6)	*β* = 95.369 (2)	*β* = 108.878 (2)
	γ = 90.000	*γ* = 105.086 (2)	*γ* = 114.208 (2)
V (Å^3)	2277.99 (3)	10400.1 (4)	1115.14(5)
Z, Z', *Dx* (Mg/m^3)	4, 1, 1.163	24, 12, 1.396	2, 1, 1.645
μ (mm^{-1})	0.656	0.962	3.679
F (000)	863	4560	552
Crystal size (mm)	0.60x06x0.02	0.40x0.10x0.01	0.55x0.35x0.05
Data collection			
ϑ Range (º)	3.019 – 70.992	2.459 – 68.999	2.539 – 27.996
Method	*ω* scans	*ω* scans	*ω* scans
hkl ranges	*h*: -18, 18;	*h*: -16, 15;	*h*: -12, 12;
	k: -27, 27;	*k*: -26, 28;	k: -14, 14;
	l: -6, 8;	*l*: -42, 43;	*l*: -16, 16;
Reflections collected	44794	133909	56601
Reflections unique	4393	38454	5388
R(int)	0.0503	0.0737	0.0651
Reflections $I > 2\sigma I$)	3994	14583	4586
Completeness (%, θ max)	100.0	99.9	99.9
T_{min}, T_{max}	0.694, 0.987	0.700, 0.990	0.237, 0.837
**Refinement**			
Data/restr./param.	4394 / 9 / 280	38454 / 13 / 1281	5388 / 0 / 271
Goodness-of-fit	1.031	1.373	1.049
R1 [$I > 2\sigma(I)$]	0.0438	0.1446	0.0324
wR2 (all data)	0.1284	0.4783	0.0836
Weighting scheme: *A*, *B*	0.0763, 0.7801	0.2000, 0.0000	0.0358, 11.3277
$\Delta\rho_{max}$ $\Delta\rho_{min}$ rms (e Å^{-3})	0.578 -0.218 0.051	1.368 -0.880 0.139	0.612 0.450 0.086

X-ray diffraction data for the single crystals of **5c hydrate** and **6b** were collected using XtalLAB Synergy-S (Rigaku - Oxford Diffraction, 2020) four circle diffractometer with a mirror monochromator and a micro-focus MoKα or CuKα radiation sources. The diffractometer was equipped with the CryoStream cryostat system. The data were collected at about 100 K using Cu Kα radiation. The obtained data sets were processed with CrysAlisPro v171.41.93a software (Rigaku-Oxford Diffraction 2021) andCrysAlisPro 1.171.40.84a (Rigaku Oxford Diffraction, 2020), respectively. In the case of **6b solvate** X-ray diffraction data for the single crystal were collected using SuperNova^{TM} four circle diffractometer with a mirror monochromator and a micro-focus sealed X-ray tubes as MoKα or CuKα radiation sources. The diffractometer was equipped with the CryoJet cryostat system. The data were collected at about 120 K using Mo Kα radiation. The obtained data set was processed with CrysAlisPro v 1.171.42.53a (Rigaku Oxford Diffraction, 2022).

The phase problem was solved by direct methods with SIR92 [Altomare A., Cascarano G., Giacovazzo C., Gualardi A. (1993) J. Appl. Crystallogr. 26, 343-350] for **5c hydrate** and with SHELXT 2014/5 (Sheldrick, 2014) for **6b** and **6b solvate** [Sheldrick G.M. (2015a). Acta Crystallogr. A71, 3-8], Parameters of the obtained models were refined using SHELXL-2013/4 (Sheldrick 2015b). SHELXL - CRYSTAL STRUCTURE REFINEMENT - MULTI-CPU VERSION [Copyright(C) George M. Sheldrick 1993-2013, Version 2013/4, [Sheldrick G.M., Acta Crystallogr. C71 (2015) 3-8]; $w = 1/[\sigma^2(F_o^2) + AP^2 + BP]$ where $P = (F_o^2+2F_c^2)/3$. The figures presenting the structural features were prepared with ORTEP-3 for Windows version 2020.1 software and all calculations were performed using WinGX integrated system – version 2001,1 [Farrugia L.J. (2012) J. Appl. Crystallogr. 45, 849-854].

In the case of pure 6b structure, i.e., that one without a cocrystallizing solvent, the structure refinement was performed only with the isotropic displacement parameters for non-hydrogen atoms due to very low-quality crystals and too many symmetrically independent molecules (12), what caused high correlations between their anisotropic displacement parameters for non-hydrogen atoms (6 displacement parameters per each non-hydrogen atom gives 1872-312 = 1560 additional parameters to be refined).

Table S2. Selected bond lengths (Å), valence angle and torsion angles (°) for **5c hydrate** and pure **6b** at T=100 K and for **6b solvate** at T=120 K. For **6b** only the essential mean values for the 12 symmetrically independent molecules A-L are given with the range of the appropriate values in parentheses.

Identification code	5c hydrate	6b		6b solvate
**Bond lengths**				
C1-C2	1.365 (2)	1.361(3)	[1.295-1.429]	1.364 (3)
C2-C3	1.454 (2)	1.462 (3)	[1.375-1.493]	1.458 (3)
C3-C4	1.366 (2)	1.364 (3)	[1.329-1.414]	1.363 (3)
C4-C4A	1.423 (2)	1.421 (3)	[1.390-1.455]	1.424 (3)
C4A-C10A	1.444 (2)	1.438 (3)	[1.401-1.489]	1.448 (3)
C4A-N5	1.339 (2)	1.335 (3)	[1.302-1.404]	1.339 (3)
N5-C5A	1.351 (2)	1.345 (4)	[1.292-1.378]	1.353 (3)
C5A-C9A	1.427 (2)	1.418 (4)	[1.368-1.460]	1.428 93)
C5A-C6	1.426 (2)	1.432 (3)	[1.410-1.451]	1.425 (3)
C6-C7	1.370 (2)	1.372 (3)	[1.313-1.406]	1.363 (3)
C7-C8	1.436 (2)	1.422 (3)	[1.376-1.464]	1.422 (3)
C8-C9	1.364 (2)	1.362 (3)	[1.339-1.400]	1.364 (3)
C9-C9A	1.424 (2)	1.434 (4)	[1.416-1.479]	1.426 (3)
C9A-N10	1.352 (2)	1.360 (4)	[1.326-1.398]	1.350 (3)
N10-C10A	1.339 (2)	1.342 (4)	[1.308-1.370]	1.335 (3)
C10A-C1	1.428 (2)	1.428 (3)	[1.368-1.460]	1.426 (3)
C2-O2	1.352 (1)	1.349 (3)	[1.327-1.370]	1.350 (3)
O2-C21	1.448 (2)	1.450 (3)	[1.415-1.460]	1.440 (3)
C21-C22	1.518 (2)	1.513 (3)	[1.494-1.526]	1.517 (3)
C22-C23	1.521(2)	1.533 (3)	[1.499-1.559]	1.526 (3)
C22-C24	1.526 (2)	1.520 (3)	[1.474-1.556]	1.524 (4)
C3-O3	1.354 (1)	1.354 (3)	[1.334-1.394]	1.349 (3)
O3-C31	1.445 (2)	1.436 (4)	[1.400-1.475]	1.444 (3)
C31-C32	1.504 (2)	1.500 (3)	[1.473-1.524]	1.511 (3)
C7-C70	1.533 (2)	1.500 (3)	[1.466-1.543]	1.499 (3)

C70-C71 / F71	1.537 (2)	1.347 (3)	[1.313-1.375]	1.336 (3)
C70-C72 / F72	1.536 (2)	1.329 (3)	[1.308-1.368]	1.354 (3)
C70-C73 / F73	1.528 (2)	1.352 (3)	[1.322-1.411]	1.333 (3)
Br1-C11				1.969 (2)
C11-C12				1.508 (3)
C12-Br2				1.960 (3)
**Valence angles**				
C1-C2-C3	120.7 (1)			121.0 (2)
C2-C3-C4	120.4 (1)			120.5 (2)
C3-C4-C4A	120.1 (1)			119.7 (2)
C4-C4A-C10A	119.6 (1)			119.8 (2)
C4-C4A-N5	118.9 (1)			118.8 (2)
C4A-N5-C5A	117.2 (1)	116.6 (2)		116.6 (2)
N5-C5A-C9A	121.1 (1)			121.7 (2)
N5-C5A-C6	119.1 (1)			118.7 (2)
C5A-C6-C7	121.2 (1)			119.5 (2)
C6-C7-C8	118.1 (1)			121.3 (2)
C6-C7-C70	122.5 (1)			120.5 (2)
C70-C7-C8	119.4 (1)			118.1 (2)
C7-C8-C9	122.4 (1)			120.6 (2)
C8-C9-C9A	120.1 (1)			120.1 (2)
C9-C9A-C5A	118.3 (1)			119.1 (2)
C5A-C9A-N10	121.9 (1)			121.8 (2)
C9-C9A-N10	119.9 (1)			119.1 (2)
C9A-N10-C10A	117.0 (1)	116.0 (2)		116.5 (2)
C10A-C1-C2	120.0 (1)			119.7 (2)
C1-C2-O2	125.6 (1)			126.2 (2)
C2-O2-C21	117.6 (1)	116.8 (2)		118.2 (2)
O2-C21-C22	107.1 (1)	107.5 (2)		107.3 (2)
C21-C22-C23	111.7 (1)	110.1 (2)		108.6 (2)
C21-C22-C24	111.3 (1)	111.3 (2)		112.3 (2)

C23-C22-C24	110.3 (1)	112.6 (2)	110.9 (2)
C2-C3-O3	114.3 (1)		112.7 (2)
C3-O3-O31	115.9 (1)	116.6 (2)	118.2 (2)
O3-C31-C32	107.8 (1)	107.0 (2)	105.4 (2)
C7-C70-C71/F71	108.5 (1)		112.6 (2)
C7-C70-C72/F72	109.7 (1)		111.1 (2)
C7-C70-C73/F73	112.2 (1)		113.5 (2)
C71/F71-C70-C72/F72	109.5 (1)		105.2 (2)
C71/F71-C70-C73/F72	109.0 (1)		107.5 (20
C72/F72-C70-C73/F73	107.9 (1)		106.3 (2)
Br1-C11-C12			107.6 (2)
C11-C12-Br2			107.7 (2)
**Torsion angles**			
C1-C2-O2-C21	3.6 (2)		-0.4 (3)
C3-C2-O2-C21	-177.0 (1)	165.3 (2)*	179.3 (2)
		179.5 (2)**	
C2-O2-C21-C22	179.3 (1)	-173.0 (2)*	171.4 (2)
		174.5 (2)**	
O2-C21-C22-C23	66.5 (1)	-69.7 (2)*	179.5 (2)
		168.4 (2)**	
O2-C21-C22-C24	-57.3 (1)	-69.7 (2)*	-56.4 (3)
		168.4 (2)**	
C2-C3-O3-C31	-174.2 (1)	-171.6 (2)*	-178.1 (2)
		175.4 (2)**	
C4-C3-O3-C31	5.8 (2)		1.6 (3)
C3-O3-C31-C32	178.7 (1)		-174.7 (2)
C8-C7-C70-C71/F71	-58.4 (2)	36.6 (3)	-46.2 (3)
C8-C7-C70-C72/F72	61.1(1)	-84.5 (3)	71.6 (3)
C8-C7-C70-C73/F73	-178.9(1)	153.4 (3)	-168.7 (2)
Br1-C11-C12-Br2			179.8 (1)

* for A, C, E, G, I, and L molecules only; ** for B, D, F, H, J, and K molecules only.

Table S3 Hydrogen bond geometry and selected weak interactions (Å, °) in the structure of **5c hydrate** and **6b** at T=100 K and for **6b solvate** at T=120 K

**Identification code 5c hydrate**	D–H	H…A	D...A	∠DHA
C21-H21A…O2W (x, -y+1/2, z-3/2)	0.99	2.55	2.505(2)	163
O1W-H1W1…N10 (-x+1, y-1/2, -z+1/2)	0.85(1)	2.07(1)	2.914(2)	176(2)
O1W-H1W2…O3W (-x+1, -y, -z+1)	0.84(1)	1.98(1)	2.772(2)	156(2)
O2W-H2W1…N5 (x, y, z+1)	0.86(1)	2.03(1)	2.879(2)	175(2)
O2W-H2W2…O3W	0.85(1)	1.97(1)	2.776(2)	157(2)
O3W-H3W1…O2W (-x+1, -y, -z+2)	0.81(1)	1.94(1)	2.723(2)	173(2)
O3W-H3W3…O1W	0.81(1)	1.95(1)	2.761(2)	174(2)
Cg2(x, -y+1/2, z-1/2)…Cg2…Cg2(x, -y+1/2, z+1/2)		3.453	3.453	178.85
π (x, -y+1/2, z-1/2)… π… π (x, -y+1/2, z+1/2)		3.348	-3.348	
off-sets		0.845	0.245	

Ring gravity centres: Cg1 for C1C2C3C4C5C6, Cg2 for C4A-N5-C5A-C9A-N10-C10A, Cg3 for C5A-C6-C7-C8-C9-C9A

**Identification code 6b**	D–H	H…A	D...A	∠DHA
C1A-H1A…F71F (-x+1, -y+1, -z)	0.95	2.51	3.455	172
C20A-H20A…F73E (-x+1, -y+1, -z)	0.99	2.37	3.249	147
C20A-H20B…O2F (x-1, y+1, z)	0.99	2.60	3.507	152
C6A-H6A…F74B	0.95	2.59	3.081	113
C9A-H9A…N5F (-x+1, -y+1, -z)	0.95	2.48	3.424	173
C1B-H1B…F72E (-x+2, -y+1, -z)	0.95	2.54	3.481	169
C9B-H9B…N5E (-x+2, -y+1, -z)	0.95	2.63	3.579	174
C1C-H1C…F71D (-x+1, -y+1, -z)	0.95	2.52	3.460	170
C20C-H20E…F73C (-x+1, -y+1, -z)	0.99	2.39	3.252	145
C20C-H20F…O2B	0.99	2.56	3.469	153
C6C-H6C…F74D	0.95	2.56	3.096	116
C9C-H9C…N5D (-x+1, -y+1, -z)	0.95	2.49	3.436	171
C1D-H1D…F72C (-x+2, -y+1, -z)	0.95	2.56	3.487	167
C1D-H1D…F73D (-x+2, -y+1, -z)	0.95	2.58	3.082	113
C30D-H30G…F71B (-x+1, -y+1, -z)	0.99	2.60	3.228	121
C9D-H9D…N5C (-x+2, -y+1, -z)	0.95	2.58	3.520	171
C1E-H1E…F71B (-x+1, -y+1, -z)	0.95	2.51	3.452	172
C20E-H20I…F73A (-x+1, -y+1, -z)	0.99	2.41	3.266	145
C20E-H20J…O2D	0.99	2.57	3.483	153
C9E-H9E…N5B (-x+1, -y+1, -z)	0.95	2.53	3.468	171
C1F-H1F…F72A (-x+2, -y+1, -z)	0.95	2.53	3.469	170
C1F-H1F…F73B (-x+2, -y+1, -z)	0.95	2.64	3.125	112
C30F-H30K…F71F (-x+2, -y, -z)	0.99	2.62	3.239	121
C9F-H9F…N5A (-x+2, -y+1, -z)	0.95	2.59	3.533	172

C1G-H1G…F71J (-x+1, -y, -z+1)	0.95	2.49	3.435	171
C20G-H20M…F72I (-x+1, -y, -z+1)	0.99	2.39	3.264	146
C20G-H20N…O2K (x, y-1, z)	0.99	2.57	3.460	149
C9G-H9G…N5J (-x+1, -y, -z+1)	0.95	2.54	3.465	166
C1H-H1H…F71I (-x, -y, -z+1)	0.95	2.63	3.549	162
C1H-H1H…F72J (-x, -y, -z+1)	0.95	2.58	3.173	121
C30H-H30O…F71H (-x+1, -y, -z+1)	0.99	2.62	3.240	120
C9H-H9H…N5I (-x, -y, -z+1)	0.95	2.65	3.591	173
C1I-H1I…F71H (-x+1, -y, -z+1)	0.95	2.46	3.403	173
C20I-H20Q…F72G (-x+1, -y, -z+1)	0.99	2.41	3.286	148
C20I-H20R…O2H	0.99	2.55	3.434	149
C9I-H9I…N5H (-x+1, -y, -z+1)	0.95	2.42	3.356	170
C1J-H1J…F71G (-x, -y, -z+1)	0.95	2.47	3.415	173
C30J-H30S…F73K (-x+1, -y+1, -z+1)	0.99	2.57	3.266	127
C9J-H9J…N5G (-x, -y, -z+1)	0.95	2.57	3.521	174
C1K-H1K…F71K (-x+1, -y+1, -z+1)	0.95	2.60	3.116	114
C1K-H1K…F73L (-x+1, -y+1, -z+1)	0.95	2.55	3.484	169
C9K-H9K…N5L (-x+1, -y+1, -z+1)	0.95	2.62	3.568	173
C1L-H1L…F72K (-x+2, -y+1, -z+1)	0.95	2.48	3.415	170
C20L-H20W…F72L (-x+2, -y+1, -z+1)	0.99	2.38	3.273	149
C20L-H20$…O2J (x+1, y, z)	0.99	2.59	3.475	149
C9L-H9L…N5K (-x+2, -y+1, -z+1)	0.95	2.49	3.433	170

**Identification code 6b solvate**	D–H	H…A	D...A	∠DHA
C11-H11A…N5 (-x+2, -y+1, -z+1)	0.99	2.64	3.556(3)	153
C12-H12A…O2	0.99	2.47	3.396(3)	155
C12-H12A…O3	0.99	2.61	3.463(3)	144
O2…O3			2.503	
C1-H1…Br1 (-x+1, -y, -z+1)	0.95	3.26	4.213	176
C4-H4…Br1 (-x+1, -y+1, -z+2)	0.95	3.14	4.070	165
C11-H11B…Br1 (-x+2, -y, -z+1)	0.99	3.23	3.964	132
C9-H9…Br2 (-x+1, -y, -z+1)	0.95	3.31	4.225	163
C32-H32C… Br2	0.98	3.28	4.232	166
C8-H8…F72 (-x+1, -y+1, -z+2)	0.95	2.59	3.369	139
C12-Br2…F73 (-x+2, -y+1, -z+1)	1.901	3.484	5.157	141
C22-H23C…Cg2 (-x+1, -y. –z+1)	0.98	2.72	3.522	139
Cg1…Cg3 (-x+1, -y+1, -z+1)			3.698	
Cg2…Cg2 (-x+1, -y+1, -z+1)			3.647	
π2…π2 (-x+1, -y+1, -z+1), off-set			3.335, 1.476	
Cg3…Cg1 (-x+1, -y+1, -z+1)			3.698	

Ring gravity centres: Cg1 for C1C2C3C4C4AC10A, Cg2 for C4AN5C5AC9AN10C10A, Cg3 for C5AC6C7C8C9C9A

Figures for 5c hydrate

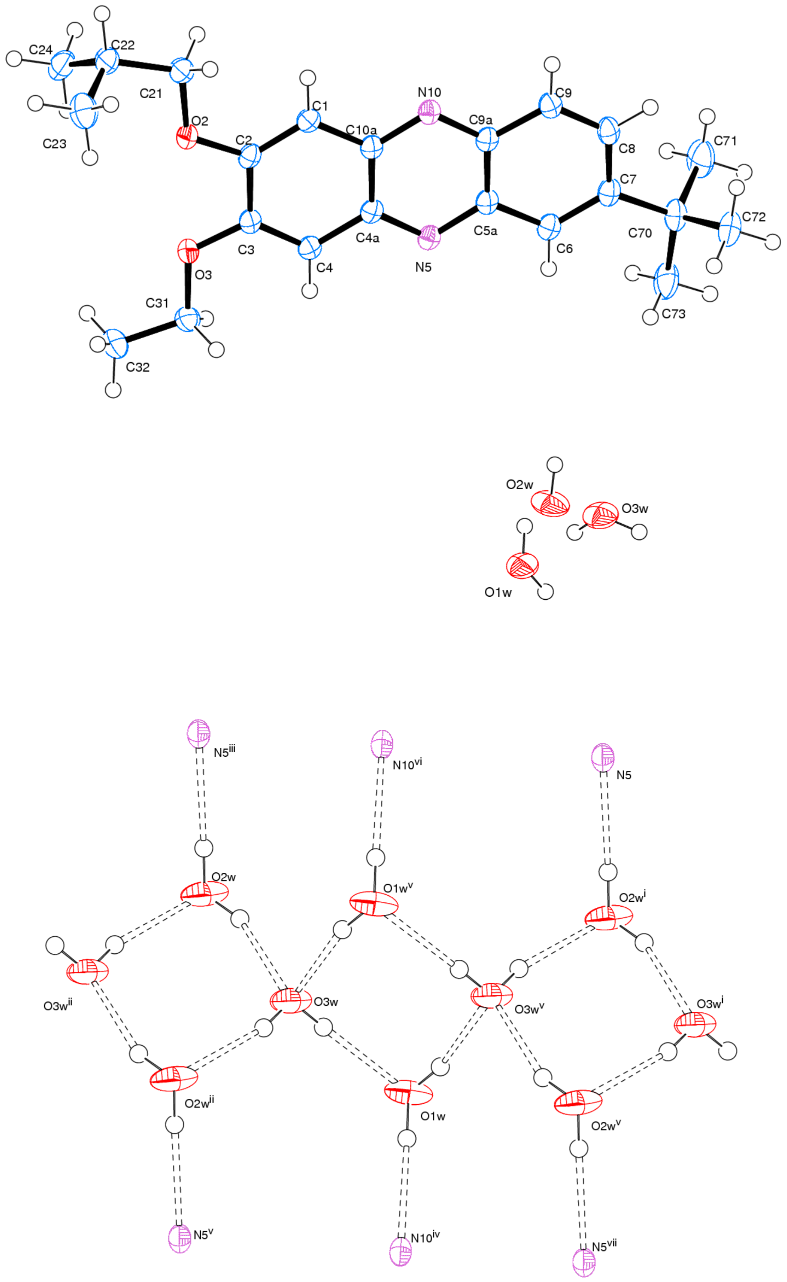

Figure S153. Top: Contents of the asymmetric unit cell in the case of **5c hydrate** with the atom numbering scheme. The non-hydrogen atoms: C, N, and O, are presented by the displacement ellipsoids at 50 % probability level in blue, magenta, and red colours, respectively. The H atoms are shown as spheres in an arbitrary scale. In this particular crystal structure, the occupancy factors of O1W, O2W and O3W water molecules are 0.870(6), 0.868(6) and 0.835(6), respectively (in other crystals the values might be lower, e.g. 0.752(6), 0.755(6) and 0.696(6), respectively). Bottom: The hydrogen bond system formed by the water molecules and extended along [001]. The geometry of the hydrogen bonds is given in Table S3. i) x, y, z-1; ii) –x+1, -y, -z+2; iii) x, y, z+1; iv) –x+1, -y-1/2, -z+1/2; v) -x+1, -y, -z+1; vi) x, -y+1/2, z+1/2; vii) –x+1, -y, -z.

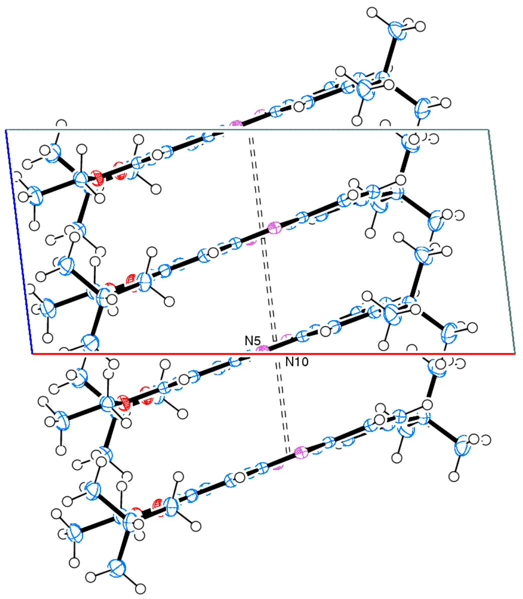

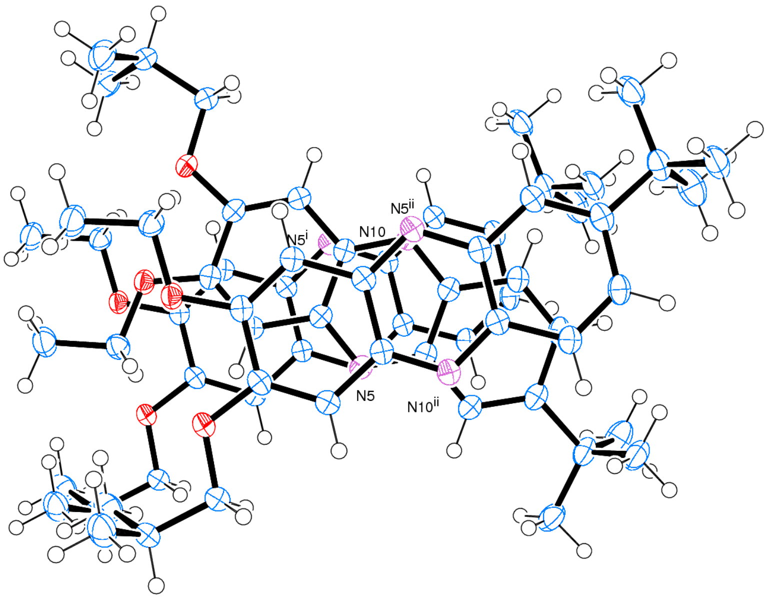

Figure S154. Top: Stacking of the **5c** molecules in [001] direction viewed along [010]. The relation of the pyrazine ring gravity centres Cg2^{ii}(x, -y+1/2, z-1/2)…Cg2 [C4aN5C5aC9aN10C10a]…Cg2^{i}(x, -y+1/2, z+1/2) = 3.453, 3.453 Å, 178.9°; Cg2^{ii}…π…Cg2^{i}= +3.348 and -3.348 Å, with the offset 0.845 and 0.245 Å, respectively. Bottom: The view of the molecular column perpendicular to the pyrazine ring.

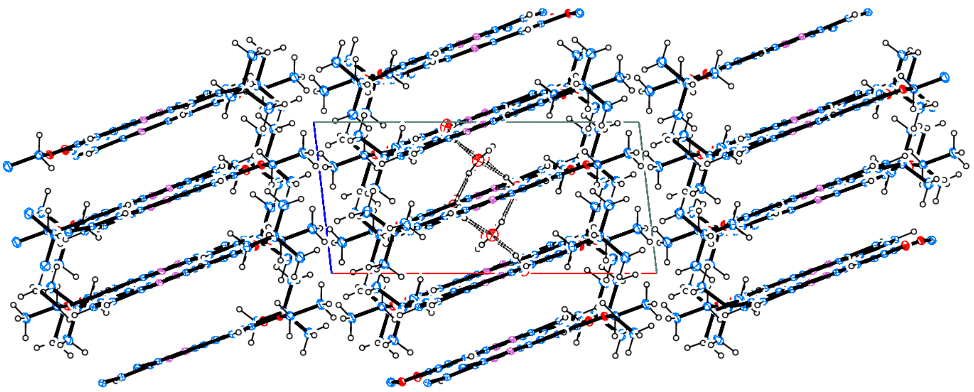

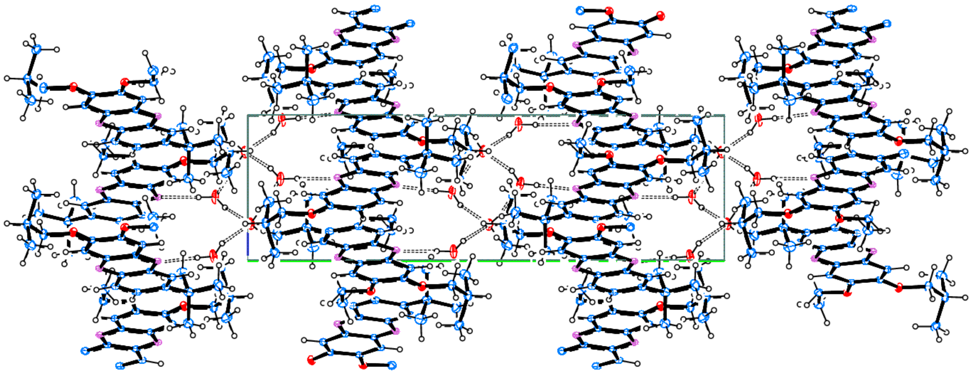

Figure S155. Top: Packing of the molecules in **5c hydrate** projected onto (010). Bottom: Packing of the molecules viewed along [100]. The lattice direction *a*, *b*, *c* are marked by red, green and blue lines, respectively. Hydrogen bonds are marked by dashed lines.

Figures for pure 6b and 6b solvate

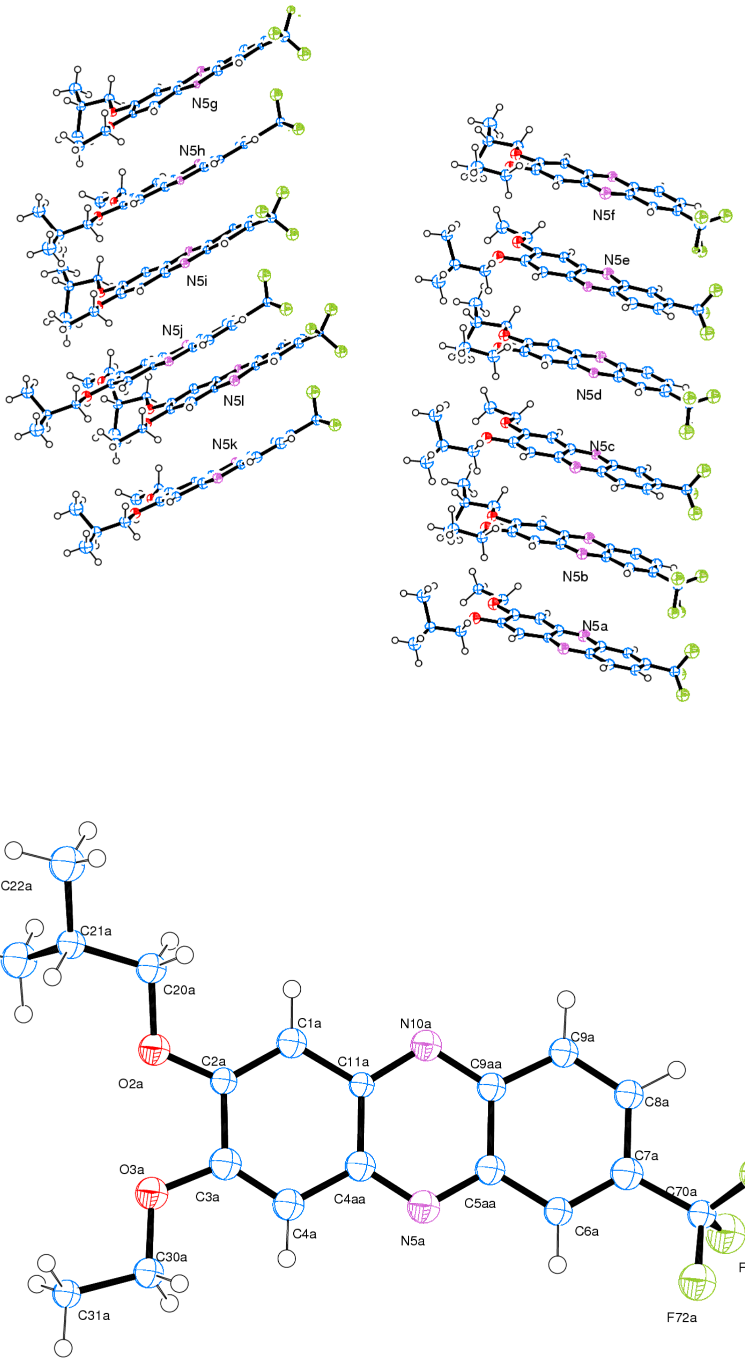

Figure S156. Top: Contents of the asymmetric unit cell in the case of pure **6b** with the twelve symmetrically independent molecules marked by letters A-L. Bottom: The adopted atom numbering scheme similar like used for **5c** shown for the A molecule. The non-hydrogen atoms: C, N, O, and F, are presented by spheres at 50 % probability level in blue, magenta, red and green colours, respectively. The H atoms are shown as spheres in an arbitrary scale.

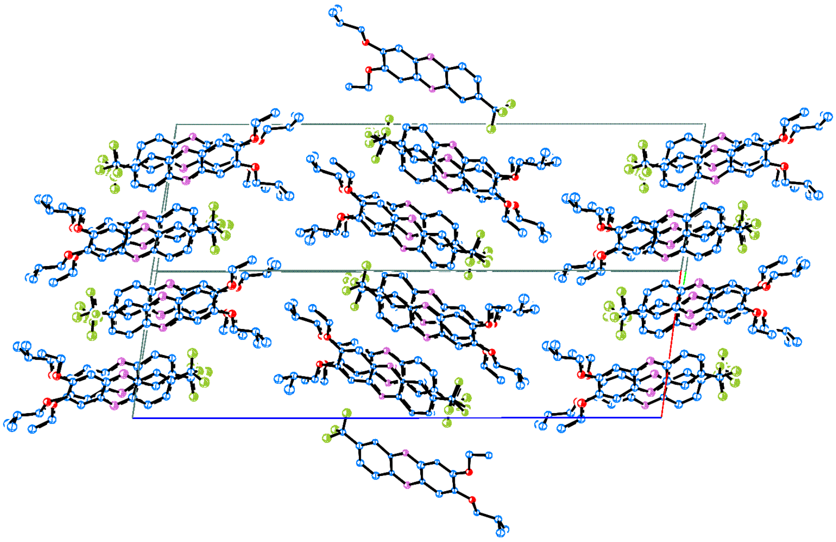

Figure S157. Packing of the molecules in the triclinic crystal structure of pure **6b** viewed along [-101] according to the refinement of the atom positions with isotropic displacement parameters and shown with H-atoms omitted for clarity. Unit cell directions a, b and c are marked by red, green, and blue lines, respectively.

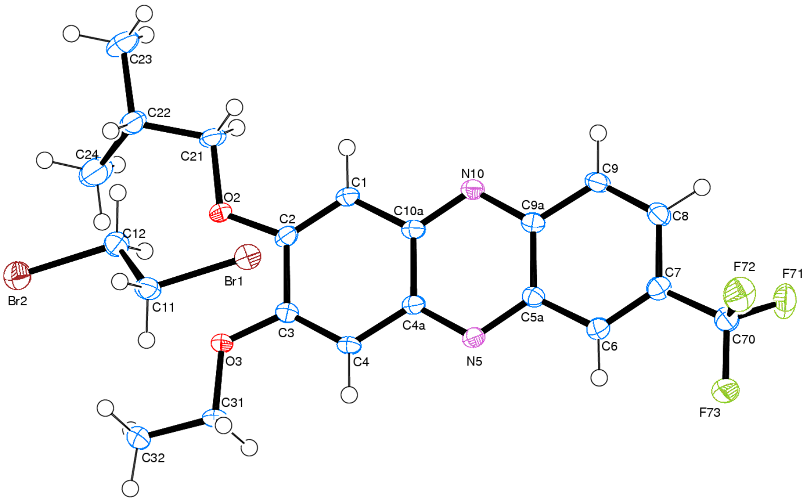

Figure S158. Contents of the asymmetric unit cell in the case of **6b solvate** with the molecule **6b** and 1,2-dibromoetane with the atom numbering scheme. The atoms: C, N, O, F, and Br are presented by the displacement ellipsoids at 50 % probability level marked by blue, magenta, red, green, and brown colours, respectively. The H atoms are shown as spheres in an arbitrary scale.

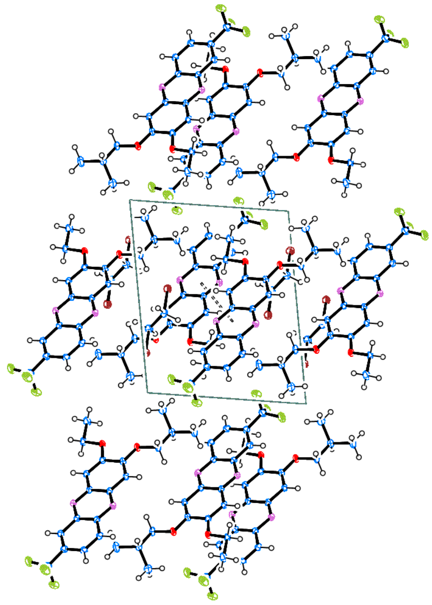

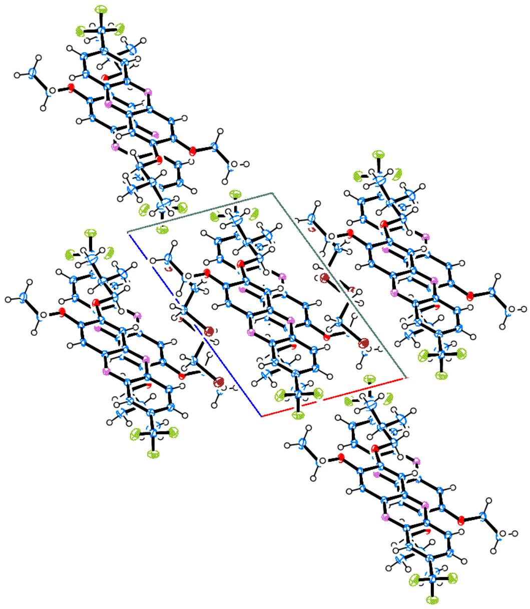

Figure S159. Top: Packing of the molecules in **6b solvate** projected along [100]. Bottom: Packing of the molecules viewed along [010]. The lattice direction *a*, *b*, *c* are marked by red, green and blue lines, respectively. The distance of pyrazine ring gravity centres of the molecules forming the dimer at (1/2,1/2,1/2) is marked by dashed lines with Cg2…Cg2 (-x+1, -y+1, -z+1) and π2…π2 (-x+1, -y+1, -z+1) distances of 3.647 and 3.335 Å, respectively.
